# Electronic Communication in Binuclear Osmium- and Iridium-Polyhydrides

**DOI:** 10.1021/acs.inorgchem.0c03680

**Published:** 2021-02-05

**Authors:** Lara Cancela, Miguel A. Esteruelas, Javier Galbán, Montserrat Oliván, Enrique Oñate, Andrea Vélez, Juan C. Vidal

**Affiliations:** † Departamento de Química Inorgánica − Instituto de Síntesis Química y Catálisis Homogénea (ISQCH) − Centro de Innovación en Química Avanzada (ORFEO−CINQA), Universidad de Zaragoza − CSIC, 50009 Zaragoza, Spain; ‡ Departamento de Química Analítica, Facultad de Ciencias − Instituto de Nanociencia de Aragón (INA-ICMA), Universidad de Zaragoza, 50009 Zaragoza, Spain; § Grupo de Espectroscopia Analítica y Sensores (GEAS) − Instituto de Investigación en Ciencias Ambientales de Aragón (IUCA), Universidad de Zaragoza, 50009 Zaragoza, Spain

## Abstract

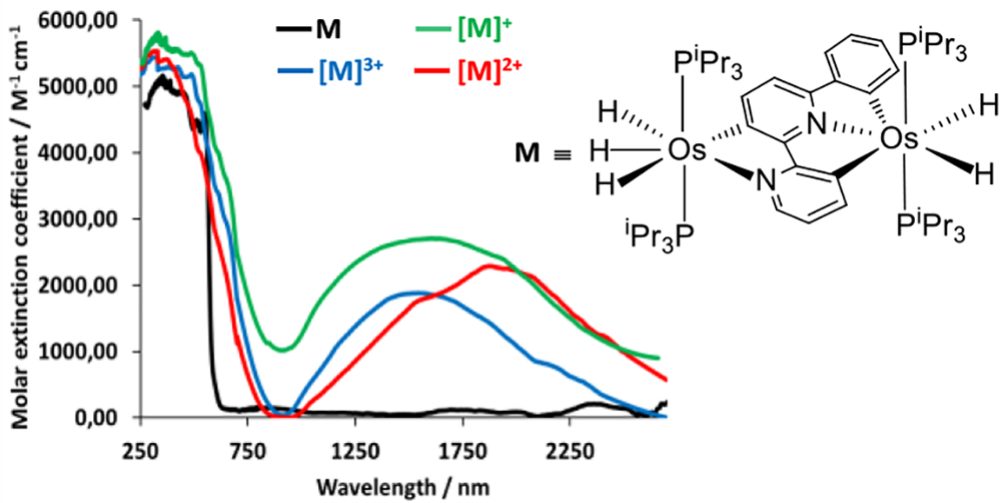

Reactions of polyhydrides OsH_6_(P^i^Pr_3_)_2_ (**1**) and IrH_5_(P^i^Pr_3_)_2_ (**2**) with rollover cyclometalated hydride complexes have been investigated in order to explore the influence of a metal center on the MH_*n*_ unit of the other in mixed valence binuclear polyhydrides. Hexahydride **1** activates an *ortho*-CH bond of the heterocyclic moiety of the trihydride metal–ligand compounds OsH_3_{κ^2^-*C*,*N*-[C_5_RH_2_N-py]}(P^i^Pr_3_)_2_ (R = H (**3)**, Me (**4**), Ph (**5**)). Reactions of **3** and **4** lead to the hexahydrides (P^i^Pr_3_)_2_H_3_Os{μ-[κ^2^-*C*,*N*-[C_5_RH_2_N-C_5_H_3_N]-*N*,*C*-κ^2^]}OsH_3_(P^i^Pr_3_)_2_ (R = H (**6**), Me (**7**)), whereas **5** gives the pentahydride (P^i^Pr_3_)_2_H_3_Os{μ-[κ^2^-*C*,*N*-[C_5_H_3_N-C_5_(C_6_H_4_)H_2_N]-*C*,*N*,*C*-κ^3^]}OsH_2_(P^i^Pr_3_)_2_ (**8**). Pentahydride **2** promotes C—H bond activation of **3** and the iridium-dihydride IrH_2_{κ^2^-*C*,*N*-[C_5_H_3_N-py]}(P^i^Pr_3_)_2_ (**9**) to afford the heterobinuclear pentahydride (P^i^Pr_3_)_2_H_3_Os{μ-[κ^2^-*C*,*N*-[C_5_H_3_N-C_5_H_3_N]-*N*,*C*-κ^2^]}IrH_2_(P^i^Pr_3_)_2_ (**10**) and the homobinuclear tetrahydride (P^i^Pr_3_)_2_H_2_Ir{μ-[κ^2^-*C*,*N*-[C_5_H_3_N-C_5_H_3_N]-*N*,*C*-κ^2^]}IrH_2_(P^i^Pr_3_)_2_ (**11**), respectively. Complexes **6**–**8** and **11** display HOMO delocalization throughout the metal–heterocycle-metal skeleton. Their sequential oxidation generates mono- and diradicals, which exhibit intervalence charge transfer transitions. This notable ability allows the tuning of the strength of the hydrogen–hydrogen and metal–hydrogen interactions within the MH_*n*_ units.

## Abstract 2

The influence of a metal center on the MH_*n*_ unit of the other in binuclear osmium- and iridium-polyhydride complexes bearing rollover cyclometalated bipyridines as a bridging ligand has been studied.

## Introduction

The hydrogen atoms of the MH_*n*_ units of L_*m*_MH_*n*_ transition metal polyhydride complexes interact with one another and with the metal center, forming Kubas type dihydrogens (d_H–H_ = 0.8–1.0 Å), elongated dihydrogens (d_H–H_ = 1.0–1.3 Å), compressed hydrides (d_H–H_ = 1.3–1.6 Å), or classical hydrides (d_H–H_ ≥ 1.6 Å). These interactions are governed by the electron density of the metal center, which is regulated by the coligands L_*m*_.^1^ Thus, the ability of such compounds to reversibly release the H_2_ molecule requires L_*m*_ ligands, which are able to modify the electron density of the metal center, in order to allow reversible changes in the inner interactions of the MH_*n*_ units. The design of such ligands is certainly a challenge of the first magnitude.

An attractive approach to the solution of this challenge is the use transition metal complexes, which should display frontier orbitals involving substantial mixing with a π-ligand backbone, whereas such a ligand should also bear atoms with free electrons. The coordination of this metal–ligand to an MH_*n*_ unit would generate species with frontier orbitals delocalized between the two metal centers connected by the π-linker. Thus, the metals can be viewed as being electronically coupled and therefore the changes in electron density at one site should perturb the electron density at the other.^2^ The search for efficient π-linkers (bridging ligands), which promote the cooperative effect between the redox active centers through electronic coupling pathways, is central for success. Unsaturated carbon chains,^3^ aromatic polycycles,^3c,4^ aromatic *N*-heterocycles,^5^ bisdioxolenes,^6^ bisdithiolenes,^7^ dithiolate,^8^ cyanide,^9^ and cyanamides^10^ have been mainly employed so far, as bridging ligands, to provide electronical coupling between metals.

The interactions between the metal centers have been grouped into three categories, according to the Robin–Day classification: weak, moderate, and strong.^11^ Compounds displaying weak interaction form class I, and their redox centers mostly behave as separated sites. On the other hand, strong interaction affords a complete electron density delocalization, and complexes with this ability are grouped as class III. Species exhibiting moderate interaction between their redox centers constitute class II.^3d,12^ The degree of interaction is efficiently assessed by means of the analysis of the intervalence charge transfer (IVCT) band in the UV–vis–NIR spectra on the basis of the Marcus–Hush theory.^13^ At the electrochemistry level, the redox potential separation between successive redox processes is also a frequently used measure, although it often presents misinterpretation issues.^14^

**Scheme 1:**
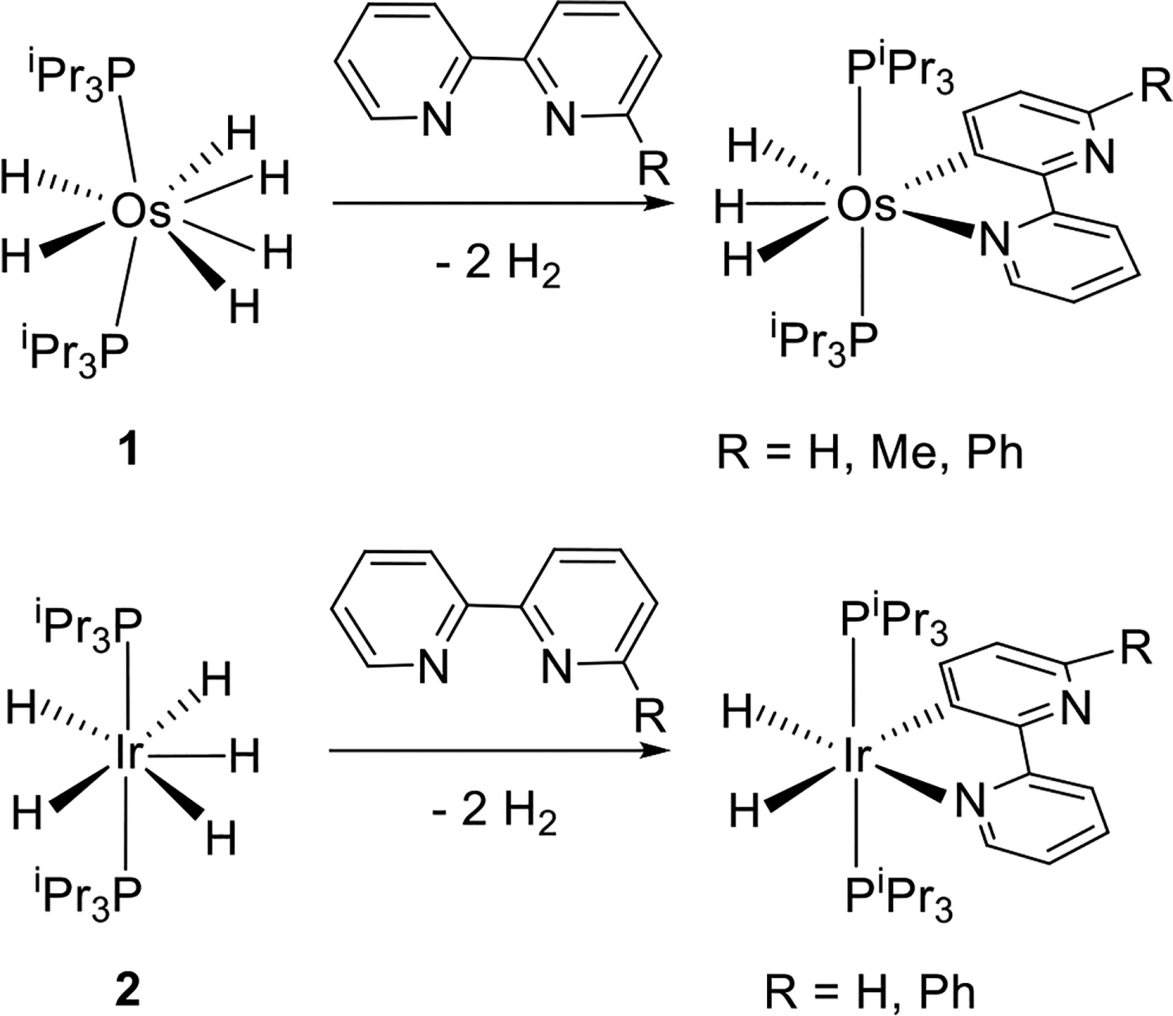
C—H Bond Activation of 2,2′-Bipyridines

We have recently shown that the platinum group polyhydrides OsH_6_(P^i^Pr_3_)_2_ (**1**) and IrH_5_(P^i^Pr_3_)_2_ (**2**) promote the activation of C—H bonds of the rings of 2,2′-bipyridines and related heterocycles to afford rollover cyclometalated trihydride- and dihydride-derivatives (Scheme 1),^15^ in agreement with the ability of polyhydrides of the platinum group metals to activate σ-bonds^1f^ and in particular the *d*^2^-hexahydride OsH_6_(P^i^Pr_3_)_2_.^16^ In the context of the rollover cyclometalation, we noted that in a few cases the resulting ligands underwent an additional cylometalation promoted by a second metal complex, to form binuclear species bearing a bridging rollover bis-cyclometalated heterocycle.^17^ Although evidence of the ability of these bridges to provide electronic coupling pathways has not been reported, these findings inspired us to prepare osmium- and iridium-polyhydrides with this class of bridging ligands and to use them as models to check our proposed approach toward the control of reversible changes in the existing interactions within the MH_*n*_ units.

This paper proves that rollover cyclometalated 2,2′-bipyridine heterocycles provide electronic coupling pathways between the metals of (P^i^Pr_3_)_2_H_n_Os(μ-L)OsH_n_(P^i^Pr_3_)_2_ (*n* = 2 or 3) and (P^i^Pr_3_)_2_H_2_Ir(μ-L)IrH_2_(P^i^Pr_3_)_2_ complexes and that changes in the electron density of a metal center influence the inner interactions of the MH_*n*_ unit of the other.

## Results and Discussion

### Metal–Ligand C–H Bond Activation

**Scheme 2:**
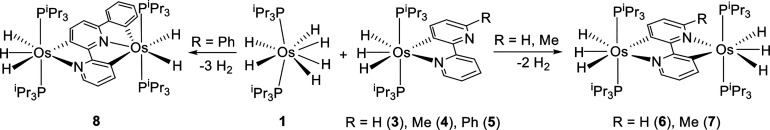
Formation of Binuclear Species **6**, **7**, and **8**

Osmium-hexahydride complex **1** is able to activate C—H bonds of the rollover cyclometalated trihydride derivatives OsH_3_{κ^2^-*C*,*N*-[C_5_RH_2_N-py]}(P^i^Pr_3_)_2_ (R = H (**3**), Me (**4**), Ph (**5**)) in toluene under reflux (Scheme 2) and in agreement with its ability to promote σ-bond activation reactions. Complexes **3** and **4** afford the binuclear-hexahydride compounds (P^i^Pr_3_)_2_H_3_Os{μ-[κ^2^-*C*,*N*-[C_5_RH_2_N–C_5_H_3_N]-*N*,*C*-κ^2^]}OsH_3_(P^i^Pr_3_)_2_ (R = H (**6**) Me (**7**)), as a result of the coordination of the free nitrogen atom of the rollover cyclometalated heterocycle and the *ortho*-CH bond activation of the other ring, whereas the reaction with the phenyl-derivative **5** leads to the pentahydride (P^i^Pr_3_)_2_H_3_Os{μ-[κ^2^-*C*,*N*-[C_5_H_3_N-C_5_(C_6_H_4_)H_2_N]-*C*,*N*,*C*-κ^3^]}OsH_2_(P^i^Pr_3_)_2_ (**8**). In contrast to **6** and **7**, complex **8** bears two different osmium(IV) OsH_*n*_(P^i^Pr_3_)_2_ moieties, OsH_3_(P^i^Pr_3_)_2_ and OsH_2_(P^i^Pr_3_)_2_. In this case, the bridging ligand acts in a dual manner: monoanionic *C*,*N*-chelate with OsH_3_(P^i^Pr_3_)_2_ and dianionic *C*,*N*,*C*-pincer with OsH_2_(P^i^Pr_3_)_2_. The difference is a consequence of the hexahydride being also able to activate the phenyl substituent of the rollover cyclometalated heterocycle of **5**. The three binuclear products can be also prepared by treatment of **1** with 0.5 equiv of the 2,2′-bipyridine. Both methods, via intermediates **3**–**5** and the one-pot synthesis procedures, afford the quantitative formation of the binuclear species, which were isolated as orange solids in about 80% yield. Complexes **6** and **8** were characterized by X-ray diffraction analysis.

**Figure 1:**
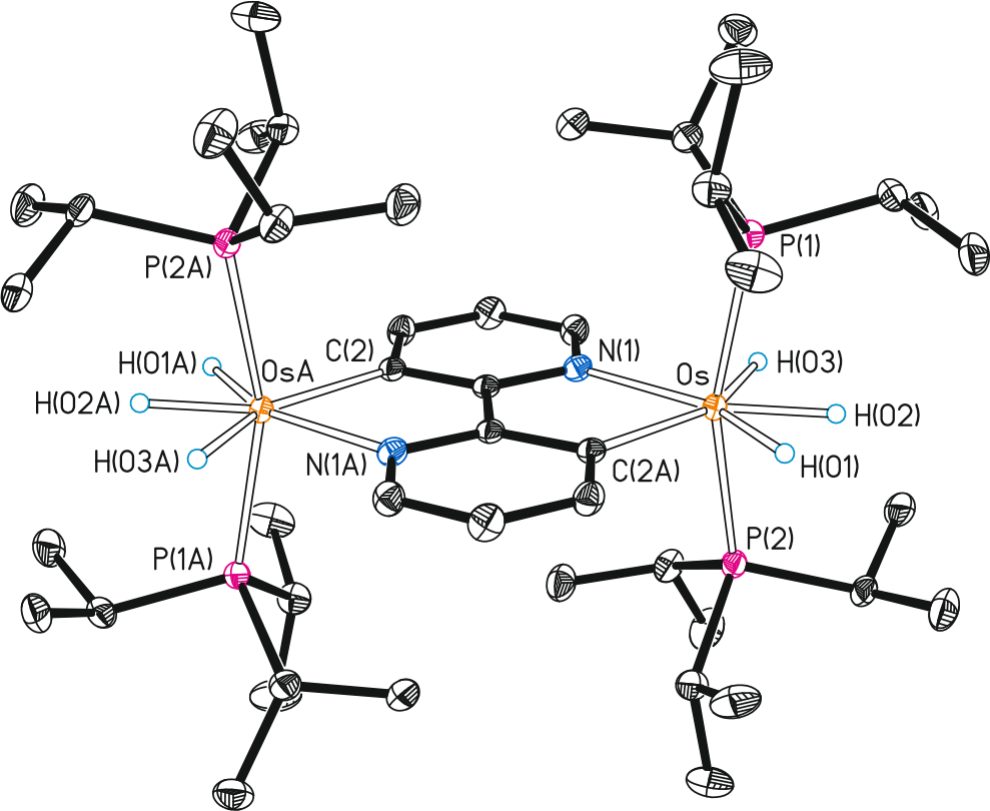
Molecular diagram of complex **6** (ellipsoids shown at 50% probability). All hydrogen atoms (except the hydrides) are omitted for clarity. Selected bond distances (Å) and angles (deg): Os—P(1) = Os(A)—P(1A) = 2.3422(6), Os—P(2) = Os(A)—P(2A) = 2.3414(6), Os—C(2A) = Os(A)—C(2) = 2.144(2), Os—N(1) = Os(A)—N(1A) = 2.1665(18); P(1)—Os—P(2) = P(2A)—Os(A)—P(1A) = 160.82(2), N(1)—Os-C(2A) = N(1A)—Os(A)–C(2) = 76.14(7).

Figure 1 shows the structure of **6**, which can be described as two equivalent OsH_3_(P^i^Pr_3_)_2_ units linked by a rollover bis-cyclometalated 2,2′-bipyridine. The coordination polyhedron around each osmium atom is the typical pentagonal bipyramid for osmium(IV) OsH_3_(Y-X)(P^i^Pr_3_)_2_ species^18^ with axial phosphines (P(1)–Os-P(2) = P(2A)—Os(A)—P(1A) = 160.82(2)°), whereas the hydride ligands lie at the joint base of the bipyramid coplanar to the heterocycle. The Os—N and Os—C bond lengths of 2.1665(18) and 2.144(2) Å are similar to those of the precursor **3**.^15^ In agreement with the high symmetry of the molecule, the ^31^P{^1^H} NMR spectrum of this compound in toluene-*d*_8_ displays a singlet at 23.1 ppm for the four equivalent phosphines. In the ^1^H NMR spectrum, the most noticeable feature is the hydride resonances, which appear between −5 and −13 ppm displaying the typical behavior observed for the inequivalent hydrides of OsH_3_(Y-X)(P^i^Pr_3_)_2_ complexes, involved in a thermally activated site exchange process.^18^ The ^31^P{^1^H}, ^1^H, and ^13^C{^1^H} NMR spectra of **7** in toluene-*d*_8_ reflect the asymmetry imposed by the methyl substituent of the heterocycle. Thus, in contrast to **6** the ^31^P{^1^H} NMR spectrum shows two singlets at 22.7 and 21.4 ppm, whereas resonances corresponding to inequivalent OsH_3_(P^i^Pr_3_)_2_ units are observed between −5 and −14 ppm in the ^1^H NMR spectrum. The ^13^C{^1^H} NMR spectrum displays two triplets (^2^*J*_C–P_ ≈ 6 Hz) at 173.9 and 168.9 ppm for the inequivalent metalated carbon atoms.

**Figure 2:**
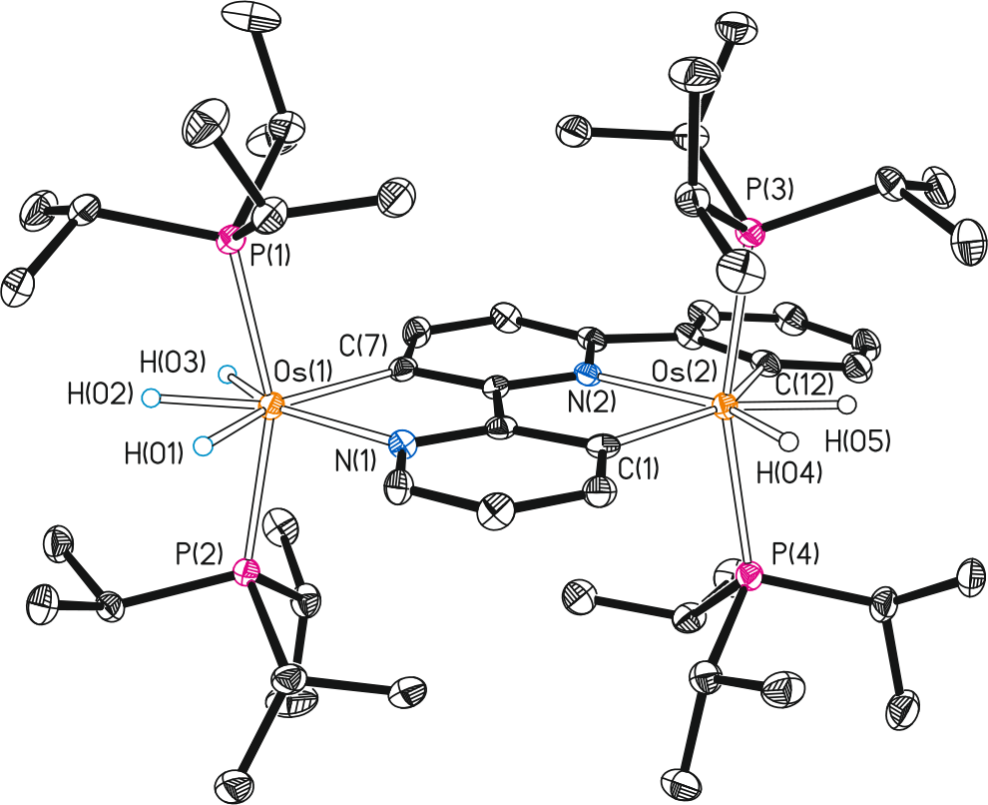
Molecular diagram of complex **8** (ellipsoids shown at 50% probability). All hydrogen atoms (except the hydrides) are omitted for clarity. Selected bond distances (Å) and angles (deg): Os(1)—P(1) = 2.3415(8), Os(1)—P(2) = 2.3418(8), Os(2)—P(3) = 2.3752(8), Os(2)—P(4) = 2.3778(8), Os(1)—N(1) = 2.185(2), Os(2)—N(2) = 2.121(2), Os(1)—C(7) = 2.143(3), Os(2)—C(1) = 2.159(3), Os(2)—C(12) = 2.137(3); P(1)—Os(1)—P(2) = 158.86(3), P(3)—Os(2)—P(4) = 164.05(3), N(1)—Os(1)—C(7) = 76.41(10), C(1)—Os(2)—N(2) = 75.77(10), C(12)—Os(2)—N(2) = 74.89(11), C(1)—Os(2)—C(12) = 150.41(12).

The structure of **8** (Figure 2) proves the dual coordination of the heterocycle in this complex, *C*,*N*-chelate to a metal center (Os(1)) and *C*,*C*,*N*-pincer to the other (Os(2)). The coordination polyhedron around both metal centers can be also idealized as a pentagonal bipyramid. However, there are significant differences between the bipyramids, which are associated with the acting fashion of the bridging ligand. The polyhedron around Os(1) resembles that of **6** with a P(1)—Os(1)—P(2) angle of 158.86(3)°. Phosphine ligands attached to Os(2) also occupy the axial positions of the bipyramid, forming a P(3)—Os(2)—P(4) angle of 164.05(3)°, whereas the pincer lies at the perpendicular joint base, coplanar to the hydride ligands, acting with a C(1)—Os(2)—C(12) angle of 150.41(12)°, which slightly deviates from the ideal value of 144°. The Os(1)—C(7) and Os—N(1) distances of 2.143(3) and 2.185(2) Å are similar to those found in **6**, whereas the Os(2)—C(1), Os(2)—C(12), and Os(2)—N(2) bond lengths compare well with the observed ones for osmium compounds bearing *C*,*N*,*C*-pincer ligands.^16c,15,19^^31^P{^1^H}, ^1^H, and ^13^C{^1^H} NMR spectra of **8** in dichloromethane-*d*_2_ are consistent with the structure shown in Figure 2. Thus, the ^31^P{^1^H} NMR spectrum contains two singlets at 24.1 and 1.8 ppm, assigned to the OsH_3_(P^i^Pr_3_)_2_ and OsH_2_(P^i^Pr_3_)_2_ units, respectively. In the ^1^H NMR spectrum, the resonances of the OsH_3_(P^i^Pr_3_)_2_ unit display the typical pattern for the cyclometalated OsH_3_(Y-X)(P^i^Pr_3_)_2_ species, between −6 and −14 ppm, along with two temperature invariant doublets (^2^*J*_H–H_ = 11.3 Hz) of triplets (^2^*J*_H–P_ = 15.1 and 17.2 Hz) at −8.48 and −9.19 ppm corresponding to the hydride ligands of the OsH_2_(P^i^Pr_3_)_2_ unit. The ^13^C{^1^H} NMR spectrum shows three triplets (^2^*J*_C–P_ = 6.1–8.5 Hz) at 169.9, 168.0, and 165.5 ppm due to the metalated carbon atoms.

**Scheme 3:**
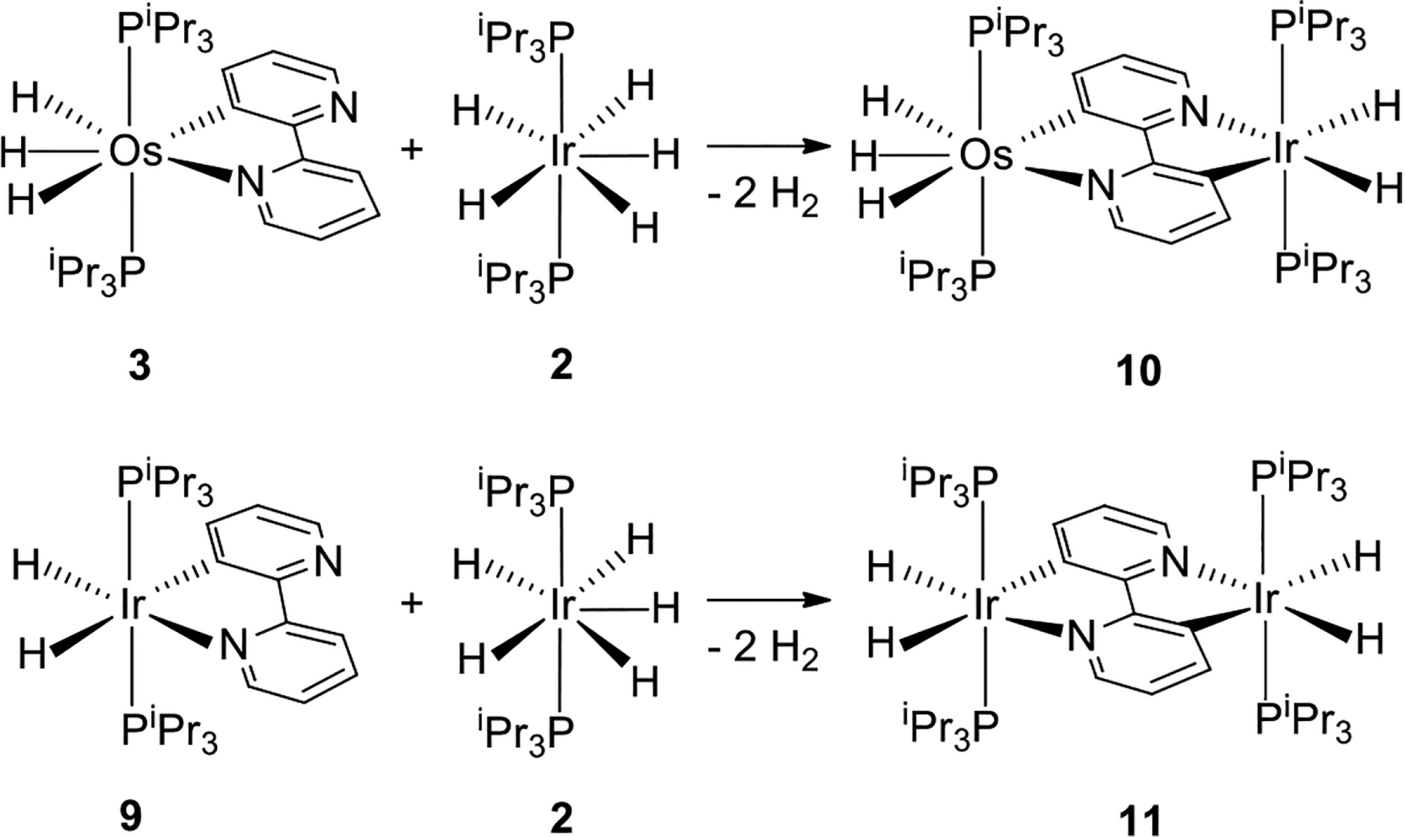
Formation of Complexes 10 and 11

The success of the reactions shown in Scheme 2 encouraged us to extend this synthetic methodology, involving polyhydride-mediated sequential rollover cyclometalation of 2,2′-bipyridines to other polyhydrides and to study its utility to generate heterobinuclear derivatives. Thus, we decided to also investigate the C—H bond activation of **3** and the iridium-dihydride IrH_2_{κ^2^-*C*,*N*-[C_5_H_3_N-py]}(P^i^Pr_3_)_2_ (**9**), promoted by the iridium-pentahydride complex **2** (Scheme 3). Treatment of toluene solutions of **3** with 1.0 equiv of **2** under reflux for 16 h leads to the heterobinuclear pentahydride (P^i^Pr_3_)_2_H_3_Os{μ-[κ^2^-*C*,*N*-[C_5_H_3_N-C_5_H_3_N]-*N*,*C*-κ^2^]}IrH_2_(P^i^Pr_3_)_2_ (**10**), which was isolated as an orange solid in 68% yield. Under the same conditions, the reaction of **9** and **2** affords the homobinuclear tetrahydride (P^i^Pr_3_)_2_H_2_Ir{μ-[κ^2^-*C*,*N*-[C_5_H_3_N-C_5_H_3_N]-*N*,*C*-κ^2^]}IrH_2_(P^i^Pr_3_)_2_ (**11**), which can be also prepared by treatment of **2** with 0.5 equiv of 2,2′-bipyridine. Complex **11** was isolated as a yellow solid in almost quantitative yield.

**Figure 3:**
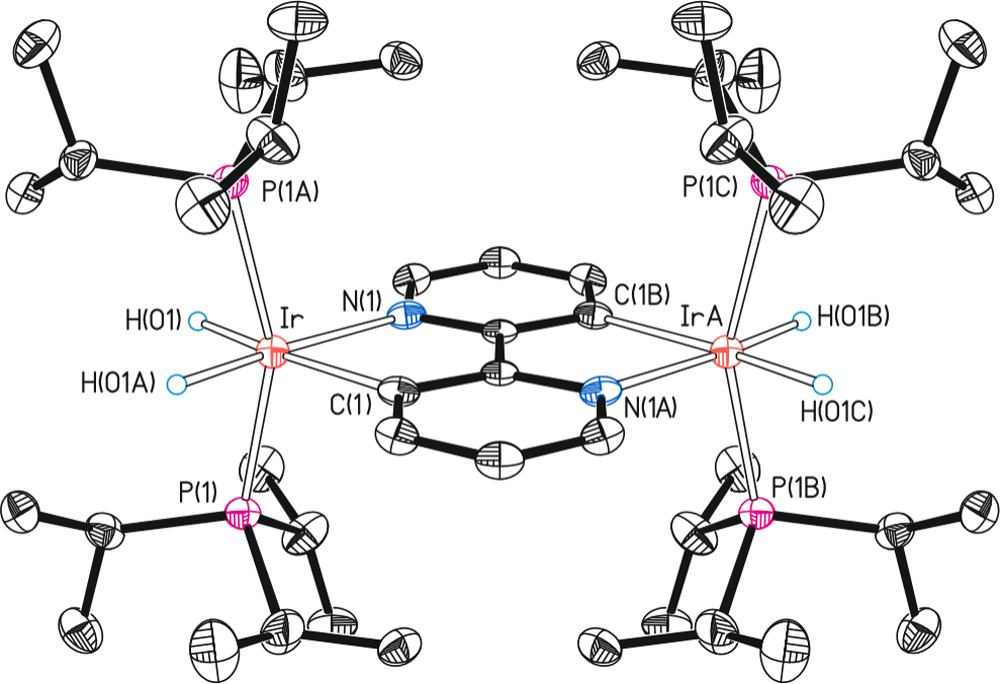
Molecular diagram of complex **11** (ellipsoids shown at 50% probability). All hydrogen atoms (except the hydrides) are omitted for clarity. Selected bond distances (Å) and angles (deg): Ir—P(1) = Ir—P(1A) = Ir(A)—P(1B) = Ir(A)—P(1C) = 2.2988(5), Ir—N(1) = Ir(A)—N(1A) = 2.1329(17), Ir—C(1) = Ir(A)—C(1B) = 2.1330(17); P(1)—Ir—P(1A) = P(1B)—Ir(A)—P(1C) = 156.94(3), N(1)—Ir—C(1) = N(1A)—Ir(A)—C(1B) = 78.47(9).

The ^31^P{^1^H}, ^1^H, and ^13^C{^1^H} NMR spectra of **10** in toluene-*d*_8_ strongly support the structure proposed for this compound, in Scheme 3. The ^31^P{^1^H} NMR spectrum contains two singlets at 30.3 and 22.4 ppm, one for each group of equivalent phosphines, whereas the ^1^H NMR spectrum is consistent with the presence in the complex of two different classes of hydride ligands. In agreement with **8**, hydrides attached to osmium give rise to resonances displaying the typical temperature-dependent pattern for a cyclometalated OsH_3_(XY)(P^i^Pr_3_)_2_ species, between −5 and −13 ppm while hydrides of the IrH_2_(P^i^Pr_3_)_2_ unit generate two temperature invariant doublets (^2^*J*_H–H_ = 3.6 Hz) of triplets (^2^*J*_H–P_ = 18.9 Hz) at −12.93 and −21.93 ppm. In the ^13^C{^1^H} NMR spectrum, the resonances due to the metalated carbon atoms appear at 173.4 and 163.2 ppm, as triplets with C–P coupling constants of about 6 Hz. Complex **11** was characterized by X-ray diffraction analysis. Figure 3 shows a view of the structure. The molecule is formed by two chemically equivalent IrH_2_(P^i^Pr_3_)_2_ moieties connected to each other through a rollover bis-cyclometalated 2,2′-bipyridine linker. It is a *d*^6^–*d*^6^ counterpart of the *d*^4^*–d*^4^ complex **6** and the *d*^4^*–d*^6^ derivative **10**. The coordination polyhedron around each iridium center is the expected octahedron with trans phosphines (P—Ir—P = 156.94(3)°). In agreement with its structure, the ^31^P{^1^H} NMR spectrum of this highly symmetrical molecule shows a singlet at 29.9 ppm for the four equivalent phosphines, the ^1^H NMR spectrum contains two doublets (^2^*J*_H–H_ = 4.1 Hz) of triplets (^2^*J*_H–P_ = 21.2 and 19.3 Hz) at −12.93 and −22.00 ppm for the inequivalent hydrides of the equivalent IrH_2_(P^i^Pr_3_)_2_ units, whereas the ^13^C{^1^H} NMR spectrum displays a triplet (^2^*J*_C–P_ = 6.5 Hz) at 163.6 for the equivalent metalated carbon atoms.

### Frontier Orbitals and Photophysical Properties

The UV–vis spectra for 1 × 10^–4^ M solutions of the mononuclear precursors **3**–**5** and **9** and binuclear derivatives **6**–**8**, **10**, and **11** in 2-methyltetrahydrofuran (MeTHF) were recorded. Figure 4 shows the spectra of **10** and their mononuclear building blocks **3** and **9,** whereas the rest are shown in Figures S19–S27. In addition, time-dependent DFT calculations (B3LYP-GD3//SDD(f)/6-31G**) were performed to their rationalization, considering tetrahydrofuran as solvent. Selected absorptions are collected in Table 1, whereas frontier orbitals are shown in Figures 5 and S28–S36.

**Figure 4:**
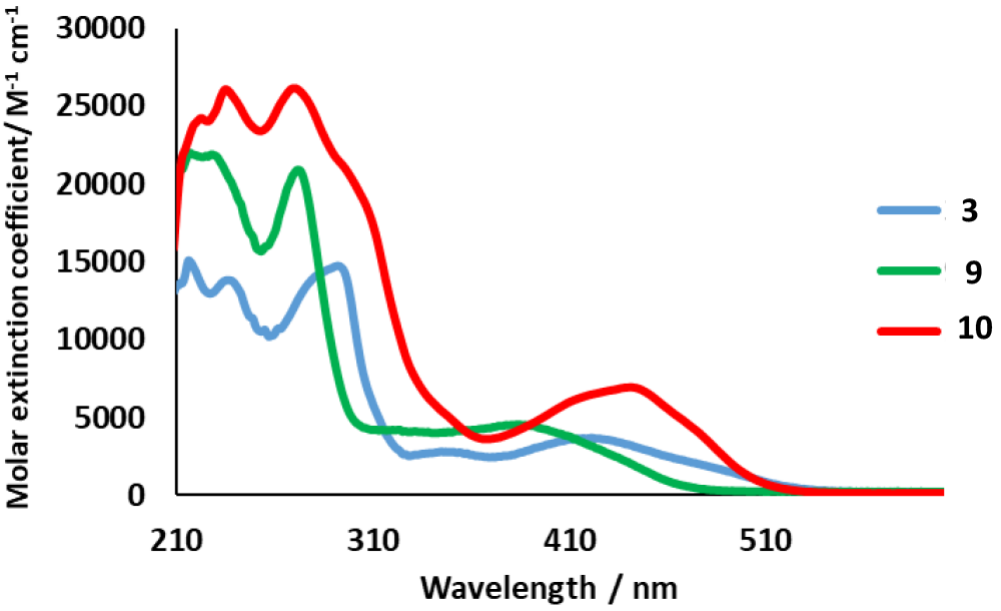
UV–vis spectra of complexes **3**, **9**, and **10** recorded in 2-methyltetrahydrofuran (1 × 10^–4^ M) at 298 K.

**Table 1:** Selected Experimental UV-vis Absorptions for **3**–**11** (in MeTHF) and Computed TD-DFT (in THF) Vertical Excitation Energies and Their Major Contributions

λ exp (nm)	ε (M^–1^ cm^–1^)	excitation energy (nm)	oscilator strength *f*	transition
Complex **3**				
241	13310	232	0.1039	HOMO–6 → LUMO (47%)
427	3360	400	0.0582	HOMO–1 → LUMO (91%)
477	1730	444 (*S*_1_)	0.0272	HOMO → LUMO (91%)
512	290	498 (*T*_1_)	0	HOMO → LUMO (95%)
Complex **4**				
243	18290	235	0.1747	HOMO–6 → LUMO (56%)
426	4590	399	0.0599	HOMO–1 → LUMO (91%)
484	2350	446 (*S*_1_)	0.0327	HOMO → LUMO (91%)
506	1230	507 (*T*_1_)	0	HOMO → LUMO (95%)
Complex **5**				
243	18360	255	0.1933	HOMO–7 → LUMO (21%)
				HOMO–5 → LUMO (54%)
453	2430	405	0.0457	HOMO–1 → LUMO (88%)
494	1360	444 (*S*_1_)	0.0344	HOMO → LUMO (88%)
513	740	504 (*T*_1_)	0	HOMO → LUMO (95%)
Complex **6**				
282	28300	282	0.2481	HOMO–2 → LUMO+3 (51%)
408	5420	394	0.0653	HOMO–2 → LUMO (96%)
470	7470	440 (*S*_1_)	0.0966	HOMO → LUMO (96%)
506	3380	511 (*T*_1_)	0	HOMO → LUMO (96%)
Complex **7**				
274	33490	270	0.1805	HOMO–2 → LUMO+4 (65%)
416	2620	396	0.0531	HOMO–2 → LUMO (96%)
478	3810	447 (*S*_1_)	0.0949	HOMO → LUMO (97%)
524	320	525 (*T*_1_)	0	HOMO → LUMO (96%)
Complex **8**				
282	35260	278	0.0482	HOMO → LUMO+6 (59%)
402	9500	383	0.1737	HOMO–2 → LUMO (50%)
				HOMO → LUMO+1 (45%)
496	2380	481 (*S*_1_)	0.0347	HOMO → LUMO (95%)
532	2310	551 (*T*_1_)	0	HOMO → LUMO (96%)
Complex **9**				
276	20330	274	0.2307	HOMO–4 → LUMO (78%)
403	3560	381 (*S*_1_)	0.0657	HOMO → LUMO (94%)
463	500	445 (*T*_1_)	0	HOMO → LUMO (82%)
Complex **10**				
224	23460	247	0.0776	HOMO–4 → LUMO+3 (89%)
404	5330	390	0.0496	HOMO–1 → LUMO (95%)
450	6250	426 (*S*_1_)	0.0798	HOMO → LUMO (95%)
504	770	492 (*T*_1_)	0	HOMO → LUMO (94%)
Complex **11**				
273	24510	266	0.3100	HOMO–8 → LUMO (40%)
				HOMO → LUMO+3 (38%)
370	1900	338	0.0484	HOMO–4 → LUMO (97%)
431	3410	390 (*S*_1_)	0.1364	HOMO → LUMO (98%)
461	900	461 (*T*_1_)	0	HOMO → LUMO (88%)

**Figure 5:**
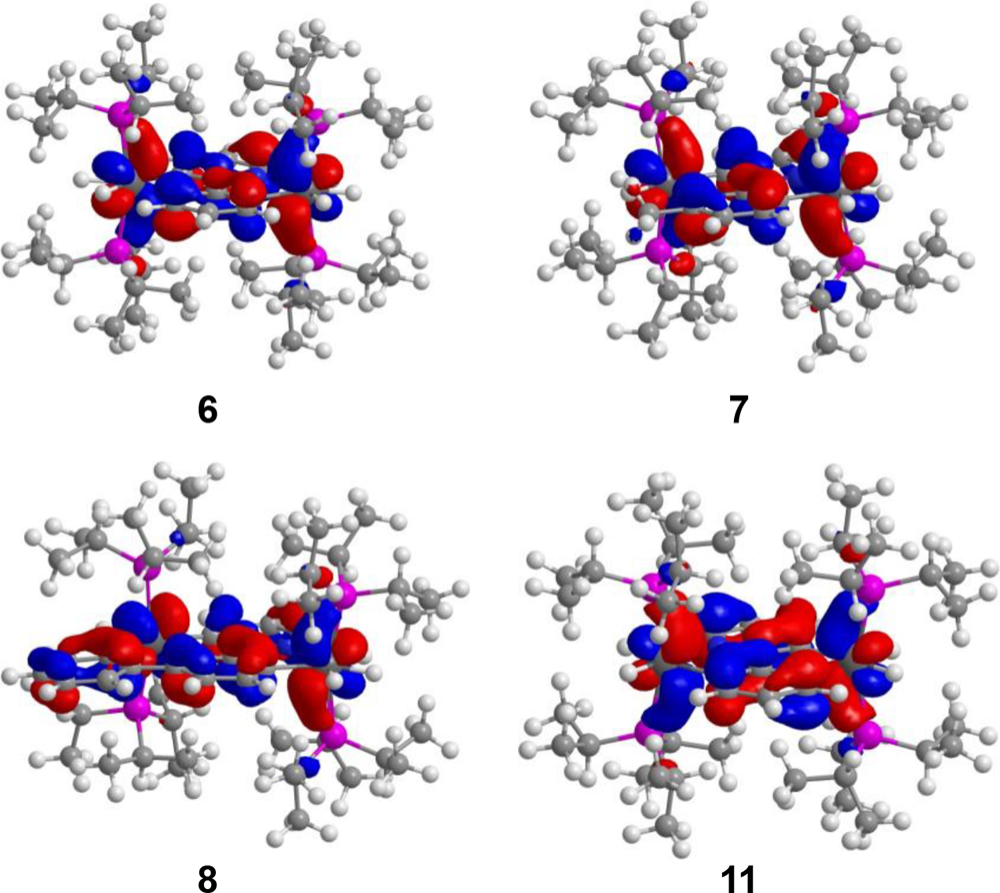
HOMO of the homobinuclear derivatives **6**–**8** and **11**.

The spectra of the osmium mononuclear precursors **3**-**5** show bands in three different regions of the spectrum: <300, 300–500, and >500 nm. The absorptions at the highest energy region correspond mainly to ^1^π–π* intraheterocycle transitions, whereas the bands between 300 and 500 nm are due to transitions from the metal to the heterocycle mixed with from the heterocycle to the heterocycle. These bands mainly result from HOMO–1-to-LUMO, and HOMO-to-LUMO transitions. Both HOMO–1 and HOMO are essentially located at the metal center and the metalated heterocycle. For HOMO, the percentage of the former is between 52% and 59% and that of the second one lies in the range 28–38%. The LUMO is almost exclusively centered on the metalated heterocycle (95%). The very weak absorption tails after 500 nm are assigned to formally spin forbidden ^3^MLCT transitions caused by the large spin–orbit coupling introduced by osmium. The spectrum of the mononuclear iridium complex **9** is similar to those of **3**-**5**. The absorptions of <300 nm should be assigned to ^1^π–π* ligand-to-ligand transitions, whereas those between 300 and 450 nm are due to spin-allowed iridium-to-heterocycle charge transfer (^1^MLCT) mixed with heterocycle-to-heterocycle transitions. The absorption tails after 450 nm correspond to formally spin forbidden ^3^MLCT transitions, which are produced by the large spin–orbit coupling introduced, in this case, by the iridium center.

Complexes **3**–**5** and **9** display a HOMO involving substantial mixing with a π-ligand backbone (Figures S28–S30 and S34). Thus, they fulfill the main requirement in order to serve as metal–ligand species, which allow building binuclear compounds bearing metals electronically coupled, where the new HOMO is delocalized between the metal centers connected by the π-linker. As a proof-of-concept validation, the HOMO of the homobinuclear derivatives **6**–**8** and **11** is clearly delocalized throughout the metal–heterocycle-metal system (Figure 5) with similar participation percentage of the three moieties. As in the mononuclear precursors, the LUMO is almost exclusively centered on the heterocycle. The UV–vis spectra of the binuclear osmium compounds **6**–**8** show bands between 274 and 496 nm corresponding to osmium-to-heterocycle charge transfer (^1^MLCT) mixed with heterocycle-to-heterocycle transitions and weak absorption tails after 500 nm due to formally spin forbidden ^3^MLCT transitions, whereas the spectrum of the binuclear iridium derivative **11** contains bands between 249 and 431 nm assigned to iridium-to-heterocycle charge transfer (^1^MLCT) mixed with heterocycle-to-heterocycle transitions and weak absorption tails after 440 nm due to formally spin forbidden ^3^MLCT transitions.

The HOMO delocalization throughout metal–heterocycle-metal of the binuclear complexes requires not only the HOMO delocalization along metal-heterocycle of the metal–ligand mononuclear precursor but also electronic compatibility between the metal fragments linked by the heterocycle. This is given in complexes **6**–**8**, where the heterocycle links two *d*^4^-metal fragments, and in complex **11** formed by two *d*^6^-metal moieties. In contrast to complexes **6**–**8** and **11**, the heterocycle of the heterobinuclear derivative **10** associates fragments of two different ions, *d*^4^ and *d*^6^, which appear to be inconsistent to produce electronic coupling. Thus, the HOMO of this compound (Figure S35) is essentially centered on the osmium atom (45%) and the heterocycle (33%), whereas the iridium center has only a residual contribution (5%). Despite this difference, the UV–vis spectrum of **10** can be rationalized in a similar manner to those of **7** and **8**.

The mononuclear complexes **3**–**5** and binuclear derivatives **6**–**8** are osmium(IV) phosphorescent emitters in the orange-red spectral region (546–728 nm) upon photoexcitation. Emission spectra in doped poly(methyl methacrylate) (PMMA) film at 5 wt % at room temperature and MeTHF at room temperature and at 77 K are shown in Figures S37–S54, whereas Table 2 shows the experimental and calculated wavelengths, observed lifetimes, quantum yields, and radiative and nonradiative rate constants. The spectra of the six compounds are very similar, which is consistent with the scarce differences found for the DFT-calculated HOMO–LUMO gaps (3.26–3.54 eV, see Table 2). Because the emissions can be attributed to *T*_1_ excited states, there is good agreement between the experimental wavelengths and those calculated through the estimation of the difference in energy between the optimized triplet states *T*_1_ and the singlet states *S*_0_ in tetrahydrofuran. The observed lifetimes are in the range of 1.5–5.2 μs. Quantum yields are modest and higher for the binuclear compounds. This poor efficiency could be related to the low value of the radiative rate constants. Phosphorescent emitters based on osmium^20^ are comparatively much less frequent than those of iridium^21^ and platinum^22^ in particular the osmium(IV) ones.^19,23^ Complexes **6**–**8** are the first reported binuclear osmium(IV) emitters. In contrast to **3**–**8**, the iridium derivatives **9**–**11** are not emissive.

**Table 2:** Emission Properties of Complexes **3**–**8**^a^

complex	HOMO (eV)	LUMO (eV)	HLG (eV)	calc λ_em_^*a*^ (nm)	media (*T*, K)	λ_em_ (nm)	τ_obs_ (μs)	Φ	*k*_r_^b^ (s^–1^)	*k*_nr_^b^ (s^–1^)	*k*_r_*/k*_nr_
**3**					PMMA (298)	599	1.5	0.01	6.6 × 10^3^	6.6 × 10^5^	0.01
	–4.81	–1.27	3.54	591	MeTHF (298)	614	2.3	0.01	4.3 × 10^3^	4.3 × 10^5^	0.01
					MeTHF (77)	578	4.1				
**4**					PMMA (298)	593	3.1	0.02	6.4 × 10^3^	3.1 × 10^5^	0.02
	–4.75	–1.24	3.51	602	MeTHF (298)	610	3.1	0.02	6.4 × 10^3^	3.1 × 10^5^	0.02
					MeTHF (77)	550	5.2				
**5**					PMMA (298)	593	3.3	0.01	3.0 × 10^3^	3.0 × 10^5^	0.01
	–4.78	–1.29	3.49	564	MeTHF (298)	611	2.7	0.01	3.7 × 10^3^	3.6 × 10^5^	0.01
					MeTHF (77)	574	3.4				
**6**					PMMA (298)	562, 599	4.4	0.03	6.8 × 10^3^	2.2 × 10^5^	0.03
	–4.58	–1.11	3.47	577	MeTHF (298)	562	3.9	0.02	5.1 × 10^3^	2.5 × 10^5^	0.02
					MeTHF (77)	546, 592	3.9				
**7**					PMMA (298)	570, 611	3.7	0.06	1.6 × 10^4^	2.5 × 10^5^	0.06
	–4.50	–1.08	3.42	596	MeTHF (298)	627	4.5	0.03	6.6 × 10^3^	2.1 × 10^5^	0.03
					MeTHF (77)	559, 620	3.5				
**8**					PMMA (298)	598, 658, 728	2.7	0.03	1.1 × 10^4^	3.6 × 10^5^	0.03
	–4.35	–1.09	3.26	620	MeTHF (298)	602, 651	1.5	0.08	5.3 × 10^4^	6.1 × 10^5^	0.08
					MeTHF (77)	584, 641, 708	3.3				

a Predicted from TD-DFT calculations in THF at 298 K by estimating the energy difference between the optimized *T*_1_ and singlet *S*_0_ state.

b Calculated according to the equations *k*_r_ = Φ/τ_obs_ and *k*_nr_ = (1 – Φ)/τ_obs_, where *k*_r_ is the radiative rate constant, *k*_nr_ is the nonradiative rate constant, Φ is the quantum yield, and τ_obs_ is the excited-state lifetime.

### Electrochemical Properties

The redox properties of the osmium precursors **3**–**5**, the mononuclear iridium complex **9**, and the binuclear derivatives **6**–**8**, **10**, and **11** were evaluated by cyclic voltammetry performed under argon atmosphere in a 0.1 M [NBu_4_]PF_6_ dichloromethane solution, and the potentials were referenced versus Fc/Fc^+^. Table 3 summarizes the main findings.

**Table 3:** Electrochemical Data of Complexes **3**–**11**

complex	*E*^ox1^ (V)	*E*_1/2_^ox1^ (V)	*E*^ox2^ (V)	*E*_1/2_^ox2^ (V)	*E*^ox3^ (V)	*E*_1/2_^ox3^ (V)	*K*_c(1–2)_	*K*_*c*(2–3)_
**3**	0.31		0.77	0.72				
**4**	0.25		0.72	0.67				
**5**	0.29	0.25	0.76	0.69				
**6**	0.09	0.06	0.42	0.39	0.73	0.69	8.30 × 10^5^	8.03 × 10^4^
**7**	0.03	0.00	0.39	0.35	0.82		1.55 × 10^6^	1.37 × 10^7^
**8**	–0.41	–0.46	0.00	–0.05	0.42	0.38	2.03 × 10^7^	6.84 × 10^6^
**9**	0.02	–0.03	0.43	0.37	0.84	0.80		
**10**	–0.32	–0.35	0.07		0.31		2.86 × 10^5^	3.24 × 10^3^
**11**	–0.18	–0.20	0.15	0.08	0.38		2.90 × 10^6^	9.86 × 10^3^

Mononuclear complexes **3**–**5** exhibit Os(IV)/Os(V) and Os(V)/Os(VI) oxidations peaks between 0.20 and 0.80 V. The second oxidation is quasi-reversible for the three compounds, whereas the first one is irreversible for **3** and **4** and quasi-reversible for **5** (Figures S55–S57). The mononuclear iridium compound **9** displays three quasi-reversible Ir(III)/Ir(IV), Ir(IV)/Ir(V), and Ir(V)/Ir(VI) oxidation peaks at 0.02, 0.43, and 0.84 V, respectively (Figure S61).

**Figure 6:**
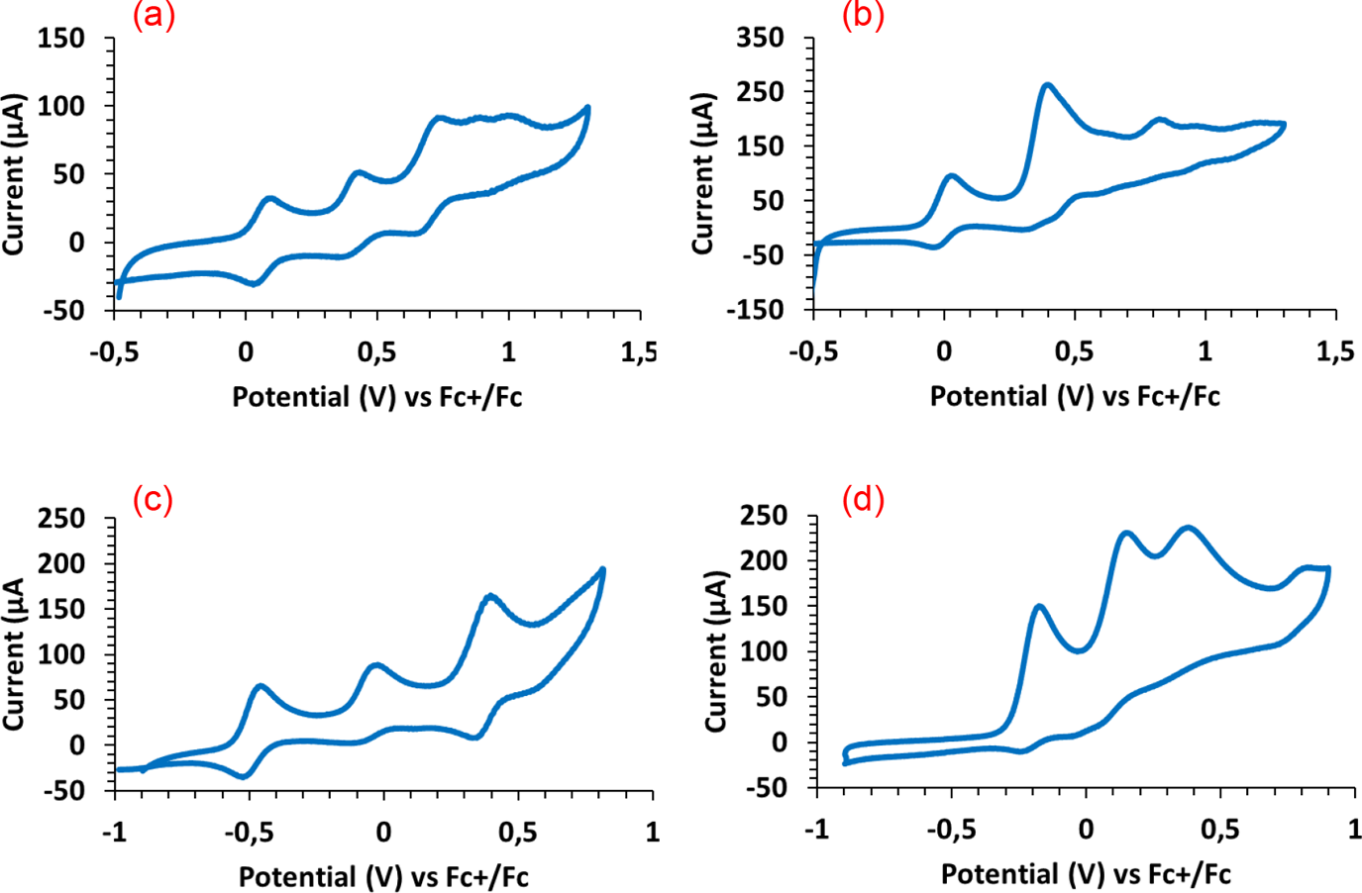
Cyclic voltammograms of 10^–3^ M dichloromethane solutions of complexes **6** (a), **7** (b), **8** (c), and **11** (d). Supporting electrolyte: [Bu_4_N]PF_6_ (0.1 M). Scan rate: 100 mV s^–1^. The potentials were referenced to the ferrocene/ferrocenium (Fc/Fc^+^) couple.

The cyclic voltammograms of the homobinuclear osmium derivatives **6**–**8** (Figure 6) contain three quasi-reversible [Os_2_]/[Os_2_]^+^, [Os_2_]^+^/[Os_2_]^2+^, and [Os_2_]^2+^/[Os_2_]^3+^ oxidation peaks between −0.45 and 0.90 V. The first of them is observed in the range −0.45–0.10 V, the second one between 0.00 and 0.42 V, and the last one in the range 0.42–0.90 V. Both separations between the consecutive waves (Δ*E*) are long, yielding large values of *K*_c_ (*K*_c_ = e^–n*F*Δ*E*/*RT*^),^24^ between 8.03 × 10^4^ and 2.03 × 10^7^. They in a first glance suggest class III radicals with the odd electron fully delocalized (eq 1; *n* = 4, 5).^3d^ The homobinuclear iridium complex **11** (Figure 6d) also exhibits three oxidation peaks at −0.18, 0.15, and 0.38 V. However, the separations between them are in this case significantly different. The separation between the first oxidation and the second one is long, giving rise to a *K*_c_ value of 2.90 × 10^6^, which lies within the range found for **6**–**8**. On the other hand, the separation between the second oxidation peak and the third one is shorter. It only allows calculating a *K*_c_ value of 9.86 × 10^3^. The heterobinuclear complex **10** (Figure S62) has three quasi-reversible oxidation peaks at −0.32, 0.07, and 0.31 V, corresponding to independent events on each metal. According to the contribution of the metal centers to the HOMO of the species generated in the process and their respective spin density maps (Figure S121), the first oxidation appears to take place on the osmium center, whereas the second and third ones should occur on the iridium center.
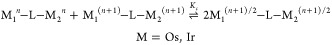
1

### UV–vis–NIR Spectra of the Oxidized Binuclear Species

UV–vis–NIR spectroelectrochemical investigations on 1 × 10^–3^ M solutions of the homobinuclear complexes **7**, **8**, and **11** and the heterobinuclear derivative **10** in dichloromethane and in the presence of 0.1 M of [NBu_4_]PF_6_ were carried out in order to corroborate the formation of mixed valence species, suggested by the electrochemical study, as a result of the performed oxidations. In contrast to **7**, **8**, **10**, and **11**, the solubility of the symmetrical complex **6** in the usual organic solvents is not enough to carry out the same spectroelectrochemical study with this compound.

**Figure 7:**
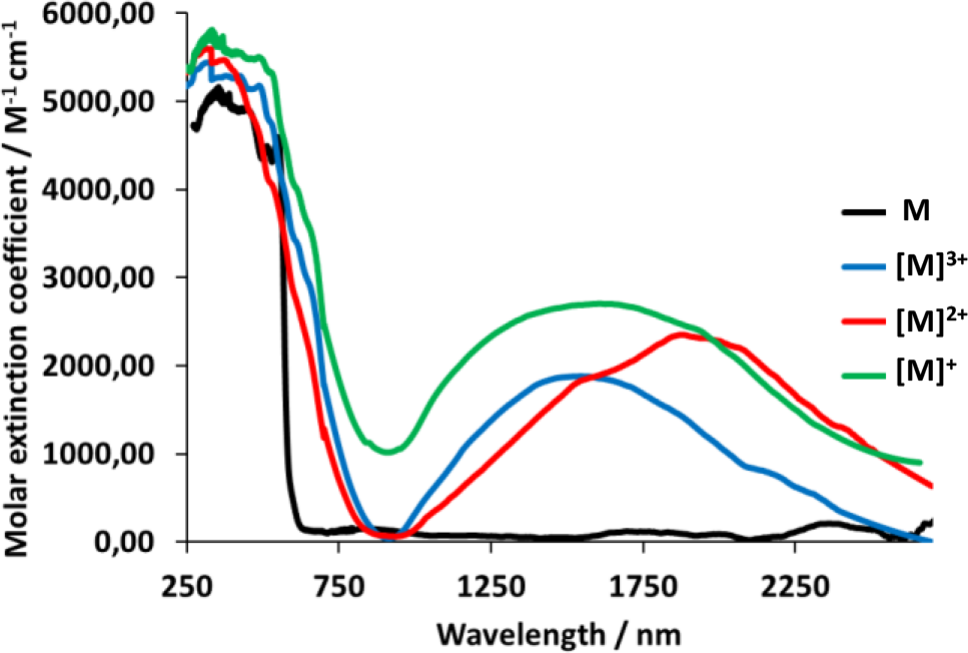
UV–vis–NIR absorption spectra of complexes **8**-[**8]**^**3+**^ in dichloromethane solutions.

The comparison of the spectra of the monocations [M_2_]^+^ with those of the neutral complexes reveals interesting findings. The spectrum of the monocation [**7**]^+^ (Figure S85) shows growing of the absorption bands in the visible region between 450 and 550 nm, with regard to that of **7** (Figure S84), together with the appearance of a broader absorption centered at 1746 nm in the NIR region. This behavior is ascribed to a HOMO(B)-to-LUMO(B) intervalence charge transfer transition (IVCT) signature by a mixed-valence species. An IVCT band is also observed in the spectrum of [**8**]^+^ (Figure 7, green line). It appears at 1705 nm, slightly red-shifted with regard to [**7**]^+^ by about 40 nm, being much more intense. In contrast, the spectrum of the diiridium cation [**11**]^+^ (Figure S93) has a much less intense IVCT band at 912 nm, blue-shifted. The spectrum of the heterobimetallic Os–Ir cation [**10**]^+^ (Figure S98) does not contain any perceptible IVCT band, in spite of that DFT calculations predict a weak transition at 1066 nm.

The oxidation of the monocations to the [M_2_]^2+^ species gives rise to the disappearance of the IVCT band in some cases. Spectra of the dications [**7**]^2+^ and [**11**]^2+^ do not contain any IVCT band (Figures S86 and S94). However, an intense IVCT transition at 2000 nm is observed in the spectrum of [**8**]^2+^ (Figure 7, red line); which is about 300 nm red-shifted with regard to that of [**8**]^+^. DFT calculations on [**8**]^2+^ reveal that the triplet state is 3.9 kcal/mol more stable than the singlet state, so that [**8**]^2+^ should be described as a diradical. The oxidation from the dications [M_2_]^2+^ to the trications [M_2_]^3+^ regenerates mixed-valence species for [**7**]^3+^ and [**8**]^3+^. Thus, the spectra of [**7**]^3+^ (Figure S87) and [**8**]^3+^ (Figure 7, blue line) contain an IVCT transition centered at about 1824 and 1614 nm, red-shifted by 77 and 15 nm with regard to those of the respective monocations.

Mixed-valence transition metal complexes can be classified using the delocalization parameter Γ,^12b^ which is calculated by means of eq 2(3d)
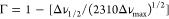
2where Δν_1/2_ and Δν_max_ are the bandwidth at the half height and the maximum absorption, respectively, for a Gaussian-shaped ICTV band (cm^–1^).

Table 4 collects the values of the delocalization parameter, calculated according to eq 2, for the ITCV bands previously mentioned. Values of Γ < 0.5 indicate mixed-valence complexes of class II, while values of Γ > 0.5 are characteristic of compounds of class III. Complexes in the borderline class II/class III display values of Γ ≈ 0.5. According to this criteria, cation [**7**]^3+^ belongs to class II, whereas ITCV bands of the cations resulting from the three sequential oxidations of the asymmetrical homobinuclear osmium complex **8** and the diiridium cation [**11**]^+^ give Γ values, which fit to class III. Cation [**7**]^+^ appears to be a species of the borderline class II/class III with a Γ-value of 0.51.

**Table 4:** Mixed-Valence and IVCT Parameters

complex	ν_max_/cm^–1^^a^	Δν_1/2_/cm^–1^^a^	Δν_1/2_^0^/cm^–1^^a^	Γ^b^
**[7]**^**+**^	9676	2287	4728	0.51
**[7]**^**3+**^	11088	3467	5061	0.31
**[8]**^**+**^	6307	1427	3817	0.63
**[8]**^**2+**^	5338	742	3511	0.78
**[8]**^**3+**^	6481	1045	3869	0.72
**[11]**^**+**^	11131	2001	5071	0.61

a From Gaussian fit of ε/ν versus ν.

b Parameters calculated using eq 2.

### Nature of the MH_*n*_ Units upon Oxidation

**Chart 1:**
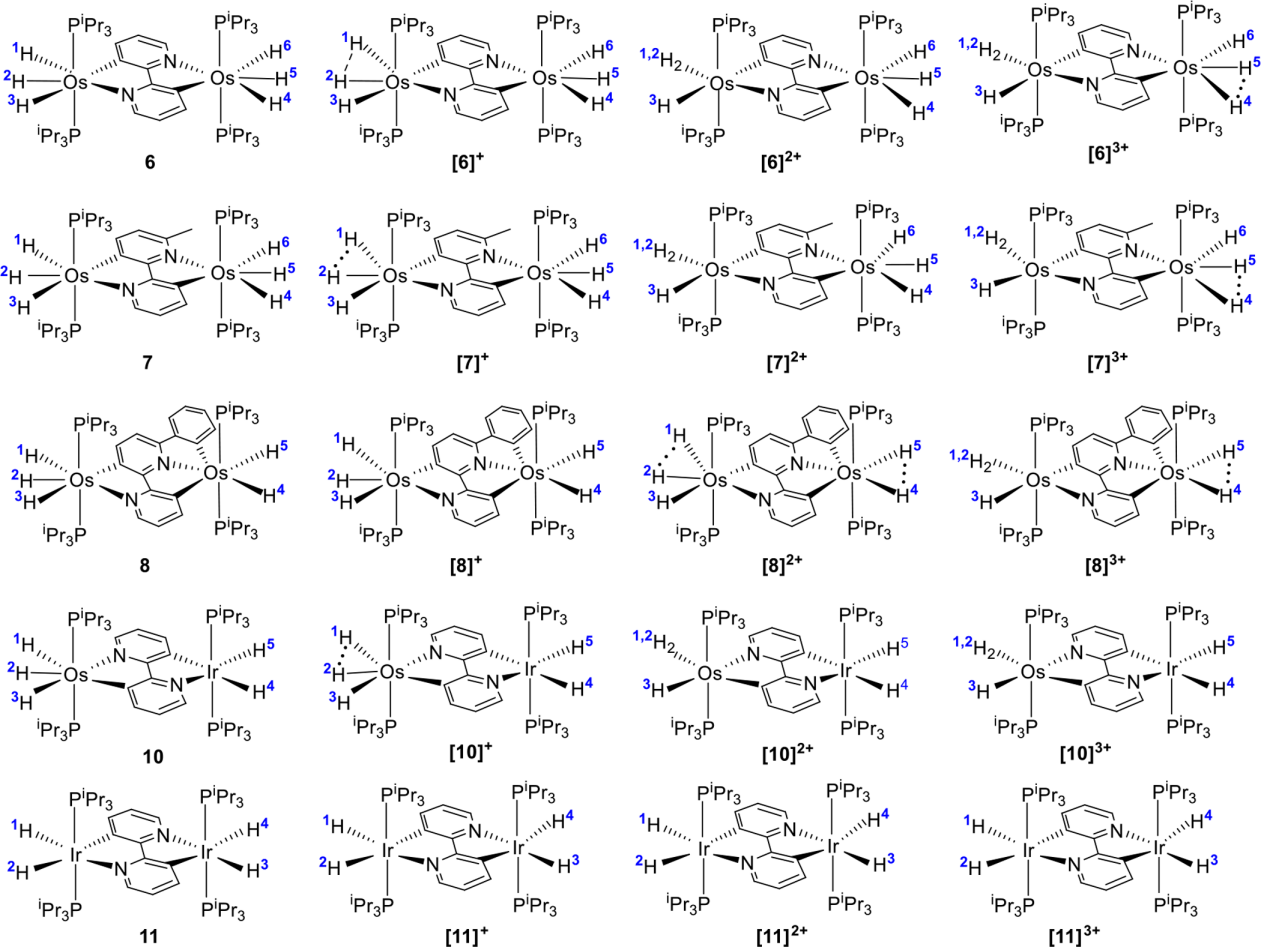
Nature of the MH_*n*_ Units of the Neutral and Cationic Forms

The dissociation energy of a hydrogen molecule from a polyhydride complex depends upon the electron density of the metal. This energy increases as the hydrogen–hydrogen interaction decreases and therefore it is higher for hydride forms than for dihydrogen ones. This is a direct consequence of the metal-dihydrogen bonding situation. Similar for all σ-complexes, the interaction between the coordinated hydrogen molecule and the transition metal in the dihydrogen compounds involves σ-donation from the σ-orbital of the coordinated bond to empty orbitals of the metal and back bonding from the metal to the σ*-orbital of the bond. The balance between donation and back-donation determines the oxidative addition degree, which has been fit to the separation between the coordinated hydrogen atoms.^1^ To gain insight into the influence of the sequential oxidation of the binuclear complexes **6**–**8**, **10**, and **11** on the respective MH_*n*_ units, we comparatively analyzed the hydrogen–hydrogen separations in the optimized structures of the generated cations (Figures S64–S83). Chart 1 gives a view of these structures, whereas Table 5 gathers the separation between the hydrogen atoms of the MH_*n*_ units.

**Table 5:** Calculated (B3LYP-D3//SDD(f)/6-31G**) Separation between the Hydrogen Atoms Bonded to the Metal

complex	H_1_—H_2_ (Å)	H_2_—H_3_ (Å)	H_4_—H_5_ (Å)	H_5_—H_6_ (Å)	H_3_—H_4_ (Å)
**6**	1.6	1.8	1.6	1.8	
**[6]**^**+**^	1.5	1.8	1.6	1.8	
**[6]**^**2+**^	0.9	2.3	1.6	1.8	
**[6]**^**3+**^	0.9	2.3	1.4	1.9	
**7**	1.6	1.8	1.6	1.7	
**[7]**^**+**^	1.5	1.8	1.6	1.7	
**[7]**^**2+**^	0.9	2.3	1.6	1.7	
**[7]**^**3+**^	0.9	2.2	1.5	1.8	
**8**	1.6	1.8	1.6		
**[8]**^**+**^	1.6	1.8	1.6		
**[8]**^**2+**^	1.5	1.9	1.5		
**[8]**^**3+**^	0.9	2.2	1.4		
**10**	1.6	1.8	2.3		
**[10]**^**+**^	1.5	1.8	2.3		
**[10]**^**2+**^	0.9	2.3	2.3		
**[10]**^**3+**^	0.9	2.2	2.4		
**11**	2.3				2.3
**[11]**^**+**^	2.3				2.4
**[11]**^**2+**^	2.3				2.5
**[11]**^**3+**^	2.3				2.5

**Figure 8:**
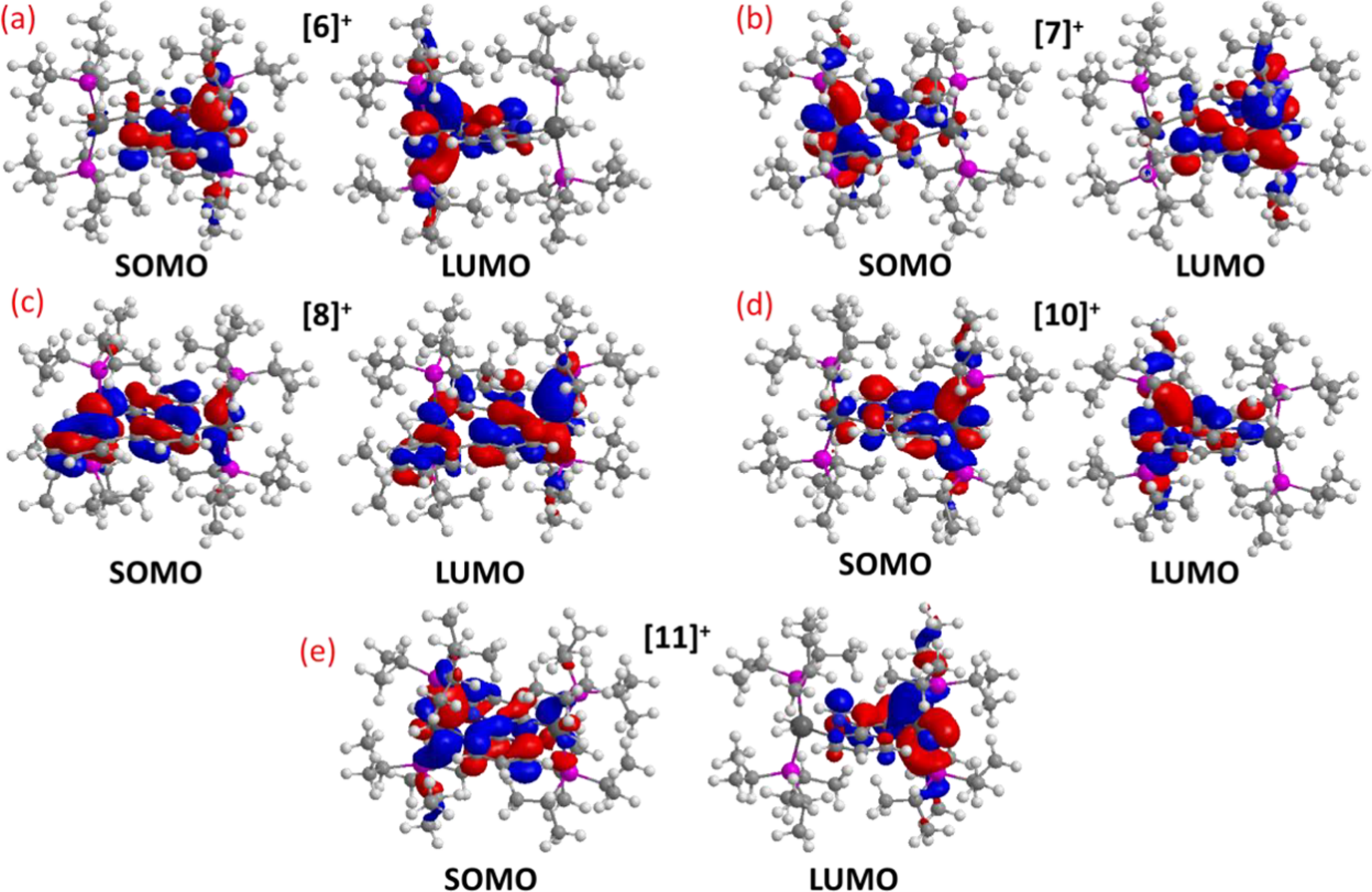
SOMO and LUMO of complexes [**6**]**^+^** (a), [**7**]**^+^** (b), [**8**]**^+^** (c), [**10**]^+^ (d), and [**11**]**^+^** (e).

The neutral complexes are in the four cases classical hydrides with separations between their hydride ligands longer than 1.6 Å. The monocations [M_2_]^+^ are also pure hydrides, although it should be mentioned that subtle but significant differences are observed between them. Two of the hydride ligands of a half of [**6**]^+^ approach about 0.1 Å to form a compressed dihydride (H(1) and H(2)). The same behavior is observed in the OsH_3_(P^i^Pr_3_)_2_ moiety of [**7**]^+^ linked to the nitrogen atom of the unsubstituted pyridyl ring and in the OsH_3_(P^i^Pr_3_)_2_ moiety of [**10**]^+^. In contrast, the hydrides of [**8**]^+^ and [**11**]^+^ are not affected. This difference in behavior appears to be connected with the distribution of the frontier orbitals of the cations (Figure 8). The SOMO of [**6**]^+^ (a), [**7**]^+^ (b), and [**10**]^+^ (d) is mainly centered on the heterocycle linker and the metal center keeping invariant the MH_*n*_ unit, while the LUMO is distributed between the heterocycle linker and the metal center of the modified MH_*n*_ unit. In contrast, both SOMO and LUMO of [**8**]^+^ (c) are delocalized on the heterocycle linker and the metals. The SOMO of [**11**]^+^ (e) is similarly distributed. However, the LUMO is mainly centered on the heterocycle linker and one of the metals.

The oxidation from the monocations to the [M_2_]^2+^ species enhances the approaching of the compressed dihydrides, which become a Kubas-type dihydrogen in [**6**]^2+^, [**7**]^2+^, and [**10**]^2+^. On the other hand, two hydrides of each OsH_*n*_ unit of the cation [**8**]^2+^ approach 0.1 Å to generate a compressed dihydride attached to each metal. In contrast to [**6**]^2+^, [**8**]^2+^ and [**10**]^2+^, the hydrides of [**11**]^2+^ remain unaltered. The oxidation of the dications has also different implications depending upon the generated trication. Cations [**6**]^3+^ and [**7**]^3+^ undergo the transformation of two hydride ligands of the previously unaffected OsH_3_(P^i^Pr_3_)_2_ moiety from classical to compressed, while the hydrogen atoms of the other are not affected. The OsH_3_(P^i^Pr_3_)_2_ moiety of [**8**]^2+^ is more sensitive to the oxidation than the OsH_2_(P^i^Pr_3_)_2_ one. Thus, while the compressed dihydrides of the OsH_3_(P^i^Pr_3_)_2_ moiety of [**8**]^2+^ are transformed into a Kubas-type dihydrogen in [**8**]^3+^, those of the OsH_2_(P^i^Pr_3_)_2_ moiety only experience a slight approach. The MH_*n*_ units of cations [M_2_]^2+^ and [M_2_]^3+^ of **10** and **11** display similar parameters.

The previous observations suggest that the MH_*n*_ units of *d*^4^-osmium fragments are more sensitive to the oxidation than those of *d*^6^-iridium fragments and that the metal center of the MH_*n*_ unit that undergoes the transformation is that with the highest contribution to the LUMO of the binuclear species.

## Concluding Remarks

The rollover cyclometalated hydride derivatives OsH_3_{κ^2^-*C*,*N*-[C_5_RH_2_N-py]}(P^i^Pr_3_)_2_ (R = H, Me, Ph) and IrH_2_{κ^2^-*C*,*N*-[C_5_RH_2_N-py]}(P^i^Pr_3_)_2_ display frontier orbitals involving substantial mixing of the metal center and the π-heterocycle backbone. The activation of an *ortho*-CH bond of the heterocyclic moiety of these metal–ligand units, promoted by the platinum group metals polyhydride complexes OsH_6_(P^i^Pr_3_)_2_ and IrH_5_(P^i^Pr_3_)_2_, gives rise to four different classes of binuclear derivatives: the hexahydride (P^i^Pr_3_)_2_H_3_Os{μ-[κ^2^-*C*,*N*-[C_5_RH_2_N-C_5_H_3_N]-*N*,*C*-κ^2^]}OsH_3_(P^i^Pr_3_)_2_ compounds with two OsH_3_(P^i^Pr_3_)_2_ halves, the homopentahydride (P^i^Pr_3_)_2_H_3_Os{μ-[κ^2^-*C*,*N*-[C_5_H_3_N-C_5_(C_6_H_4_)H_2_N]-*C*,*N*,*C*-κ^3^]}OsH_2_(P^i^Pr_3_)_2_ derivative bearing OsH_3_(P^i^Pr_3_)_2_ and OsH_2_(P^i^Pr_3_)_2_ fragments, the heteropentahydride (P^i^Pr_3_)_2_H_3_Os{μ-[κ^2^-*C*,*N*-[C_5_H_3_N-C_5_H_3_N]-*N*,*C*-κ^2^]}IrH_2_(P^i^Pr_3_)_2_ with OsH_3_(P^i^Pr_3_)_2_ and IrH_2_(P^i^Pr_3_)_2_ units, and the tetrahydride (P^i^Pr_3_)_2_H_2_Ir{μ-[κ^2^-*C*,*N*-[C_5_H_3_N-C_5_H_3_N]-*N*,*C*-κ^2^]}IrH_2_(P^i^Pr_3_)_2_ complex formed by two IrH_2_(P^i^Pr_3_)_2_ moieties. With the exception of the heterobinuclear pentahydride (P^i^Pr_3_)_2_H_3_Os{μ-[κ^2^-*C*,*N*-[C_5_H_3_N-C_5_H_3_N]-*N*,*C*-κ^2^]}IrH_2_(P^i^Pr_3_)_2_, these compounds display HOMO delocalization throughout the metal–heterocycle-metal skeleton. This electronic situation lends them interesting electrochemical properties. Their sequential oxidation allows generating mixed valence species, including mono- and diradicals, which exhibit intervalence charge transfer transitions. This noticeable ability allows us to govern the strength of the hydrogen–hydrogen and metal–hydrogen interactions within the MH_*n*_ units of these compounds. This finding should be of paramount importance for the attractive goal of reversibly controlling the coordination of the hydrogen molecule in transition metal polyhydride complexes.

## Experimental Section

### General Information

All reactions were carried out with exclusion of air using Schlenk-tube techniques or in a drybox. Instrumental methods and X-ray details are given in the Supporting Information. In the NMR spectra (Figures S1–S18) the chemical shifts (in ppm) are referenced to residual solvent peaks (^1^H, ^13^C{^1^H}) or external 85% H_3_PO_4_ (^31^P{^1^H}). Coupling constants *J* and *N* (*N* = *J*_P–H_ + *J*_P’-H_ for ^1^H and *N* = *J*_P–C_ + *J*_P’-C_ for ^13^C{^1^H}) are given in hertz.

### Preparation of (P^i^Pr_3_)_2_H_3_Os{μ-[κ^2^-*C*,*N*-[C_5_H_3_N-C_5_H_3_N]-*N*,*C*-κ^2^]}OsH_3_(P^i^Pr_3_)_2_ (**6**)

This compound can be prepared by two methods. Method a: A mixture of OsH_6_(P^i^Pr_3_)_2_ (**1**, 77 mg, 0.149 mmol) and OsH_3_{κ^2^-*C*,*N*-[C_5_H_3_N-py]}(P^i^Pr_3_)_2_ (**3**, 100 mg, 0.149 mmol) in toluene (6 mL) was refluxed for 16 h. The resulting suspension was cooled to room temperature, and the solvent was removed in vacuo. The addition of methanol (4 mL) caused the precipitation of a pale orange solid that was washed with further portions of methanol (3 × 3 mL) and finally it was dried in vacuo. Yield: 131 mg (74%). Method b: A mixture of **1** (150 mg, 0.29 mmol) and 2,2′-bipyridine (23 mg, 0.145 mmol) in toluene (6 mL) was refluxed for 16 h. The workup of the reaction is analogous as that described in Method a. Yield: 133 mg (78%). Anal. Calcd for C_46_H_96_N_2_Os_2_P_4_: C, 46.76; H, 8.19; N, 2.37. Found: C, 46.76; H 8.29; N, 2.31. IR (cm^–1^): ν(Os–H) 1985, 2102 (w). ^1^H NMR (300.13 MHz, toluene-*d*_8_, 298 K): δ 8.80 (d, ^3^*J*_H–H_ = 5.4, 2H, py), 8.30 (d, ^3^*J*_H–H_ = 7.3, 2H, py), 6.29 (m, 2H, py), 1.89 (m, 12H, PCH(CH_3_)_2_), 1.02 (dvt, ^3^*J*_H–H_ = 6.6, *N* = 13.0, 36H, PCH(CH_3_)_2_), 0.99 (dvt, ^3^*J*_H–H_ = 6.7, *N* = 13.4, 36H, PCH(CH_3_)_2_), −9.12 (br, 4H, Os–H), −12.30 (br, 2H, Os–H). ^1^H NMR (300.13 MHz, toluene-*d*_8_, high field region, 223 K): δ −5.92 (br, 2H, Os–H), −12.13 (br, 4H, Os–H). The low solubility of the solid precluded obtaining its ^13^C{^1^H} NMR spectrum. ^31^P{^1^H} NMR (121.50 MHz, toluene-*d*_8_, 298 K): δ 23.1 (s). *T*_1_(min) (ms, OsH. 300 MHz, toluene-*d*_8_, 223 K): 54 ± 5 (−5.92 ppm); 97 ± 10 (−12.13).

### Preparation of (P^i^Pr_3_)_2_H_3_Os{μ-[κ^2^-*C*,*N*-[C_5_H_2_MeN-C_5_H_3_N]-*N*,*C*-κ^2^]}OsH_3_(P^i^Pr_3_)_2_ (**7**)

This compound can be prepared by two methods. Method a: A mixture of **1** (76 mg, 0.146 mmol) and OsH_3_{κ^2^-*C*,*N*-[C_5_(Me)H_2_N-py]}(P^i^Pr_3_)_2_ (**4**, 100 mg, 0.146 mmol) in toluene (4 mL) was refluxed for 16 h, giving a dark orange suspension. After cooling the mixture to room temperature, the solvent was removed in vacuo, affording an orange residue. Addition of cold methanol (3 mL) caused the precipitation of an orange solid that was washed with cold methanol (3 × 3 mL) and dried in vacuo. Yield: 136 mg (78%). Method b: A mixture of **1** (100 mg, 0.194 mmol) and 6-methyl-2,2′-bipyridine (14.7 μL, 0.095 mmol) in toluene (4 mL) was refluxed for 16 h, giving a dark orange solution. The workup of the reaction is analogous at that described in Method a. Yield: 96 mg (83%). Anal. Calcd for C_47_H_98_N_2_Os_2_P: C, 47.21; H, 8.26; N, 2.34. Found: C, 47.31; H, 7.96; N, 2.34. HRMS (electrospray, *m*/*z*): calculated for C_47_H_98_N_2_Os_2_P_4_ [M]^+^, 1198.5905, found, 1198.5911. IR (cm^–1^): ν(Os–H) 1978 (w). ^1^H NMR (300.13 MHz, toluene-*d*_*8*_, 298 K): δ 8.85 (d, 1H, ^3^*J*_H–H_ = 5.0, py), 8.32 (d, ^3^*J*_H–H_ = 7.4, 1H, Me-py), 8.21 (d, ^3^*J*_H–H_ = 7.1, 1H, py), 6.62 (d, ^3^*J*_H–H_ = 7.4, 1H, Me-py), 6.25 (m, 1H, py), 2.91 (s, 3H, CH_3_), 1.93 (m, 12H, PC*H*(CH_3_)_2_), 1.04 (dvt, ^3^*J*_H–H_ = 6.5, *N* = 13.3, 36H, PCH(C*H*_3_)_2_), 1.09–0.92 (m, 72H, PCH(C*H*_3_)_2_), −9.21 (br, 4H, Os–H), −12.26 (br, 1H, Os–H), −13.17 (br, 1H, Os–H). ^13^C{^1^H}-apt NMR (75.48 MHz, toluene-*d*_8_, 298 K): δ 173.9 (t, ^2^*J*_C–P_ = 6.8, Os–C py), 173.7 (s, C Me-py), 173.6 (s, C py), 168.9 (t, ^2^*J*_C–P_*=* 6.2, Os–C Me-py), 153.8 (s, CH Me-py), 150.9 (s, CH py), 150.0 (s, CH py), 149.9 (s, C Me-py), 122.0 (s, CH py), 121.4 (s, CH Me-py), 33.3 (s, CH_3_), 28.3 (vt, *N* = 23.0, P*C*H(CH_3_)_2_), 28.2 (vt, *N* = 23.2, P*C*H(CH_3_)_2_), 20.6, 20.5, and 20.4 (all s, PCH(*C*H_3_)_2_). ^31^P{^1^H} NMR (121.50 MHz, toluene-*d*_*8*_, 298 K): δ 22.7 (s), 21.4 (s). *T*_1(min)_ (ms, OsH, 300 MHz, toluene-*d*_*8*_, 213 K): 57 ± 6 (−6.01 ppm); 57 ± 6 (−12.16 ppm); 48 ± 5 (−13.10 ppm).

### Preparation of (P^i^Pr_3_)_2_H_3_Os{μ-[κ^2^-*C*,*N*-[C_5_H_3_N-C_5_(C_6_H_4_)H_2_N]-*C*,*N*,C-κ^3^]}OsH_2_(P^i^Pr_3_)_2_ (**8**)

This compound can be prepared by two methods. Method a: A mixture of **1** (66 mg, 0.128 mmol) and OsH_3_{κ^2^-*C*,*N*-[C_5_(Ph)H_2_N-py]}(P^i^Pr_3_)_2_ (**5**, 96 mg, 0.128 mmol) in toluene (3 mL) was refluxed for 16 h, giving a dark orange suspension. After the mixture was cooled to room temperature, the solvent was removed in vacuo, affording an orange residue. Addition of cold methanol (3 mL) caused the precipitation of an orange solid that was washed with methanol (3 × 3 mL) and dried in vacuo. Yield: 122 mg (76%) Method b: A mixture of **1** (150 mg, 0.290 mmol) and 6-phenyl-2,2′-bipyridine (33.7 mg, 0.145 mmol) in toluene (5 mL) was refluxed for 16 h, giving a dark orange suspension. The workup of the reaction is analogous at that described in Method a. Yield: 147.5 mg (81%). Anal. Calcd for C_52_H_98_N_2_Os_2_P_4_: C, 49.74; H, 7.87; N, 2.23. Found: C, 49.48; H, 7.72; N, 2.14. HRMS (electrospray, *m*/*z*): calculated for C_52_H_97_N_2_Os_2_P_4_ [M – H]^+^, 1255.5826; found, 1255.5451. IR (cm^–1^): ν(Os–H) 2141, 2106 (w). ^1^H NMR (300.13 MHz, CD_2_Cl_2_, 298 K): δ 8.63 (d, 1H, ^3^*J*_H–H_ = 5.5, py), 8.14 (d, ^3^*J*_H–H_ = 7.8, 1H, central py), 7.83 (d, ^3^*J*_H–H_ = 7.4, 1H, py), 7.78 (d, ^3^*J*_H–H_ = 7.4, 1H, Ph), 7.51 (d, ^3^*J*_H–H_ = 7.6, 1H, Ph), 7.10 (d, ^3^*J*_H–H_ = 7.8, 1H, central py), 6.80 (t, ^3^*J*_H–H_*=* 7.4, 1H, Ph), 6.63 (t, ^3^*J*_H–H_ = 7.2, 1H, Ph), 6.21 (t, ^3^*J*_H–H_ = 5.6, 1H, py), 1.97 (m, 12H, PC*H*(CH_3_)_2_), 0.97 (dvt, ^3^*J*_H–H_ = 6.4, *N* = 13, 36H, PCH(C*H*_3_)_2_), 0.81 (dvt, ^3^*J*_H–H_ = 6.4, *N* = 12.2, 36H, PCH(C*H*_3_)_2_), −8,48 (dt, ^2^*J*_H–H_ = 11.3, ^2^*J*_H–P_ = 15.1, 1H, Os–H), −9.19 (dt, ^2^*J*_H–H_ = 11.3, ^2^*J*_H–P_ = 17.2, 1H, Os–H), −9.49 (br, 2H, Os–H), −12.47 (br, 1H, Os–H). ^1^H NMR (300.13 MHz, CD_2_Cl_2_, 203 K, high field region): δ – 6.23 (br, 1H, Os–H), −8.48 (dt, ^2^*J*_H–H_ = 16.8, ^2^*J*_H–P_ = 14.2_,_ 1H, Os–H), −9.22 (dt, ^2^*J*_H–H_ = 16.9, ^2^*J*_H–P_ = 10.8_,_ 1H, Os–H), −12.57 (br, 1H, Os–H), −12.89 (br, 1H, Os–H). ^13^C{^1^H}-apt NMR (75.48 MHz, CD_2_Cl_2_, 298 K): δ 175.2 (s, C py), 170.9 (s, C central py), 169.9 (t, ^2^*J*_C–P_ = 8.3, Os–C Ph), 168.0 (t, ^2^*J*_C–P_*=* 6.1, Os–C central py), 165.5 (t, ^2^*J*_C–P_ = 8.5, Os–C py), 156.7 (s, C central py), 151.9 (s, CH central py), 150.3 (s, C Ph), 149.9 (s, CH Ph), 149.5 (s, CH py), 146.9 (s, CH Ph), 126.9 (s, CH Ph), 122.4 (s, CH py), 122.2 (s, CH Ph), 119.8 (s, CH Ph), 113.8 (s, CH central py), 28.4 (vt, *N* = 23.4, P*C*H(CH_3_)_2_), 27.0 (vt, *N* = 23.6, P*C*H(CH_3_)_2_), 20.6, 20.3, 19.9, and 19.4 (all s, PCH(*C*H_3_)_2_). ^31^P{^1^H} NMR (121.50 MHz, CD_2_Cl_2_, 298 K): δ 24.1 (s), 1.8 (s).

### Preparation of (P^i^Pr_3_)_2_H_3_Os{μ-[κ^2^-*C*,*N*-[C_5_H_3_N-C_5_H_3_N]-*N*,*C*-κ^2^]}IrH_2_(P^i^Pr_3_)_2_ (**10**)

A mixture of **2** (100 mg, 0.193 mmol) and OsH_3_{κ^2^-*C,N*-(C_5_H_3_N-py)}(P^i^Pr_3_)_2_ (**3**, 129 mg, 0.193 mmol) in toluene (4 mL) was refluxed for 16 h. After this time, the resulting dark orange solution was cooled to room temperature, filtered through Celite and the solvent was removed in vacuo. The addition of pentane (5 mL) caused the precipitation of an orange solid; this was washed with further portions of pentane (3 × 2 mL) and finally dried in vacuo. Yield: 155 mg (68%). Anal. Calcd for C_46_H_95_IrN_2_OsP_4_: C, 46.72; H, 8.10; N, 2.37. Found: C, 46.72; H, 8.12; N, 2.36. HRMS (electrospray, *m*/*z*) calcd for C_46_H_94_IrN_2_OsP_4_ [M – H]^+^, 1183.5585; found, 1183.5529. IR (cm^–1^): ν(Ir–H) 2141 (m), ν(Os–H) 2102 (m), 1988 (m). ^1^H NMR (300.13 MHz, C_6_D_6_, 298 K): δ 8.93 (d, ^3^*J*_H–H_ = 5.5, 1H, CH py), 8.47 (m, 2H, CH py), 8.11 (d, ^3^*J*_H–H_ = 7.3, 1H, CH py), 6.38 (m, 2H, CH py), 2.04 (m, 6H, PC*H*(CH_3_)_2_), 1.93 (m, 6H, PC*H*(CH_3_)_2_), 1.05 (m, 72H, PCH(C*H*_3_)_2_), −8.98 (br, 2H, Os–H), −12.24 (br, 1H, Os–H), −13.05 (dt, ^2^*J*_H–H_ = 4.1, ^2^*J*_H–P_ = 21.4, 1H, Ir–H), −22.16 (dt, ^2^*J*_H–H_ = 4.1, ^2^*J*_H–P_ = 19.3, 1H, Ir -H). ^1^H NMR (300.13 MHz, toluene-*d*_8_, 223 K, high field region): δ – 5.80 (br, 1H, Os–H), −12.10 (br, 2H, Os–H), −12.93 (br t_,_^2^*J*_H–P_ = 18.9, 1H, Ir–H), −21.93 (dt_,_^2^*J*_H–H_ = 3.6, ^2^*J*_H–P_ = 18.9, 1H, Ir–H). ^13^C{^1^H}-apt NMR (75.48 MHz, C_6_D_6_, 298 K): δ 176.1, 174.0 (both s, C py), 173.4 (t, ^2^*J*_C–P_ = 6.4, Os–C), 163.2 (t, ^2^*J*_C–P_ = 6.2, Ir–C), 152.1, 150.1, 150.0, 147.7, 122.2, 122.1 (all s, CH py), 28.0 (vt, *N* = 23.3, P*C*H(CH_3_)_2_), 27.4 (vt, *N* = 26.8, P*C*H(CH_3_)_2_), 20.4, 20.3, 20.3, 20.1 (all s, PCH(*C*H_3_)_2_). ^31^P{^1^H} NMR (161.99 MHz, C_6_D_6_, 298 K): δ 30.3 (s, Ir–P), 22.4 (s, Os–P). *T*_1_(min) (ms, OsH, 300 MHz, toluene-*d*_8_, 243 K): 66 ± 7 (−12.15 ppm), value of the resonance at −5.98 ppm could not be calculated due to the broadness of it.

### Preparation of (P^i^Pr_3_)_2_H_2_Ir{μ-[κ^2^-*C*,*N*-[C_5_H_3_N-C_5_H_3_N]-*N*,*C*-κ^2^]}IrH_2_(P^i^Pr_3_)_2_ (**11**)

This compound can be prepared by two methods. Method a: A mixture of **2** (115 mg, 0.224 mmol) and IrH_2_{κ^2^-*C*,*N*-[C_5_H_3_N-py]}(P^i^Pr_3_)_2_ (**9**, 150 mg, 0.224 mmol) in toluene (8 mL) was refluxed for 16 h. After this time, the resulting yellow dark solution was cooled to room temperature, filtered through Celite, and the solvent was removed in vacuo. The addition of pentane (5 mL) caused the precipitation of a yellow solid, which was washed with further portions of pentane (3 × 5 mL), and finally, it was dried in vacuo. Yield: 225 mg (85%). Method b: A mixture of **2** (200 mg, 0.386 mmol) and 2,2′-bipyridine (30 mg, 0.193 mmol) in toluene (8 mL) was refluxed for 16 h. The workup of the reaction is analogous at that described in Method a. Yield: 204 mg (89%). Anal. Calcd for C_46_H_94_Ir_2_N_2_P_4_: C, 46.68; H, 8.01; N, 2.37. Found: C, 46.83; H, 8.18; N, 2.36. HRMS (electrospray, *m*/*z*) calcd for C_46_H_93_Ir_2_N_2_P_4_ [M – H]^+^, 1183.5542; found, 1183.5351. IR (cm^–1^): ν(Ir–H) 2151 (m), 1931 (m). ^1^H NMR (300 MHz, C_6_D_6_, 298 K): δ 8.57 (d, ^3^*J*_H–H_ = 5.1, 2H, CH py), 8.22 (d, ^3^*J*_H–H_ = 7.1, 2H, CH py), 6.46 (dd, ^3^*J*_H–H_ = 7.1, ^3^*J*_H–H_ = 5.1, 2H, CH py), 2.04 (m, 12H, PC*H*(CH_3_)_2_), 1.08 (dvt, ^3^*J*_H–H_ = 6.6, *N* = 13.5, 36H, PCH(C*H*_3_)_2_), 1.04 (dvt, ^3^*J*_H–H_ = 6.7, *N* = 13.1, 36H, PCH(C*H*_3_)_2_), −12.93 (dt, ^2^*J*_H–H_ = 4.1, ^2^*J*_H–P_ = 21.2, 1H, Ir–H), −22.00 (dt, ^2^*J*_H–H_ = 4.1, ^2^*J*_H–P_ = 19.3, 1H, Ir–H). ^13^C{^1^H}-apt NMR (75.45 MHz, C_6_D_6_, 298 K): δ 177.3 (s, C py), 163.6 (t, ^2^*J*_C–P_ = 6.5, Ir–C py), 150.8, 148.5, 121.9 (all s, CH py), 27.4 (vt, *N* = 26.8, P*C*H(CH_3_)_2_), 20.4, 20.1 (both s, PCH(*C*H_3_)_2_). ^31^P{^1^H} NMR (121.5 MHz, C_6_D_6_, 298 K): δ 29.9 (s).

### UV–vis–NIR Spectroelectrochemical Investigations

Spectroelectrochemical experiments combine UV–vis–NIR spectroscopic measurements and redox processes at the same time. Thus, they allow obtaining the spectra of specific controlled oxidation states. The electrochemical measurements were performed with a micro-Autolab FRA2 Type III (Methrom, Utrecht, Netherlands) potentiostat controlled by NOVA (v.2.1.4) software. For the optical measurements, a JASCO V670 spectrophotometer using quartz (1 mm optical path length) was used. The spectroelectrochemical cell (1 mL volume) was a DRP-PTGRID-TRANSCELL (DropSens). It contains an optically transparent Pt grid working electrode (0.6 × 0.4 cm) which allows the bulk electrolysis of the solution contained in the cell, a Ag/AgCl reference electrode, and a platinum counter electrode. The experiments were performed under argon and protected from the light in dichloromethane solution (10^–3^ M) with [Bu_4_N]PF_6_ as a supporting electrolyte (0.1 M). To obtain the UV–vis–NIR spectra, anodic potentials according to the previously measured cyclic voltammograms were applied for the corresponding oxidations to [M_2_]^+^, [M_2_]^2+^, and [M_2_]^3+^ during the wavelength scan.

## Supporting Information Available

Experimental details; crystallographic data; NMR spectra; experimental and computed UV/vis spectra; photophysical, UV–vis–NIR spectroelectrochemistry, electrochemical and theoretical studies (PDF)
Cartesian coordinates of the optimized structures (XYZ)

## Supplementary Material

ic0c03680_si_001.pdf

ic0c03680_si_002.xyz
