# Supplementary material for: Electronic Communication in Binuclear Osmium- and Iridium-Polyhydrides
Source: Inorg Chem. 2021 Feb 5;60(4):2783–96. doi: 10.1021/acs.inorgchem.0c03680 (PMC9179948; doi:10.1021/acs.inorgchem.0c03680)
Supplement: Supplementary file 1 — ic0c03680_si_001.pdf [file ic0c03680_si_001.pdf]

SUPPORTING INFORMATION

Electronic communication in Binuclear Osmium- and Iridium-Polyhydrides

Lara Cancela, Miguel A. Esteruelas,* Javier Galbán, Montserrat Oliván, Enrique Oñate, Andrea Vélez, and Juan C. Vidal

* e-mail: maester@unizar.es

Contents:

- Experimental Details	S2
- Structural Analysis of Complexes **6**, **8** and **11**	S3
- NMR Spectra	S5
- Computational Details	S23
- Energies of Optimized Structures	S24
- UV-vis Spectra of Complexes **3-11** (Observed and Calculated)	S30
- Analysis of Computed UV-vis Data for **3-11**	S34
- Theoretical Analysis of Molecular Orbitals of Complexes **3-11**	S42
- Normalized Excitation and Emission Spectra of Complexes **3-8**	S60
- Cyclic Voltammograms	S69
- Optimized Structures of Complexes **6**, [**6**]$^{+}$, [**6**]$^{2+}$, [**6**]$^{3+}$, **7**, [**7**]$^{+}$, [**7**]$^{2+}$, [**7**]$^{3+}$, **8**, [**8**]$^{+}$, [**8**]$^{2+}$, [**8**]$^{3+}$, **10**, [**10**]$^{+}$, [**10**]$^{2+}$, [**10**]$^{3+}$, **11**, [**11**]$^{+}$, [**11**]$^{2+}$, and [**11**]$^{3+}$	S74
- UV-vis-NIR Spectra of Complexes **7**, **8**, and **11**	S84
- UV-vis-NIR Spectra of [**7**]$^{+}$, [**8**]$^{+}$, [**10**]$^{+}$ and [**11**]$^{+}$ (Observed and Calculated)	S90
- Calculated HOMO, SOMO and LUMO of Complexes **6**, [**6**]$^{+}$, [**6**]$^{2+}$, [**6**]$^{3+}$, **7**, [**7**]$^{+}$, [**7**]$^{2+}$, [**7**]$^{3+}$, **8**, [**8**]$^{+}$, [**8**]$^{2+}$, [**8**]$^{3+}$, **10**, [**10**]$^{+}$, [**10**]$^{2+}$, [**10**]$^{3+}$, **11**, [**11**]$^{+}$, [**11**]$^{2+}$, and [**11**]$^{3+}$	S92
- Spin Density Distributions for the Optimized Triplet T_1 of **3-8**, and **10-11**	S99
- Spin Density Distributions for the Optimized Structures of [**6**]$^{+}$, [**7**]$^{+}$, [**8**]$^{+}$, [**10**]$^{+}$ and [**11**]$^{+}$.	S100
- References	S101

● **Experimental Details**

General Information. All reactions were carried out with exclusion of air using Schlenk-tube techniques or in a drybox. Pentane, dichloromethane and toluene were obtained oxygen- and water-free from an MBraun solvent purification apparatus, while methanol, 2-MeTHF and fluorobenzene were dried and distilled under argon prior to use. ^{1}H, ^{13}C{^{1}H}, and ^{31}P{^{1}H} NMR spectra were recorded on Bruker 300 ARX or Bruker Avance 300 MHz. Chemical shifts (expressed in ppm) are referenced to residual solvent peaks (^{1}H, ^{13}C{^{1}H}), or external 85% H_3PO_4 (^{31}P{^{1}H}). Coupling constants *J* and *N* ($N = J_{P\text{-}H} + J_{P'\text{-}H}$ for ^{1}H and $N = J_{P\text{-}C} + J_{P'\text{-}C}$ for ^{13}C{^{1}H}) are given in hertz. Attenuated total reflection infrared spectra (ATR-IR) of solid samples were run on a PerkinElmer Spectrum 100 FT-IR spectrometer. Elemental analyses were carried out in a PerkinElmer 2400 CHNS/O analyzer. High-resolution electrospray mass spectra were acquired using a MicroTOF-Q hybrid quadrupole time-of-flight spectrometer (Bruker Daltonics, Bremen, Germany). UV-visible were recorded on an Evolution 600 spectrophotomer. Steady-state photoluminescence spectra were recorded on a Jobin-Yvon Horiba Fluorolog FL-3-11 spectrofluorimeter. Lifetimes were measured using an IBH 5000F coaxial nanosecond flash lamp. Quantum yields were measured using the Hamamatsu Absolute PL Quantum Yield Measurement System C11347-11. Spectroelectrochemical studies were performed with a micro-Autolab FRA2 Type III (Methrom, Utrecht, Netherlands) potentiostat controlled by NOVA (v.2.1.4) software, and connected to a JASCO V670 spectrophotomer using a DRP-PTGRID-TRANSCELL (DropSens). Cyclic voltammetry measurements were performed using a Voltalab PST050 potentiostat with Pt wire as working electrode, Pt wire as counter electrode, and saturated calomel (SCE) as reference electrode. The experiments were carried out under argon in dichloromethane solutions (10^{-3} M), with $[Bu_4N]PF_6$ as supporting electrolyte (0.1 M). Scan rate was 100 mV s^{-1}. The potentials were referenced to the ferrocene/ferrocenium (Fc/Fc^{+}) couple. $OsH_6(P^iPr_3)_2$,[1] $OsH_2Cl_2(P^iPr_3)_2$,[1] $IrH_5(P^iPr_3)_2$,[2] $IrCl_2H(P^iPr_3)_2$,[2] 6-phenyl-2,2'-bipyridine,[3] 6-methyl-2,2'-bipyridine,[4] $OsH_3\{\kappa^2$-*C*,*N*-$(C_5H_3N\text{-py})\}(P^iPr_3)_2$ (**3**),[5] $OsH_3\{\kappa^2$-*C*,*N*-$[C_5(Me)H_2N\text{-py}]\}(P^iPr_3)_2$ (**4**),[5] $OsH_3\{\kappa^2$-*C*,*N*-$[C_5(Ph)H_2N\text{-py}]\}(P^iPr_3)_2$ (**5**),[5] and $IrH_2\{\kappa^2$-*C*,*N*-$(C_5H_3N\text{-py})\}(P^iPr_3)_2$ (**9**),[5] were prepared as reported previously.

● **Structural Analysis of Complexes 6, 8, and 11.**

X-ray data were collected for the complexes on a Bruker Smart APEX diffractometer equipped with a normal focus, and 2.4 kW sealed tube source (Mo radiation, λ = 0.71073 Å). Data were collected over the complete sphere covering 0.3° in w. Data were corrected for absorption by using a multiscan method applied with the SADABS program.[6] The structures were solved by Patterson or direct methods and refined by full-matrix least squares on F^2 with SHELXL2016,[7] including isotropic and subsequently anisotropic displacement parameters. The hydrogen atoms were observed in the last Fourier Maps or calculated, and refined freely or using a restricted riding model. In the structure of complex **11**, the nitrogen and carbons atoms attached to the iridium atoms of the heterocyclic ligand were observed equally disordered.

Crystal data for **6** (CCDC 2046570): $C_{46}H_{96}N_2Os_2P_4$, M_W 1181.52, orange, irregular block (0.126 x 0.101 x 0.072 mm^3), triclinic, space group P-1, *a*: 9.2084(4) Å, *b*: 12.2277(6) Å, *c*: 12.3820(6) Å, α: 110.2990(10)°, β: 92.3750(10)°, γ: 101.6730(10)°, *V* = 1271.06(10) $Å^3$, *Z* = 1, *Z'* = 0.5, D_{calc}: 1.544 g cm^{-3}, F(000): 598, T = 100(2) K, μ 5.151 mm^{-1}. 22632 measured reflections (2θ: 3-57°, ω scans 0.3°), 6090 unique (R_{int} = 0.0261); min./max. transm. factors 0.667/0.862. Final agreement factors were R^1 = 0.0175 (5732 observed reflections, I > 2σ (I)) and wR^2 = 0.0406; data/restraints/parameters 6090/3/ 265; GoF = 1.003. Largest peak and hole: 0.902 (close to osmium atoms) and -0.616 e/ $Å^3$.

Crystal data for **8** (CCDC 2046571): $C_{52}H_{98}N_2Os_2P_4$ x 0.5(C_7H_8), M_W 1301.66, orange, irregular block (0.142 x 0.101 x 0.091 mm^3), triclinic, space group P-1, *a*: 12.1513(6) Å, *b*: 12.4558(6) Å, *c*: 20.3165(10) Å, α: 98.7680(10)°, β: 91.9120(10)°, γ: 110.7430(10)°, *V* = 2829.3(2) $Å^3$, *Z* = 2, *Z'* = 1, D_{calc}: 1.528 g cm^{-3}, F(000): 1322, T = 100(2) K, μ 4.636 mm^{-1}. 50861 measured reflections (2θ: 3-57°, ω scans 0.3°), 13684 unique (R_{int} = 0.0385); min./max. transm. factors 0.716/0.862. Final agreement factors were R^1 = 0.0251 (11119 observed reflections, I > 2σ(I)) and wR^2 = 0.0565; data/restraints/parameters 13684/5/ 631; GoF = 1.020. Largest peak and hole: 1.491 (close to osmium atoms) and -0.618 e/ $Å^3$.

Crystal data for **11** (CCDC 2046572): $C_{46}H_{94}Ir_2N_2P_4$, M_W 1183.51, yellow, irregular block (0.269 x 0.187 x 0.081 mm^3), monoclinic, space group $P2_1/m$, *a*: 11.9554(6) Å, *b*: 24.5517(13) Å, *c*: 9.4888(5) Å, *β*: 114.3730(10)°, *V* = 2537.0(2) $Å^3$, *Z* = 2, *Z'* = 0.5, D_{calc}: 1.549 g cm^{-3}, F(000): 1196, T = 100(2) K, μ 5.398 mm^{-1}. 16787 measured reflections (2θ: 3-57°, ω scans 0.3°), 3184 unique (R_{int} = 0.0216); min./max. transm. factors 0.511/0.862. Final agreement factors were R^1 = 0.0148 (3073 observed reflections, $I > 2\sigma(I)$) and wR^2 = 0.0362; data/restraints/parameters 3184/1/ 158; GoF = 1.054. Largest peak and hole: 1.019 (close to iridium atoms) and -0.595 e/ $Å^3$.

• **NMR Spectra**

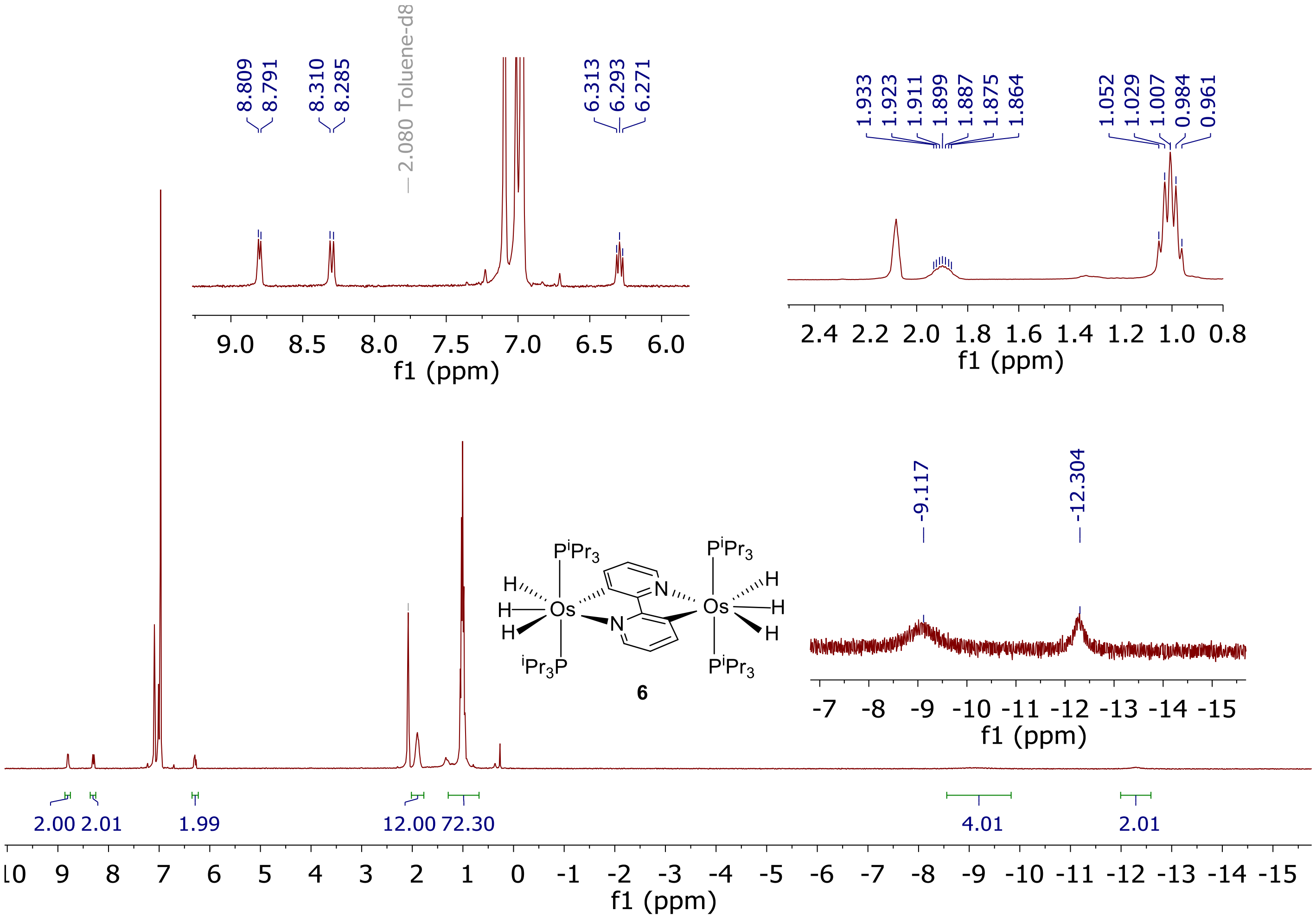

Figure S1. ^{1}H NMR spectrum (300.13 MHz, toluene-d_8, 298 K) of compound **6**.

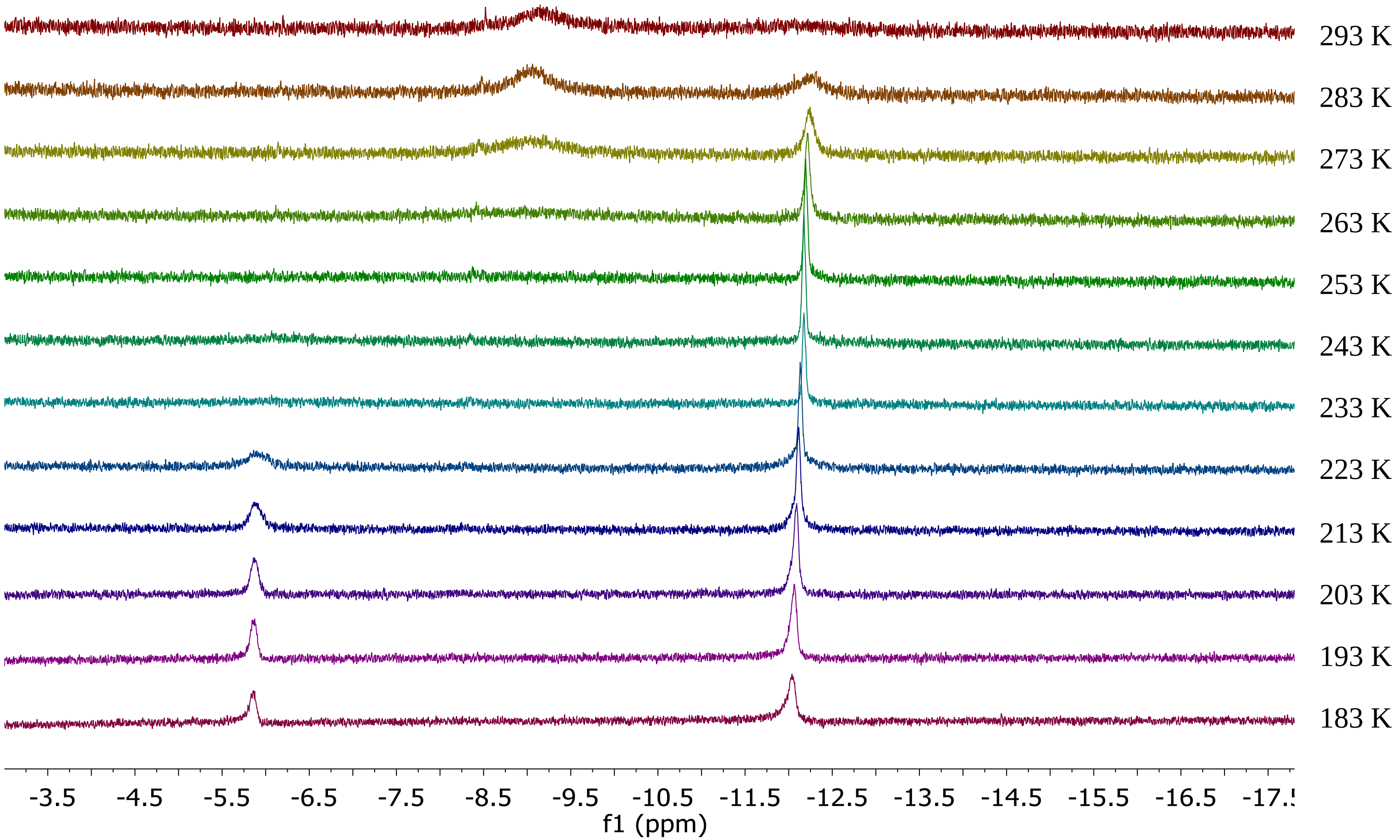

Figure S2. High field region of the ^{1}H NMR spectra (300.13 MHz, toluene-d_8) of compound **6** as a function of the temperature.

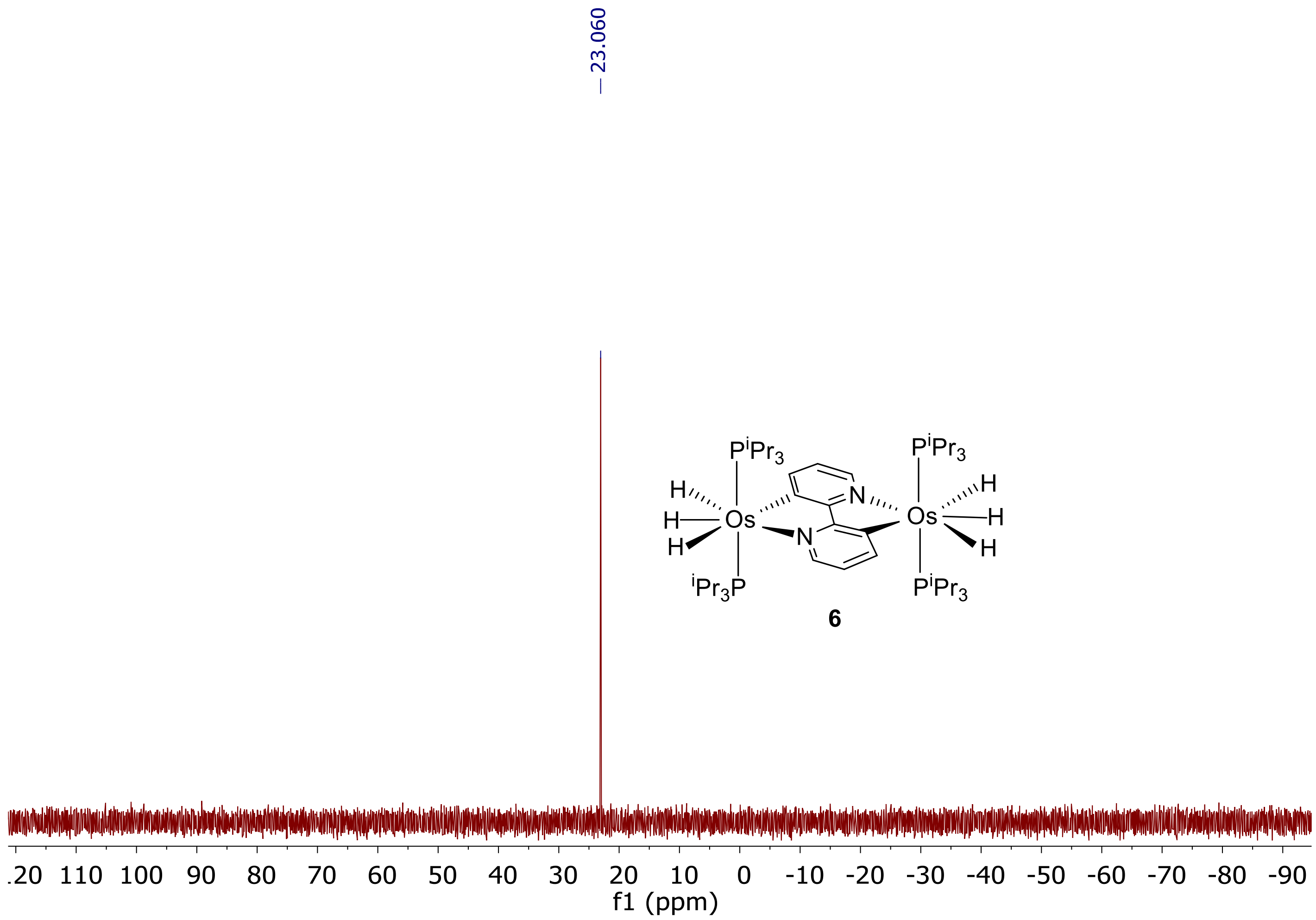

Figure S3. $^{31}P\{^1H\}$ NMR spectrum (121.49 MHz, toluene-d_8, 298 K) of compound **6**.

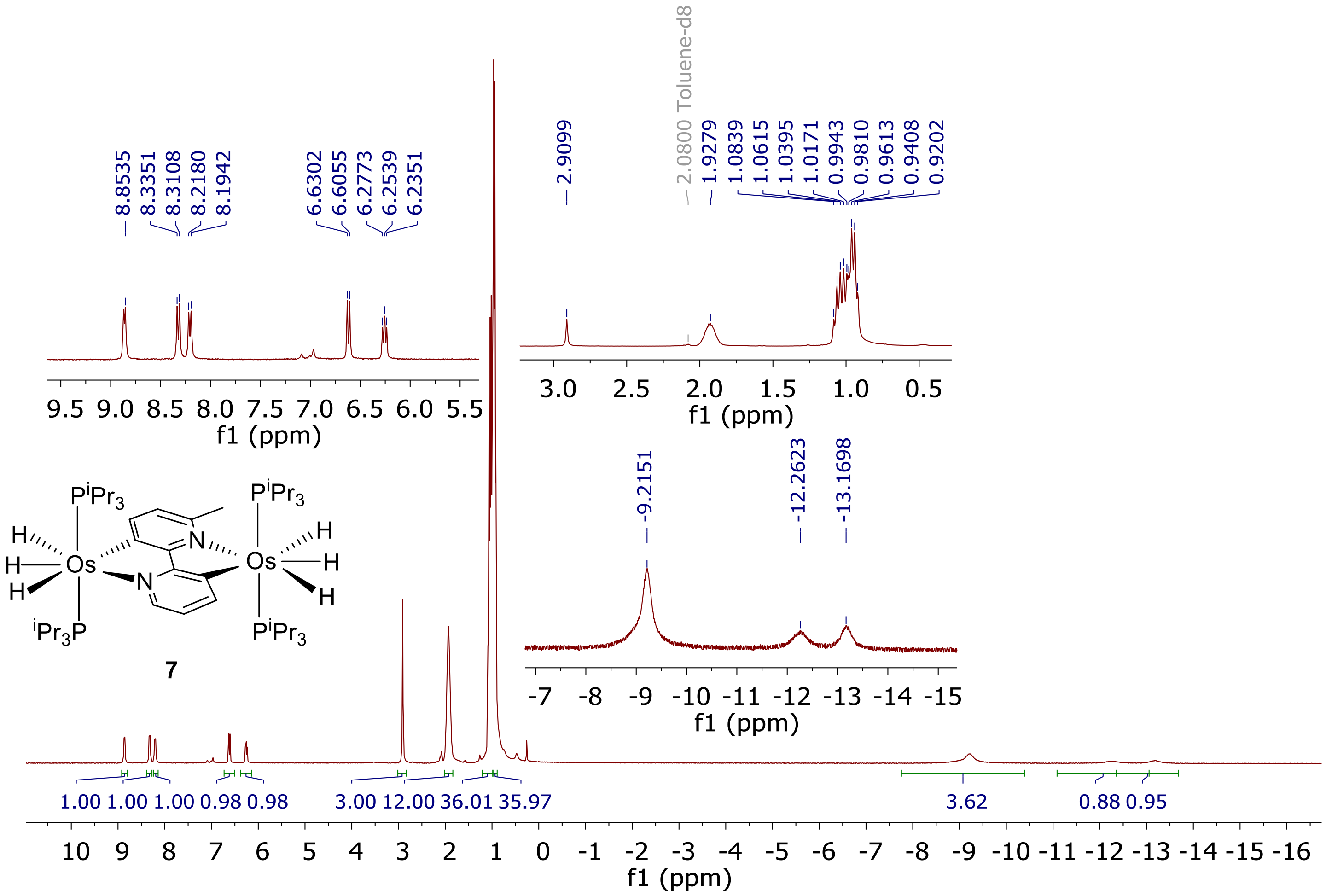

Figure S4. ^{1}H NMR spectrum (300.13 MHz, toluene-d_8, 298 K) of compound **7**.

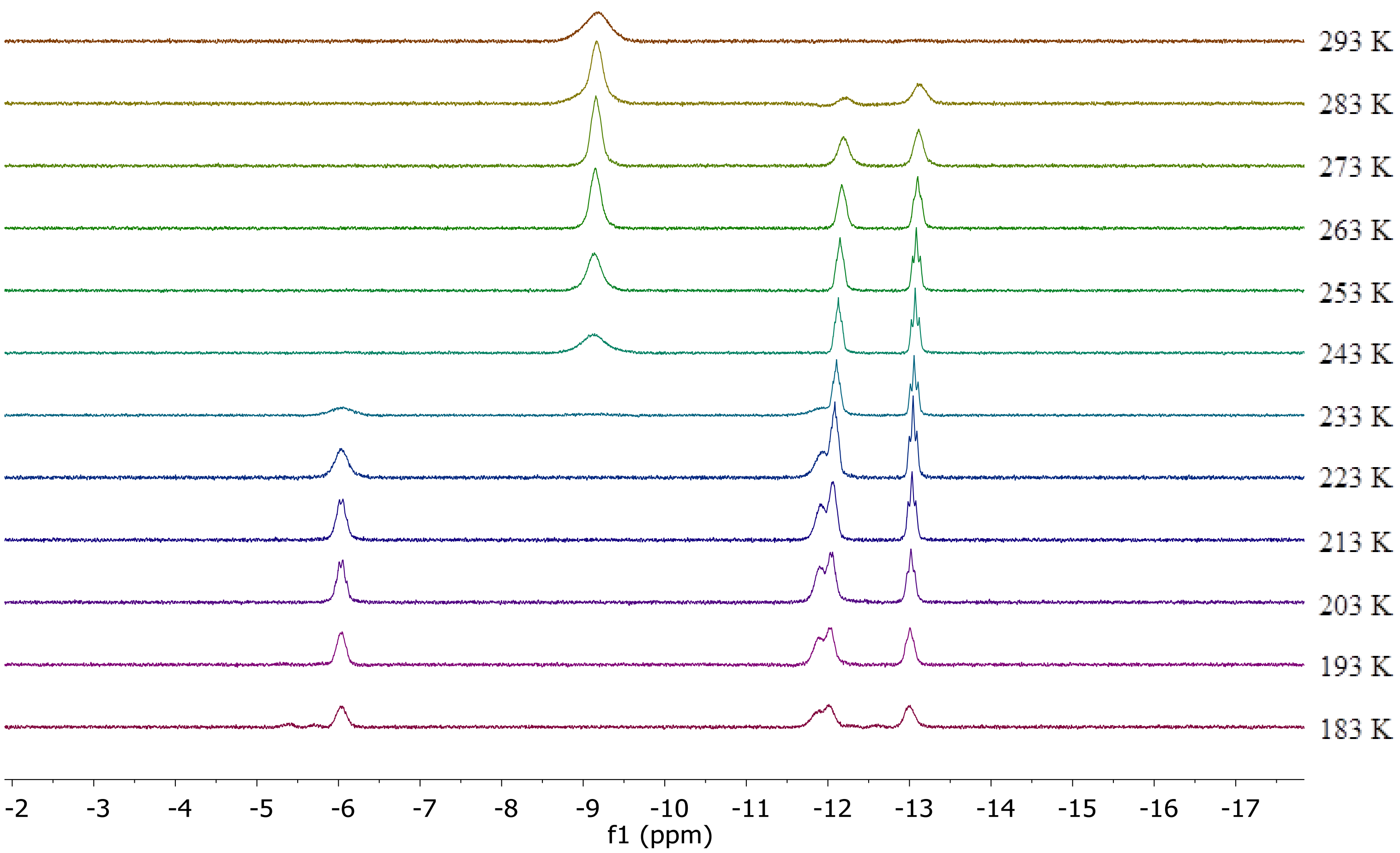

Figure S5. High field region of the ^{1}H NMR spectra (300.13 MHz, toluene-d_8) of compound **7** as a function of the temperature.

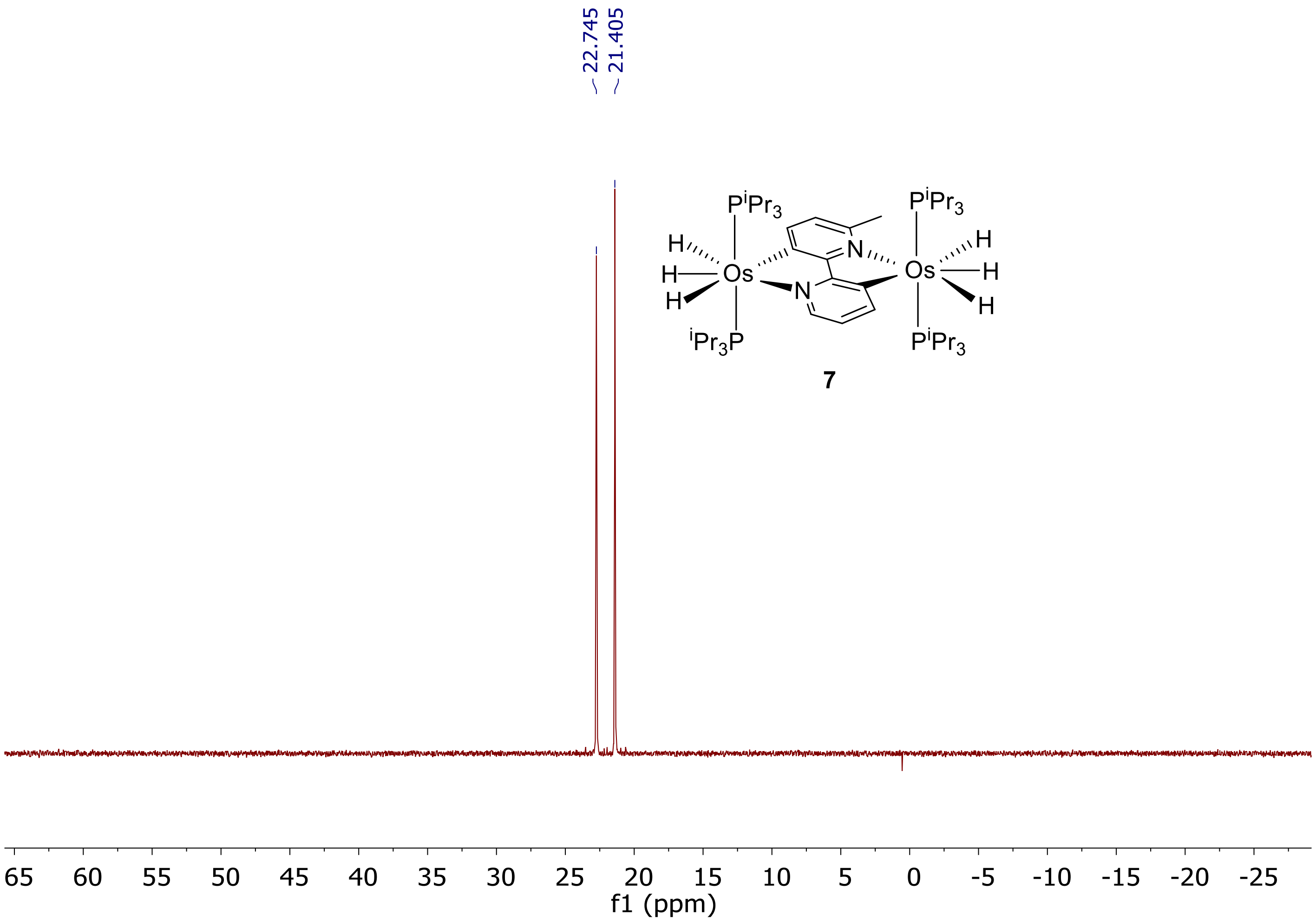

Figure S6. $^{31}P\{^{1}H\}$ NMR spectrum (121.49 MHz, toluene-d_8, 298 K) of compound **7**.

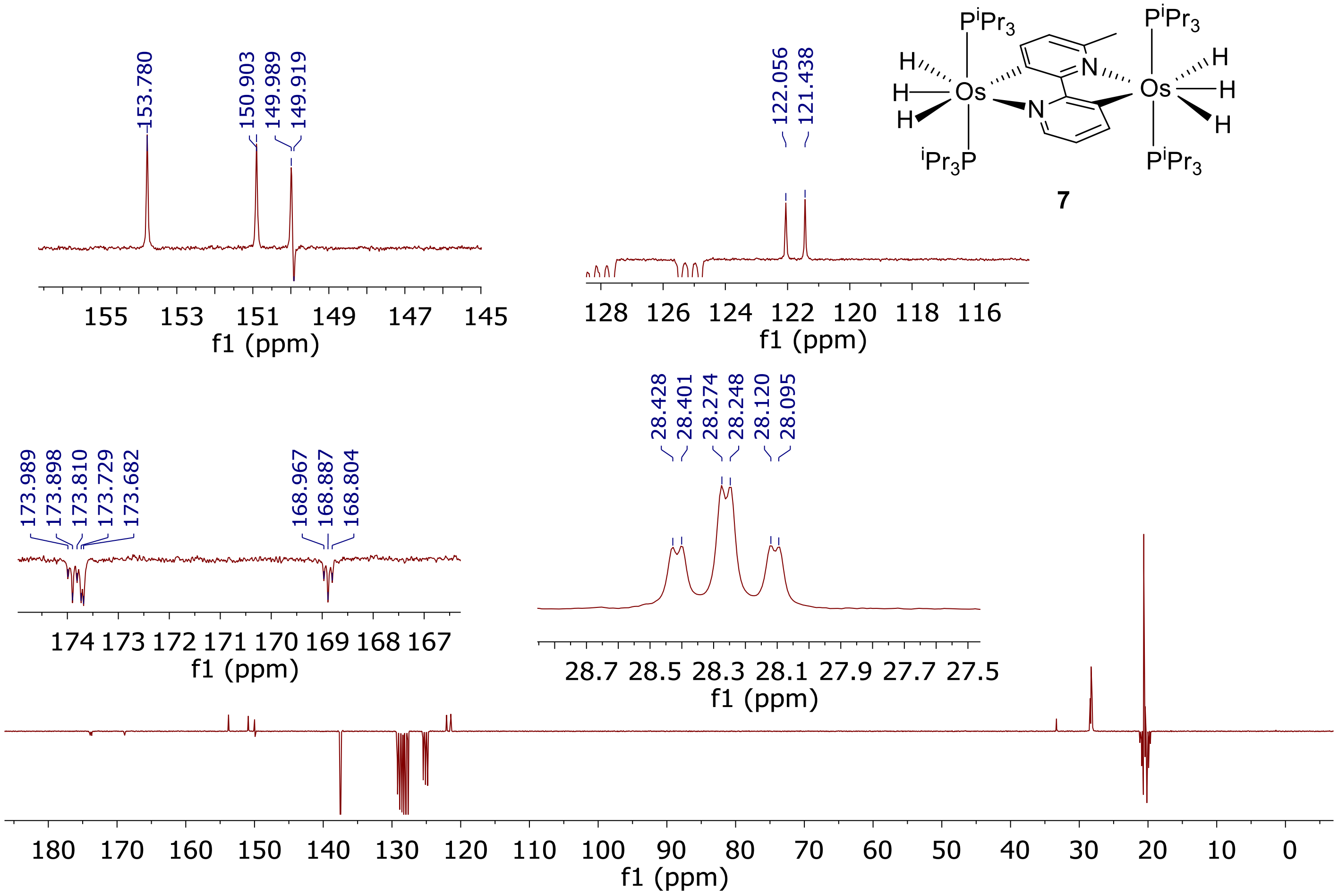

Figure S7. $^{13}C\{^{1}H\}$-apt NMR spectrum (75.48 MHz, toluene-d_8, 298 K) of compound **7**.

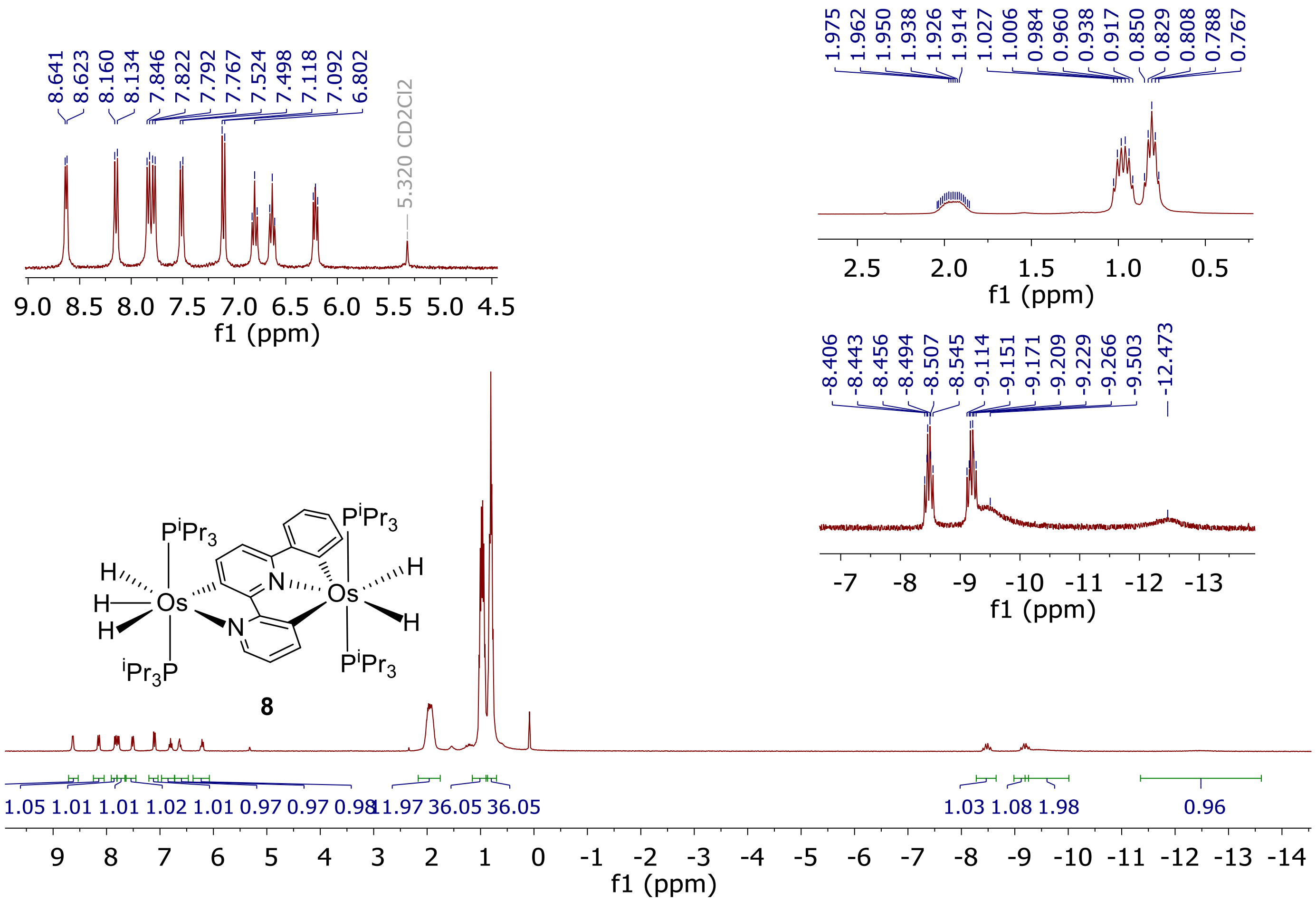

Figure S8. ^{1}H NMR spectrum (300.13 MHz, CD_2Cl_2, 298 K) of compound **8**.

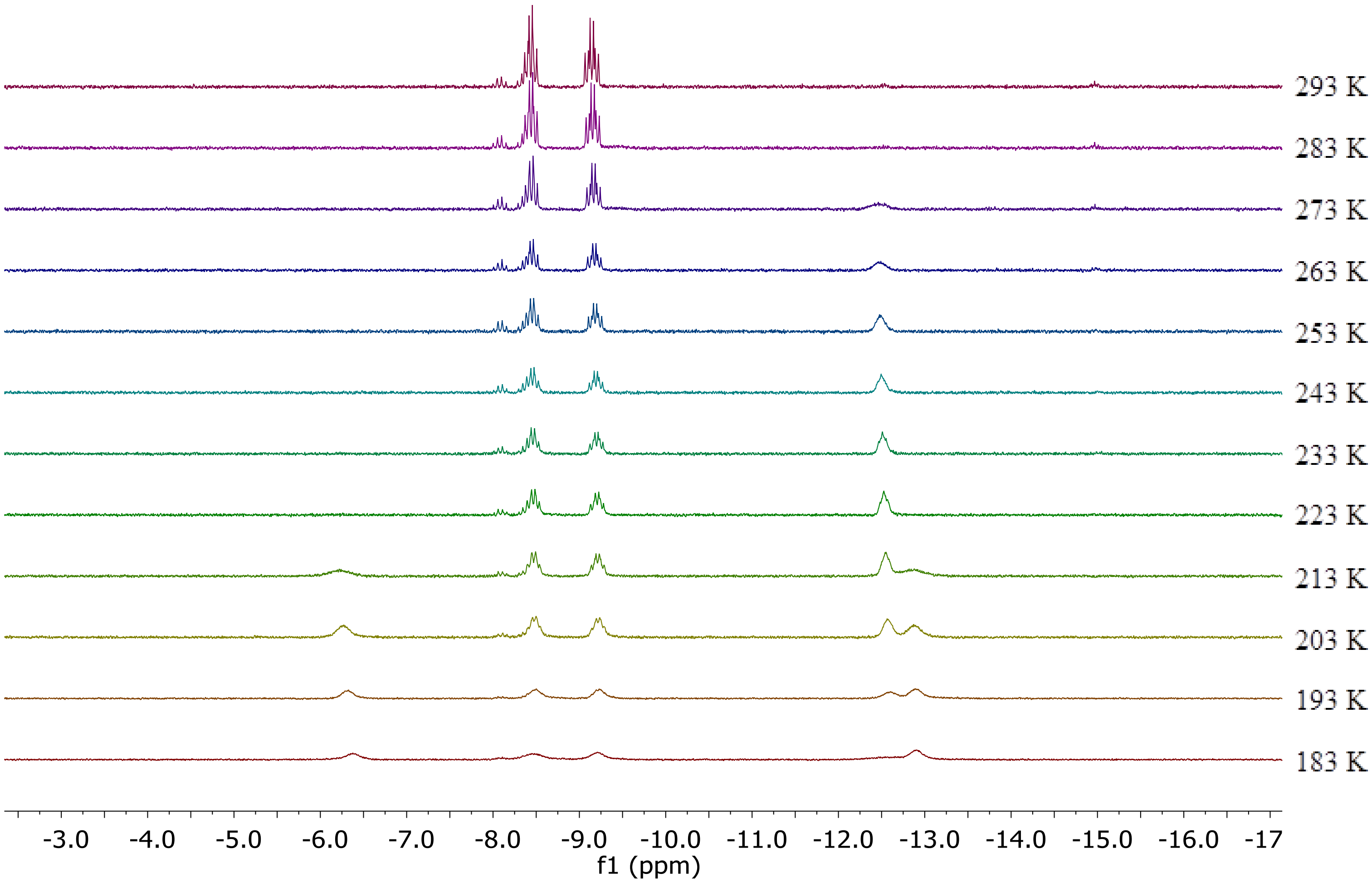

Figure S9. High field region of the ^{1}H NMR spectra (300.13 MHz, CD_2Cl_2) of compound **8** as a function of the temperature.

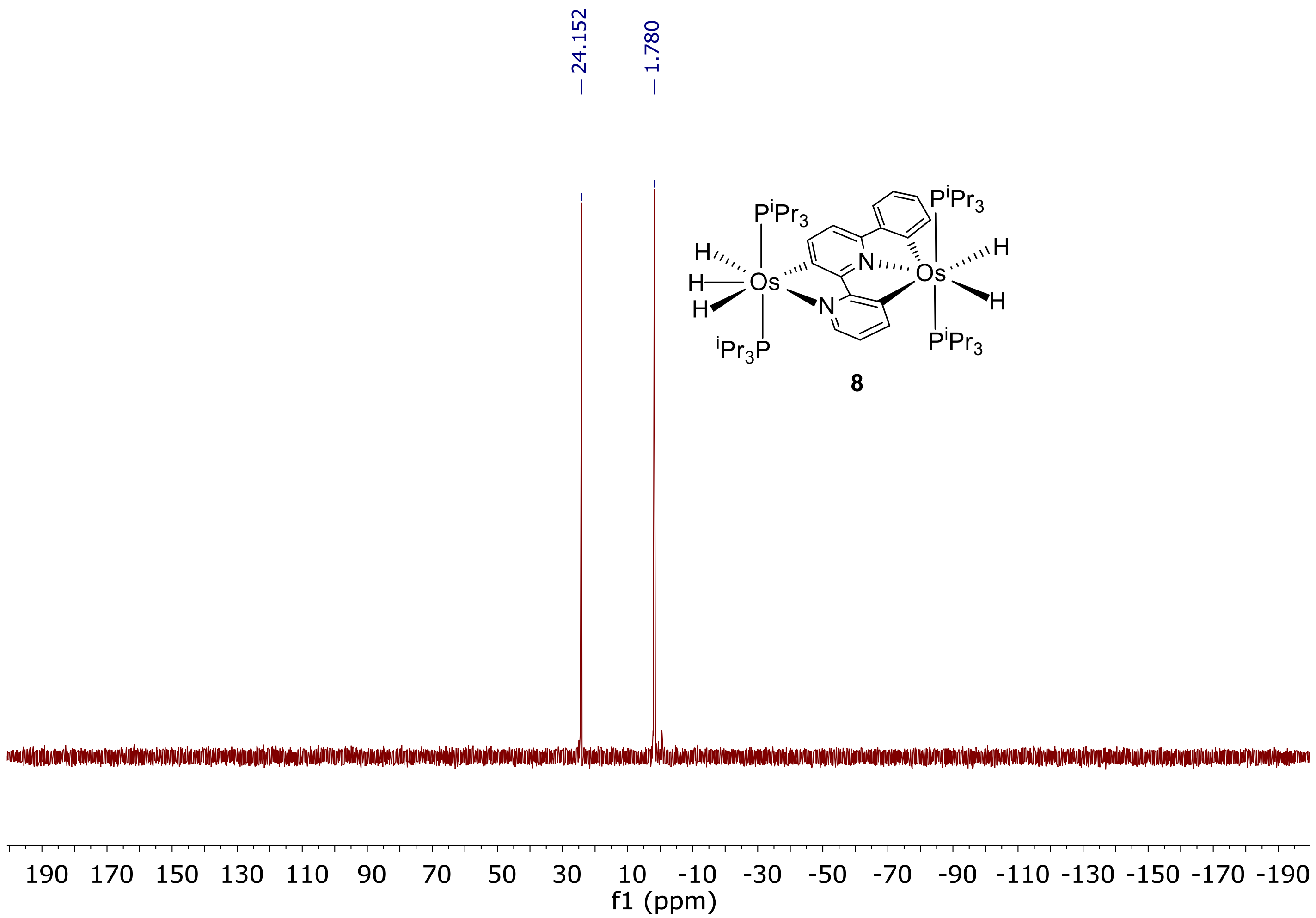

Figure S10. $^{31}P\{^{1}H\}$ NMR spectrum (121.49 MHz, CD_2Cl_2, 298 K) of compound **8**.

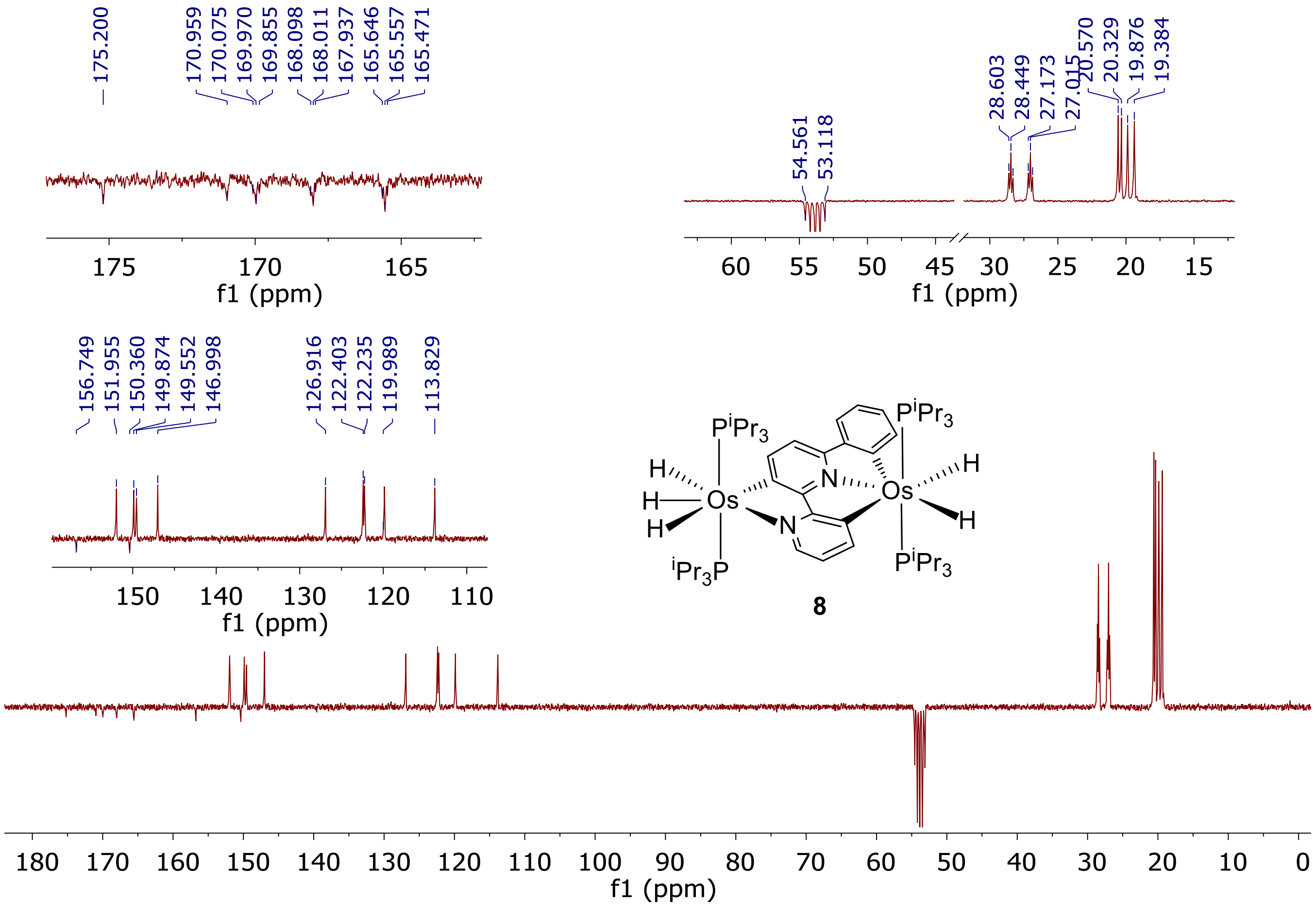

Figure S11. $^{13}C\{^1H\}$-apt NMR spectrum (75.48 MHz, CD_2Cl_2, 298 K) of compound **8**.

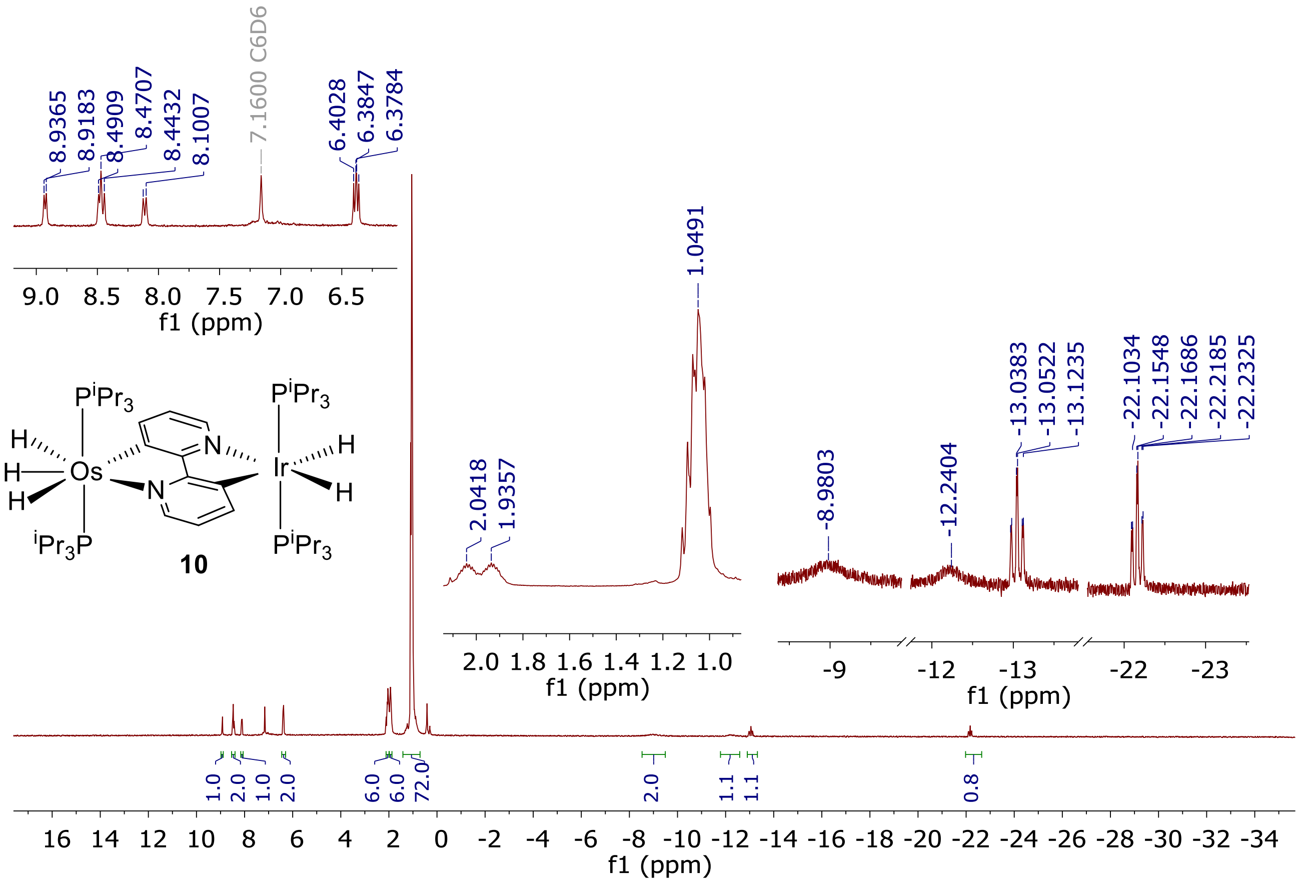

Figure S12. ^{1}H NMR spectrum (300.13 MHz, C_6D_6, 298 K) of compound **10**.

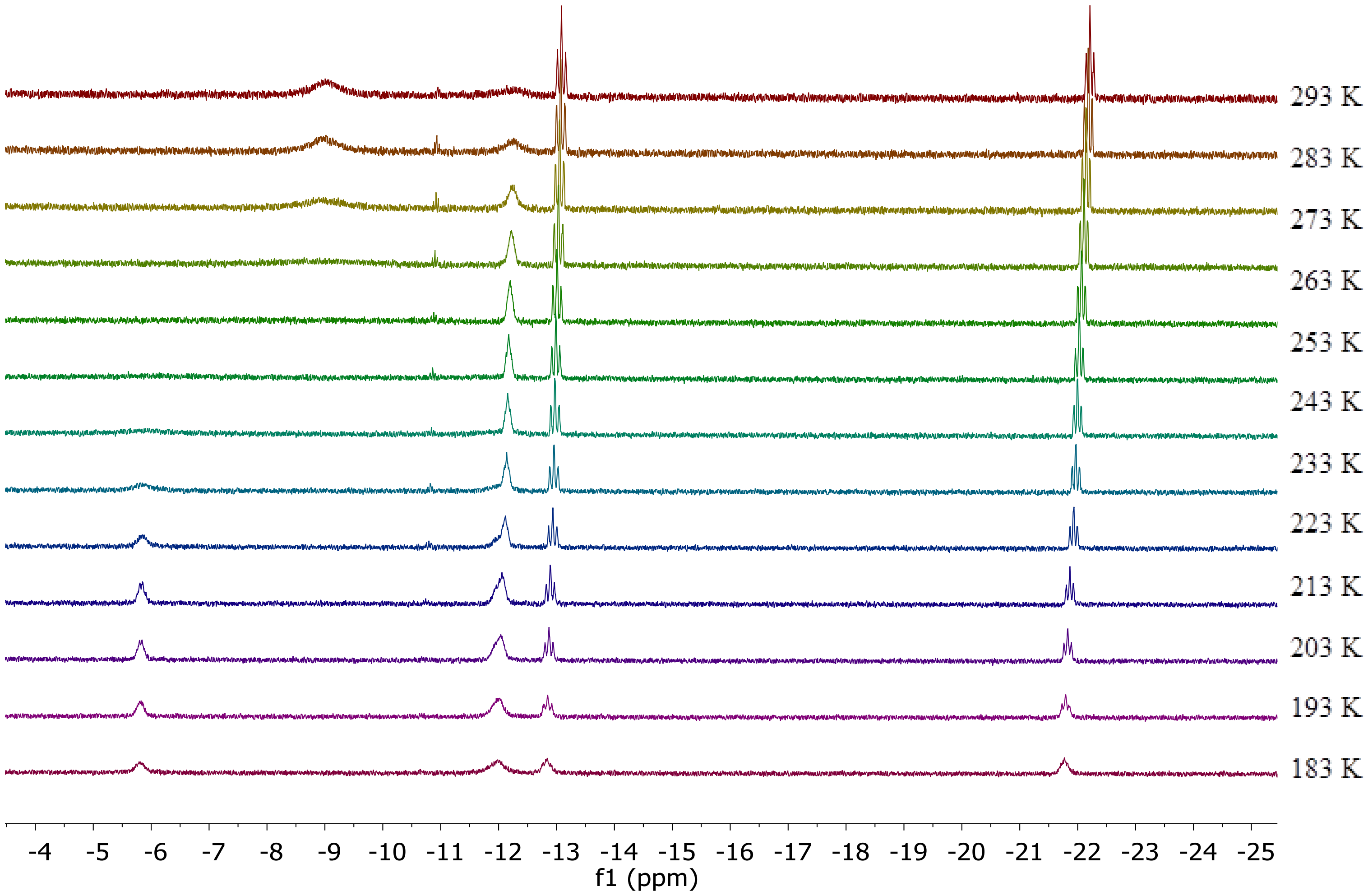

Figure S13. High field region of the ^{1}H NMR spectra (300.13 MHz, toluene-d_8) of compound **10** as a function of the temperature.

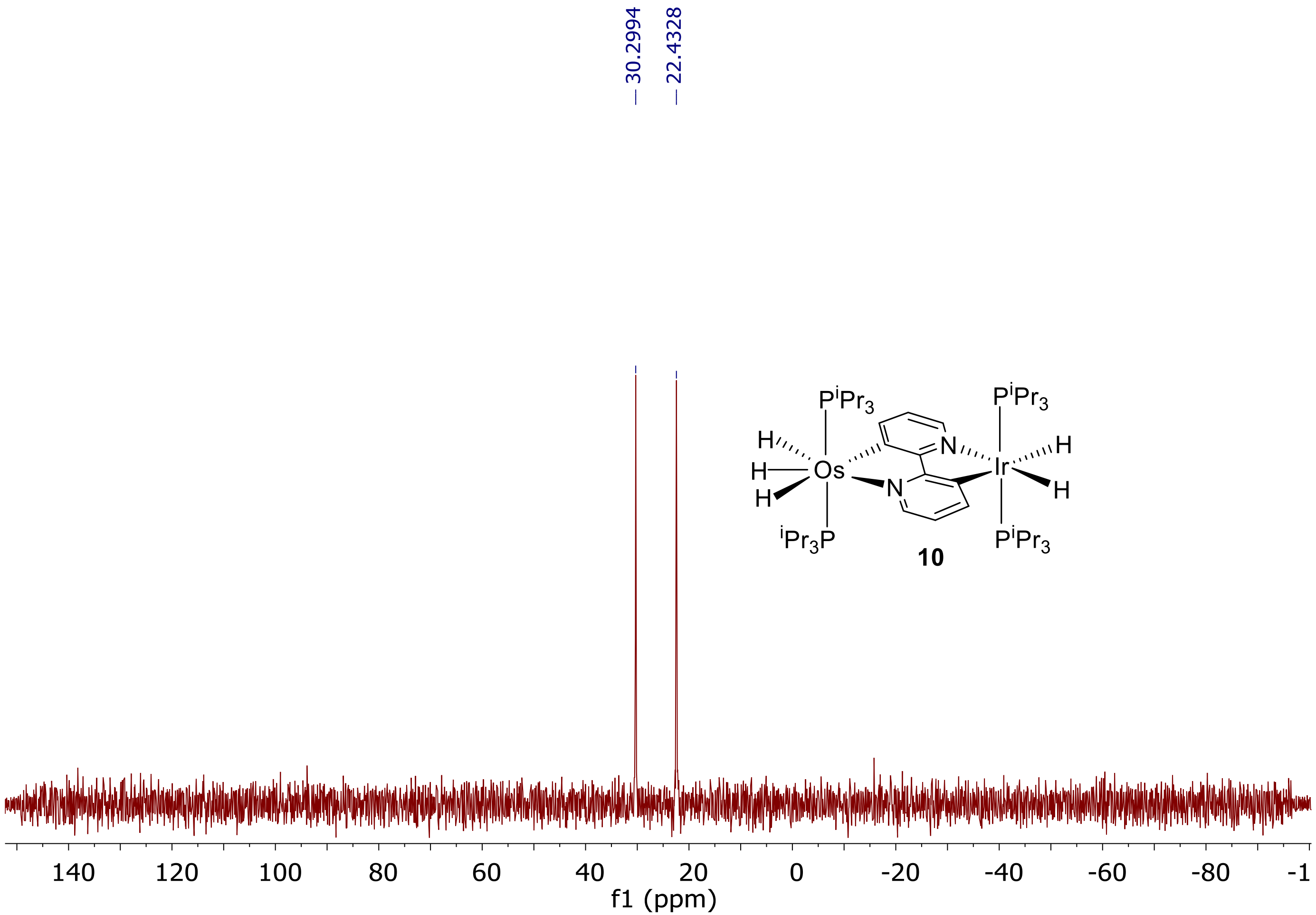

Figure S14. $^{31}P\{^1H\}$ NMR spectrum (121.49 MHz, C_6D_6, 298 K) of compound **10**.

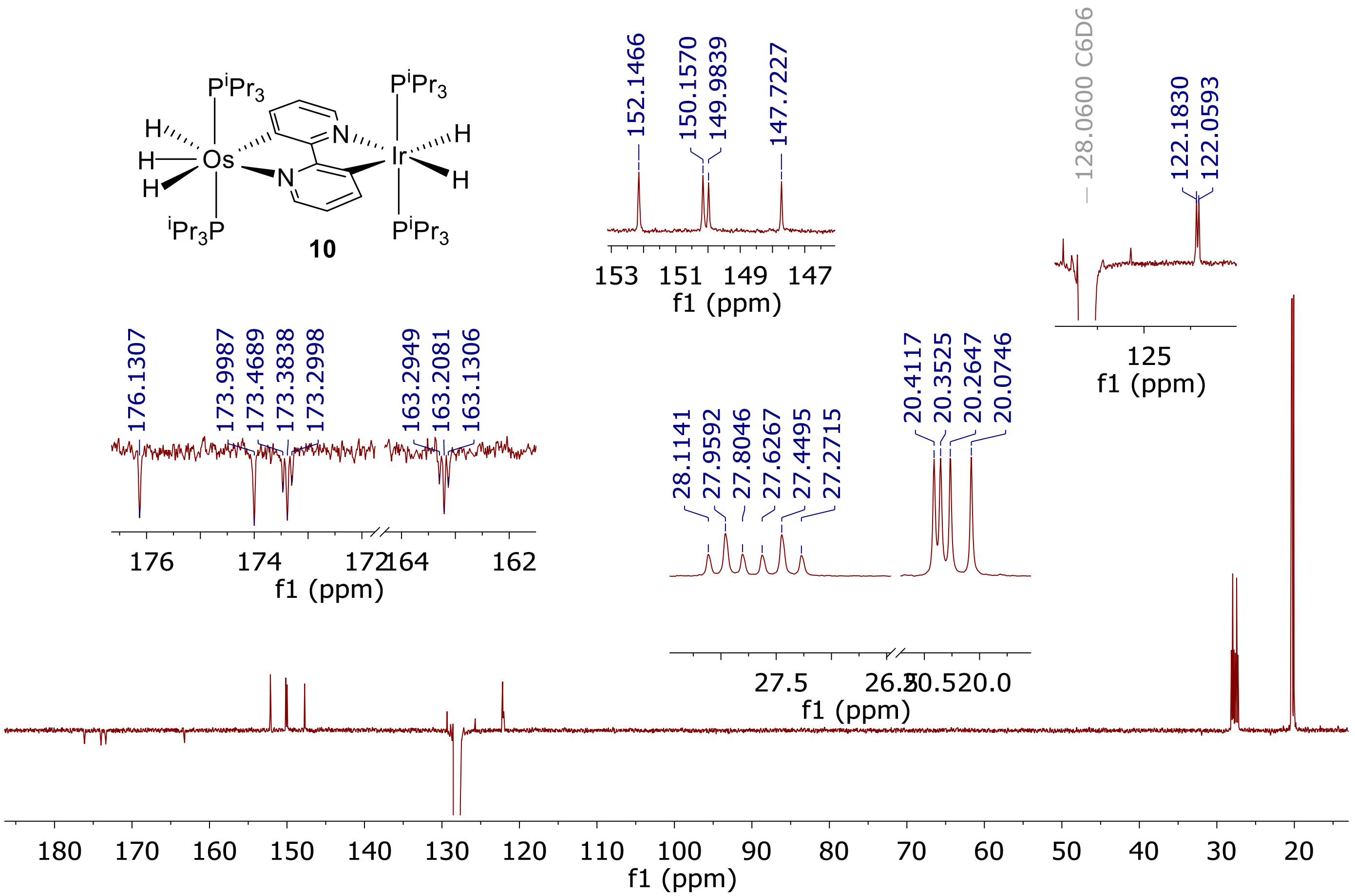

Figure S15. $^{13}C\{^{1}H\}$-apt NMR spectrum (75.48 MHz, C_6D_6, 298 K) of compound **10**.

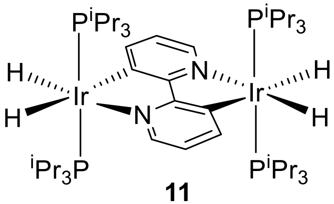

11

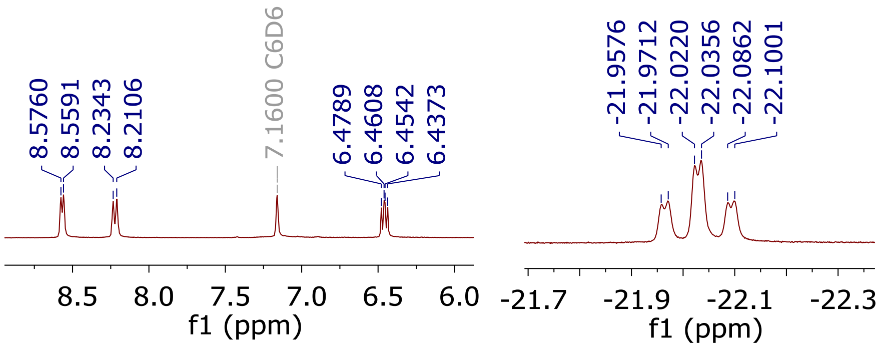

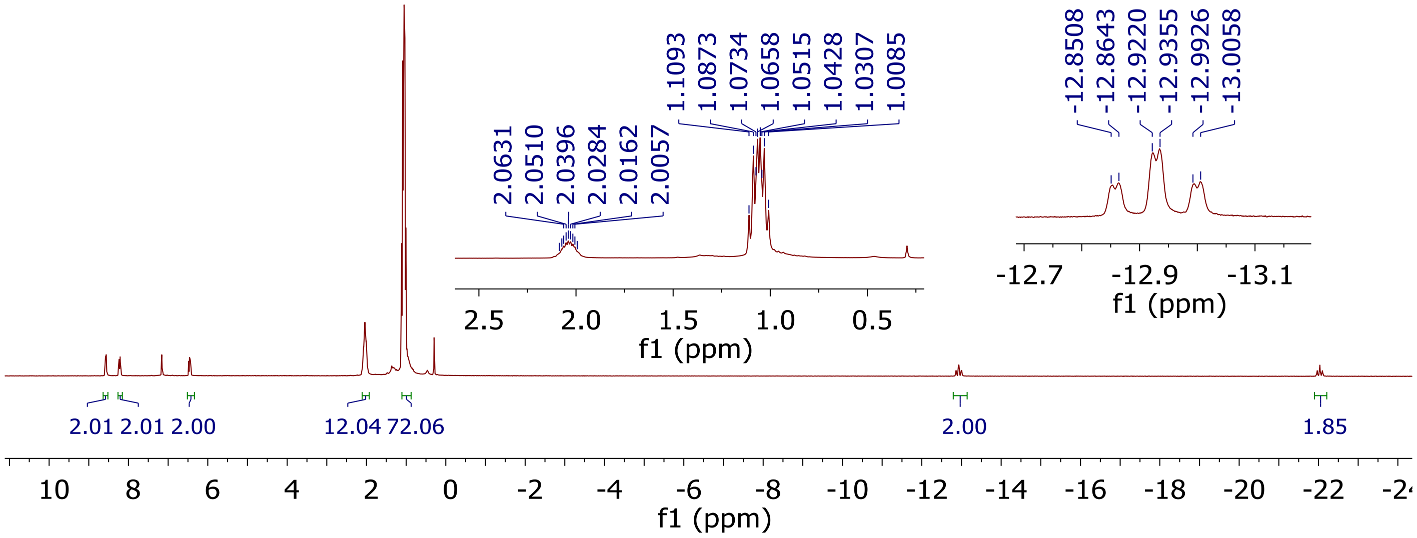

Figure S16. ^{1}H NMR spectrum (300.13 MHz, C_6D_6, 298 K) of compound **11**.

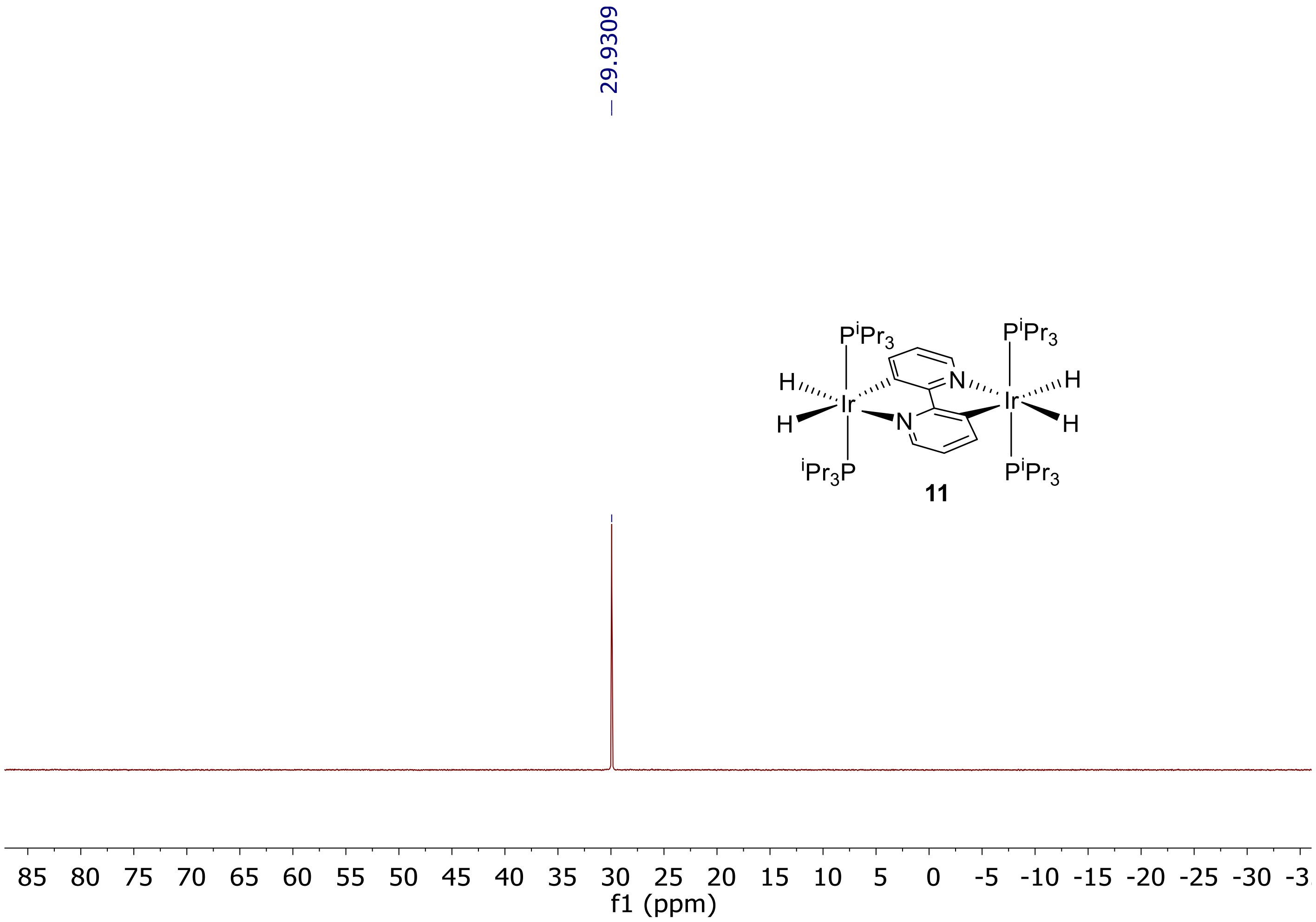

Figure S17. $^{31}P\{^1H\}$ NMR spectrum (121.49 MHz, C_6D_6, 298 K) of compound **11**.

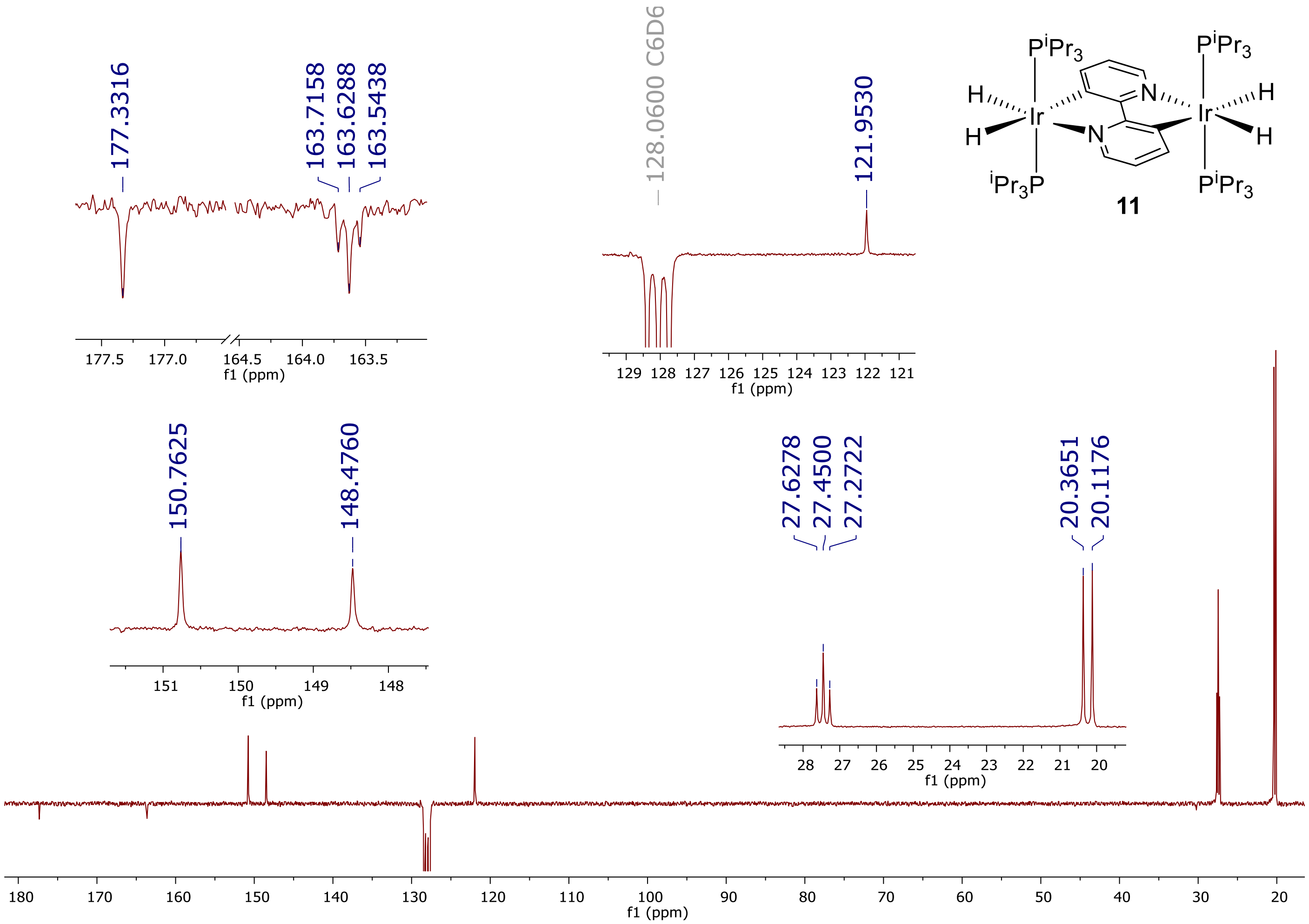

Figure S18. $^{13}C\{^1H\}$-apt NMR spectrum (75.48 MHz, C_6D_6, 298 K) of compound **11**.

● **Computational Details**

All calculations were performed at the DFT level using the B3LYP functional[8] supplemented with the Grimme's dispersion correction D3[9] as implemented in Gaussian09.[10] Os and Ir atoms were described by means of an effective core potential SDD for the inner electron[11] and its associated double-ζ basis set for the outer ones, complemented with a set of f-polarization functions for osmium.[12] The 6-31G** basis set was used for the H, C, N, and P.[13] All minima were verified to have no negative frequencies. The geometries were fully optimized in in THF (ε = 7.4257) and dichloromethane (ε = 8.93) solvents using the continuum SMD model.[14] We performed TD-DFT calculations at the same level of theory in THF and CH_2Cl_2 calculating the lowest 50 excitations. The UV/vis absorption spectra were obtained by using the GaussSum 3 software.[15] The phosphorescence emission compares well with the 0-0 transition calculated taking into account the zero point energies (zpe) of the geometries of both the optimized S_0 and T_1 states in THF.

● **Energies of Optimized Structures**

Complex 3-S_0 (THF)

E= -1981.56005037 a.u.

Sum of electronic and zero-point Energies= -1980.811153

Sum of electronic and thermal Energies= -1980.769955

Sum of electronic and thermal Enthalpies= -1980.769011

Sum of electronic and thermal Free Energies= -1980.882093

Complex 3-T_1 (THF)

E= -1981.46737940 a.u.

Sum of electronic and zero-point Energies= -1980.734068

Sum of electronic and thermal Energies= -1980.692228

Sum of electronic and thermal Enthalpies= -1980.691284

Sum of electronic and thermal Free Energies= -1980.806652

Complex 4-S_0 (THF)

E= -2020.88592522 a.u.

Sum of electronic and zero-point Energies= -2020.109700

Sum of electronic and thermal Energies= -2020.066541

Sum of electronic and thermal Enthalpies= -2020.065597

Sum of electronic and thermal Free Energies= -2020.183450

Complex 4-T_1 (THF)

E= -2020.80678788 a.u.

Sum of electronic and zero-point Energies= -2020.034021

Sum of electronic and thermal Energies= -2019.990250

Sum of electronic and thermal Enthalpies= -2019.989306

Sum of electronic and thermal Free Energies= -2020.109362

Complex 5-S_0 (THF)

E= -2212.64382705 a.u.

Sum of electronic and zero-point Energies= -2211.813436

Sum of electronic and thermal Energies= -2211.767594

Sum of electronic and thermal Enthalpies= -2211.766650

Sum of electronic and thermal Free Energies= -2211.890145

Complex 5-T_1 (THF)

E= -2212.55889421 a.u.

Sum of electronic and zero-point Energies= -2211.732697

Sum of electronic and thermal Energies= -2211.685801

Sum of electronic and thermal Enthalpies= -2211.684856

Sum of electronic and thermal Free Energies= -2211.812451

Complex 6-S_o (THF)

E= -3467.39675170 a.u.

Sum of electronic and zero-point Energies= -3505.340480

Sum of electronic and thermal Energies= -3505.266050

Sum of electronic and thermal Enthalpies= -3505.265106

Sum of electronic and thermal Free Energies= -3505.441138

Complex 6-T_1 (THF)

E= -3467.31408546 a.u.

Sum of electronic and zero-point Energies= -3465.977791

Sum of electronic and thermal Energies= -3465.903267

Sum of electronic and thermal Enthalpies= -3465.902323

Sum of electronic and thermal Free Energies= -3466.082120

Complex 6 (CH_2Cl_2)

E= -3467.71187007 a.u.

Complex 6 cationic (CH_2Cl_2)

E= -3467.55527754 a.u.

Complex 6 dicationic (CH_2Cl_2)

E= -3467.38051277 a.u.

Complex 6 tricationic (CH_2Cl_2)

E= -3467.20019244 a.u.

Complex 7-S_0 (THF)

E= -3506.70879833 a.u.

Sum of electronic and zero-point Energies= -3505.340480

Sum of electronic and thermal Energies= -3505.266050

Sum of electronic and thermal Enthalpies= -3505.265106

Sum of electronic and thermal Free Energies= -3505.441138

Complex 7-T_1 (THF)

E= -3506.62817524 a.u.

Sum of electronic and zero-point Energies= -3505.264074

Sum of electronic and thermal Energies= -3505.188781

Sum of electronic and thermal Enthalpies= -3505.187836

Sum of electronic and thermal Free Energies= -3505.367589

Complex 7 (CH_2Cl_2)

E= -3507.03223251 a.u.

Complex 7 cationic (CH_2Cl_2)

E= -3506.87814198 a.u.

Complex 7 dicationic (CH_2Cl_2)

E= -3506.70401088 a.u.

Complex 7 tricationic (CH_2Cl_2)

E= -3506.52571415 a.u.

Complex 8-S_0 (THF)

E= -697.58752877 a.u.

Sum of electronic and zero-point Energies= -3696.183006

Sum of electronic and thermal Energies= -3696.106339

Sum of electronic and thermal Enthalpies= -3696.105395

Sum of electronic and thermal Free Energies= -3696.286669

Complex 8-T_1 (THF)

E= -3697.51068899 a.u.

Sum of electronic and zero-point Energies= -3696.109567

Sum of electronic and thermal Energies= -3696.032249

Sum of electronic and thermal Enthalpies= -3696.031304

Sum of electronic and thermal Free Energies= -3696.215026

Complex 8 (CH_2Cl_2)

E= -3697.59623633 a.u.

Complex 8 cationic (CH_2Cl_2)

E= -3697.44512806 a.u.

Complex 8 dicationic S_0 (CH_2Cl_2)

E= -3697.27144954 a.u.

Sum of electronic and zero-point Energies= -3695.858981

Sum of electronic and thermal Energies= -3695.783874

Sum of electronic and thermal Enthalpies= -3695.782929

Sum of electronic and thermal Free Energies= -3695.959939

Complex 8 dicationic T_1 (CH_2Cl_2)

E= -3697.26783151 a.u.

Sum of electronic and zero-point Energies= -3695.860529

Sum of electronic and thermal Energies= -3695.783688

Sum of electronic and thermal Enthalpies= -3695.782743

Sum of electronic and thermal Free Energies= -3695.966108

Complex 8 tricationic (CH_2Cl_2)

E= -3697.08336373 a.u.

Complex 10-S_0 (THF)

E= -3480.78542690 a.u.

Sum of electronic and zero-point Energies= -3479.451435

Sum of electronic and thermal Energies= -3479.378529

Sum of electronic and thermal Enthalpies= -3479.377584

Sum of electronic and thermal Free Energies= -3479.553110

Complex 10-T_1 (THF)

E= -3480.70147834 a.u.

Sum of electronic and zero-point Energies= -3479.371285

Sum of electronic and thermal Energies= -3479.297674

Sum of electronic and thermal Enthalpies= -3479.296730

Sum of electronic and thermal Free Energies= -3479.474701

Complex 10 (CH_2Cl_2)

E= -3480.79384939 a.u.

Complex 10 cationic (CH_2Cl_2)

E= -3480.63739182 a.u.

Complex 10 dicationic (CH_2Cl_2)

E= -3480.46353128 a.u.

Complex 10 tricationic (CH_2Cl_2)

E= -3480.27600419 a.u.

Complex 11-S_0 (THF)

E= -3493.86691405 a.u.

Sum of electronic and zero-point Energies= -3492.541719

Sum of electronic and thermal Energies= -3492.468874

Sum of electronic and thermal Enthalpies= -3492.467929

Sum of electronic and thermal Free Energies= -3492.644279

Complex 11-T_1 (THF)

E= -3493.77479664 a.u.

Sum of electronic and zero-point Energies= -3492.453522

Sum of electronic and thermal Energies= -3492.379950

Sum of electronic and thermal Enthalpies= -3492.379006

Sum of electronic and thermal Free Energies= -3492.558169

Complex 11 (CH_2Cl_2)

E= -3493.87558233 a.u.

Complex 11 cationic (CH_2Cl_2)

E= -3493.71142115 a.u.

Complex 11 dicationic (CH_2Cl_2)

E= -3493.53472906 a.u.

Complex 11 tricationic (CH_2Cl_2)

E= -3493.34905943 a.u.

● **UV-vis Spectra of Complexes 3-11 (Observed and Calculated)**

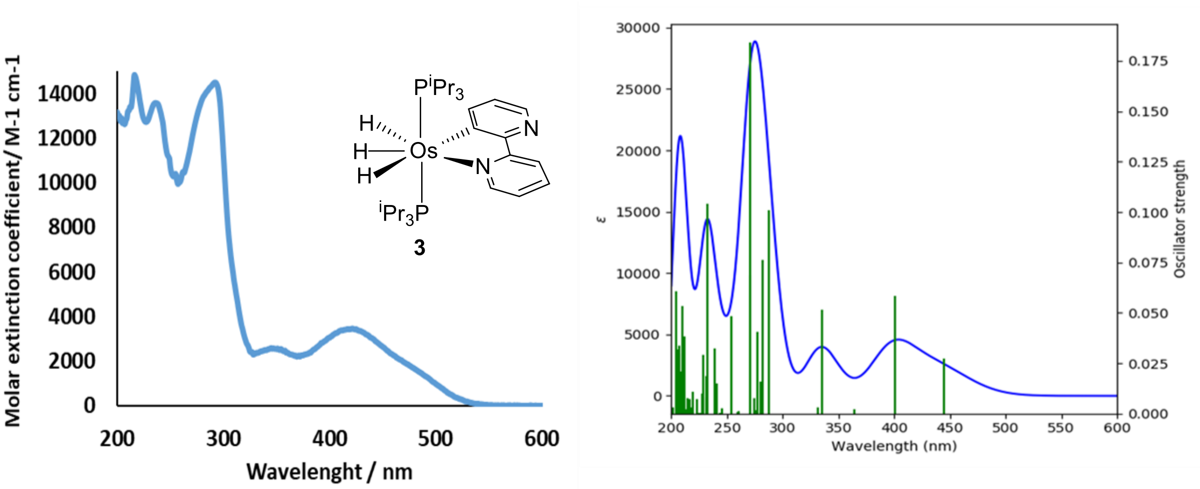

Figure S19. Observed UV-vis of complex **3** in 2-MeTHF (1.0 x 10^{-4} M) and calculated (B3LYP(GD3)//SDD(f)/6-31G**) in THF.

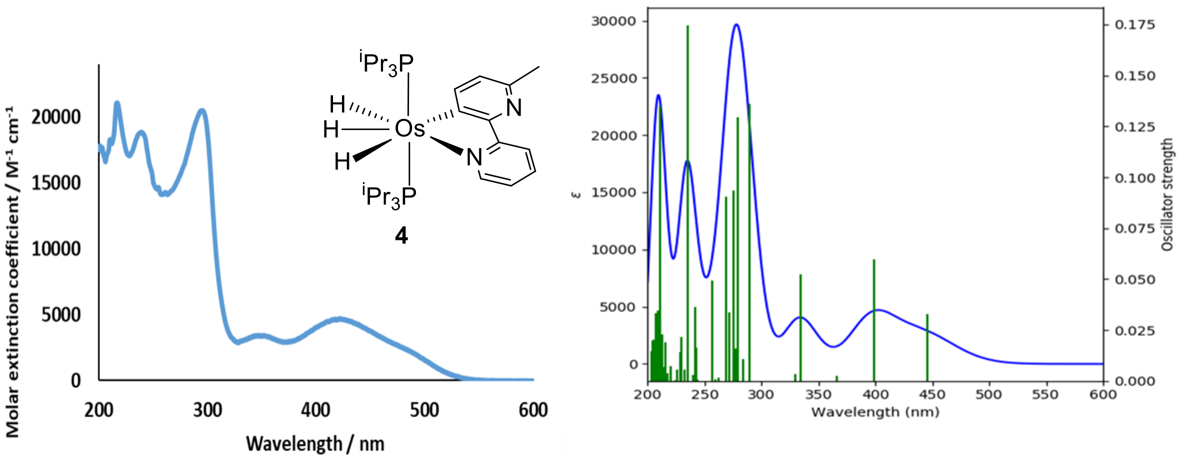

Figure S20. Observed UV-vis of complex **4** in 2-MeTHF (1.0 x 10^{-4} M) and calculated (B3LYP(GD3)//SDD(f)/6-31G**) in THF.

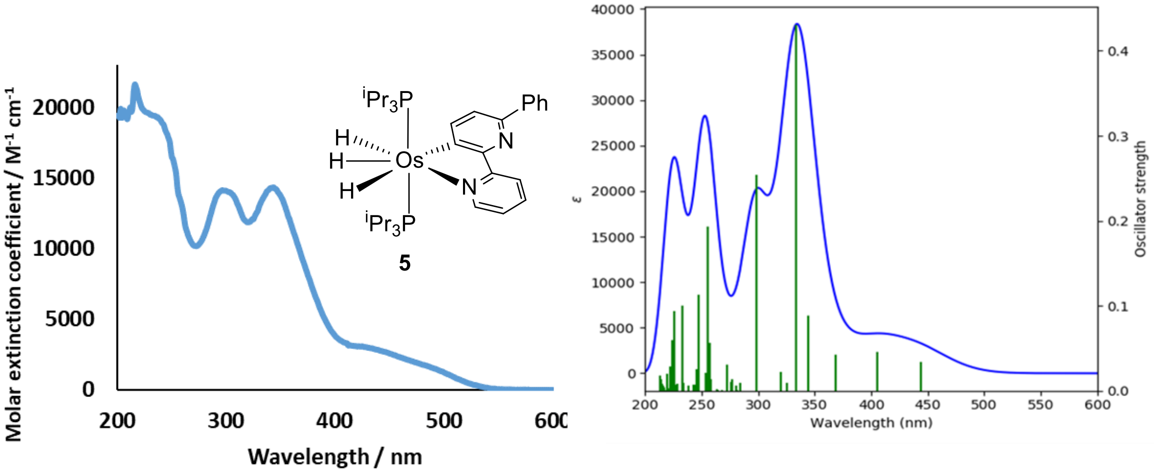

Figure S21. Observed UV-vis of complex **5** in 2-MeTHF (1.0 x 10^{-4} M) and calculated (B3LYP(GD3)//SDD(f)/6-31G**) in THF.

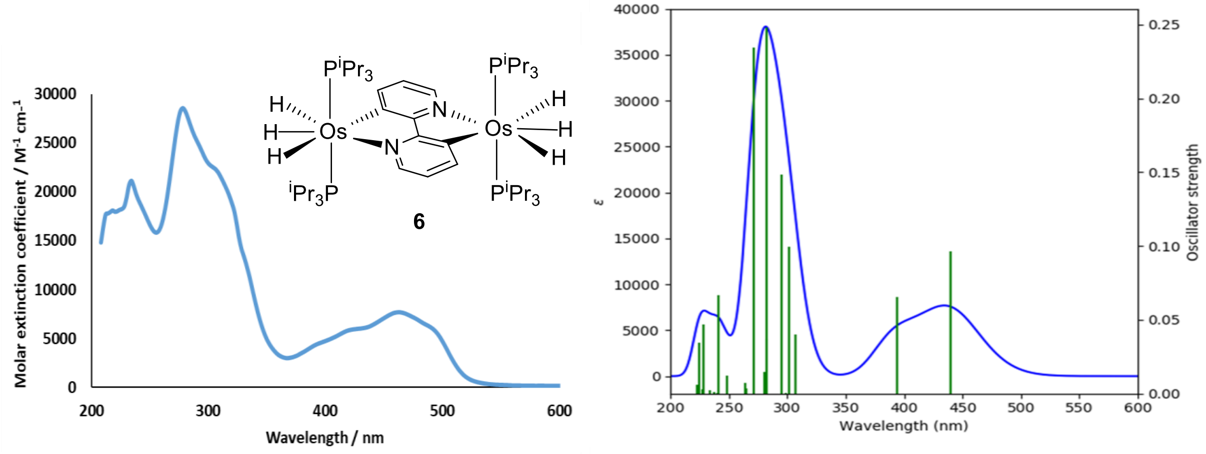

Figure S22. Observed UV-vis of complex **6** in 2-MeTHF (1.0 x 10^{-4} M) and calculated (B3LYP(GD3)//SDD(f)/6-31G**) in THF.

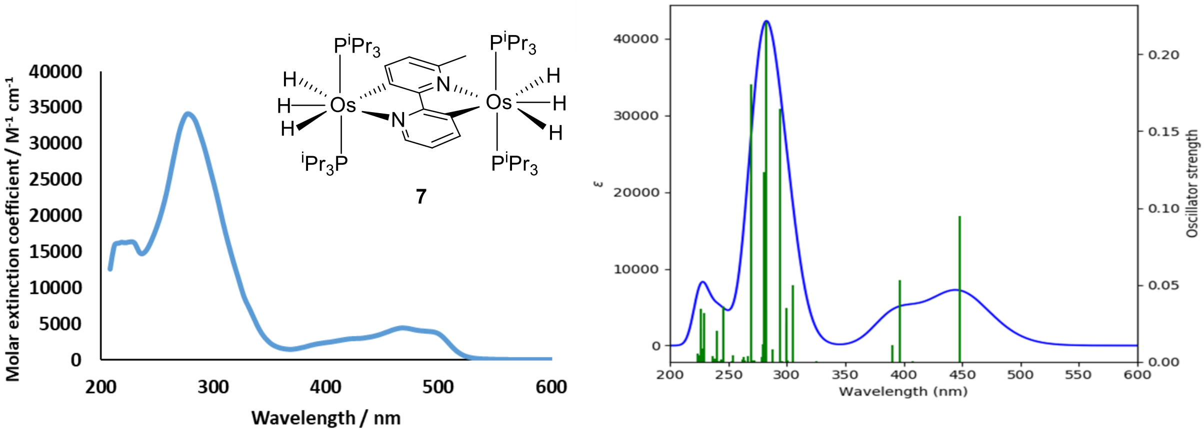

Figure S23. Observed UV-vis of complex **7** in 2-MeTHF (1.0 x 10^{-4} M) and calculated (B3LYP(GD3)//SDD(f)/6-31G**) in THF.

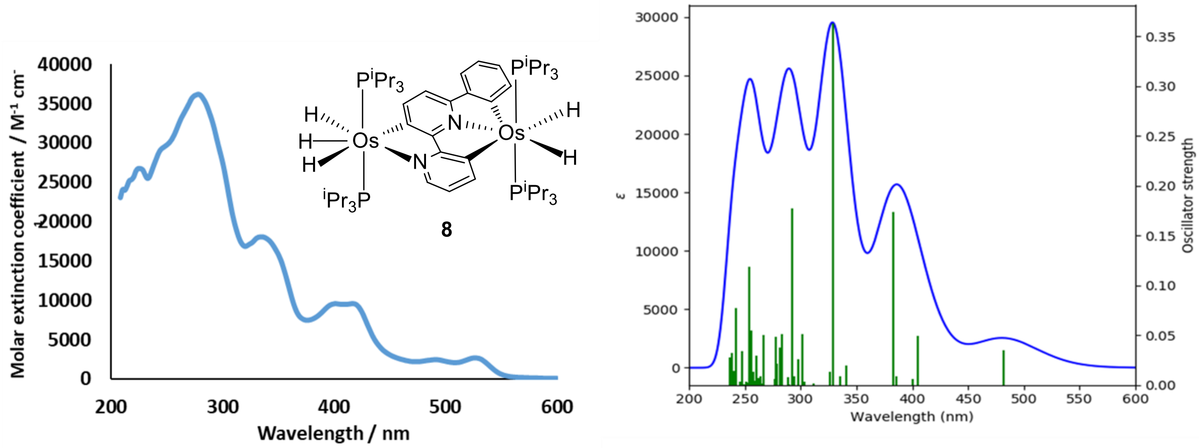

Figure S24. Observed UV-vis of complex **8** in 2-MeTHF (1.0 x 10^{-4} M) and calculated (B3LYP(GD3)//SDD(f)/6-31G**) in THF.

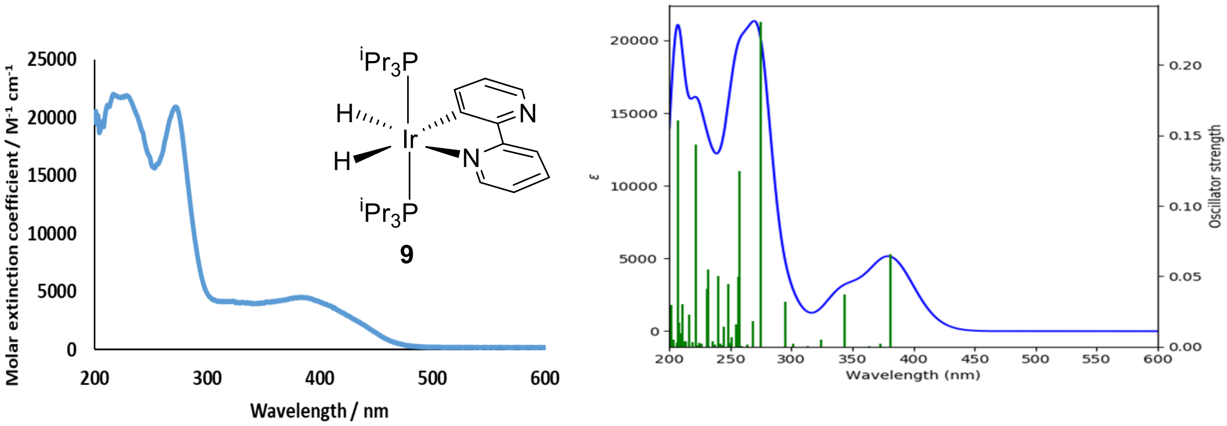

Figure S25. Observed UV-vis of complex **9** in 2-MeTHF (1.0 x 10^{-4} M) and calculated (B3LYP(GD3)//SDD(f)/6-31G**) in THF.

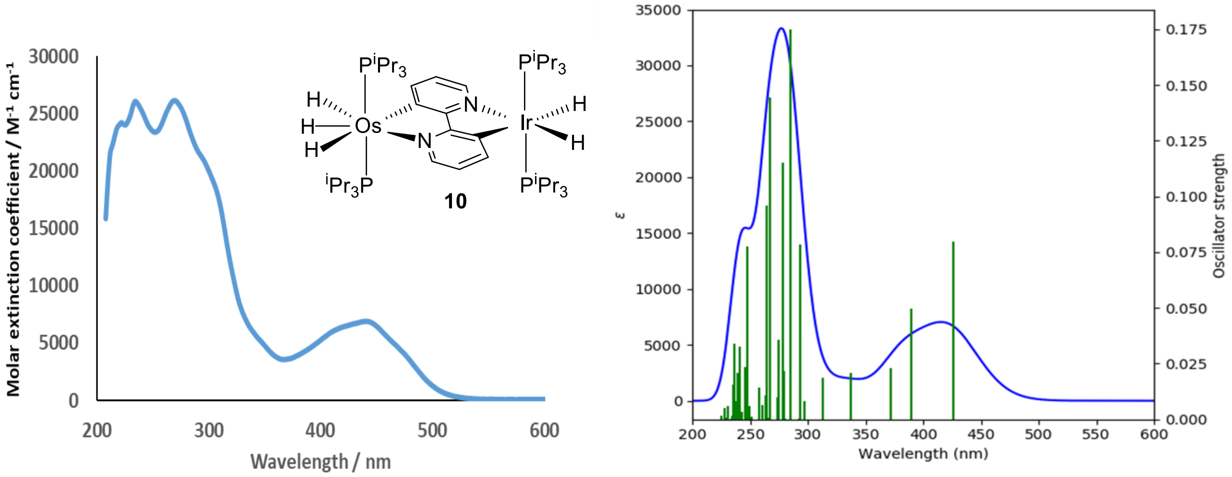

Figure S26. Observed UV-vis of complex **10** in 2-MeTHF (1.0 x 10^{-4} M) and calculated (B3LYP(GD3)//SDD(f)/6-31G**) in THF.

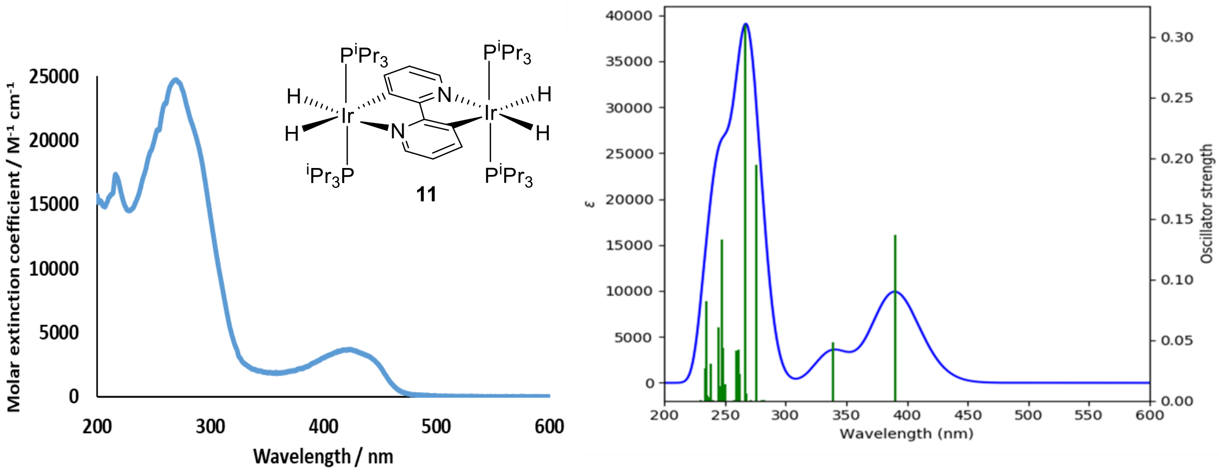

Figure S27. Observed UV-vis of complex **11** in 2-MeTHF (1.0 x 10^{-4} M) and calculated (B3LYP(GD3)//SDD(f)/6-31G**) in THF.

● **Analysis of Computed UV-vis Data for 3-11.**

Selected transitions for UV spectra, energies, oscillator strengths, and molecular orbital contributions to the transitions are given in Tables S1, S3, S5, S7, S9, S11, S13, S15, and S17. In order to facilitate the understanding of the electronic transitions, an analysis of the change in charge density for each different group in which the molecules have been divided with the GaussSum program is included in S2, S4, S6, S8, S10, S12, S14, S16, and S18.

Table S1. Selected transitions for the calculated UV-vis spectrum of complex 3 in THF.

No.	λ (nm)	Osc. strength	Symmetry	Major contributions	Minor contributions
**1**	498	0	Triplet	HOMO->LUMO (95%)	H-3->LUMO (3%)
**3**	444	0.0272	Singlet	HOMO->LUMO (91%)	H-1->LUMO (7%)
**4**	400	0.0582	Singlet	H-1->LUMO (91%)	HOMO->LUMO (7%)
**10**	335	0.0517	Singlet	H-1->L+1 (93%)	
**50**	232	0.1039	Singlet	H-7->LUMO (11%)<br>H-6->LUMO (47%)<br>HOMO->L+5 (11%)	H-4->L+1(2%), H-3->LUMO (2%),<br>H-3->L+1 (4%), H-3->L+3 (7%)

Table S2. Composition (%) of the selected transitions for the calculated UV-vis spectrum of complex 3.

No.	λ (nm)	Osc. strength	Symmetry	Osmium	Hydrides	Phosphine 1	Phosphine 2	Ligand
**1**	498	0	Triplet	58-->3 (-55)	0-->0 (0)	7-->1 (-6)	6-->1 (-5)	30-->95 (65)
**3**	444	0.0272	Singlet	60-->3 (-57)	0-->0 (0)	7-->1 (-6)	6-->1 (-5)	27-->95 (68)
**4**	400	0.0582	Singlet	73-->3 (-70)	0-->0 (0)	8-->1 (-7)	8-->1 (-7)	11-->95 (84)
**10**	335	0.0517	Singlet	74-->4 (-70)	0-->0 (0)	8-->1 (-7)	8-->1 (-7)	10-->95 (85)
**50**	232	0.0185	Singlet	20-->4 (-16)	15-->0 (-15)	21-->2 (-19)	19-->1 (-18)	25-->92 (67)

Table S3. Selected transitions for the calculated UV-vis spectrum of complex 4 in THF.

No.	λ (nm)	Osc. strength	Symmetry	Major contributions	Minor contributions
**1**	507	0	Triplet	HOMO->LUMO (95%)	H-3->LUMO (3%)
**3**	446	0.0327	Singlet	HOMO->LUMO (91%)	H-1->LUMO (7%)
**4**	399	0.0599	Singlet	H-1->LUMO (91%)	HOMO->LUMO (7%)
**10**	334	0.0525	Singlet	H-1->L+1 (93%)	
**48**	235	0.1747	Singlet	H-6->LUMO (56%), HOMO->L+5 (15%)	H-4->L+1 (5%), H-3->LUMO (2%), H-3->L+1 (6%), H-3->L+3 (8%)

Table S4. Composition (%) of the selected transitions for the calculated UV-vis spectrum of complex 4.

No.	λ (nm)	Osc. strength	Symmetry	Osmium	Hydrides	Phosphine 1	Phosphine 2	Ligand
**1**	507	0	Triplet	54-->3 (-51)	0-->0 (0)	6-->1 (-5)	6-->1 (-5)	34-->94 (60)
**3**	446	0.0327	Singlet	57-->3 (-54)	0-->0 (0)	6-->1 (-5)	6-->1 (-5)	30-->94 (64)
**4**	399,	0.0599	Singlet	73-->3 (-70)	0-->0 (0)	8-->1 (-7)	8-->1 (-7)	11-->94 (83)
**10**	334	0.0525	Singlet	74-->4 (-70)	0-->0 (0)	8-->1 (-7)	8-->1 (-7)	10-->95 (85)
**48**	235	0.1747	Singlet	18-->4 (-14)	0-->0 (0)	6-->1 (-5)	6-->1 (-5)	71-->94 (23)

Table S5. Selected transitions for the calculated UV-vis spectrum of complex 5 in THF.

No.	λ (nm)	Osc. strength	Symmetry	Major contributions	Minor contributions
1	504	0	Triplet	HOMO->LUMO (95%)	
3	444	0.0344	Singlet	HOMO->LUMO (88%)	H-1->LUMO (9%)
5	405	0.0457	Singlet	H-1->LUMO (89%)	HOMO->LUMO (9%)
11	344	0.0884	Singlet	H-1->L+1 (63%), HOMO->L+2 (29%)	H-1->L+2 (4%)
21	298	0.2545	Singlet	H-3->LUMO (17%), H-2->LUMO (75%)	H-1->L+2 (3%)
49	255	0.1933	Singlet	H-7->LUMO (21%), H-5->LUMO (54%)	H-6->LUMO (6%), H-2->L+1 (9%), H-2->L+2 (3%)

Table S6. Composition (%) of the selected transitions for the calculated UV-vis spectrum of complex 5.

No.	λ (nm)	Osc. strength	Symmetry	Osmium	Hydrides	Phosphine 1	Phosphine 2	Ligand
1	504	0	Triplet	52-->3 (-49)	0-->0 (0)	5-->1 (-4)	5-->1 (-4)	38-->96 (58)
3	444	0.0344	Singlet	53-->3 (-50)	0-->0 (0)	5-->1 (-4)	6-->1 (-5)	35-->96 (61)
5	405	0.0457	Singlet	71-->3 (-68)	0-->0 (0)	8-->1 (-7)	8-->1 (-7)	12-->96 (84)
11	344	0.0884	Singlet	67-->3 (-64)	0-->0 (0)	8-->1 (-7)	7-->1 (-6)	18-->95 (77)
21	298	0.2545	Singlet	20-->3 (-17)	5-->0 (-5)	2-->1 (-1)	4-->1 (-3)	69-->96 (27)
49	255	0.1933	Singlet	6-->3 (-3)	5-->0 (-5)	1-->1 (0)	2-->1 (-1)	86-->95 (9)

Table S7. Selected transitions for the calculated UV-vis spectrum of complex 6 in THF.

No.	λ (nm)	Osc. strength	Symmetry	Major contributions	Minor contributions
1	511	0	Triplet	HOMO->LUMO (96%)	H-4->LUMO (2%)
4	440	0.0966	Singlet	HOMO->LUMO (96%)	
7	394	0.0653	Singlet	H-2->LUMO (96%)	HOMO->LUMO (2%)
28	294	0.1481	Singlet	H-3->L+1 (85%)	H-2->L+3 (6%), H-1->L+1 (4%), HOMO->L+3 (4%)
37	282	0.2481	Singlet	H-4->LUMO (27%), H-2->L+3 (51%), HOMO->L+3 (15%)	
95	228	0.0471	Singlet	H-1->L+7 (96%)	

Table S8. Composition (%) of the selected transitions for the calculated UV-vis spectrum of complex 6.

No.	λ (nm)	Osc. strength	Symmetry	Osmium 1	Osmium 2	Ligand	Hydride 1	Hydride 2	Phosphine 1	Phosphine 2
1	511	0	Triplet	24-->2 (-22)	24-->2 (-22)	35-->93(58)	0-->0 (0)	0-->0 (0)	8-->1 (-7)	8-->1 (-7)
4	440	0.0966	Singlet	25-->2 (-23)	25-->2 (-23)	34-->93(59)	0-->0 (0)	0-->0 (0)	8-->1 (-7)	8-->1 (-7)
7	394	0.0653	Singlet	36-->2 (-34)	36-->2 (-34)	13-->93(80)	0-->0 (0)	0-->0 (0)	7-->1 (-6)	7-->1 (-6)
28	294	0.1481	Singlet	37-->4 (-33)	37-->4 (-33)	10-->86(76)	0-->0 (0)	0-->0 (0)	8-->2 (-6)	8-->2 (-6)
37	282	0.2481	Singlet	27-->2 (-25)	27-->2 (-25)	33-->92(59)	0-->0 (0)	0-->0 (0)	7-->2 (-5)	7-->2 (-5)
95	228	0.0471	Singlet	33-->2 (-31)	33-->2 (-31)	14-->92(78)	0-->0 (0)	0-->0 (0)	9-->2 (-7)	9-->2 (-7)

Table S9. Selected transitions for the calculated UV-vis spectrum of complex 7 in THF.

No.	λ (nm)	Osc. strength	Symmetry	Major contributions	Minor contributions
1	525	0	Triplet	HOMO->LUMO (96%)	H-4->LUMO (3%)
2	447	0.0949	Singlet	HOMO->LUMO (97%)	
7	396	0.0531	Singlet	H-2->LUMO (96%)	
28	294	0.1643	Singlet	H-3->L+1 (77%)	H-4->LUMO (3%), H-2->L+4 (4%), H-1->L+1 (3%), HOMO->L+4 (9%)
46	270	0.1805	Singlet	H-4->LUMO (22%), H-2->L+4 (65%)	H-3->L+1 (2%), H-3->L+4 (3%), HOMO->L+4 (3%)
97	226	0.0345	Singlet	H-1->L+7 (76%)	H-9->LUMO (8%), H-4->L+1 (6%), H-2->L+7 (2%)

Table S10. Composition (%) of the selected transitions for the calculated UV-vis spectrum of complex 7.

No.	λ (nm)	Osc. strength	Symmetry	Osmium 1	Osmium 2	Ligand	Hydride 1	Hydride 2	Phosphine 1	Phosphine 2
1	525	0	Triplet	23-->2 (-21)	25-->2 (-23)	37-->93(56)	0-->0 (0)	0-->0 (0)	7-->2 (-5)	8-->1 (-7)
2	447	0.0949	Singlet	23-->2 (-21)	25-->2 (-23)	36-->93(57)	0-->0 (0)	0-->0 (0)	7-->2 (-5)	9-->1 (-8)
7	396	0.0531	Singlet	61-->2 (-59)	13-->2 (-11)	12-->93(81)	0-->0 (0)	0-->0 (0)	12-->2 (-10)	3-->1 (-2)

28	294	0.1643	Singlet	18-->4 (-14)	52-->4 (-48)	14-->88(74)	0-->0 (0)	0-->0 (0)	4-->2 (-2)	11-->2 (-9)
46	270	0.1805	Singlet	45-->2 (-43)	15-->3 (-12)	26-->93(67)	0-->0 (0)	0-->0 (0)	9-->1 (-8)	4-->2 (-2)
97	226	0.0345	Singlet	31-->7 (-24)	28-->2 (-26)	24-->88(64)	0-->0 (0)	0-->0 (0)	9-->1 (-8)	8-->2 (-6)

Table S11. Selected transitions for the calculated UV-vis spectrum of complex 8 in THF.

No.	λ (nm)	Osc. strength	Symmetry	Major contributions	Minor contributions
1	551	0	Triplet	HOMO->LUMO (96%)	
2	481	0.0347	Singlet	HOMO->LUMO (95%)	
10	383	0.1737	Singlet	H-2->LUMO (50%), HOMO->L+1 (45%)	
19	329	0.3633	Singlet	H-2->L+1 (33%), H-1->L+1 (15%), HOMO->L+2 (42%)	H-3->L+1 (2%), HOMO->L+1 (2%)
48	278	0.0482	Singlet	HOMO->L+6 (59%)	H-4->L+1 (3%), H-3->L+2 (2%), H-2->L+3 (3%), H-2->L+4 (2%), H-1->L+3 (5%), H-1->L+4 (3%), HOMO->L+7 (4%)
94	241	0.077	Singlet	H-1->L+7 (71%)	H-10->LUMO (2%), H-5->L+1 (4%), H-5->L+2 (4%), H-4->L+2 (2%), H-3->L+6 (3%), H-2->L+6 (4%)

Table S12. Composition (%) of the selected transitions for the calculated UV-vis spectrum of complex 8.

No.	λ (nm)	Osc. strength	Symmetry	Osmium 1	Osmium 2	Ligand	Hydride 1	Hydride 2	Phosphine 1	Phosphine 2
1	551	0	Triplet	22-->3 (-19)	22-->2 (-20)	47-->92(45)	0-->0 (0)	0-->0 (0)	3-->2 (-1)	7-->1 (-6)
2	481	0.0347	Singlet	22-->3 (-19)	22-->2 (-20)	47-->92(45)	0-->0 (0)	0-->0 (0)	3-->2 (-1)	7-->1 (-6)
10	383	0.1737	Singlet	32-->2 (-30)	24-->3 (-21)	29-->91(62)	0-->0 (0)	0-->0 (0)	9-->2 (-7)	6-->2 (-4)

19	329	0.3633	Singlet	31-->3 (-28)	24-->3 (-21)	31-->90(59)	0-->0 (0)	0-->0 (0)	8-->2 (-6)	6-->2 (-4)
48	278	0.0482	Singlet	24-->11 (13)	22-->8 (-14)	42-->77(35)	0-->0 (0)	0-->0 (0)	4-->3 (-1)	7-->1 (-6)
94	241	0.077	Singlet	34-->2 (-32)	19-->3 (-16)	30-->92(62)	0-->0 (0)	0-->0 (0)	11-->2 (-9)	7-->2 (-5)

Table S13. Selected transitions for the calculated UV-vis spectrum of complex 9 in THF.

No.	λ (nm)	Osc. strength	Symmetry	Major contributions	Minor contributions
1	445	0	Triplet	HOMO->LUMO (82%)	H-4->LUMO (9%), H-3->LUMO (3%)
4	381	0.0657	Singlet	HOMO->LUMO (94%)	H-3->LUMO (2%)
10	343	0.0368	Singlet	H-3->LUMO (90%)	H-2->LUMO (6%)
22	274	0.2307	Singlet	H-4->LUMO (78%)	H-5->LUMO (4%), H-3->L+1 (8%), HOMO->L+3 (3%)

Table S14. Composition (%) of the selected transitions for the calculated UV-vis spectrum of complex 9.

No.	λ (nm)	Osc. strength	Symmetry	Osmium	Hydrides	Phosphine 1	Phosphine 2	Ligand
1	445	0	Triplet	42-->2 (-40)	0-->0 (0)	7-->1 (-6)	7-->1 (-6)	45-->96 (51)
4	381	0.0657	Singlet	44-->2 (-42)	0-->0 (0)	7-->1 (-6)	7-->1 (-6)	42-->96 (54)
10	343	0.0368	Singlet	59-->2 (-57)	4-->0 (-4)	10-->1 (-9)	9-->1 (-8)	18-->96 (78)
22	274	0.2307	Singlet	23-->3 (-20)	0-->0 (0)	5-->1 (-4)	6-->1 (-5)	65-->96 (31)

Table S15. Selected transitions for the calculated UV-vis spectrum of complex 10 in THF.

No.	λ (nm)	Osc. strength	Symmetry	Major contributions	Minor contributions
1	492	0	Triplet	HOMO->LUMO (94%)	H-7->LUMO (2%)
3	426	0.0798	Singlet	HOMO->LUMO (95%)	H-1->LUMO (3%)
6	390	0.0496	Singlet	H-1->LUMO (95%)	HOMO->LUMO (3%)
30	285	0.175	Singlet	H-2->L+1 (88%)	H-7->LUMO (5%)

71	247	0.0776	Singlet	H-4->L+3 (89%)	H-7->L+1 (2%)

Table S16. Composition (%) of the selected transitions for the calculated UV-vis spectrum of complex 10.

No.	λ (nm)	Osc. strength	Symmetry	Osmium	Iridium	Ligand	Hydride Os	Hydride Ir	Phosphine Os	Phosphine Ir
1	492	0	Triplet	44-->2 (-42)	5-->2 (-3)	33-->94(61)	0-->0 (0)	0-->0 (0)	14-->2 (-12)	3-->1 (-2)
3	426	0.0798	Singlet	46-->2 (-44)	5-->2 (-3)	32-->94(62)	0-->0 (0)	0-->0 (0)	14-->2 (-12)	3-->1 (-2)
6	390	0.0496	Singlet	71-->2 (-69)	2-->2 (0)	12-->94(82)	0-->0 (0)	0-->0 (0)	13-->2 (-11)	2-->1 (-1)
30	285	0.175	Singlet	15-->5 (-10)	37-->4 (-33)	20-->87(67)	0-->0 (0)	0-->0 (0)	6-->2 (-4)	21-->2 (-19)
71	247	0.0776	Singlet	1-->2 (1)	58-->2 (-56)	26-->92(66)	0-->0 (0)	0-->0 (0)	1-->2 (1)	14-->2 (-12)

Table S17. Selected transitions for the calculated UV-vis spectrum of complex 11 in THF.

No.	λ (nm)	Osc. strength	Symmetry	Major contributions	Minor contributions
1	461	0	Triplet	HOMO->LUMO (88%)	H-8->LUMO (3%), H-4->LUMO (7%)
3	390	0.1364	Singlet	HOMO->LUMO (98%)	
12	338	0.0484	Singlet	H-4->LUMO (97%)	
31	276	0.1947	Singlet	H-8->LUMO (27%), H-1->L+1 (63%)	HOMO->L+3 (6%)
42	266	0.31	Singlet	H-8->LUMO (40%), HOMO->L+3 (38%)	H-5->L+1 (9%), H-1->L+1 (5%)
72	247	0.1332	Singlet	H-4->L+3 (59%), H-3->L+2 (14%)	H-8->LUMO (4%), H-5->L+1 (6%), H-2->L+4 (6%), H-2->L+5 (3%)

Table S18. Composition (%) of the selected transitions for the calculated UV-vis spectrum of complex 11.

No.	λ (nm)	Osc. strength	Symmetry	Iridium 1	Iridium 2	Ligand	Hydride 1	Hydride 2	Phosphine 1	Phosphine 2
1	461	0	Triplet	19-->2 (-17)	19-->2 (-17)	41-->94(53)	0-->0 (0)	0-->0 (0)	10-->1 (-9)	10-->1 (-9)
3	390	0.1364	Singlet	19-->2 (-17)	19-->2 (-17)	42-->94(52)	0-->0 (0)	0-->0 (0)	10-->1 (-9)	10-->1 (-9)
12	338	0.0484	Singlet	24-->3 (-21)	24-->3 (-21)	24-->89(65)	0-->0 (0)	0-->0 (0)	14-->2 (-12)	14-->2 (-12)
31	276	0.1947	Singlet	20-->2 (-18)	20-->2 (-18)	40-->92(52)	0-->0 (0)	0-->0 (0)	10-->2 (-8)	10-->2 (-8)

42	266	0.31	Singlet	30-->13(-17)	30-->13(-17)	25-->70(45)	1-->0 (-1)	1-->0 (-1)	6-->2 (-4)	6-->2 (-4)

● **Theoretical Analysis of Molecular Orbitals of Complexes 3-11.**

Table S19. Composition of the frontier orbitals of 3.

MO	eV	Osmium	Hydrides	Phosphine1	Phosphine2	Ligand
**L+9**	1.81	42	14	22	21	1
**L+8**	1.63	95	0	1	4	0
**L+7**	1.55	59	4	19	15	2
**L+6**	1.21	93	2	6	3	-3
**L+5**	1.05	4	0	1	1	95
**L+4**	0.73	79	5	4	4	9
**L+3**	0.25	14	0	3	1	83
**L+2**	0.19	82	0	3	4	11
**L+1**	-0.68	4	0	1	1	95
**LUMO**	-1.27	3	0	1	1	95
**HOMO**	-4.81	59	0	7	6	28
**H-1**	-5.09	74	0	8	8	10
**H-2**	-5.98	13	18	2	2	65
**H-3**	-6.22	14	0	5	6	75
**H-4**	-6.37	16	0	37	36	11
**H-5**	-6.97	13	19	3	4	60
**H-6**	-7.20	8	0	5	4	82
**H-7**	-7.41	22	18	23	21	16
**H-8**	-7.57	9	4	44	17	25
**H-9**	-7.61	24	12	20	6	37

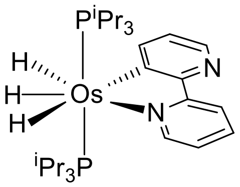

3

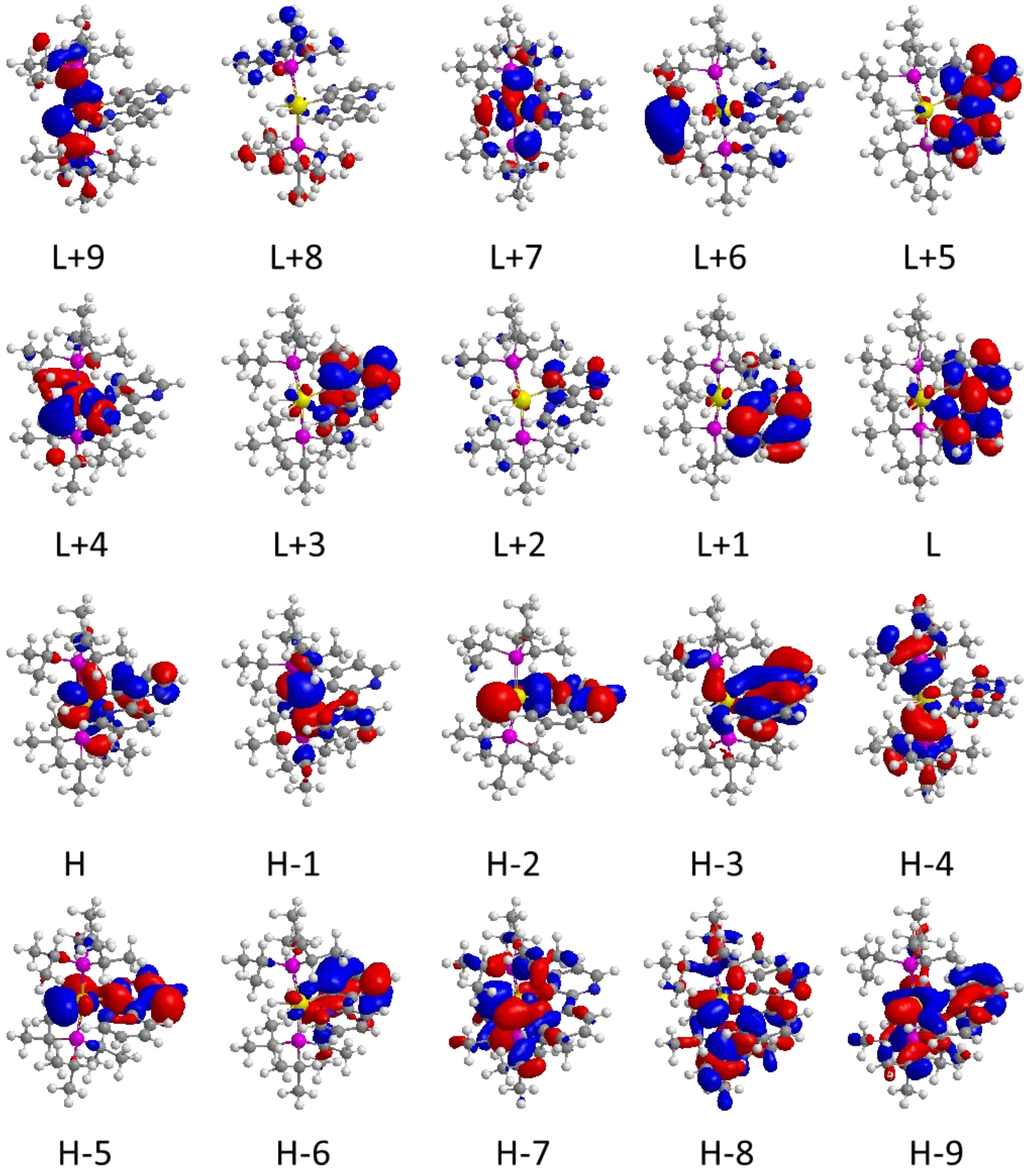

Figure S28. Frontier molecular orbitals of complex **3** (isovalue 0.03 au).

Table S20. Composition of the frontier orbitals of 4.

MO	eV	Osmium	Hydrides	Phosphine1	Phosphine2	Ligand
**L+9**	1.81	46	14	19	19	2
**L+8**	1.62	95	0	4	1	0
**L+7**	1.56	58	6	16	16	3
**L+6**	1.20	93	2	5	3	-2
**L+5**	1.04	3	0	1	1	95
**L+4**	0.72	79	5	3	4	10
**L+3**	0.34	5	0	2	1	92
**L+2**	0.19	91	0	4	4	2
**L+1**	-0.65	4	0	1	1	95
**LUMO**	-1.24	3	0	1	1	94
**HOMO**	-4.75	56	0	6	6	32
**H-1**	-5.06	74	0	8	8	10
**H-2**	-5.97	13	17	2	1	67
**H-3**	-6.13	16	0	3	5	76
**H-4**	-6.37	18	0	38	38	6
**H-5**	-6.92	12	19	3	4	62
**H-6**	-7.10	8	0	4	3	85
**H-7**	-7.40	20	18	23	21	18
**H-8**	-7.51	6	3	25	11	55
**H-9**	-7.59	16	7	35	6	35

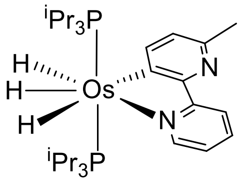

4

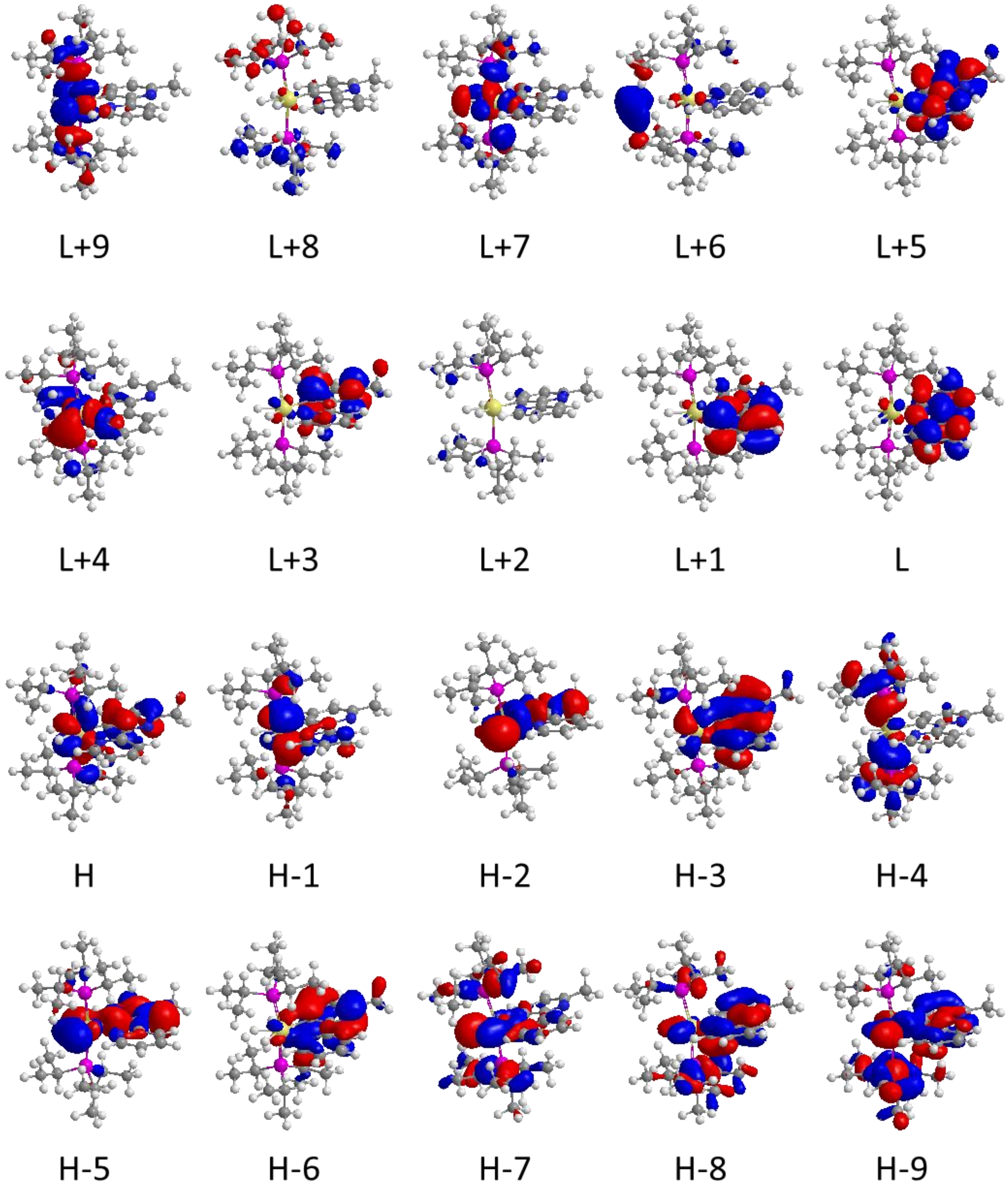

Figure S29. Frontier molecular orbitals of complex **4** (isovalue 0.03 au).

Table S21. Composition of the frontier orbitals of 5.

MO	eV	Osmium	Hydrides	Phosphine1	Phosphine2	Ligand
**L+9**	1.54	59	3	-2	11	30
**L+8**	1.40	30	0	0	3	67
**L+7**	1.17	90	3	5	5	-1
**L+6**	0.72	19	1	1	2	77
**L+5**	0.68	63	4	2	3	28
**L+4**	0.19	77	0	4	4	15
**L+3**	0.10	14	0	1	0	85
**L+2**	-0.56	2	0	1	0	97
**L+1**	-0.76	4	0	1	1	94
**LUMO**	-1.29	3	0	1	1	96
**HOMO**	-4.78	52	0	5	5	38
**H-1**	-5.07	73	0	9	8	9
**H-2**	-6.03	20	3	1	5	71
**H-3**	-6.09	14	14	3	1	69
**H-4**	-6.41	19	0	38	39	3
**H-5**	-6.64	1	0	0	0	98
**H-6**	-6.72	0	0	0	0	99
**H-7**	-7.01	12	19	4	4	61
**H-8**	-7.40	17	17	27	22	17
**H-9**	-7.43	4	0	7	4	85

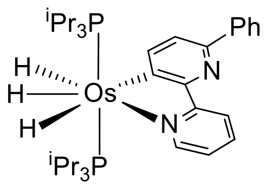

5

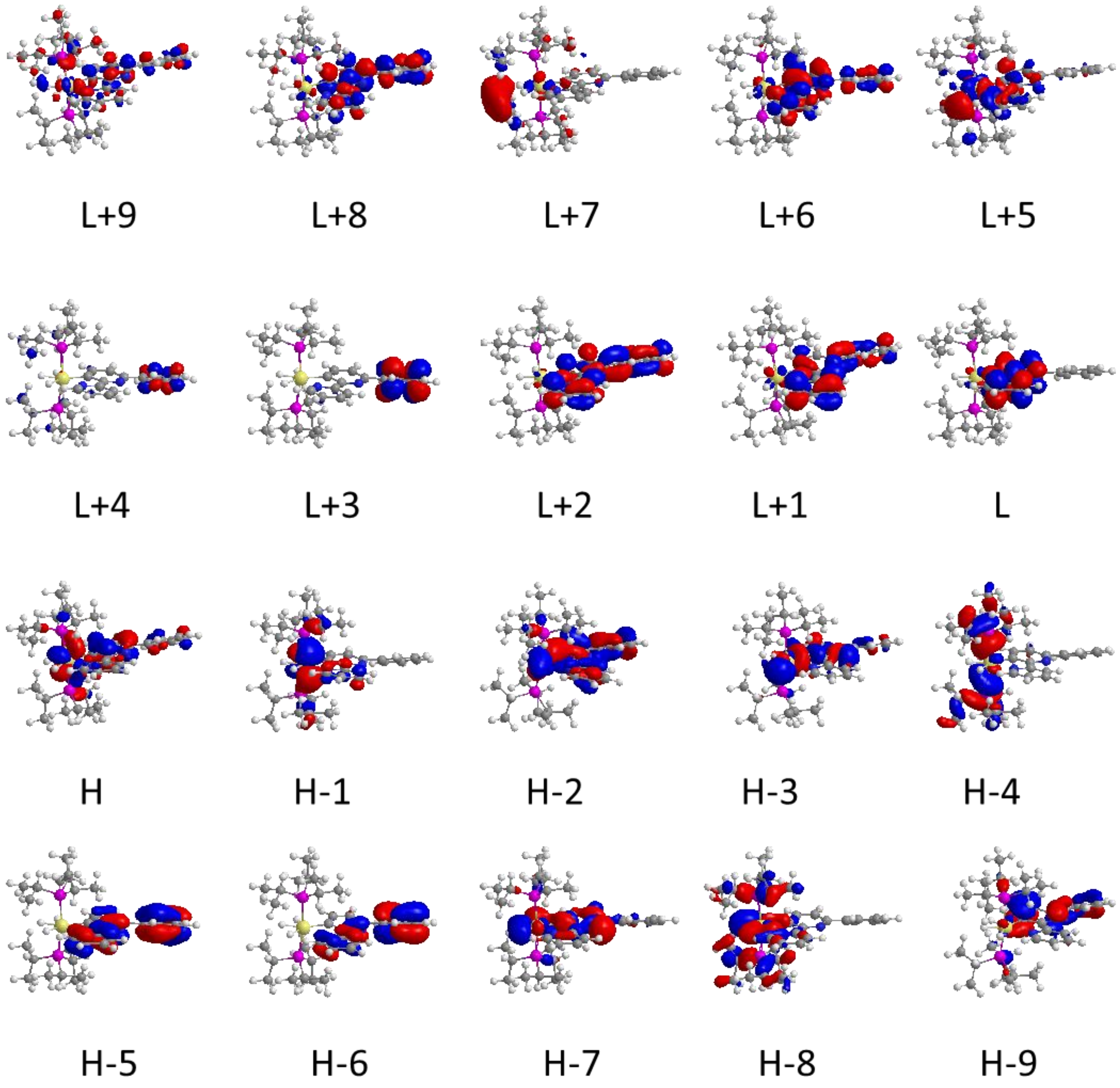

Figure S30. Frontier molecular orbitals of complex **5** (isovalue 0.03 au).

Table S22. Composition of the frontier orbitals of 6.

MO	eV	Osmium 1	Osmium 2	Ligand	Hydrides 1	Hydrides 2	Phosphine 1	Phosphine 2
**L+9**	1.53	32	32	3	3	3	14	14
**L+8**	1.47	35	35	0	1	1	14	14
**L+7**	1.26	2	2	92	0	0	2	2
**L+6**	0.83	44	44	7	1	1	2	2
**L+5**	0.47	40	40	13	2	2	1	1
**L+4**	0.29	47	47	2	0	0	3	3
**L+3**	0.20	2	2	92	0	0	2	2
**L+2**	0.18	46	46	1	0	0	3	3
**L+1**	-0.09	5	5	86	0	0	2	2
**LUMO**	-1.11	2	2	93	0	0	1	1
**HOMO**	-4.58	25	25	34	0	0	8	8
**H-1**	-4.87	33	33	14	0	0	9	9
**H-2**	-5.00	37	37	13	0	0	7	7
**H-3**	-5.04	38	38	9	0	0	8	8
**H-4**	-6.10	9	9	71	0	0	5	5
**H-5**	-6.23	9	9	48	14	14	3	3
**H-6**	-6.25	9	9	53	12	12	3	3
**H-7**	-6.43	11	11	0	0	0	39	39
**H-8**	-6.44	12	12	2	0	0	37	37
**H-9**	-7.24	11	11	26	13	13	13	13

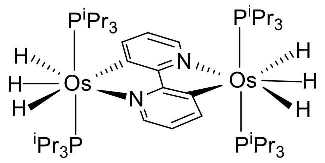

6

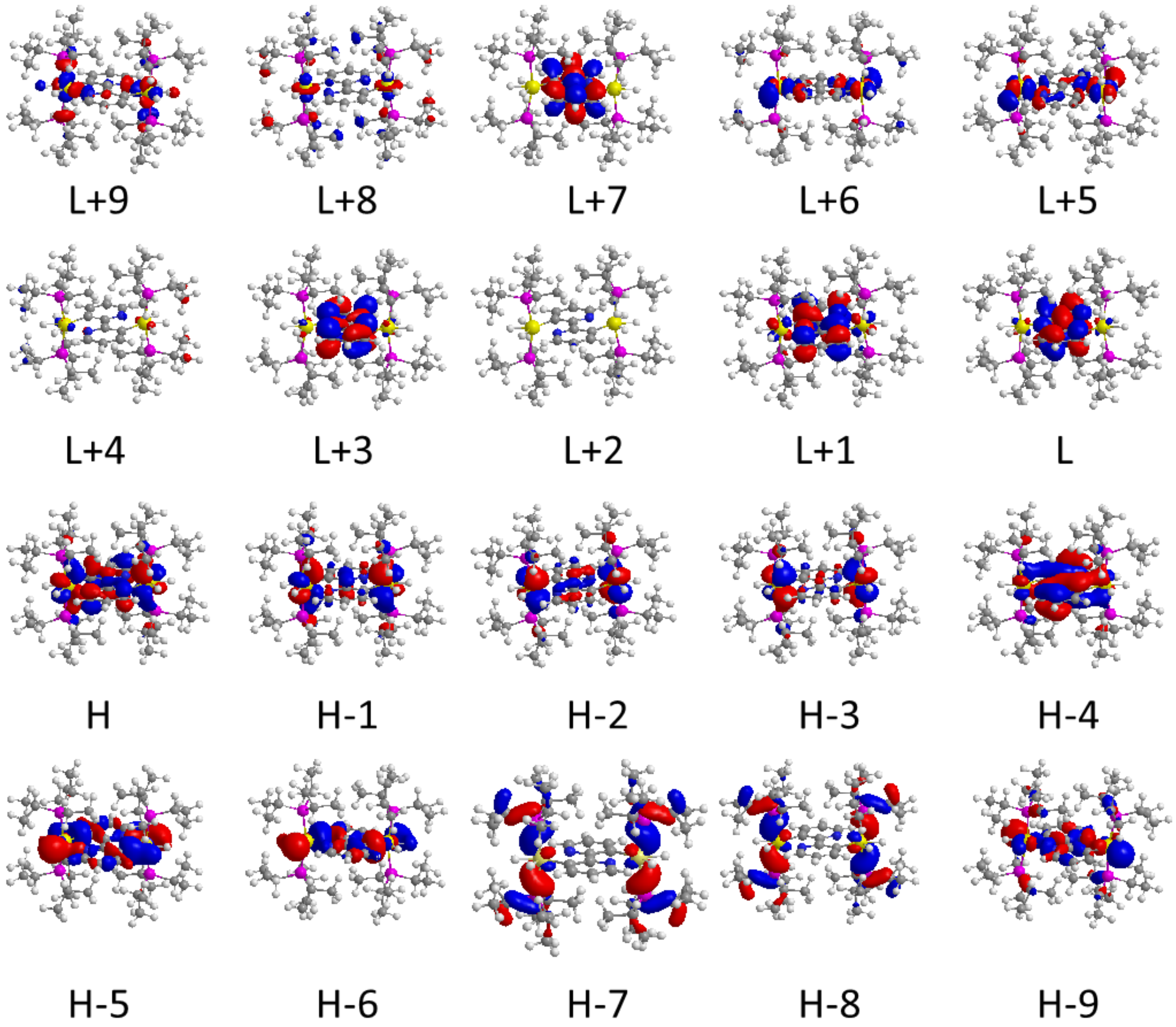

Figure S31. Frontier molecular orbitals of complex **6** (isovalue 0.03 au).

Table S23. Composition of the frontier orbitals of 7.

MO	eV	Osmium 1	Osmium 2	Ligand	Hydrides 1	Hydrides 2	Phosphine 1	Phosphine 2
**L+9**	1.50	32	36	0	1	2	15	14
**L+8**	1.43	57	15	2	3	0	17	6
**L+7**	1.31	8	2	88	0	0	0	2
**L+6**	0.97	67	21	5	1	0	5	1
**L+5**	0.59	19	63	12	1	2	1	2
**L+4**	0.31	2	3	93	0	0	1	2
**L+3**	0.27	32	60	2	0	0	2	3
**L+2**	0.18	58	34	2	0	0	5	2
**L+1**	-0.05	4	4	87	0	0	2	3
**LUMO**	-1.08	2	2	93	0	0	2	1
**HOMO**	-4.50	23	25	36	0	0	7	9
**H-1**	-4.84	34	32	15	0	0	9	9
**H-2**	-4.95	61	13	12	0	0	12	3
**H-3**	-4.99	15	60	10	0	0	3	12
**H-4**	-6.04	10	11	70	0	0	3	5
**H-5**	-6.13	14	5	54	18	5	4	1
**H-6**	-6.20	4	14	48	7	21	1	4
**H-7**	-6.39	22	0	0	0	0	78	0
**H-8**	-6.42	0	23	1	0	0	0	76
**H-9**	-7.12	3	4	80	0	0	9	4

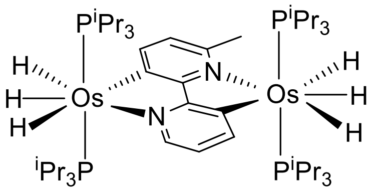

7

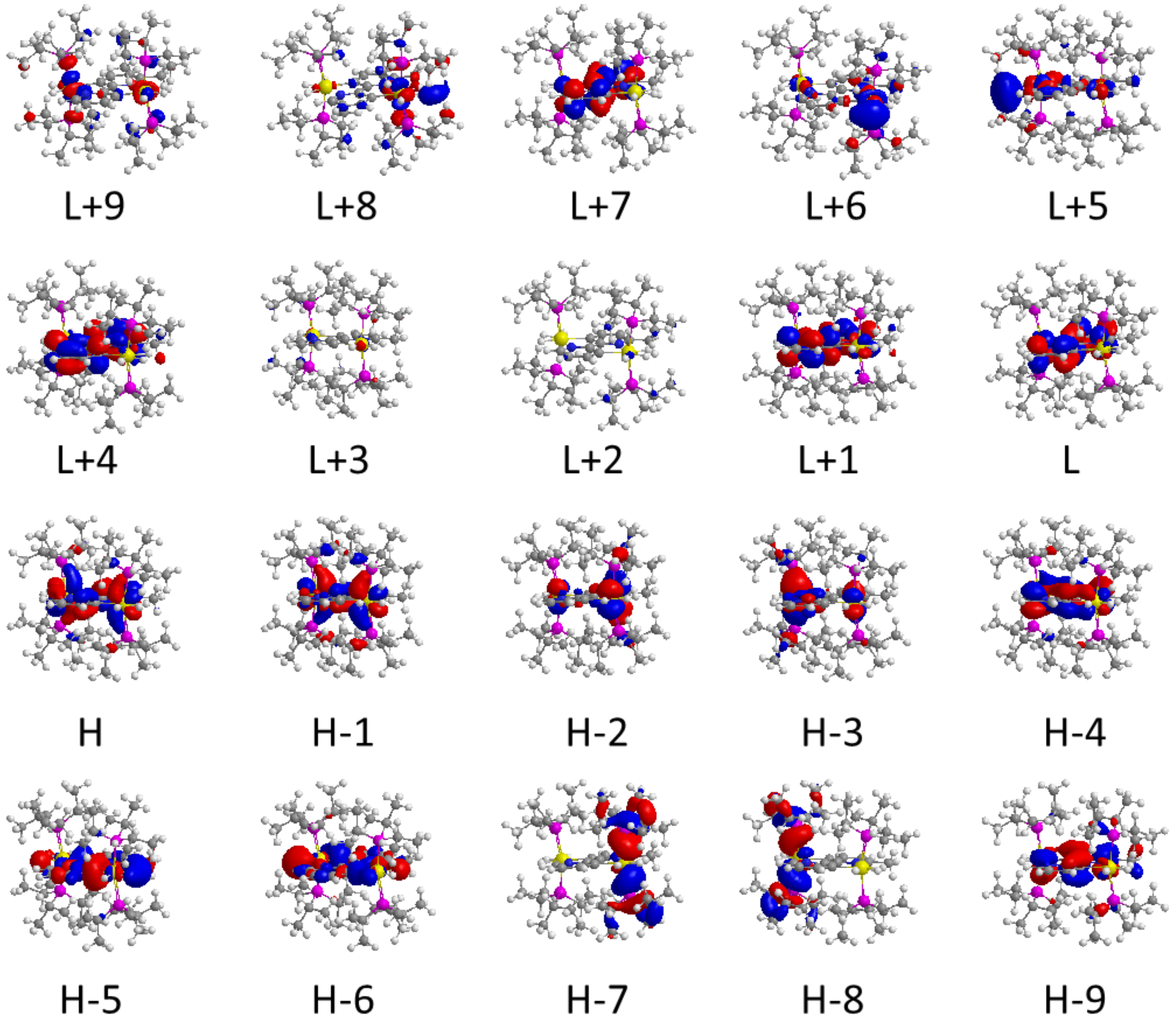

Figure S32. Frontier molecular orbitals of complex **7** (isovalue 0.03 au).

Table S24. Composition of the frontier orbitals of 8.

MO	eV	Osmium 1	Osmium 2	Ligand	Hydrides 1	Hydrides 2	Phosphine 1	Phosphine 2
**L+9**	1.36	62	5	13	7	0	10	3
**L+8**	1.12	68	11	7	3	0	9	2
**L+7**	0.90	2	3	92	0	0	2	2
**L+6**	0.67	5	0	91	0	0	3	0
**L+5**	0.57	12	69	12	1	3	1	3
**L+4**	0.28	39	52	4	0	0	2	3
**L+3**	0.20	48	44	3	0	0	3	3
**L+2**	-0.04	4	2	90	0	0	2	1
**L+1**	-0.59	2	4	91	0	0	2	2
**LUMO**	-1.09	3	2	92	0	0	2	1
**HOMO**	-4.35	22	22	47	0	0	3	7
**H-1**	-4.88	38	19	26	0	0	9	7
**H-2**	-4.98	41	27	13	0	0	14	5
**H-3**	-5.03	12	61	10	0	0	4	13
**H-4**	-5.89	6	13	74	0	0	3	4
**H-5**	-6.05	10	2	67	0	0	21	1
**H-6**	-6.13	13	1	72	7	1	5	0
**H-7**	-6.26	1	17	50	2	24	0	5
**H-8**	-6.43	2	20	8	0	0	2	68
**H-9**	-6.50	12	3	54	0	0	21	9

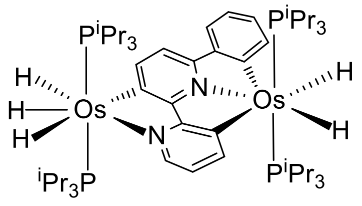

8

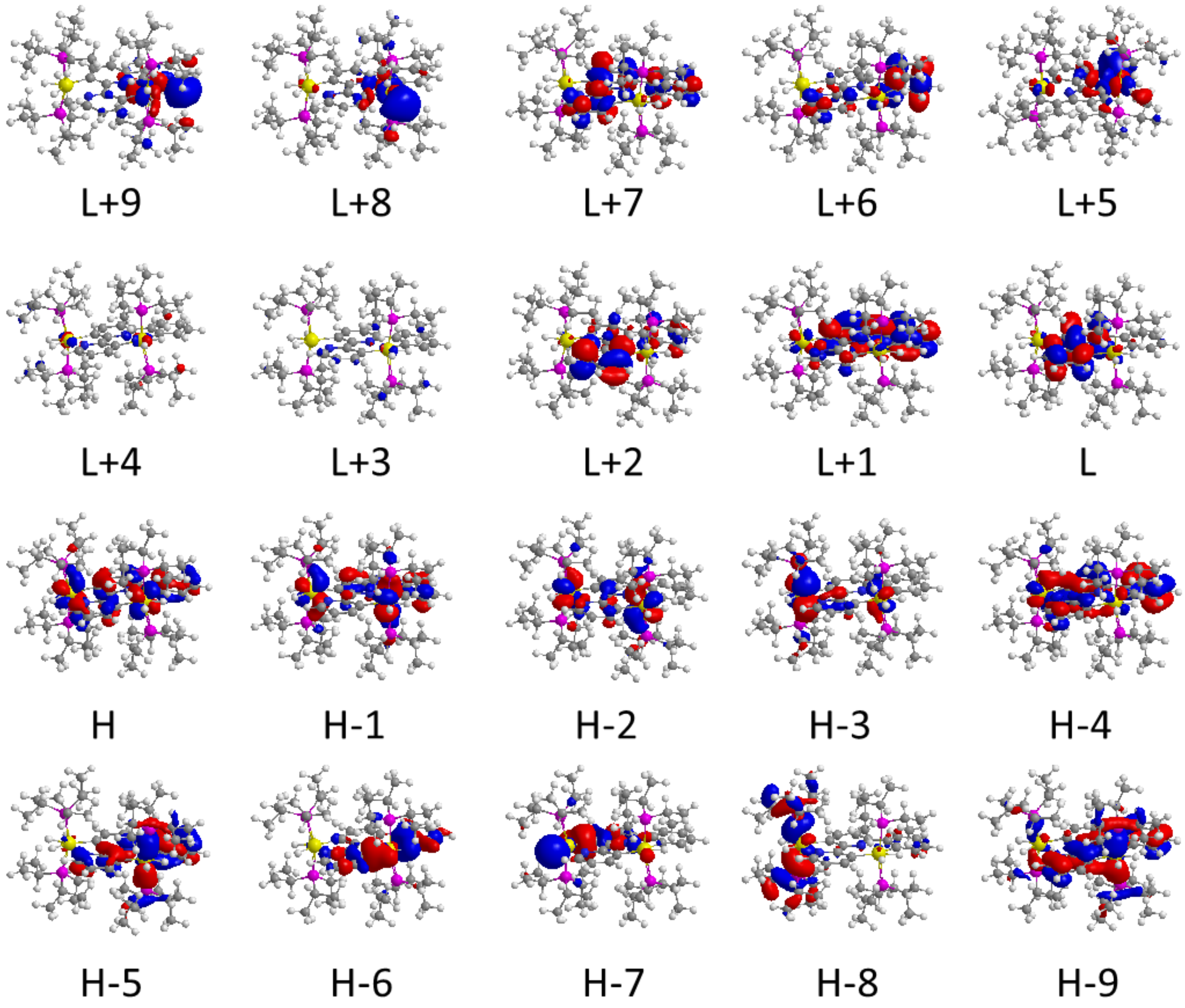

Figure S33. Frontier molecular orbitals of complex **8** (isovalue 0.03 au).

Table S25. Composition of the frontier orbitals of 9.

MO	eV	Osmium	Hydrides	Phosphine1	Phosphine2	Ligand
**L+9**	1.83	36	3	36	22	2
**L+8**	1.69	90	0	5	5	0
**L+7**	1.45	71	4	15	16	-5
**L+6**	1.26	76	1	13	9	1
**L+5**	1.03	5	0	1	1	93
**L+4**	0.82	79	2	6	7	7
**L+3**	0.27	11	0	2	1	86
**L+2**	0.22	85	0	3	4	9
**L+1**	-0.68	4	0	0	0	95
**LUMO**	-1.25	2	0	1	1	96
**HOMO**	-5.20	44	0	7	7	42
**H-1**	-5.44	86	3	1	1	9
**H-2**	-5.58	25	20	3	4	48
**H-3**	-5.62	62	3	10	9	16
**H-4**	-6.33	18	0	3	4	74
**H-5**	-6.53	25	0	37	36	1
**H-6**	-6.97	10	9	2	2	77
**H-7**	-7.10	18	31	9	10	33
**H-8**	-7.22	11	0	4	7	77
**H-9**	-7.62	1	0	28	7	64

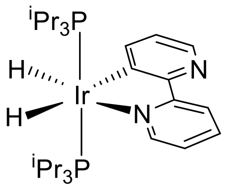

9

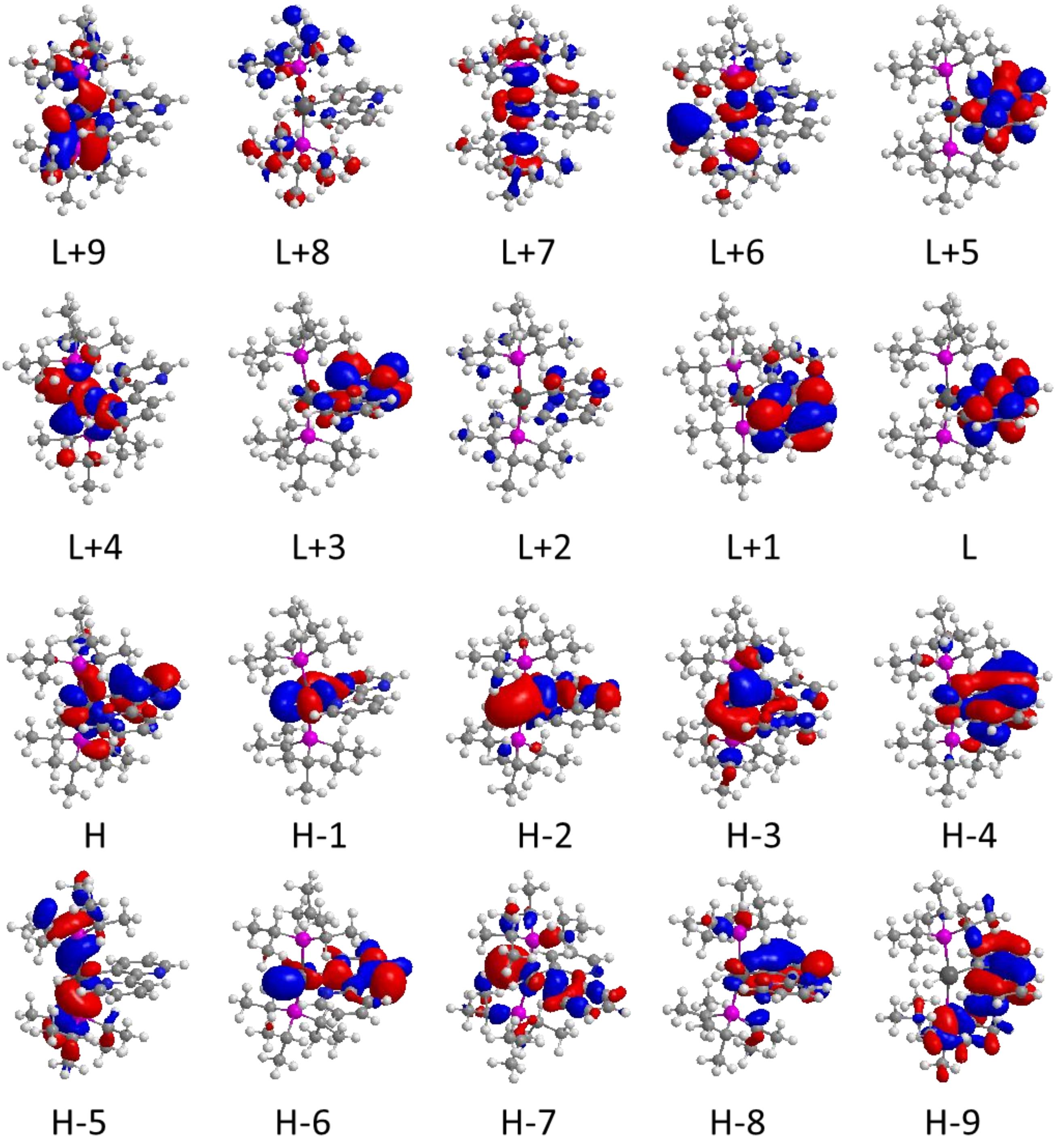

Figure S34. Frontier molecular orbitals of complex **9** (isovalue 0.03 au).

Table S26. Composition of the frontier orbitals of 10.

MO	eV	Osmium	Iridium	Ligand	Hydrides Os	Hydrides Ir	Phosphine Os	Phosphine Ir
**L+9**	1.53	51	14	0	3	1	20	11
**L+8**	1.36	4	43	2	0	8	2	39
**L+7**	1.27	2	2	92	0	0	2	2
**L+6**	0.88	34	54	6	1	0	2	4
**L+5**	0.53	50	32	12	2	0	1	2
**L+4**	0.31	33	59	1	0	0	2	4
**L+3**	0.24	2	2	92	0	0	2	2
**L+2**	0.20	59	33	1	0	0	4	3
**L+1**	-0.05	5	4	86	0	0	3	2
**LUMO**	-1.07	2	2	94	0	0	2	1
**HOMO**	-4.65	45	5	33	0	0	14	3
**H-1**	-5.00	72	2	12	0	0	13	2
**H-2**	-5.10	16	38	18	0	0	6	22
**H-3**	-5.34	0	77	13	0	6	0	4
**H-4**	-5.49	1	59	25	0	0	1	14
**H-5**	-5.92	1	30	42	1	21	0	5
**H-6**	-6.18	16	2	50	23	4	4	1
**H-7**	-6.18	8	21	56	0	0	5	9
**H-8**	-6.41	23	0	1	0	0	75	0
**H-9**	-6.73	0	27	2	0	0	0	70

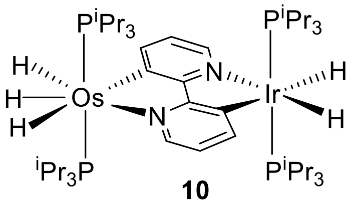

10

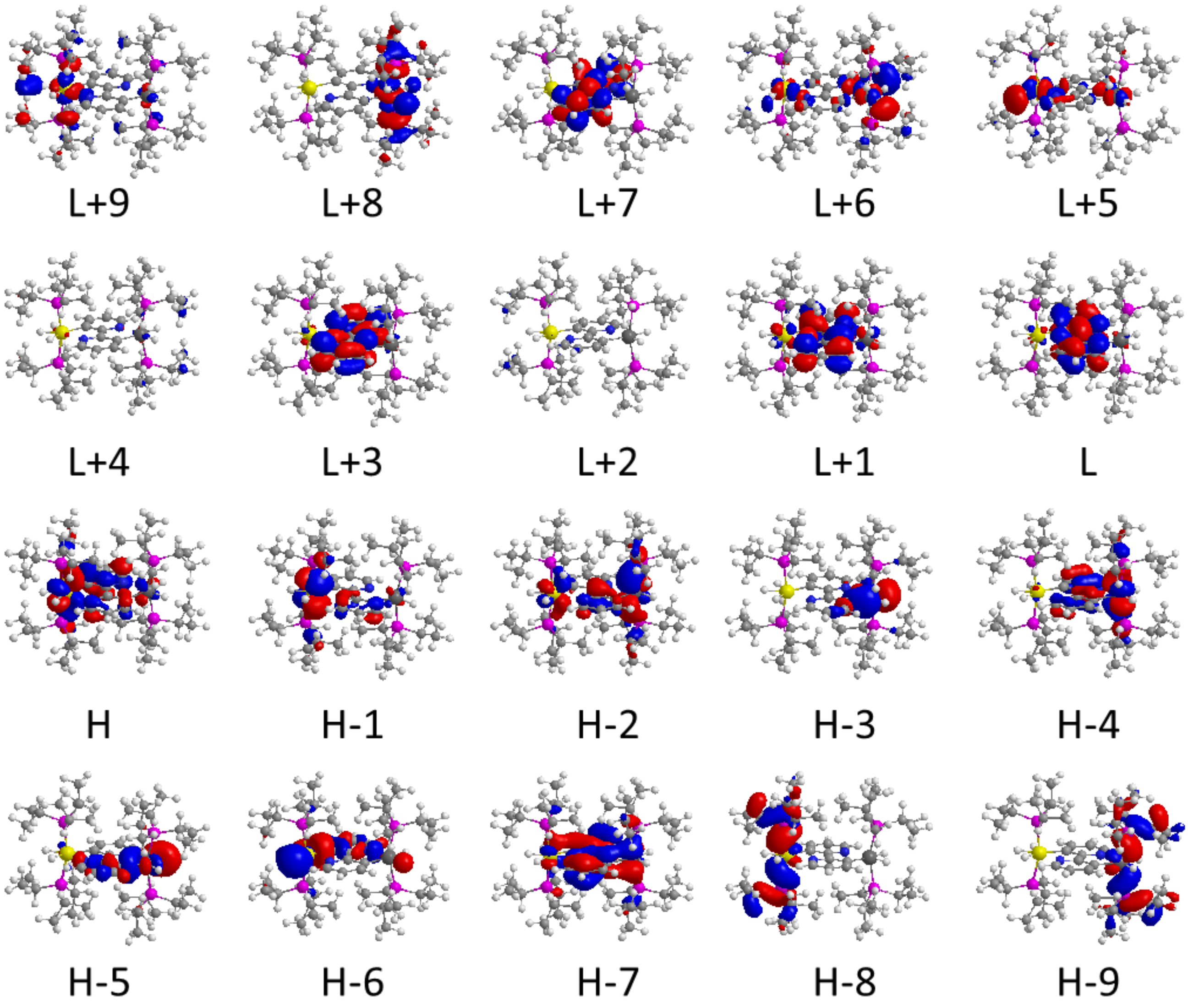

Figure S35. Frontier molecular orbitals of complex **10** (isovalue 0.03 au).

Table S27. Composition of the frontier orbitals of 11.

MO	eV	Iridium 148	Iridium 147	Ligand	Hydrides 148	Hydrides 147	Phosphine 148	Phosphine 147
**L+9**	1.40	23	23	2	5	5	22	22
**L+8**	1.35	24	24	3	4	4	21	21
**L+7**	1.28	2	2	92	0	0	2	2
**L+6**	0.92	44	44	6	0	0	3	3
**L+5**	0.60	42	42	11	0	0	2	2
**L+4**	0.33	46	46	1	0	0	3	3
**L+3**	0.28	2	2	92	0	0	2	2
**L+2**	0.21	46	46	2	0	0	4	4
**L+1**	-0.02	4	4	87	0	0	2	2
**LUMO**	-1.03	2	2	94	0	0	1	1
**HOMO**	-4.85	19	19	42	0	0	10	10
**H-1**	-5.21	27	27	14	0	0	16	16
**H-2**	-5.27	36	36	15	4	4	2	2
**H-3**	-5.36	41	41	9	2	2	2	2
**H-4**	-5.43	27	27	29	0	0	8	8
**H-5**	-5.54	35	35	16	0	0	7	7
**H-6**	-5.84	14	14	43	12	12	3	3
**H-7**	-5.88	16	16	43	10	10	2	2
**H-8**	-6.25	18	18	46	0	0	9	9
**H-9**	-6.69	14	14	1	0	0	36	36

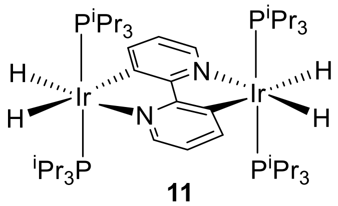

11

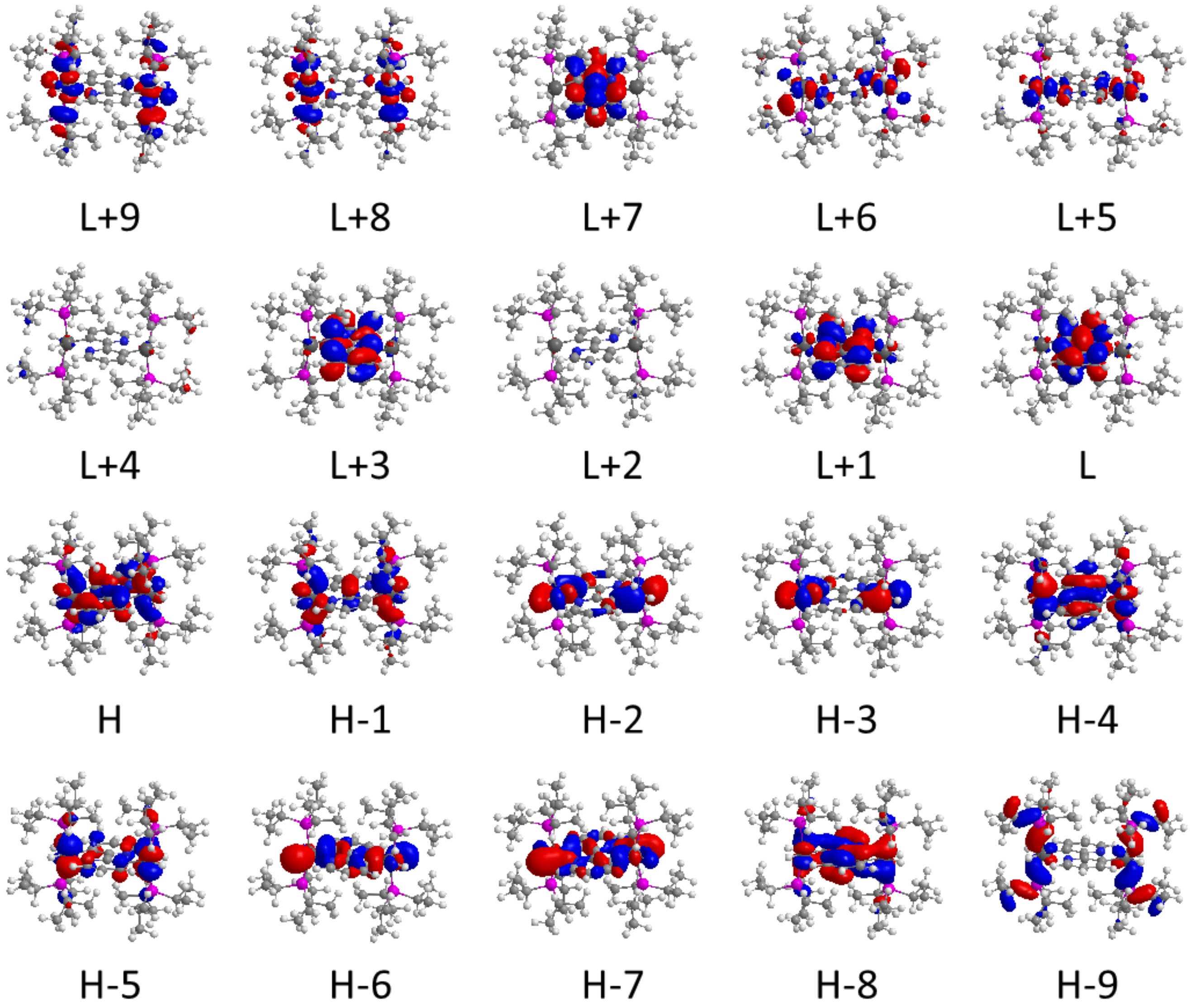

Figure S36. Frontier molecular orbitals of complex **11** (isovalue 0.03 au).

● **Normalized Excitation and Emission and Spectra of Complexes 3-8**.

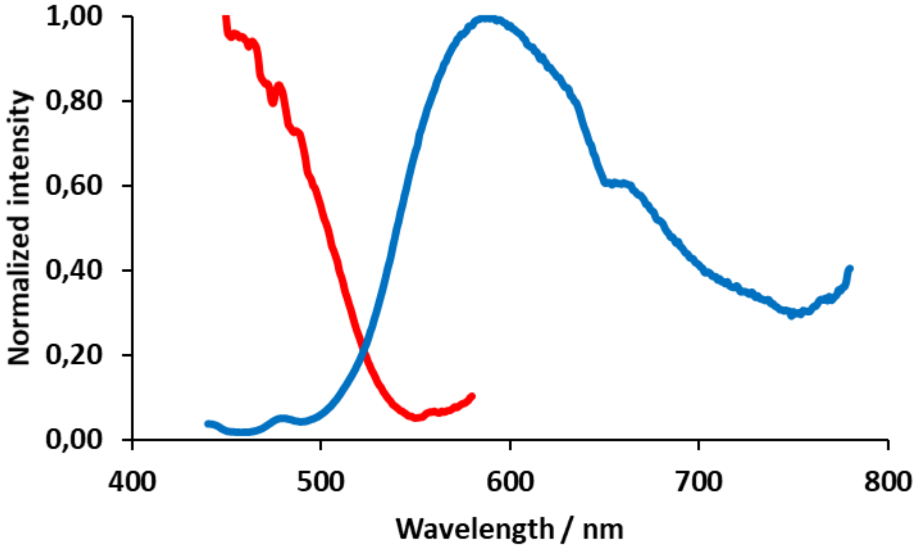

Figure S37. Normalized emission spectrum of complex **3** in PMMA film (5 wt%) at 298 K.

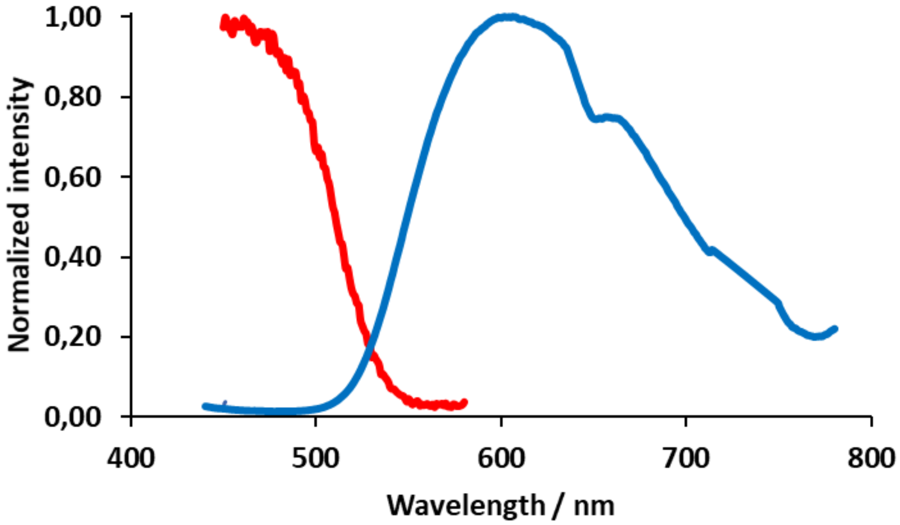

Figure S38. Normalized emission spectrum of complex **3** in a 1 x 10^{-5} M solution in 2-MeTHF at 298 K.

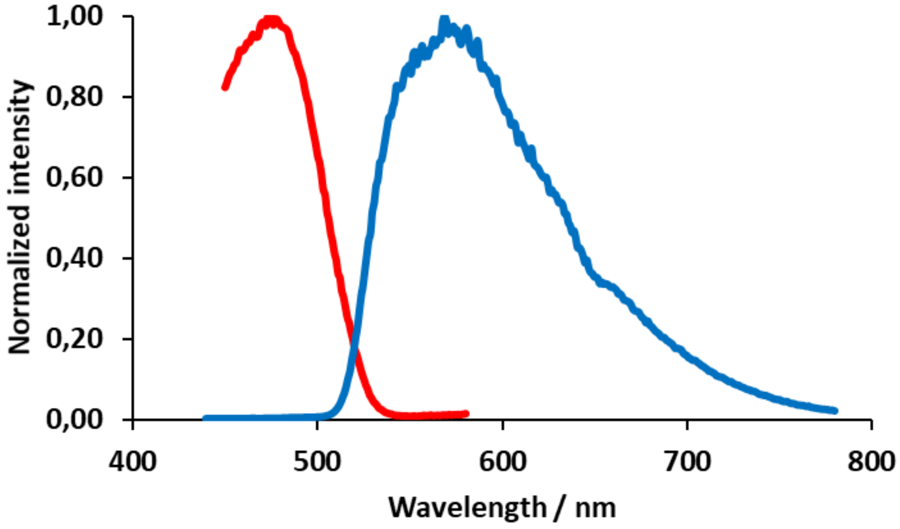

.

Figure S39. Normalized emission spectrum of complex **3** in a 1 x 10^{-5} M solution in 2-MeTHF at 77 K.

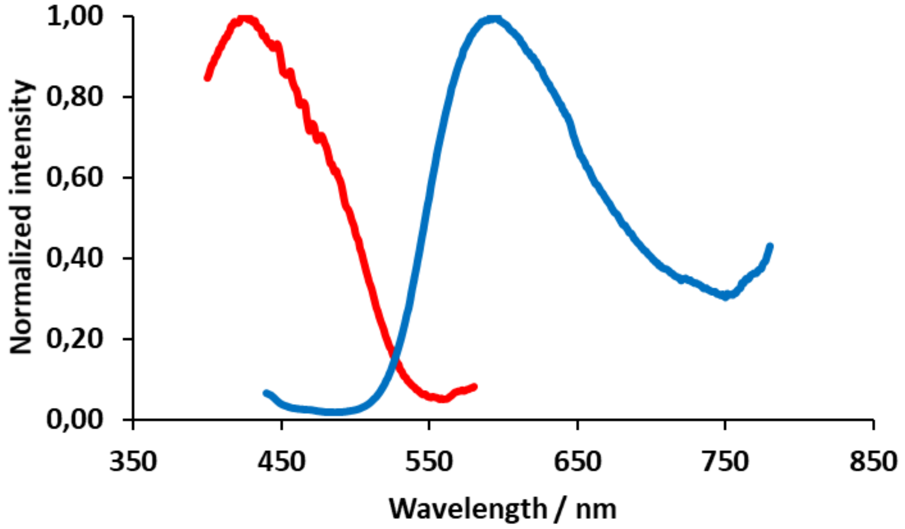

Figure S40. Normalized emission spectrum of complex **4** in PMMA film (5 wt%) at 298 K.

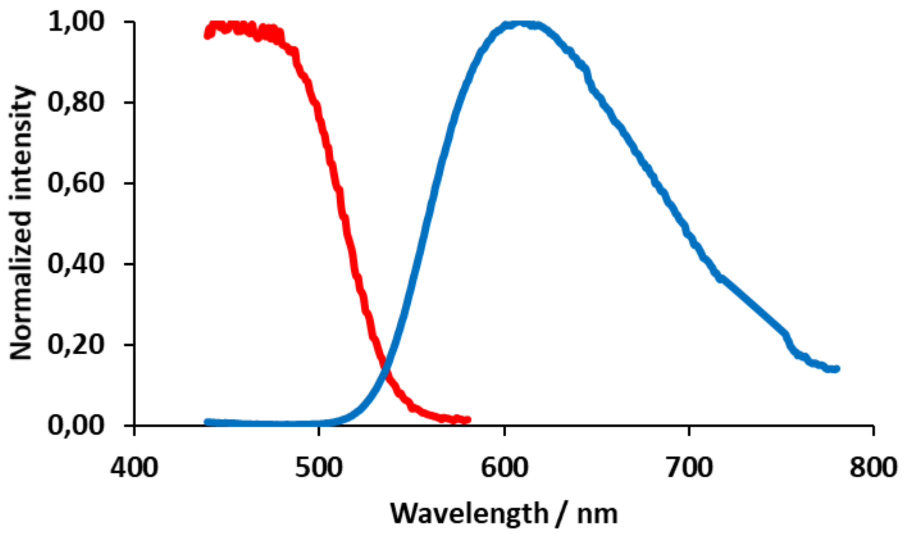

Figure S41. Normalized emission spectrum of complex **4** in a 1 x 10^{-5} M solution in 2-MeTHF at 298 K.

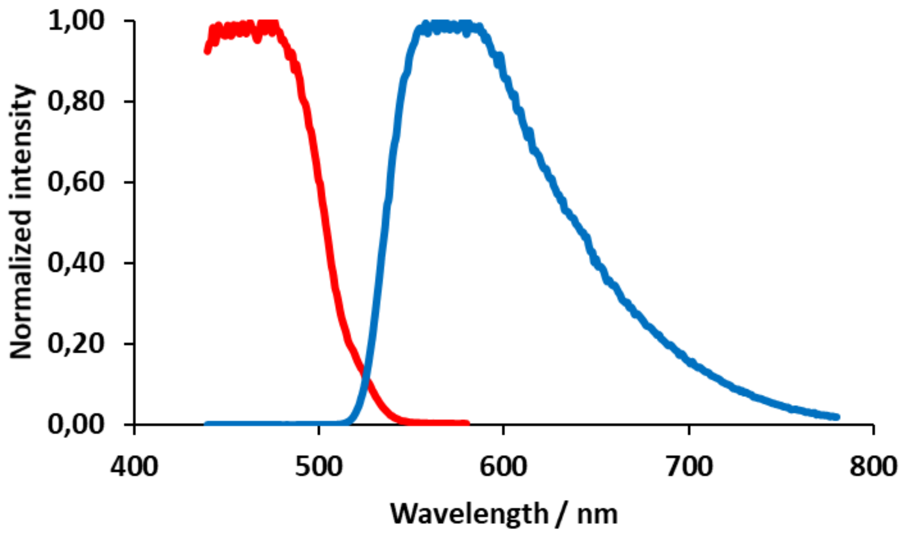

Figure S42. Normalized emission spectrum of complex **4** in a 1 x 10^{-5} M solution in 2-MeTHF at 77 K.

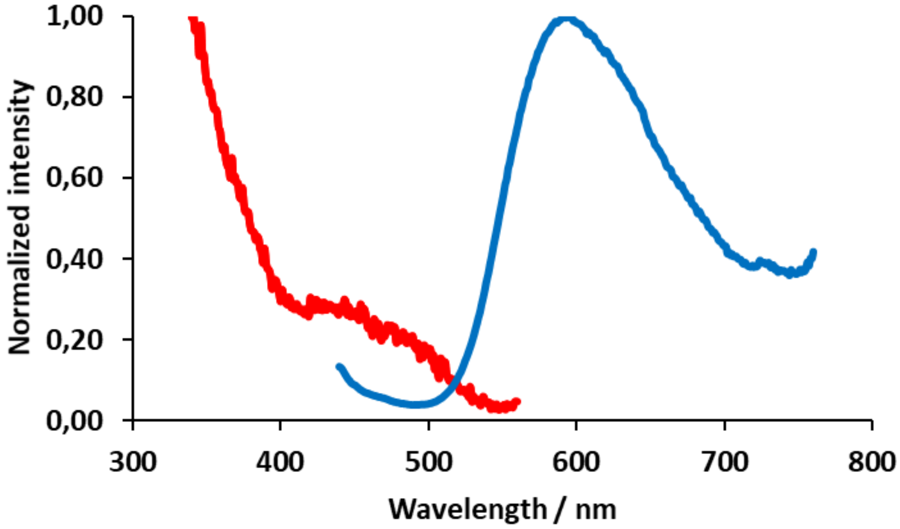

Figure S43. Normalized emission spectrum of complex **5** in PMMA film (5 wt%) at 298 K.

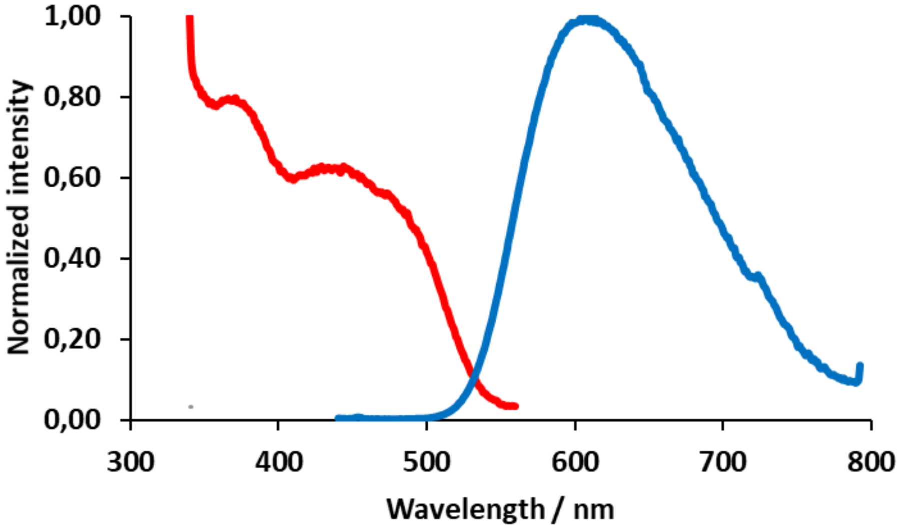

Figure S44. Normalized emission spectrum of complex **5** in a 1 x 10^{-5} M solution in 2-MeTHF at 298 K.

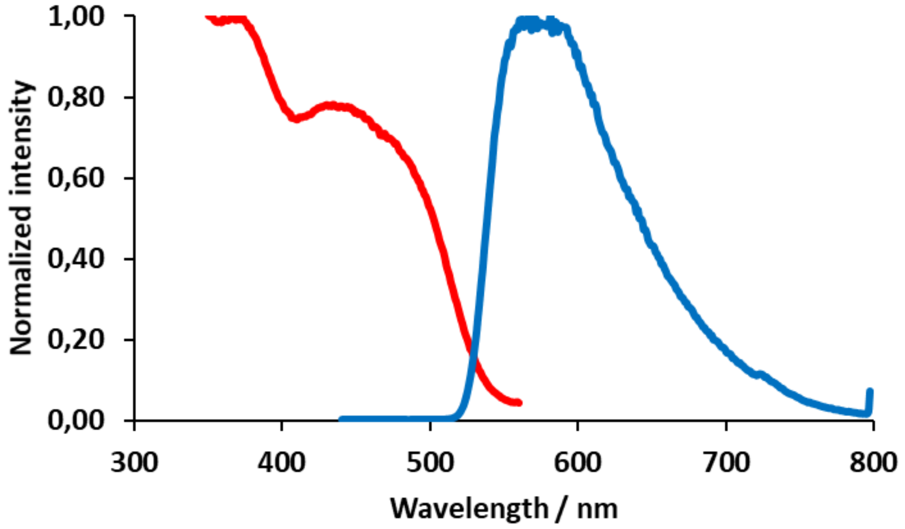

Figure S45. Normalized emission spectrum of complex **5** in a 1 x 10^{-5} M solution in 2-MeTHF at 77 K.

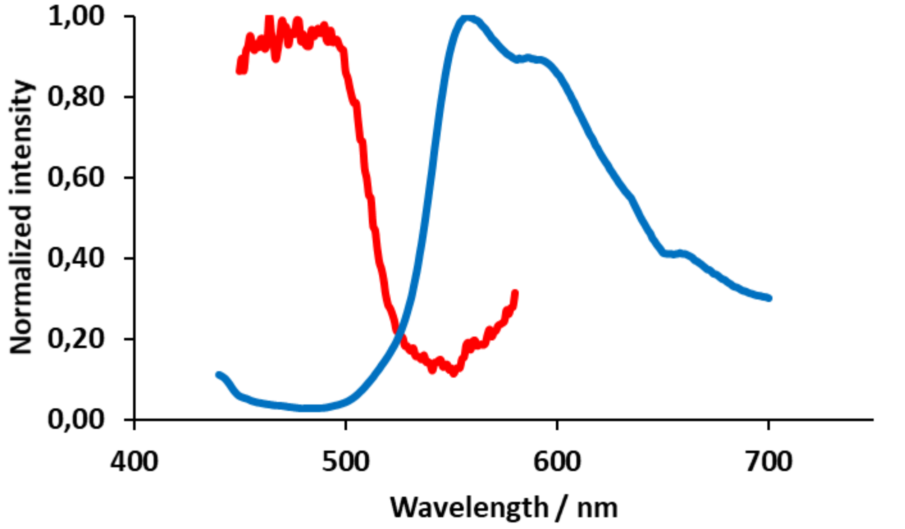

Figure S46. Normalized emission spectrum of complex **6** in PMMA film (5 wt%) at 298 K.

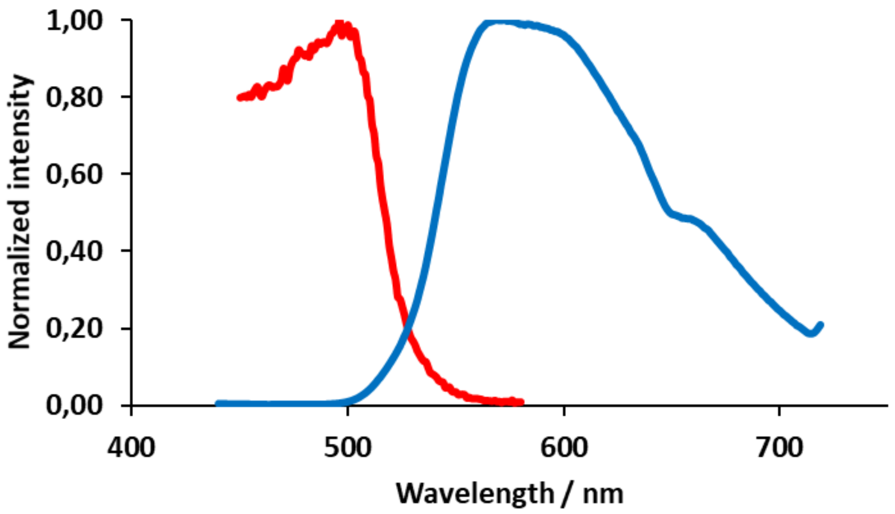

Figure S47. Normalized emission spectrum of complex **6** in a 1 x 10^{-5} M solution in 2-MeTHF at 298 K.

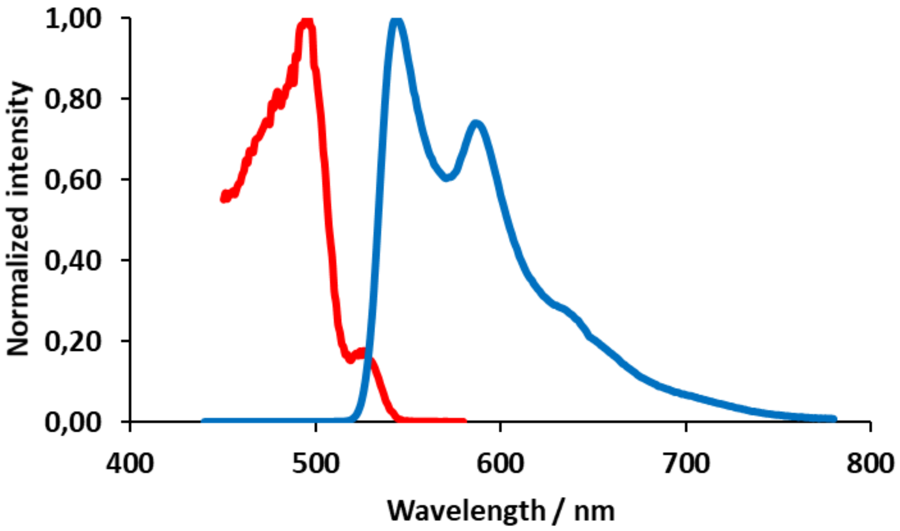

Figure S48. Normalized emission spectrum of complex **6** in a 1 x 10^{-5} M solution in 2-MeTHF at 77 K.

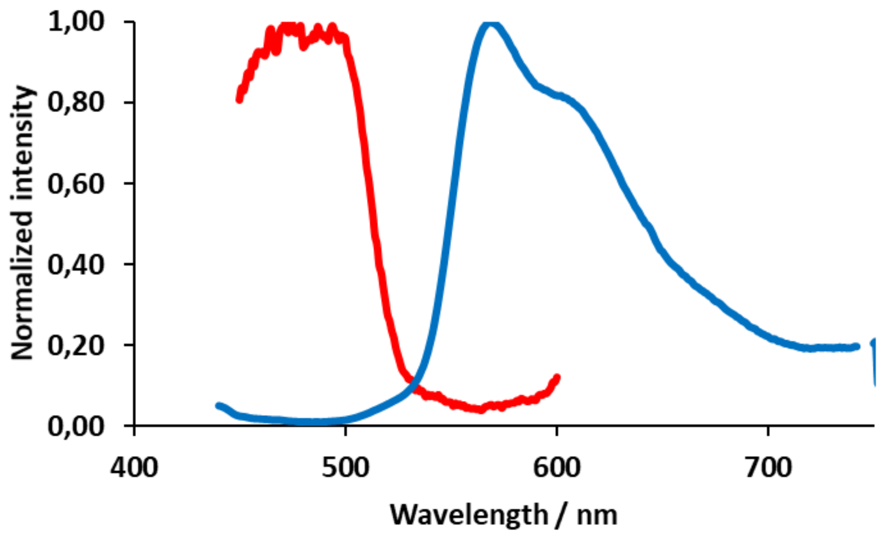

Figure S49. Normalized emission spectrum of complex **7** in PMMA film (5 wt%) at 298 K.

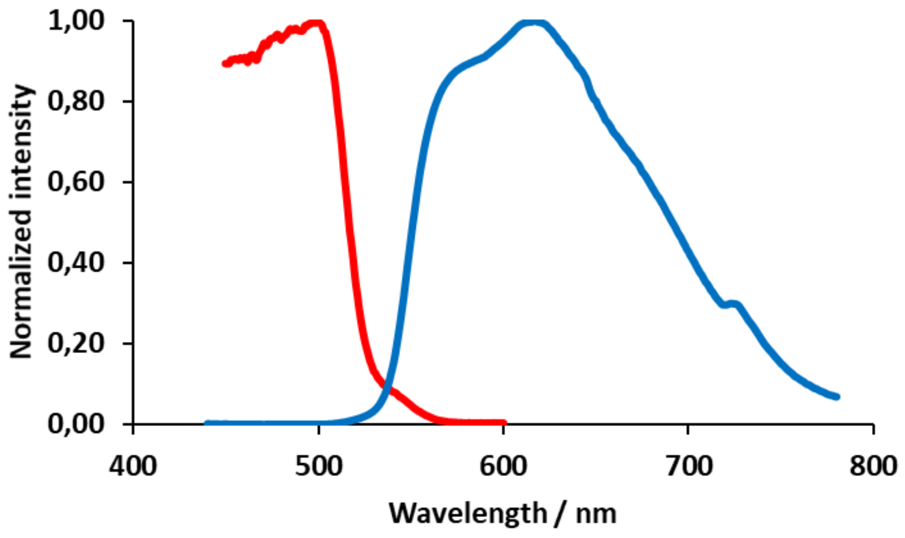

Figure S50. Normalized emission spectrum of complex **7** in a 1 x 10^{-5} M solution in 2-MeTHF at 298 K.

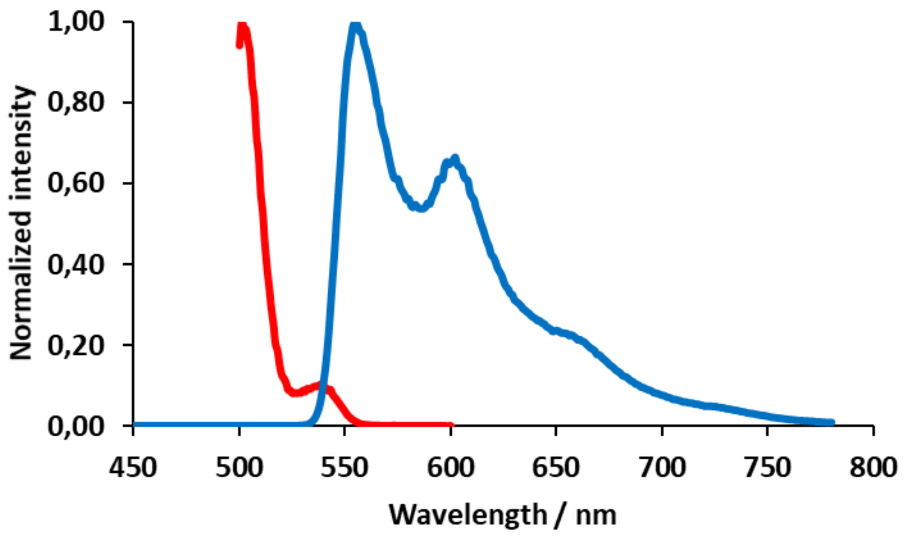

Figure S51. Normalized emission spectrum of complex **7** in a 1 x 10^{-5} M solution in 2-MeTHF at 77 K.

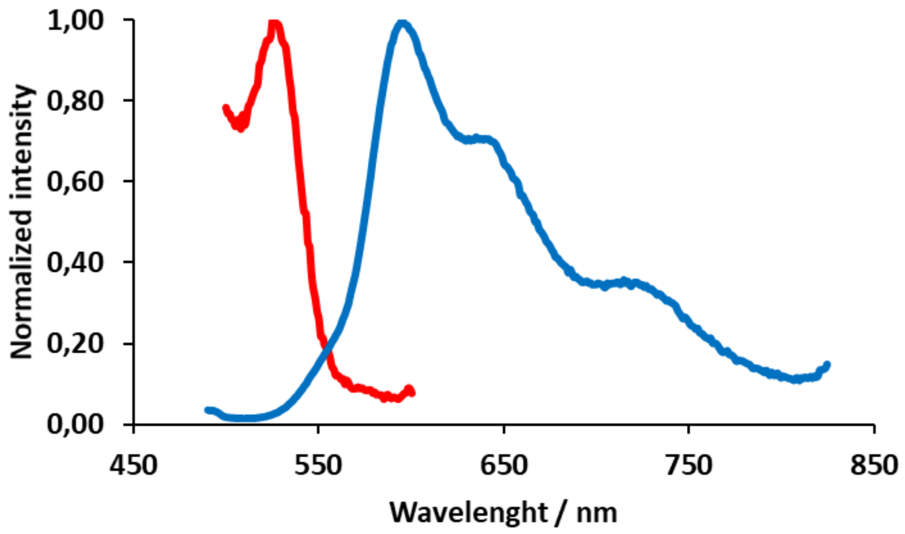

Figure S52. Normalized emission spectrum of complex **8** in PMMA film (5 wt%) at 298 K.

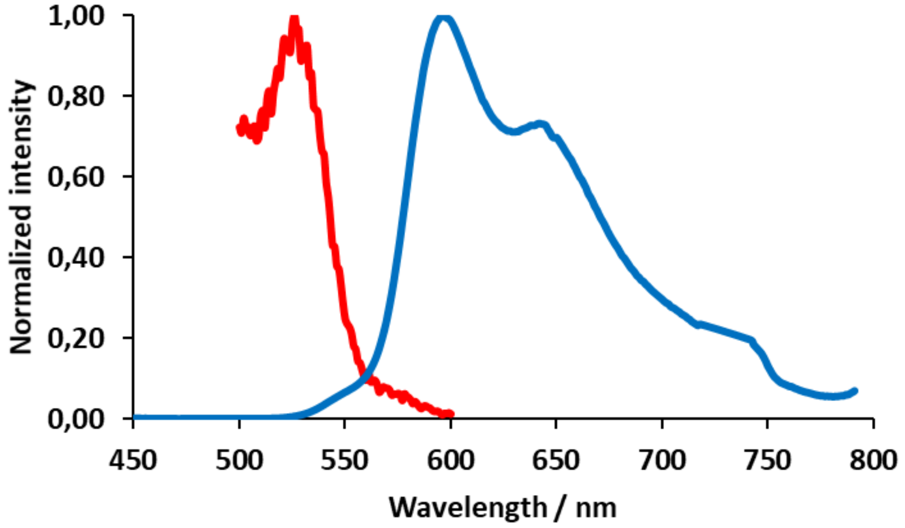

Figure S53. Normalized emission spectrum of complex **8** in a 1 x 10^{-5} M solution in 2-MeTHF at 298 K.

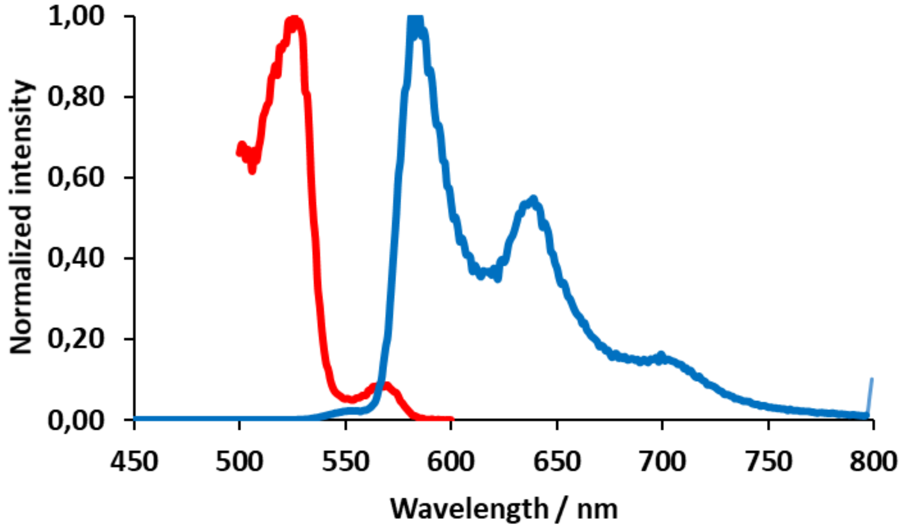

Figure S54. Normalized emission spectrum of complex **8** in a 1 x 10^{-5} M solution in 2-MeTHF at 77 K.

• **Cyclic Voltammograms**

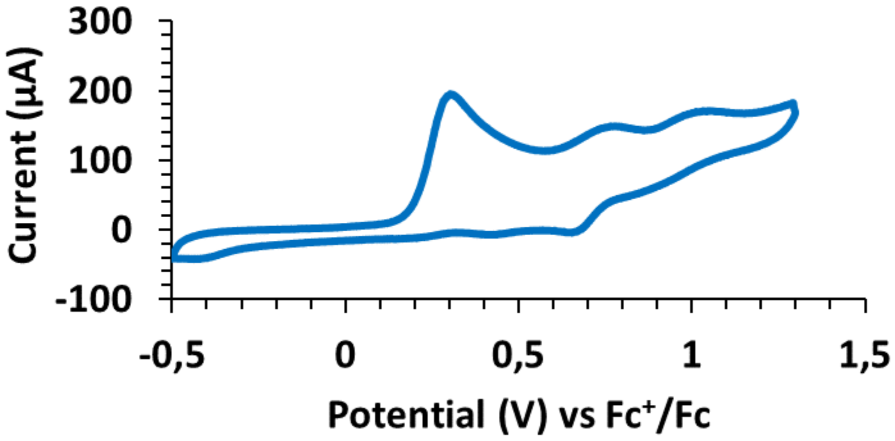

Figure S55. Cyclic voltammogram of complex **3** in dichloromethane 10^{-3} M solution with $[Bu_4N]PF_6$ as supporting electrolyte (0.1 M) at a scan rate of 100 Mv s^{-1}. The potentials were referenced to the Fc/Fc^+ couple.

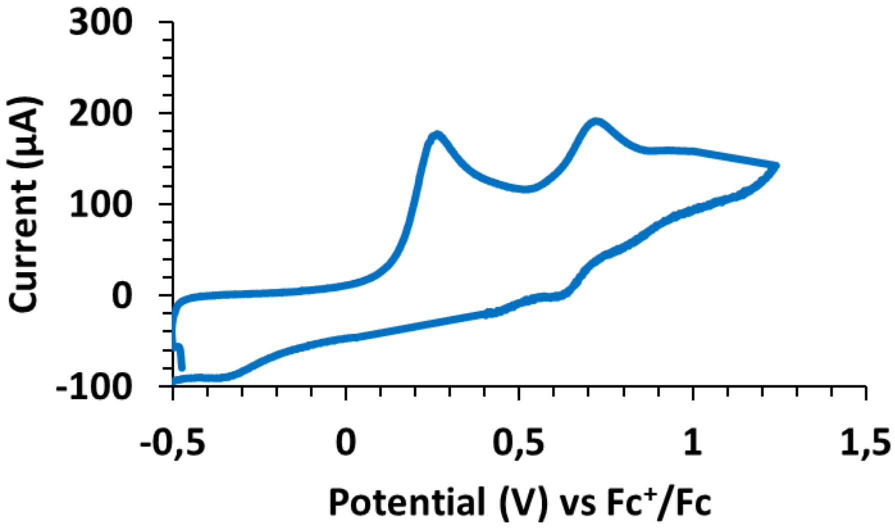

Figure S56. Cyclic voltammogram of complex **4** in dichloromethane 10^{-3} M solution with $[Bu_4N]PF_6$ as supporting electrolyte (0.1 M) at a scan rate of 100 Mv s^{-1}. The potentials were referenced to the Fc/Fc^+ couple.

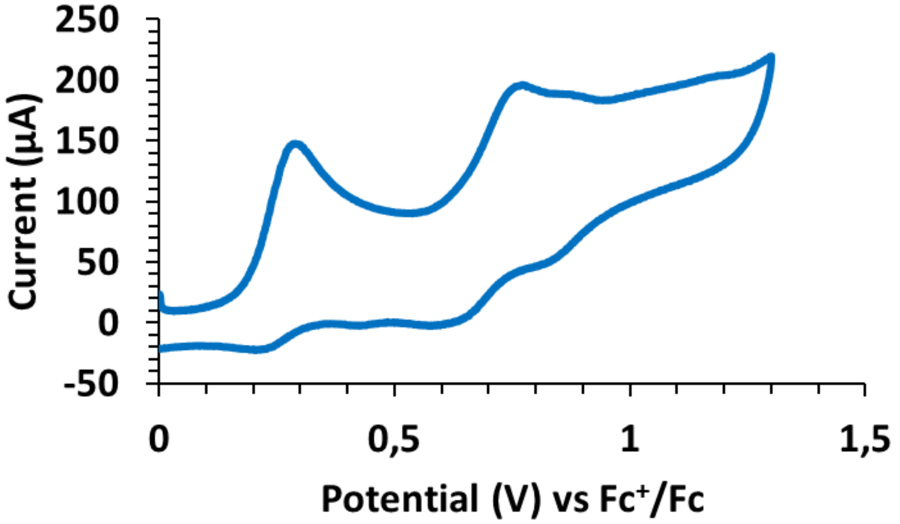

Figure S57. Cyclic voltammogram of complex **5** in dichloromethane 10^{-3} M solution with $[Bu_4N]PF_6$ as supporting electrolyte (0.1M) at a scan rate of 100 Mv s^{-1}. The potentials were referenced to the Fc/Fc^+ couple.

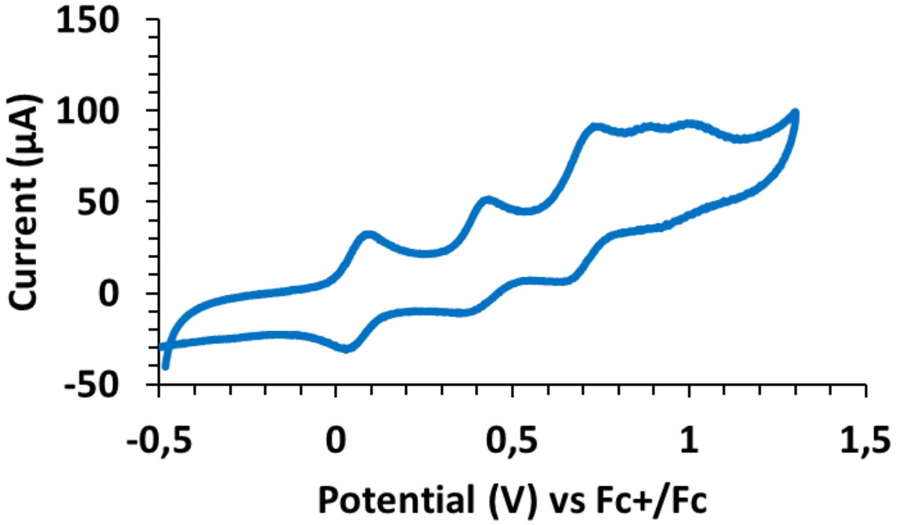

Figure S58. Cyclic voltammogram of complex **6** in dichloromethane 10^{-3} M solution with $[Bu_4N]PF_6$ as supporting electrolyte (0.1M) at a scan rate of 100 Mv s^{-1}. The potentials were referenced to the Fc/Fc^+ couple.

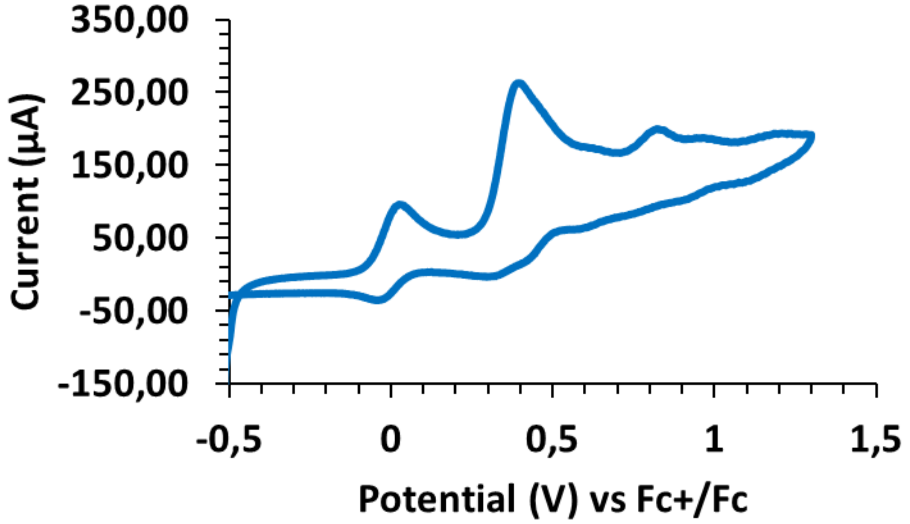

Figure S59. Cyclic voltammogram of complex **7** in dichloromethane 10^{-3} M solution with $[Bu_4N]PF_6$ as supporting electrolyte (0.1M) at a scan rate of 100 Mv s^{-1}. The potentials were referenced to the Fc/Fc^+ couple.

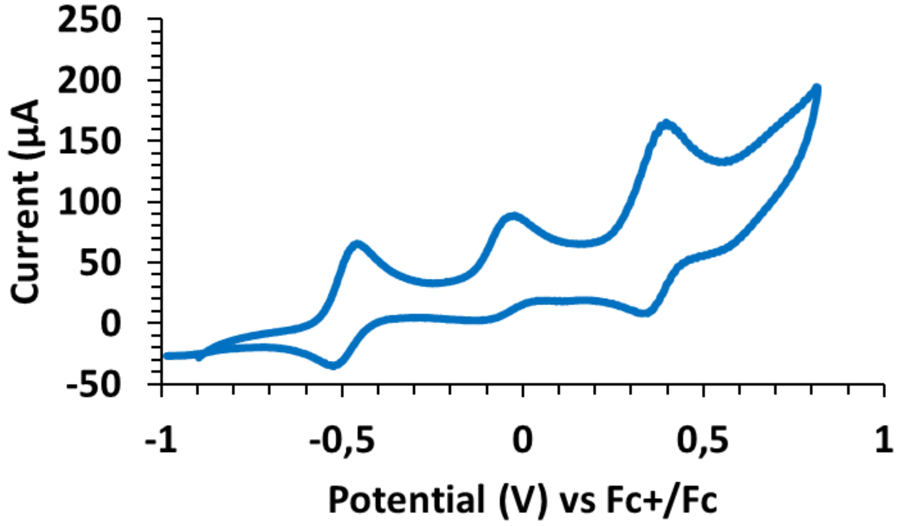

Figure S60. Cyclic voltammogram of complex **8** in dichloromethane 10^{-3} M solution with $[Bu_4N]PF_6$ as supporting electrolyte (0.1M) at a scan rate of 100 Mv s^{-1}. The potentials were referenced to the Fc/Fc^+ couple.

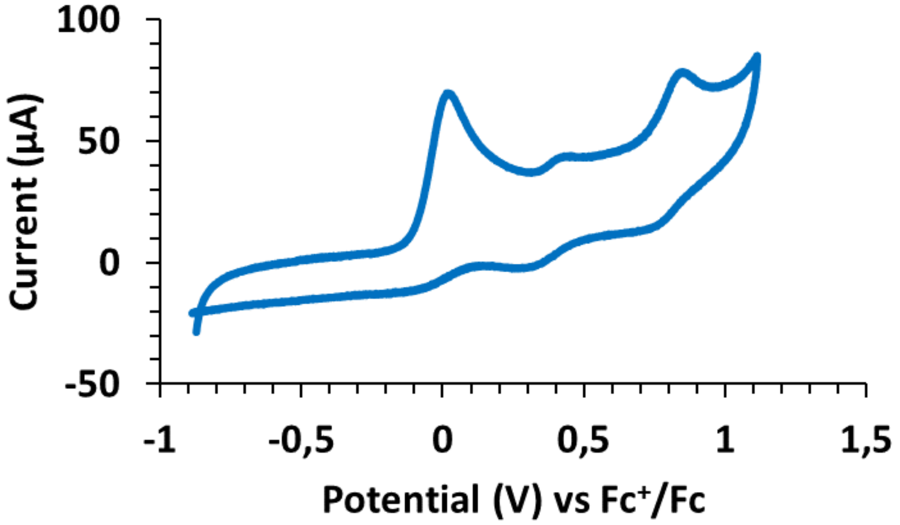

Figure S61. Cyclic voltammogram of complex **9** in dichloromethane 10^{-3} M solution with $[Bu_4N]PF_6$ as supporting electrolyte (0.1M) at a scan rate of 100 Mv s^{-1}. The potentials were referenced to the Fc/Fc^+ couple.

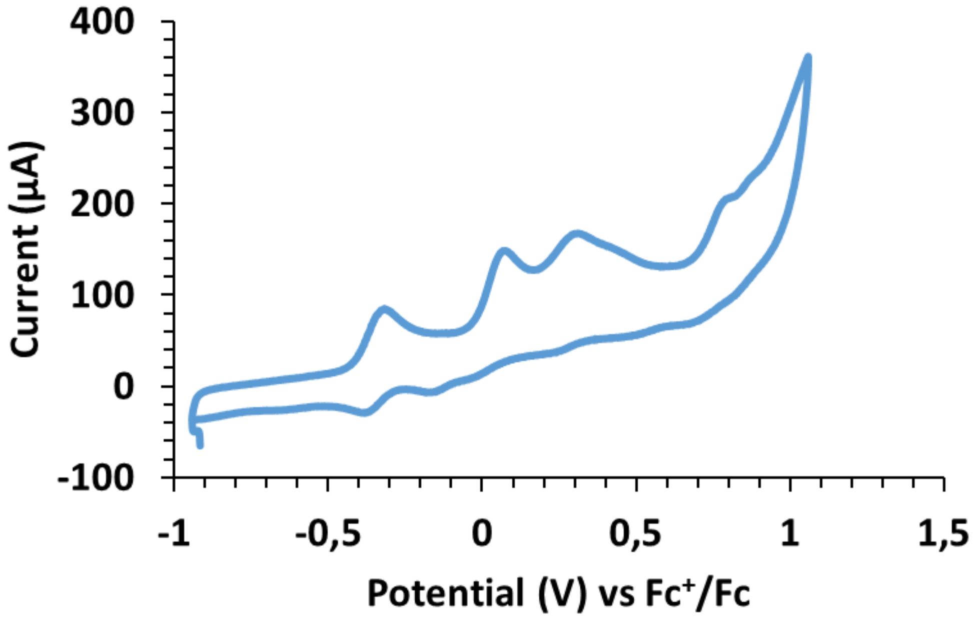

Figure S62. Cyclic voltammogram of complex **10** in dichloromethane 10^{-3} M solution with $[Bu_4N]PF_6$ as supporting electrolyte (0.1M) at a scan rate of 100 Mv s^{-1}. The potentials were referenced to the Fc/Fc^+ couple.

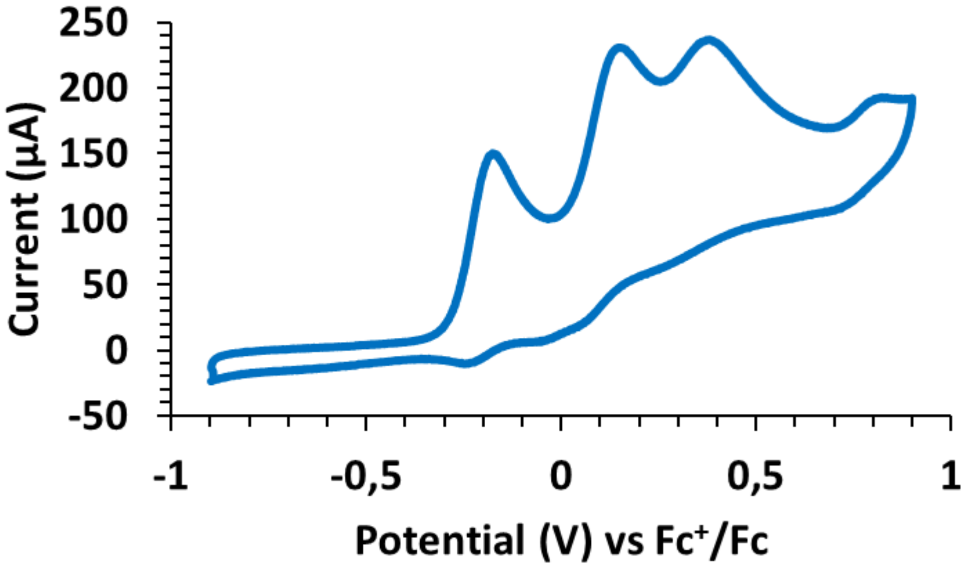

Figure S63. Cyclic voltammogram of complex **11** in dichloromethane 10^{-3} M solution with $[Bu_4N]PF_6$ as supporting electrolyte (0.1M) at a scan rate of 100 Mv s^{-1}. The potentials were referenced to the Fc/Fc^+ couple.

● Optimized Structures of Complexes 6, [6]$^{+}$, [6]$^{2+}$, [6]$^{3+}$, 7, [7]$^{+}$, [7]$^{2+}$, [7]$^{3+}$, 8, [8]$^{+}$, [8]$^{2+}$, [8]$^{3+}$, 10, [10]$^{+}$, [10]$^{2+}$, [10]$^{3+}$, 11, [11]$^{+}$, [11]$^{2+}$, and [11]$^{3+}$.

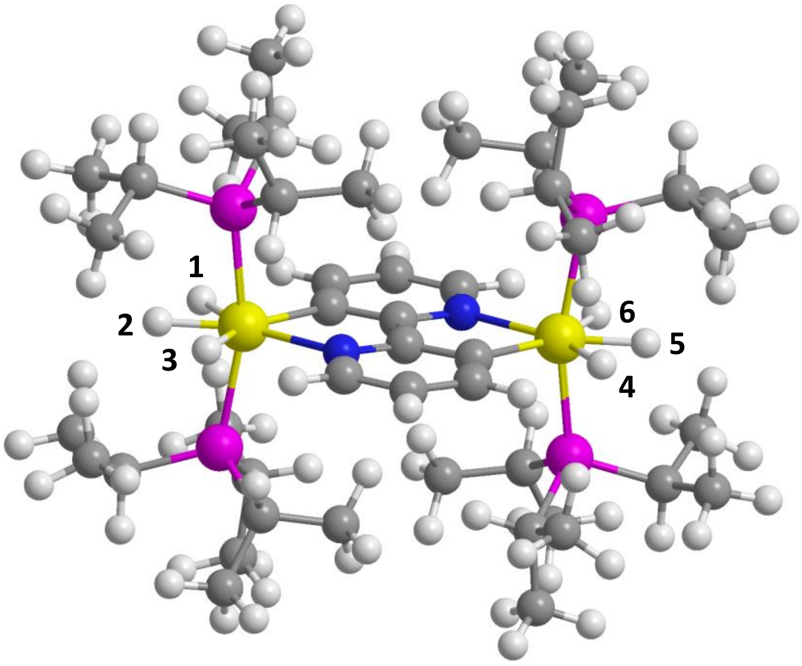

Figure S64. Optimized structure of complex **6**.

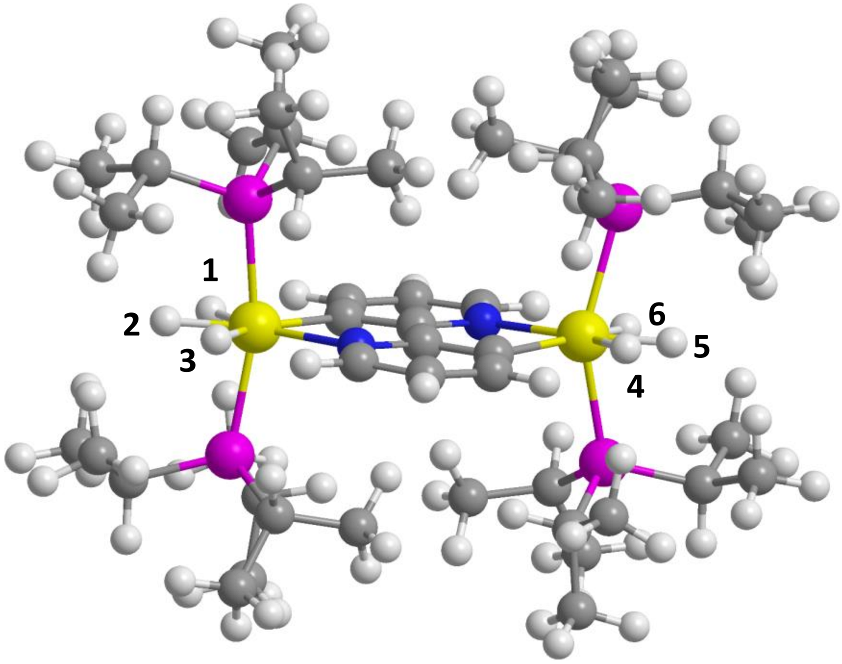

Figure S65. Optimized Structure of complex [**6**]$^{+}$.

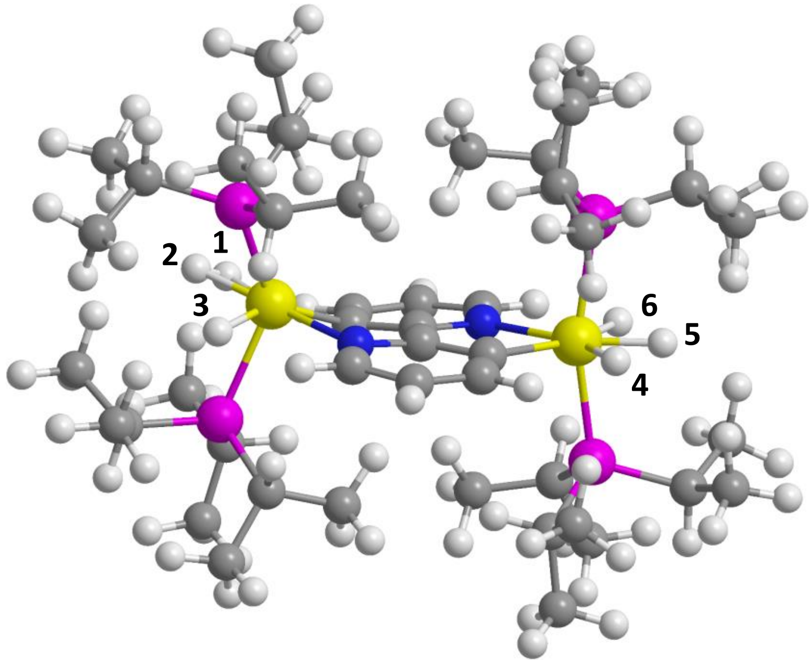

Figure S66. Optimized structure of complex [**6**]$^{2+}$.

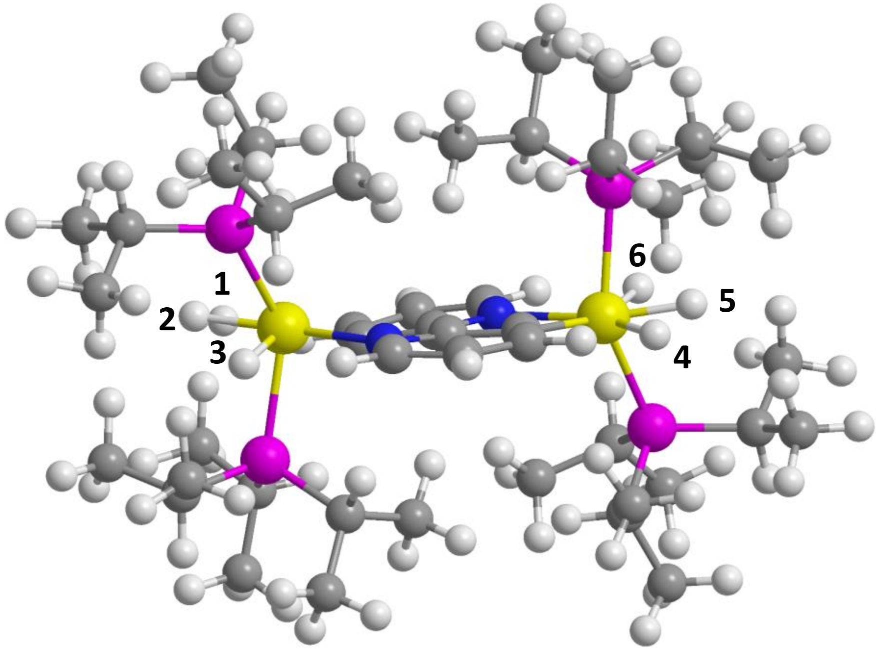

Figure S67. Optimized Structure of complex [**6**]$^{3+}$.

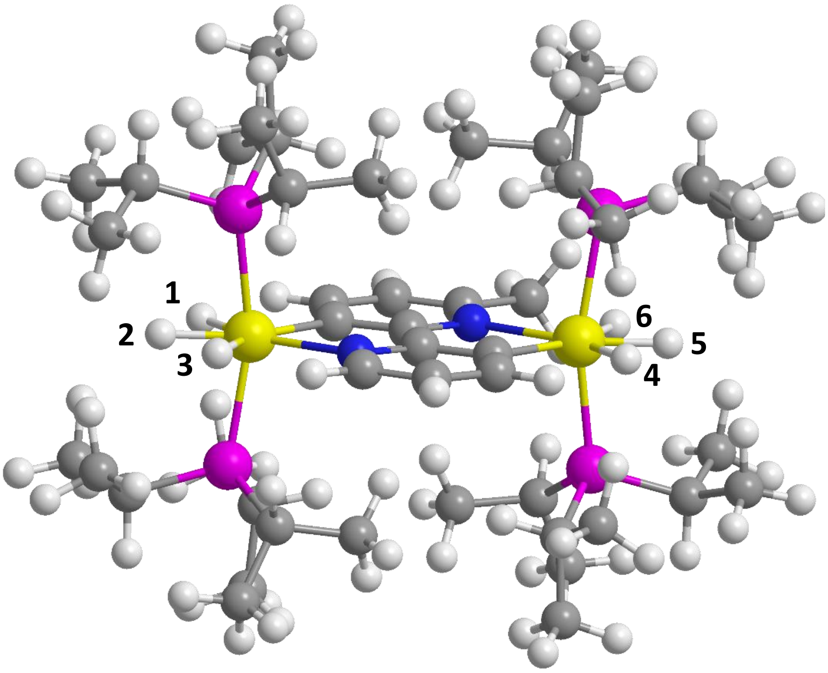

Figure S68. Optimized structure of complex **7**.

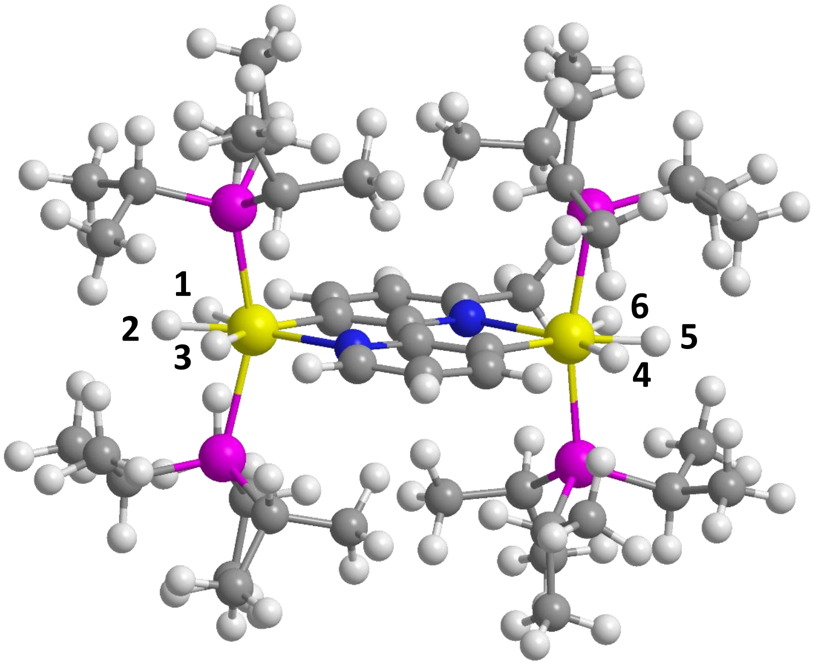

Figure S69. Optimized structure of complex [**7**]$^{+}$.

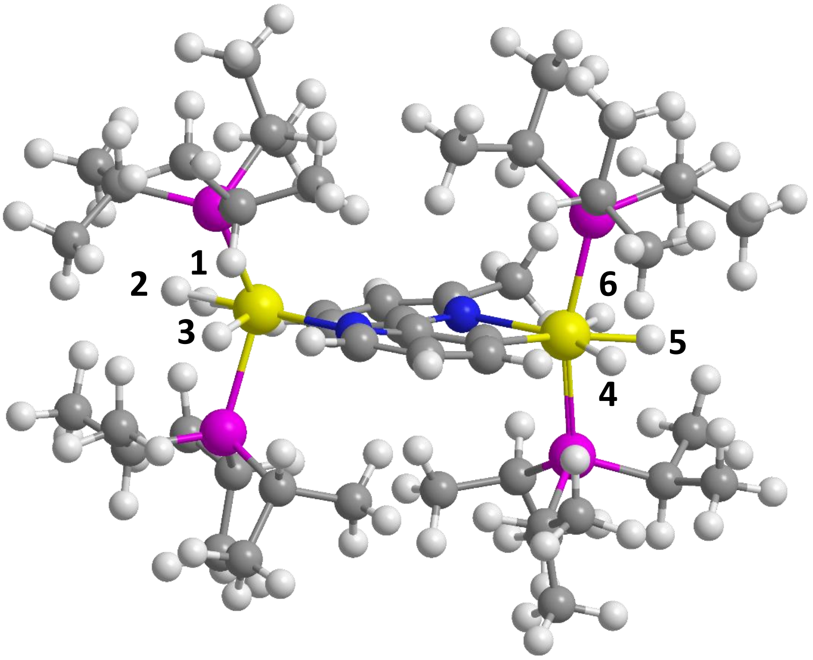

Figure S70. Optimized structure of complex [**7**]$^{2+}$.

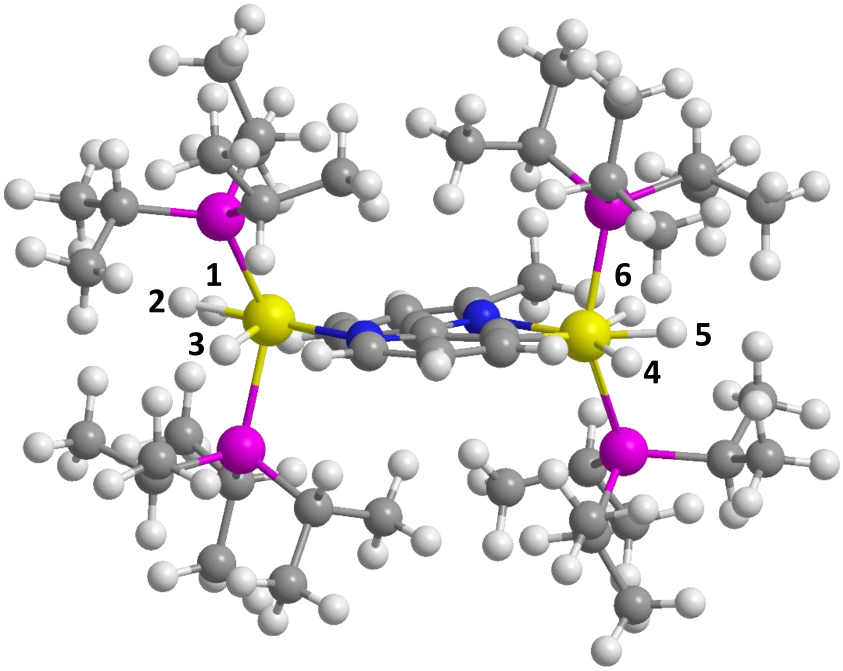

Figure S71. Optimized structure of complex [**7**]$^{3+}$.

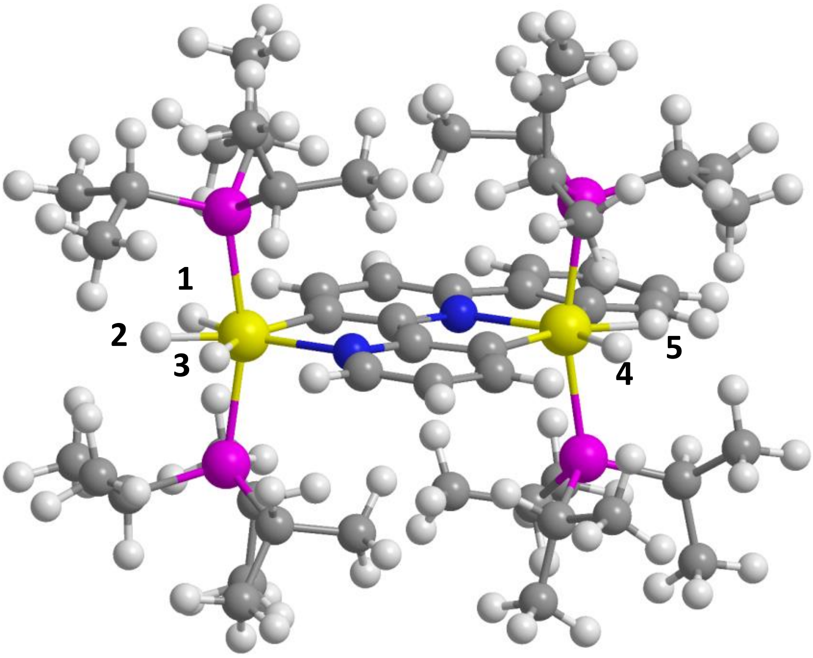

Figure S72. Optimized structure of complex **8**.

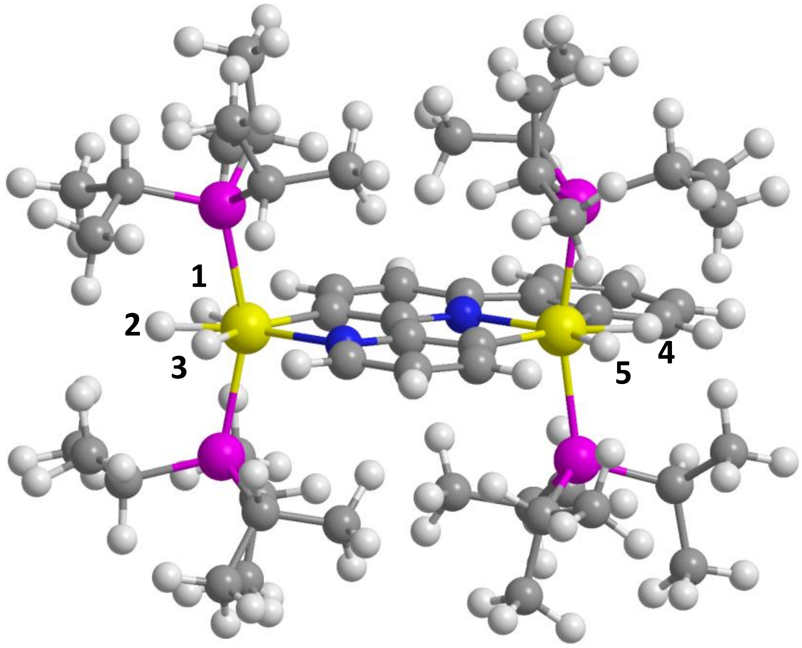

Figure S73. Optimized structure of complex [**8**]$^{+}$.

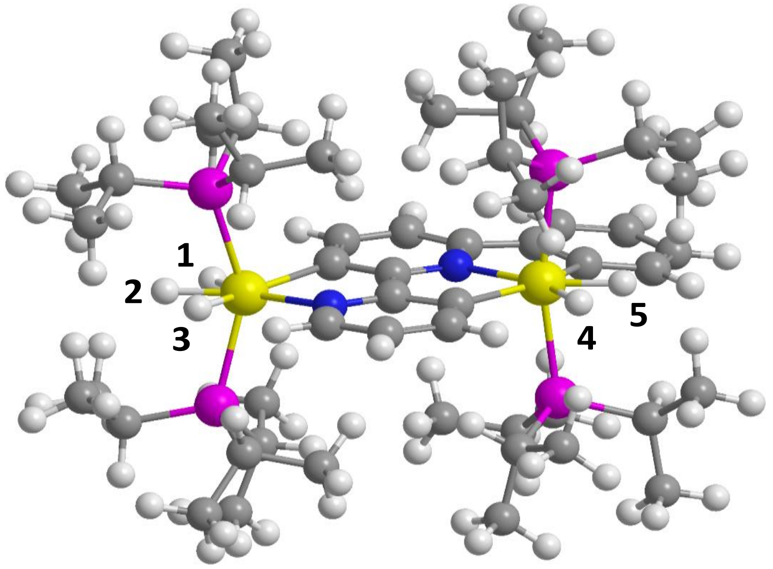

Figure S74. Optimized structure of complex [**8**]$^{2+}$.

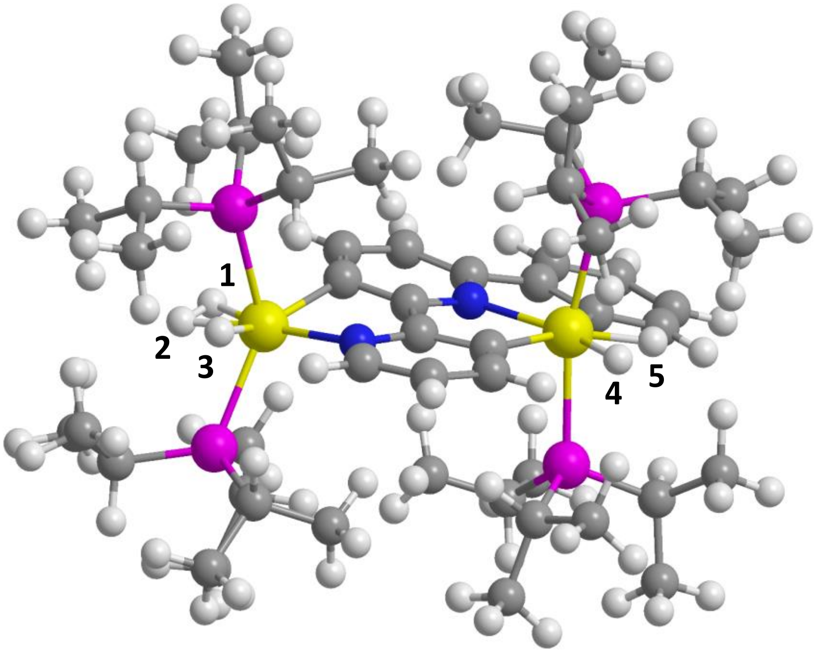

Figure S75. Optimized structure of complex [**8**]$^{3+}$.

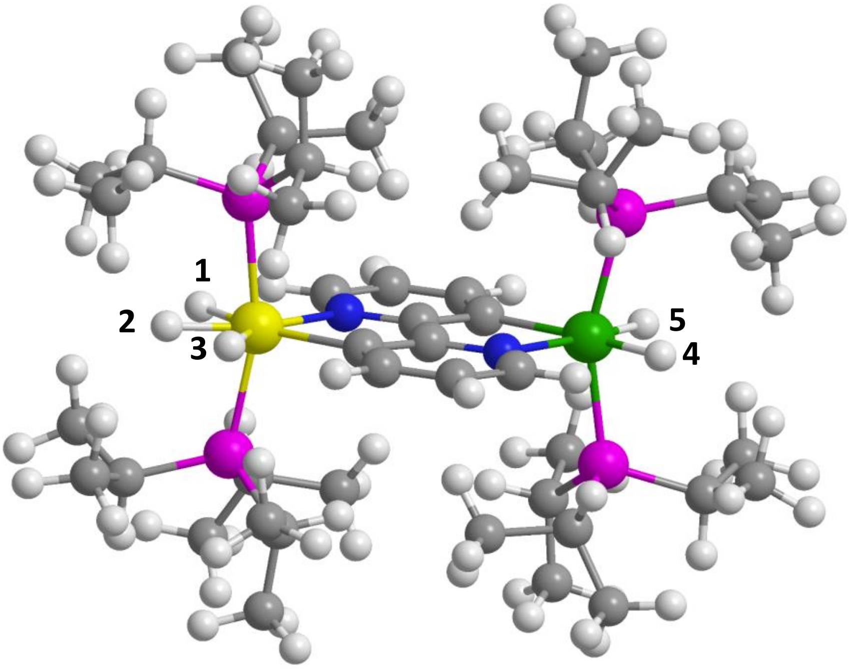

Figure S76. Optimized structure of complex **10**.

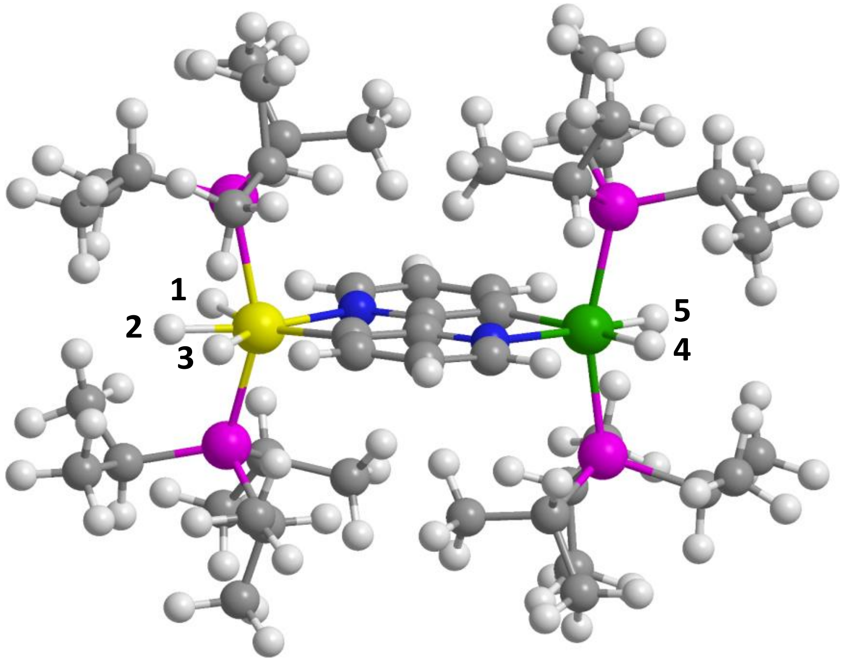

Figure S77. Optimized Structure of complex [**10**]$^{+}$.

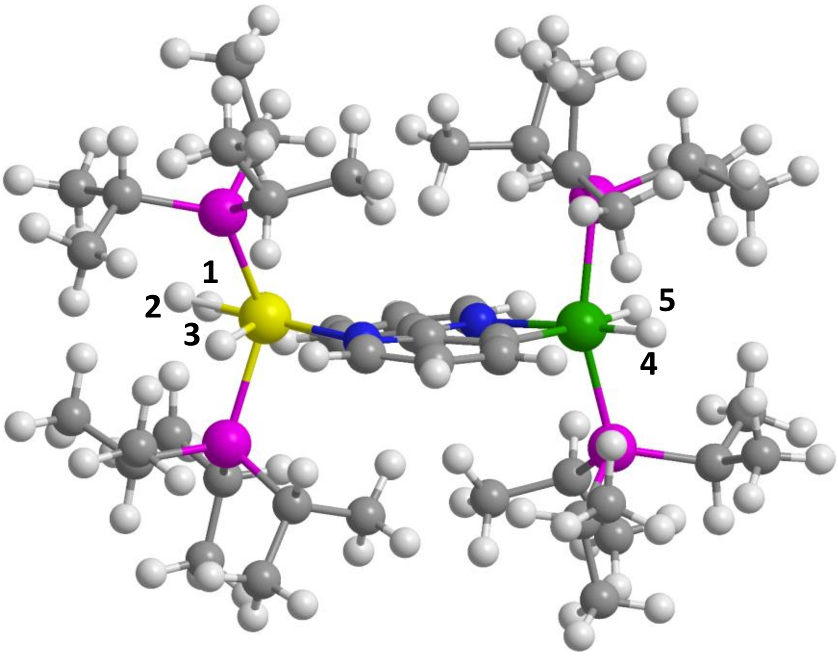

Figure S78. Optimized Structure of complex [**10**]$^{2+}$.

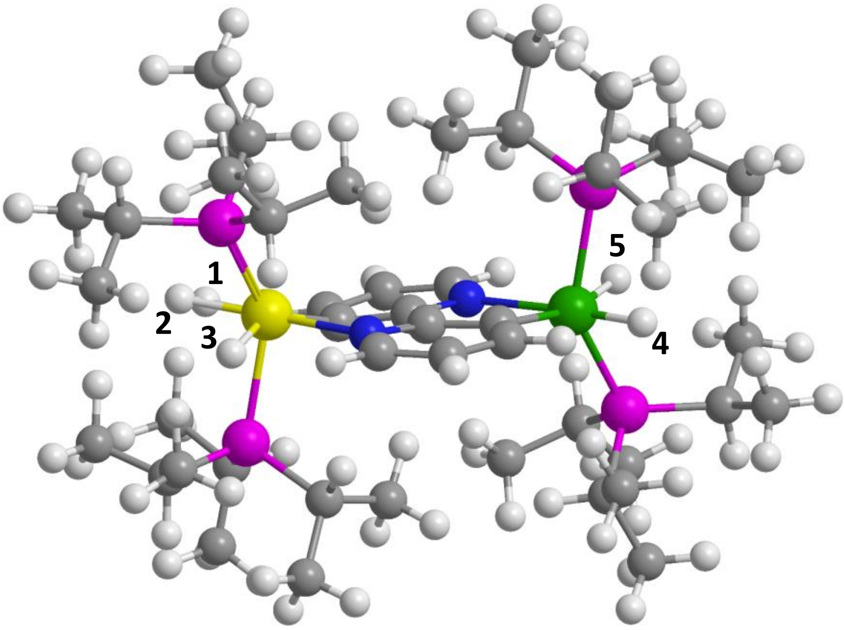

Figure S79. Optimized structure of complex [**10**]$^{3+}$.

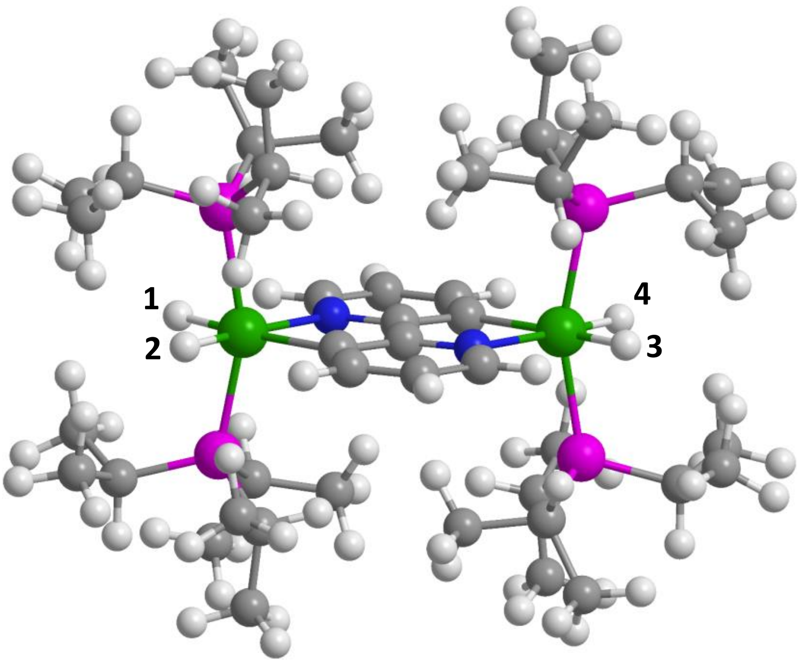

Figure S80. Optimized structure of complex **11**.

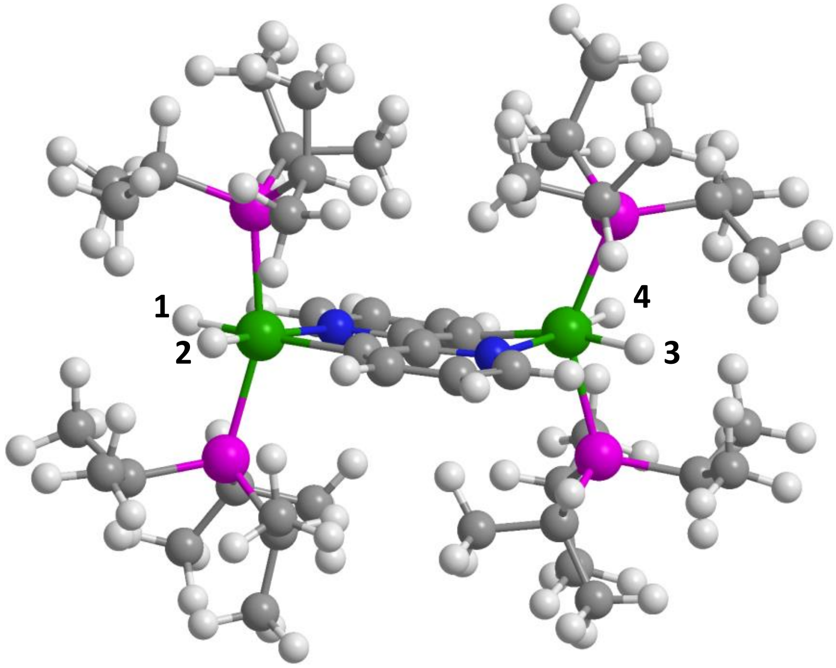

Figure S81. Optimized structure of complex [**11**]$^{+}$.

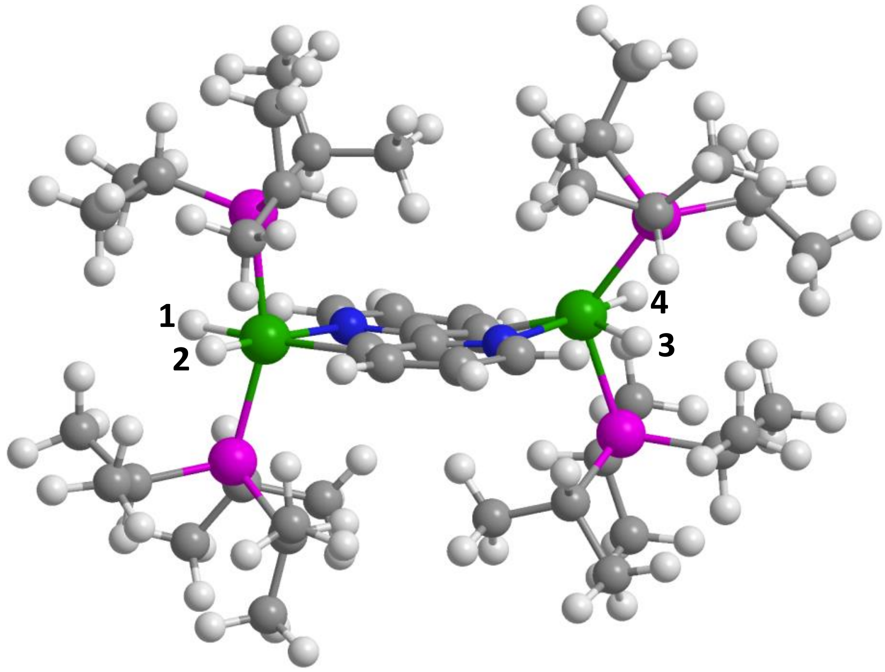

Figure S82. Optimized structure of complex [**11**]$^{2+}$.

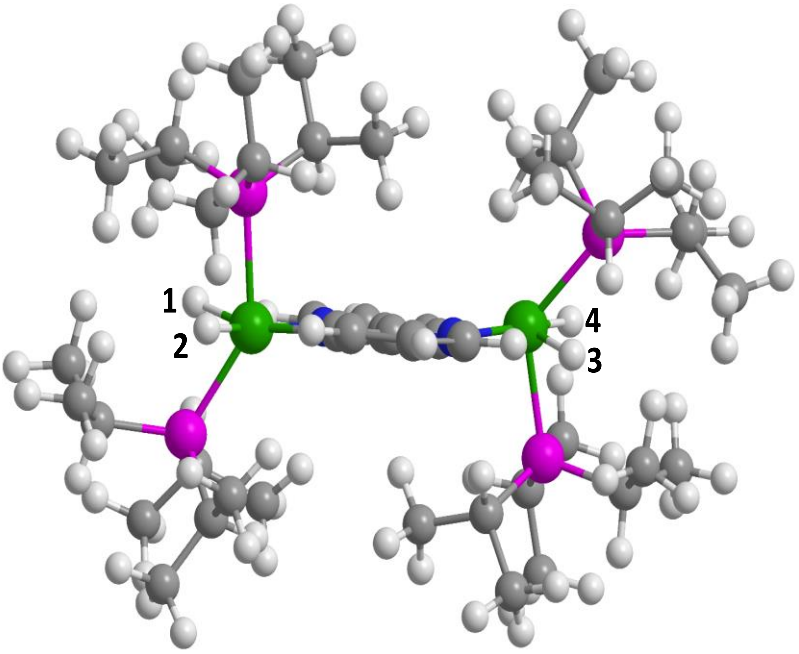

Figure S83. Optimized structure of complex [**11**]$^{3+}$.

● **UV-vis-NIR Spectra of Complexes 7, 8, and 11.**

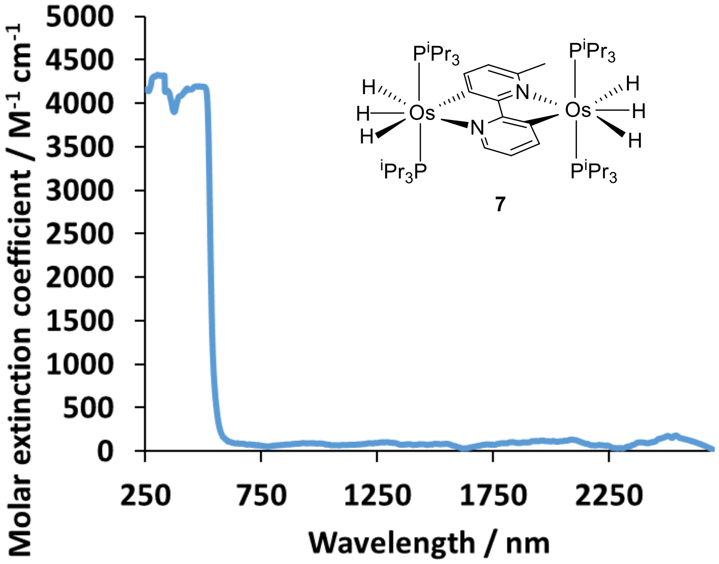

Figure S84. UV-vis-NIR spectrum of complex **7** in CH_2Cl_2**.**

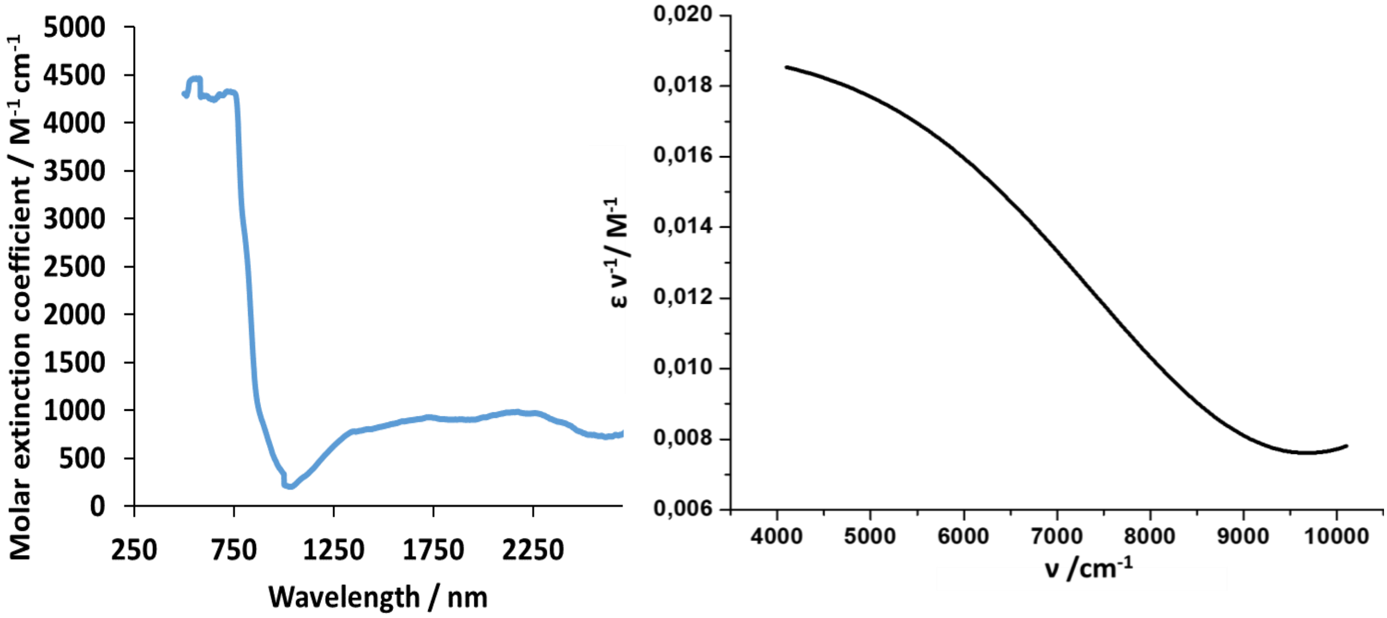

Figure S85. UV-vis-NIR spectrum of complex [**7**]$^+$ in CH_2Cl_2 (left) and the $\varepsilon\nu^{-1}$ (M^{-1}) versus ν (cm^{-1}) Gaussian fit (right).

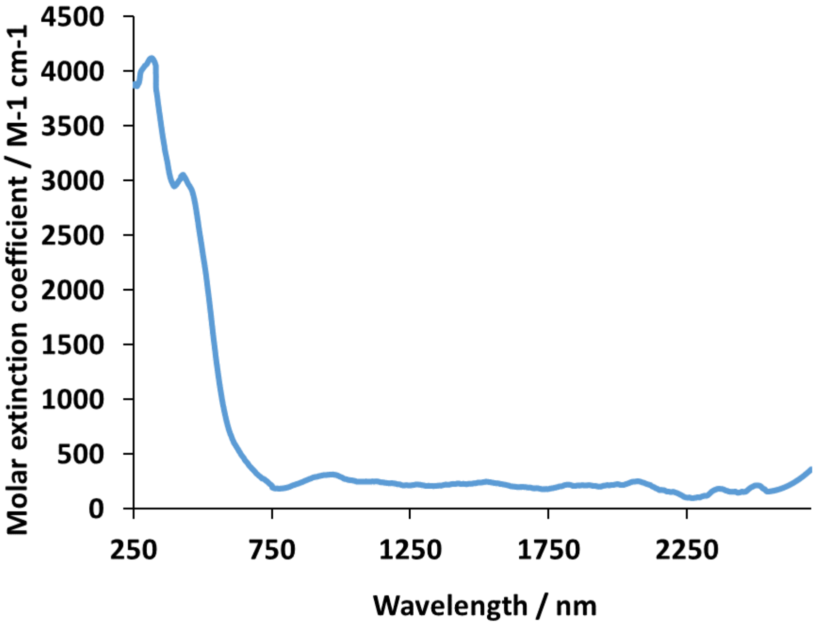

Figure S86. UV-vis-NIR spectrum of complex [**7**]$^{2+}$ in CH_2Cl_2.

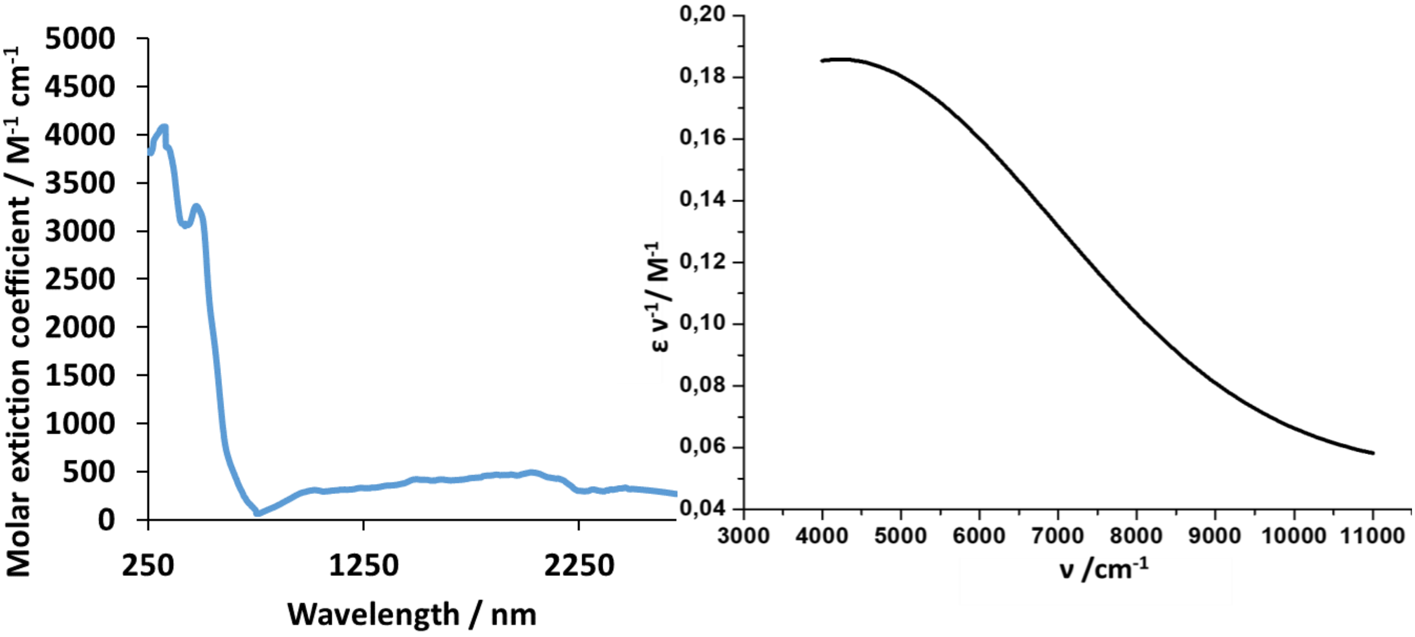

Figure S87. UV-vis-NIR spectrum of complex [**7**]$^{3+}$ in CH_2Cl_2 (left) and the $\varepsilon\nu^{-1}$ (M^{-1}) versus ν (cm^{-1}) Gaussian fit (right).

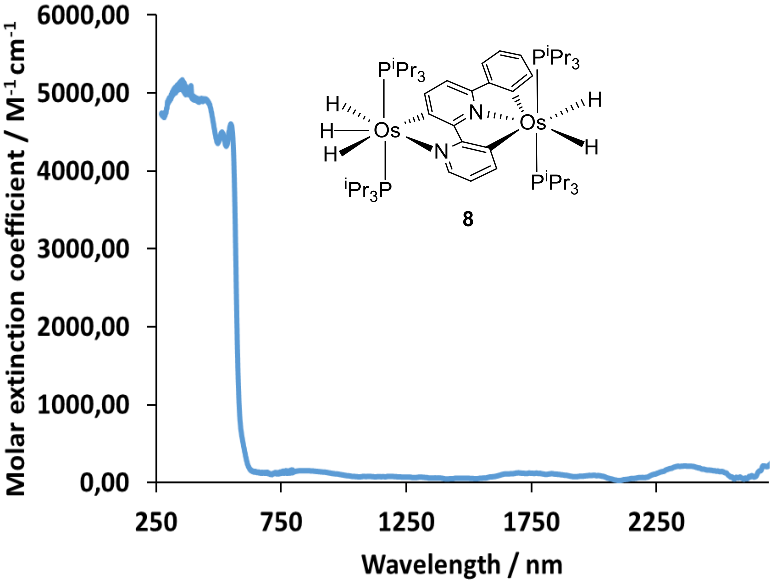

Figure S88. UV-vis-NIR spectrum of complex **8** in CH_2Cl_2.

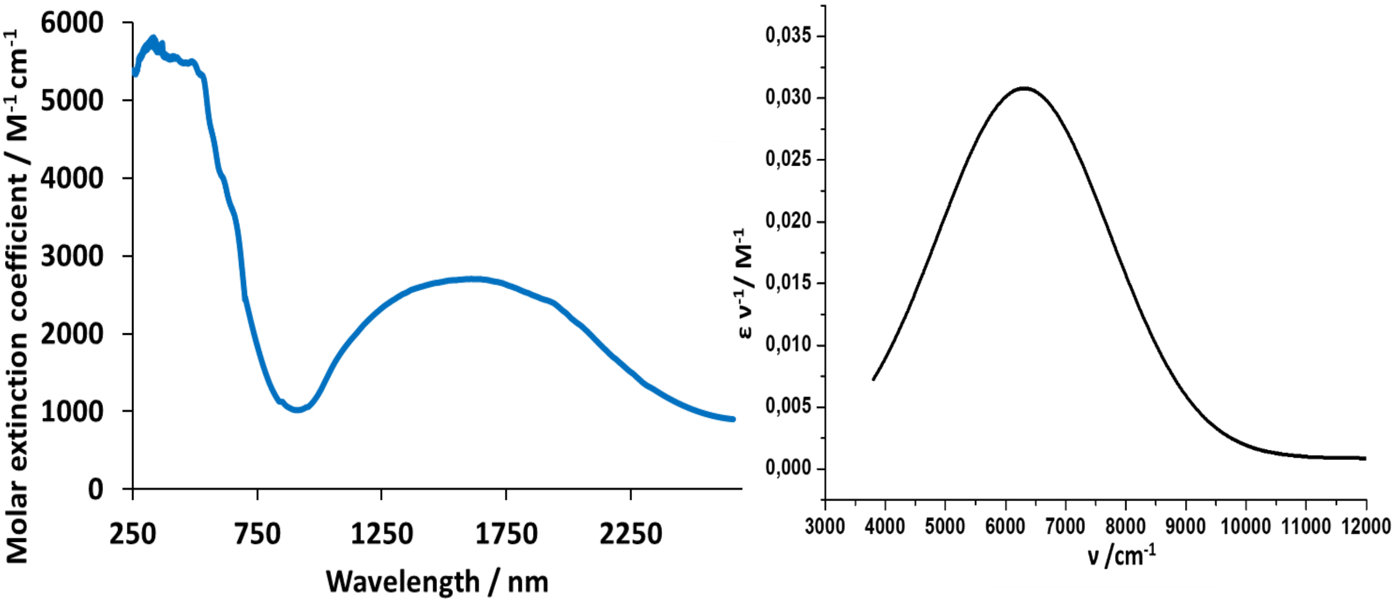

Figure S89. UV-vis-NIR spectrum of complex $[\mathbf{8}]^+$ in CH_2Cl_2 (left) and the $\varepsilon\nu^{-1}$ (M^{-1}) versus ν (cm^{-1}) Gaussian fit (right).

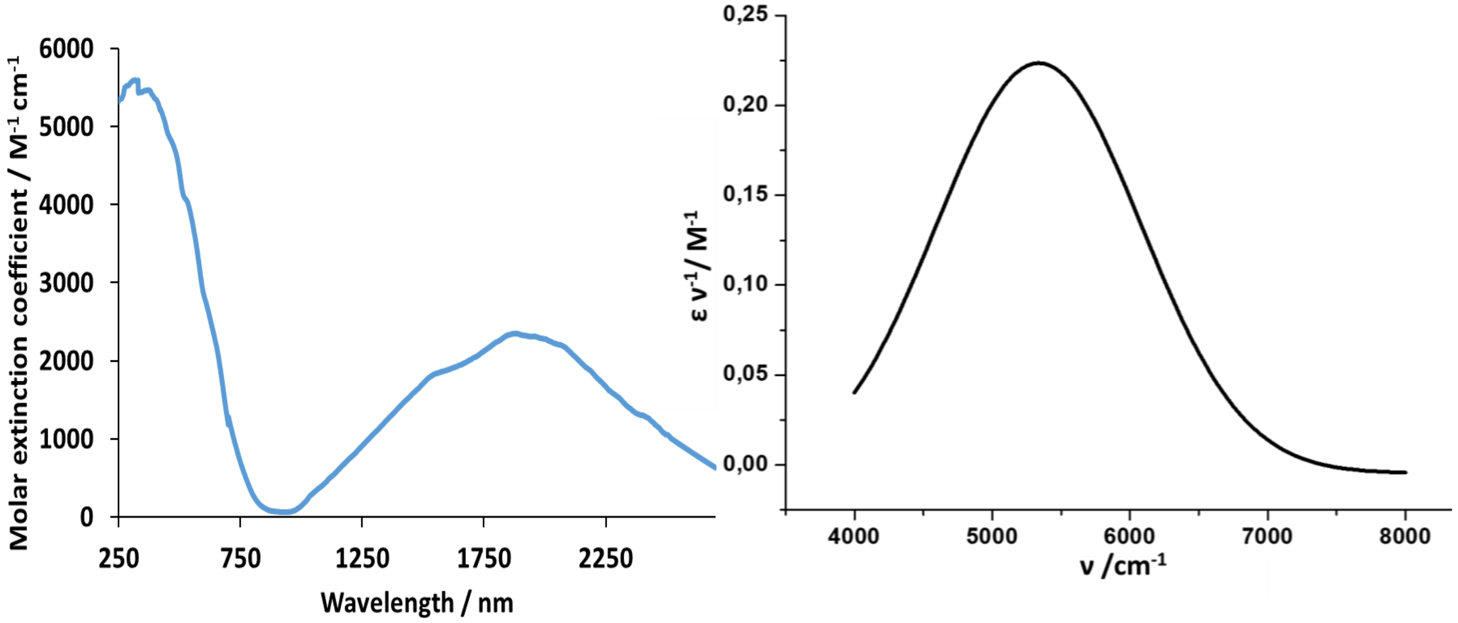

Figure S90. UV-vis-NIR spectrum of complex [**8**]$^{2+}$ in CH_2Cl_2 (left) and the εν^{-1} (M^{-1}) versus ν (cm^{-1}) Gaussian fit (right).

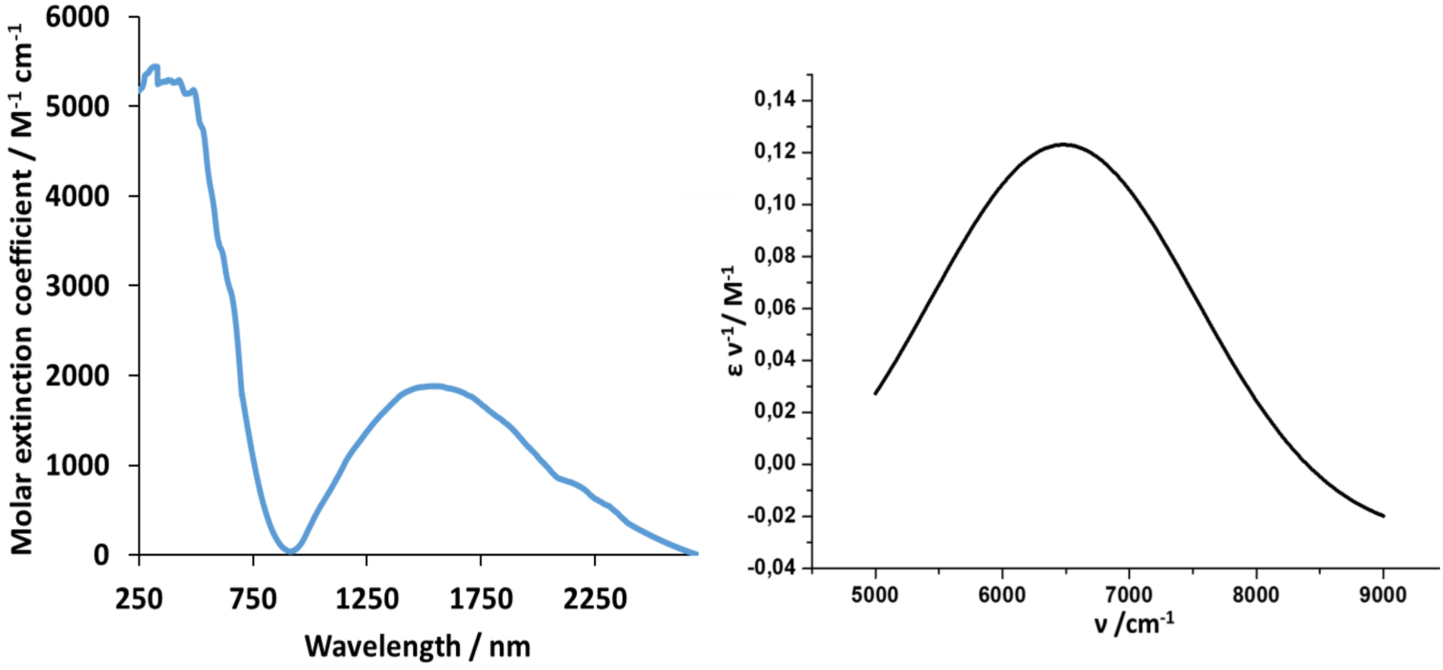

Figure S91. UV-vis-NIR spectrum of complex [**8**]$^{3+}$ in CH_2Cl_2 (left) and the εν^{-1} (M^{-1}) versus ν (cm^{-1}) Gaussian fit (right).

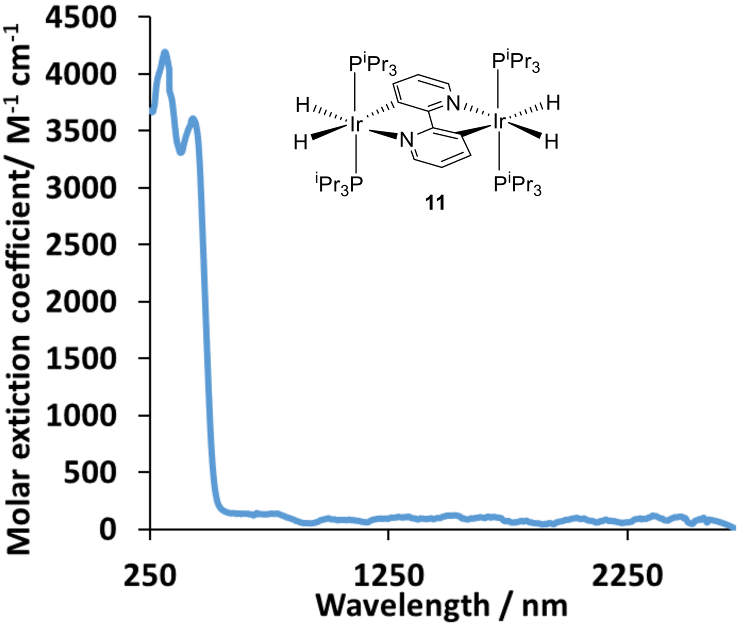

Figure S92. UV-vis-NIR spectrum of complex **11** in CH_2Cl_2.

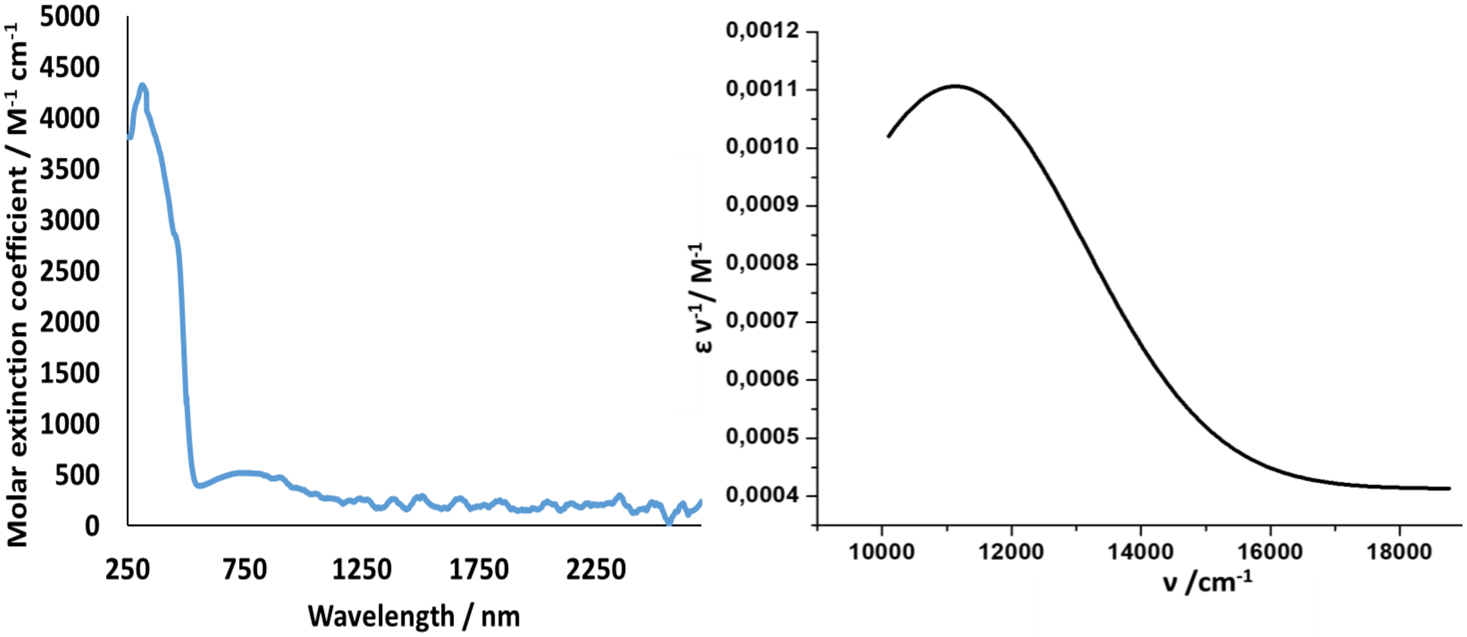

Figure S93. UV-vis-NIR spectrum of complex [**11**]$^+$ in CH_2Cl_2 (left) and the $\varepsilon\nu^{-1}$ (M^{-1}) versus ν (cm^{-1}) Gaussian fit (right).

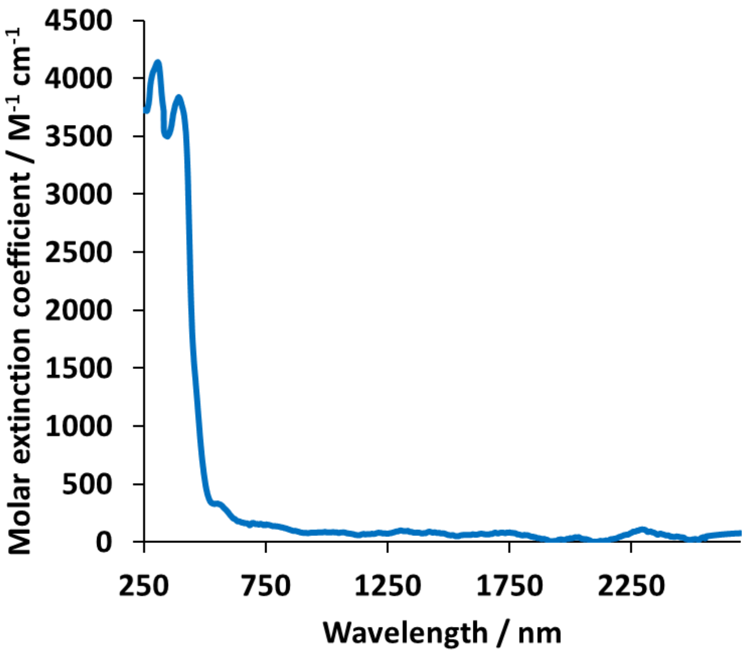

Figure S94. UV-vis-NIR spectrum of complex [**11**]$^{2+}$ in CH_2Cl_2.

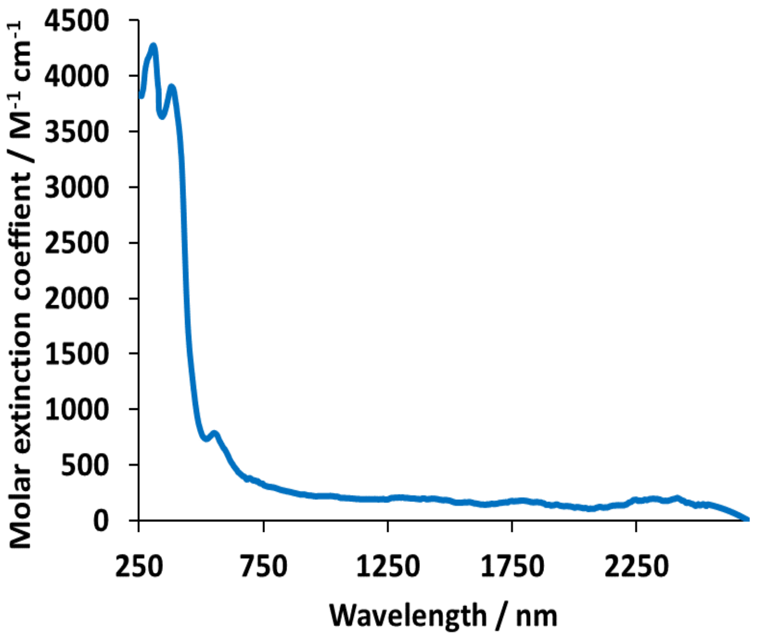

Figure S95. UV-vis-NIR spectrum of complex [**11**]$^{3+}$ in CH_2Cl_2.

● **UV-vis-NIR Spectra of Complexes [7]$^+$, [8]$^+$, [10]$^+$, and [11]$^+$ (Observed and Calculated)**

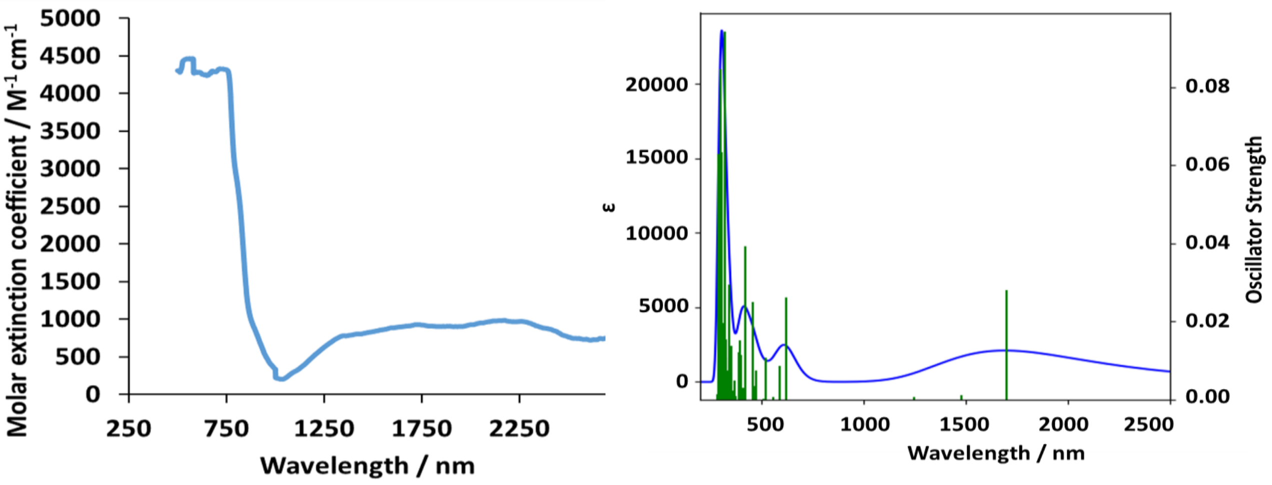

Figure S96. Observed UV-vis-NIR of complex [**7**]$^+$ in CH_2Cl_2 (1.0 x 10^{-3} M) and calculated (B3LYP(GD3)//SDD(f)/6-31G**) in CH_2Cl_2.

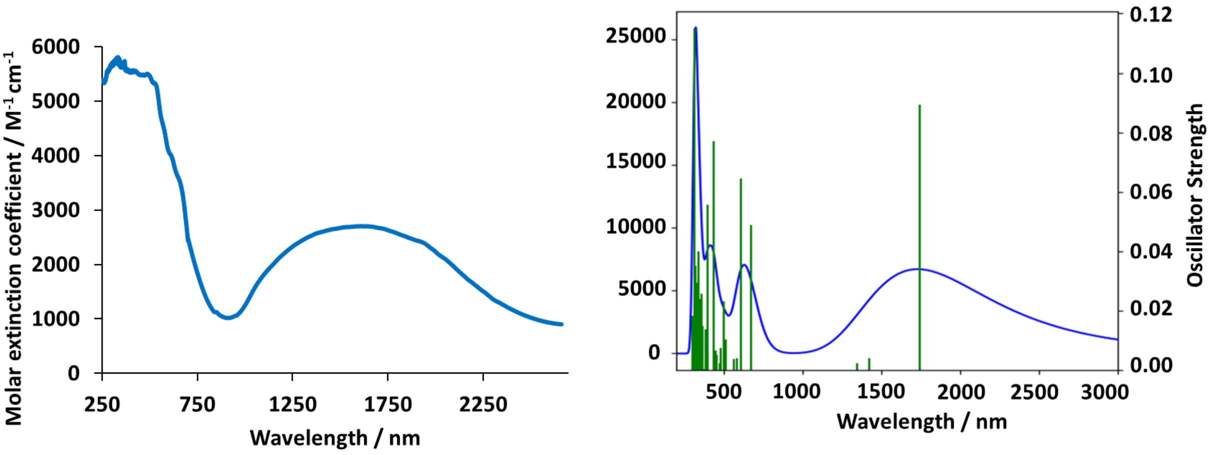

Figure S97. Observed UV-vis-NIR of complex [**8**]$^+$ in CH_2Cl_2 (1.0 x 10^{-3} M) and calculated (B3LYP(GD3)//SDD(f)/6-31G**) in CH_2Cl_2.

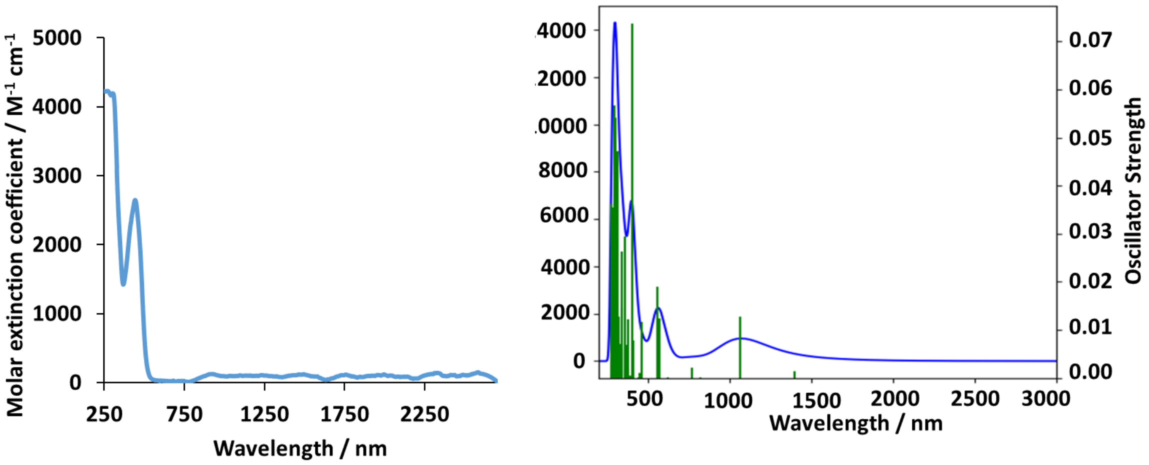

Figure S98. Observed UV-vis-NIR of complex [**10**]$^{+}$ in CH_2Cl_2 (1.0 x 10^{-3} M) and calculated (B3LYP(GD3)//SDD(f)/6-31G**) in CH_2Cl_2.

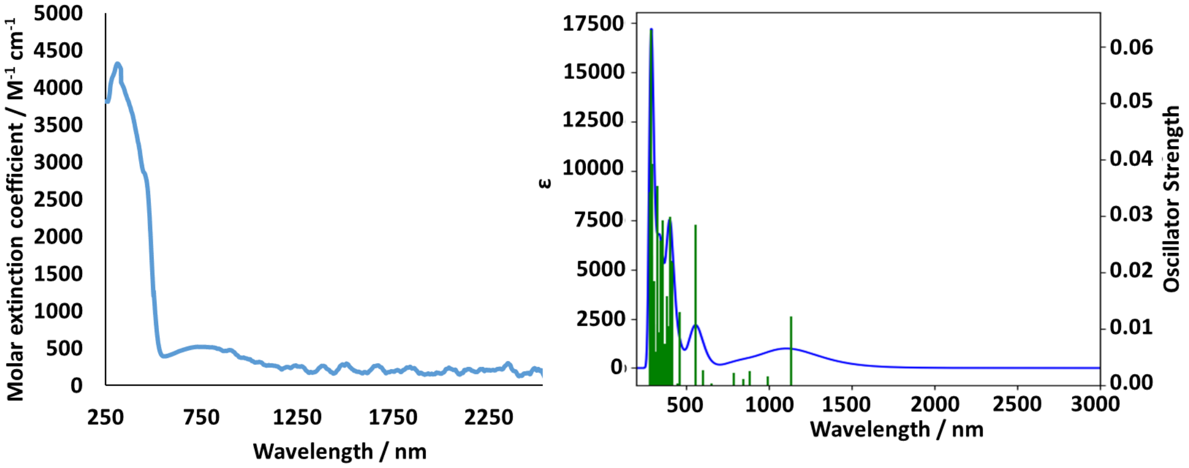

Figure S99. Observed UV-vis-NIR of complex [**11**]$^{+}$ in CH_2Cl_2 (1.0 x 10^{-3} M) and calculated (B3LYP(GD3)//SDD(f)/6-31G**) in CH_2Cl_2.

● Calculated HOMO, SOMO and LUMO of complexes 6, [6]$^{+}$, [6]$^{2+}$, [6]$^{3+}$, 7, [7]$^{+}$, [7]$^{2+}$, [7]$^{3+}$, 8, [8]$^{+}$, [8]$^{2+}$, [8]$^{3+}$, 10, [10]$^{+}$, [10]$^{2+}$, [10]$^{3+}$, 11, [11]$^{+}$, [11]$^{2+}$, and [11]$^{3+}$

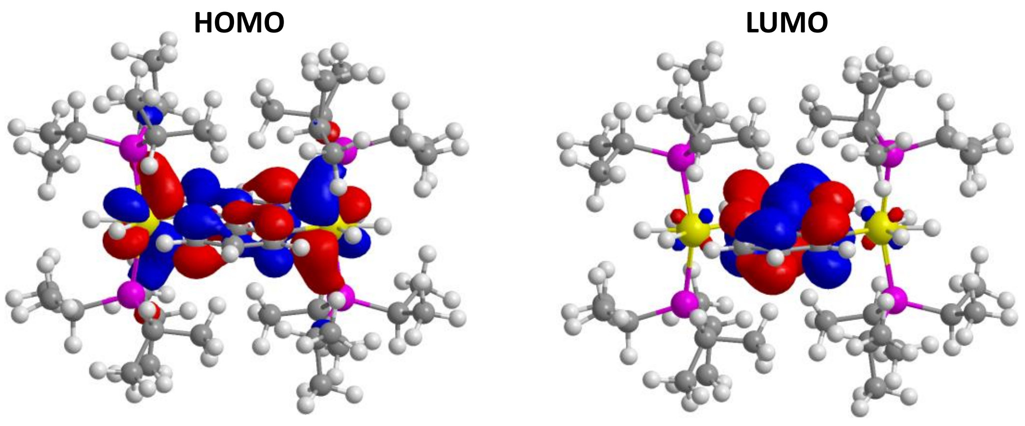

Figure S100. HOMO and LUMO of complex **6**.

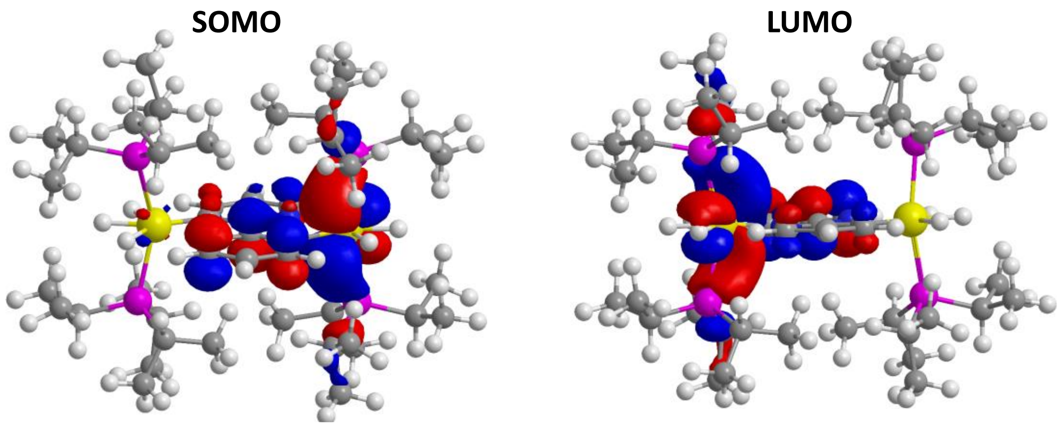

Figure S101. SOMO and LUMO of complex [**6**]$^{+}$.

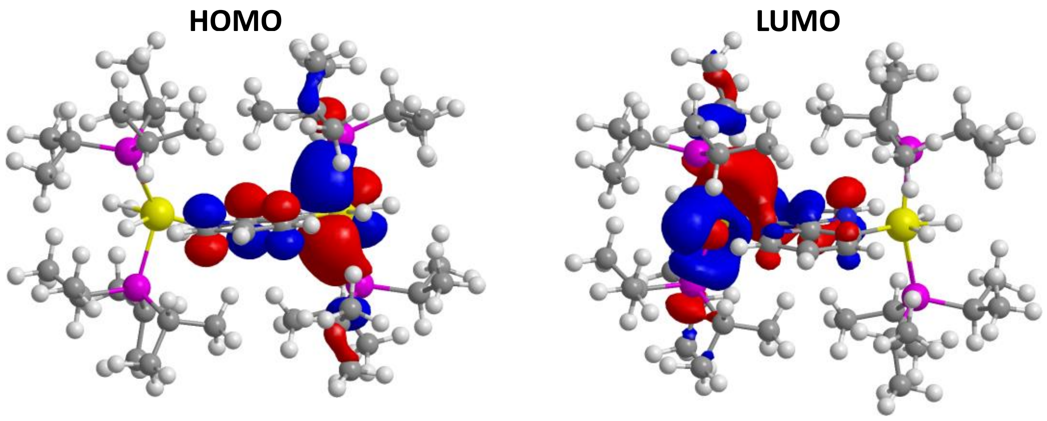

Figure S102. HOMO and LUMO of complex [**6**]$^{2+}$.

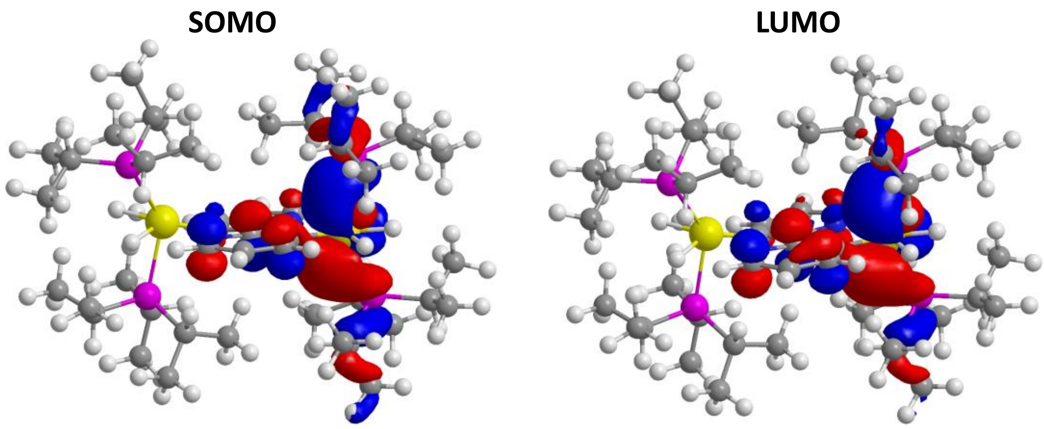

Figure S103. SOMO and LUMO of complex [**6**]$^{3+}$.

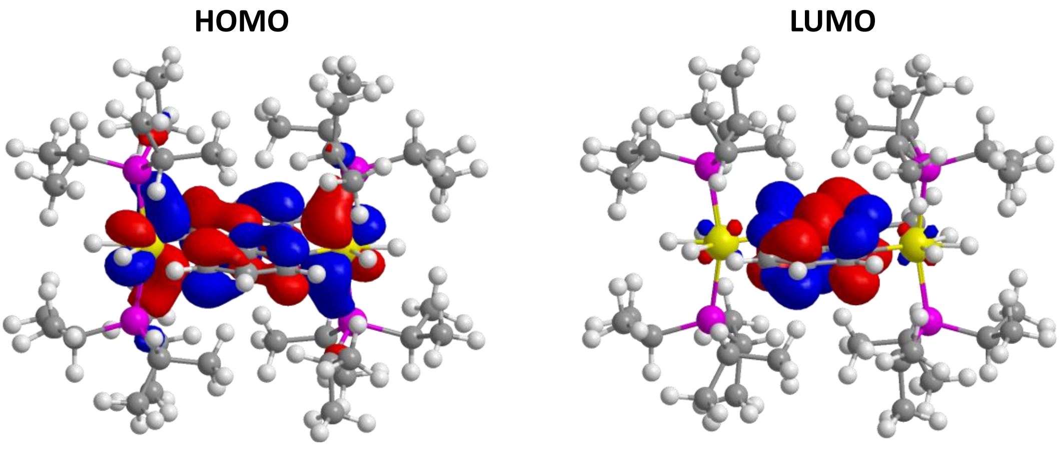

Figure S104. HOMO and LUMO of complex **7**.

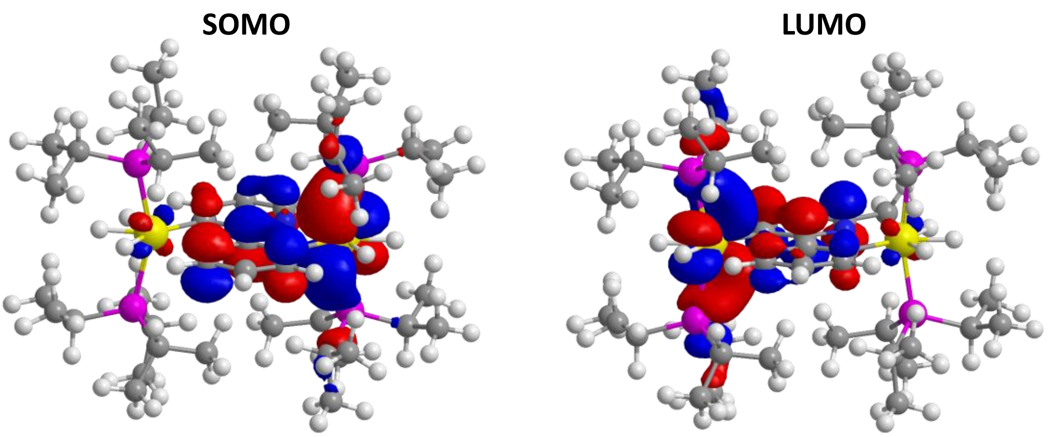

Figure S105. SOMO and LUMO of complex [**7**]$^{+}$.

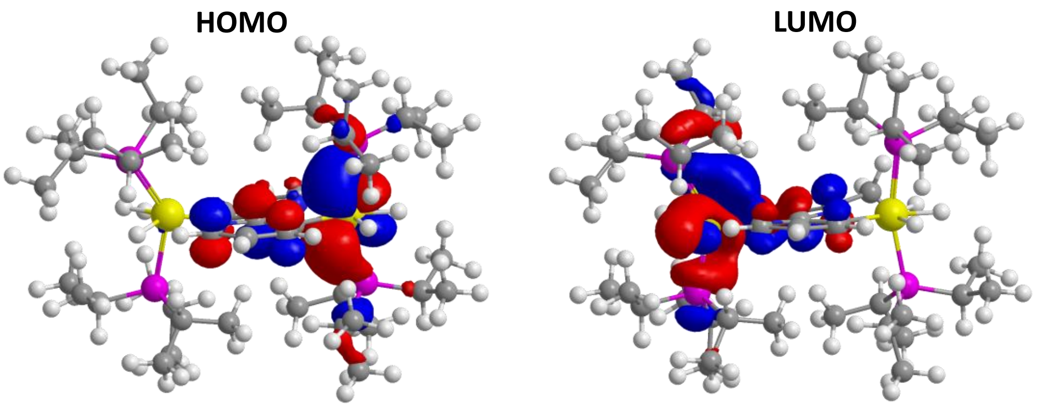

Figure S106. HOMO and LUMO of complex [**7**]$^{2+}$.

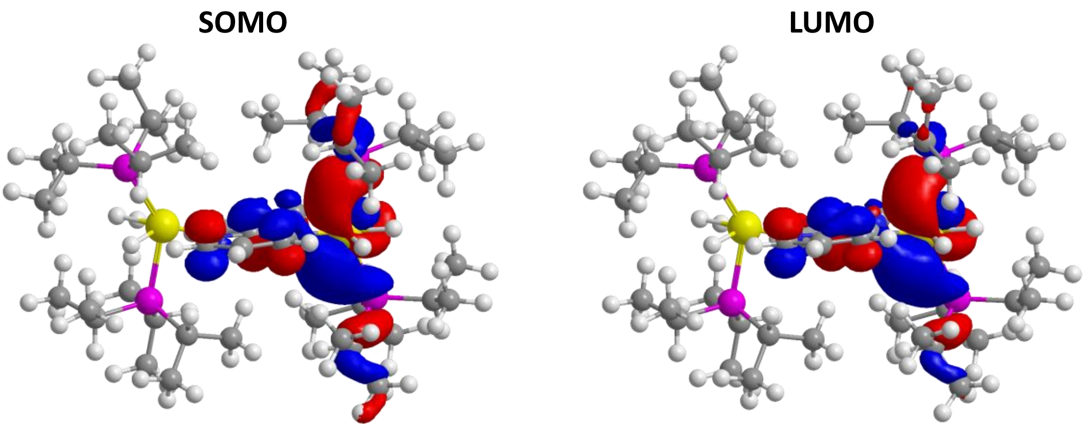

Figure S107. SOMO and LUMO of complex [**7**]$^{3+}$.

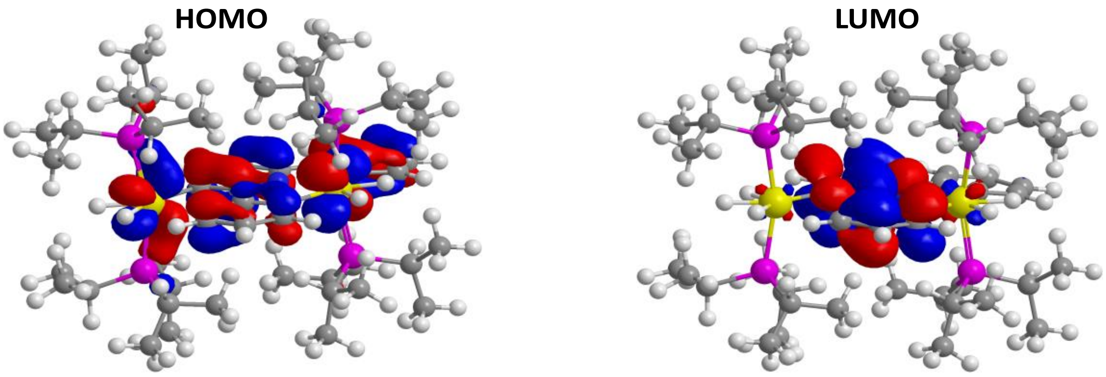

Figure S108. HOMO and LUMO of complex **8**.

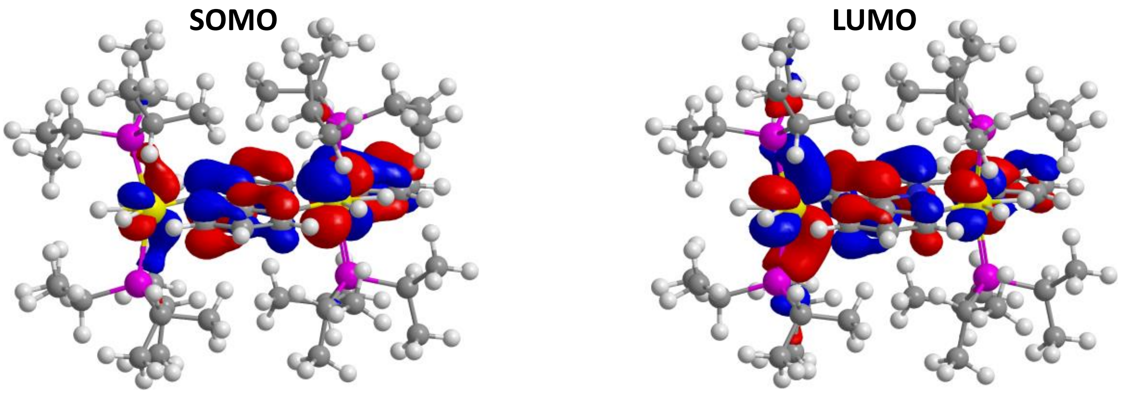

Figure S109. SOMO and LUMO of complex [**8**]$^{+}$.

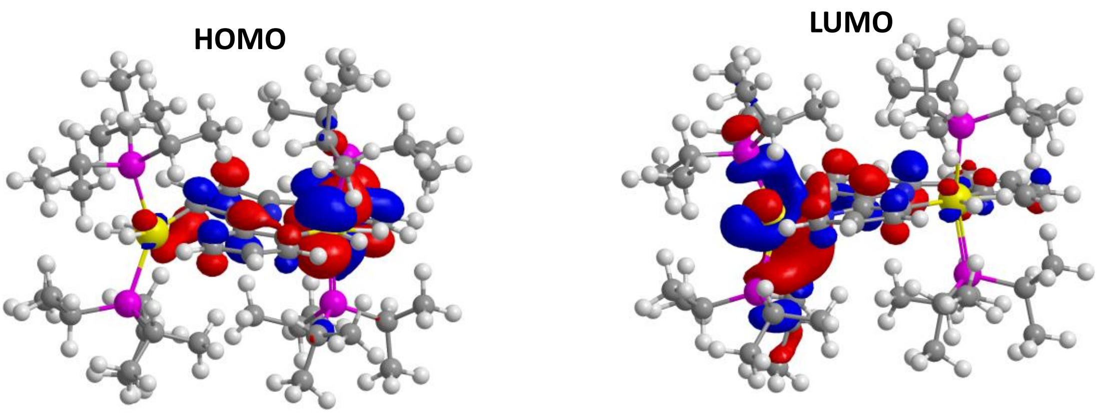

Figure S110. HOMO and LUMO of complex [**8**]$^{2+}$.

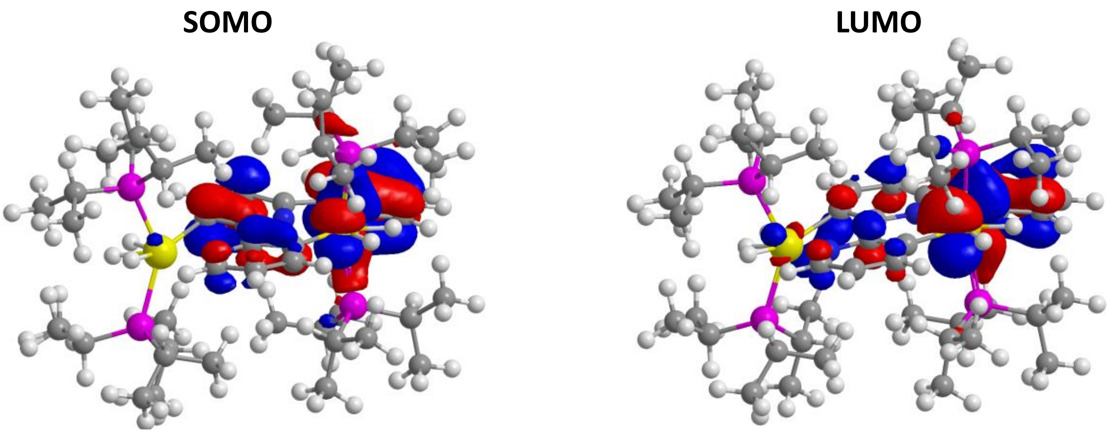

Figure S111. SOMO and LUMO of complex [**8**]$^{3+}$.

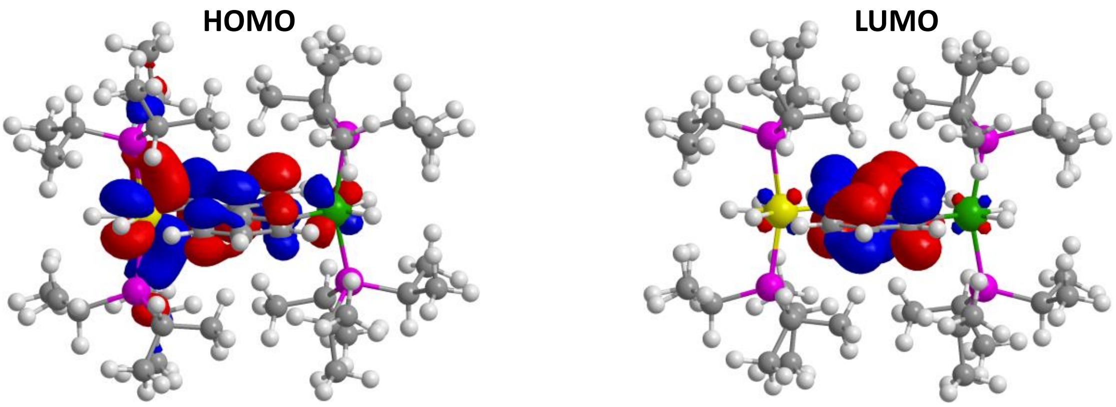

Figure S112. HOMO and LUMO of complex **10**.

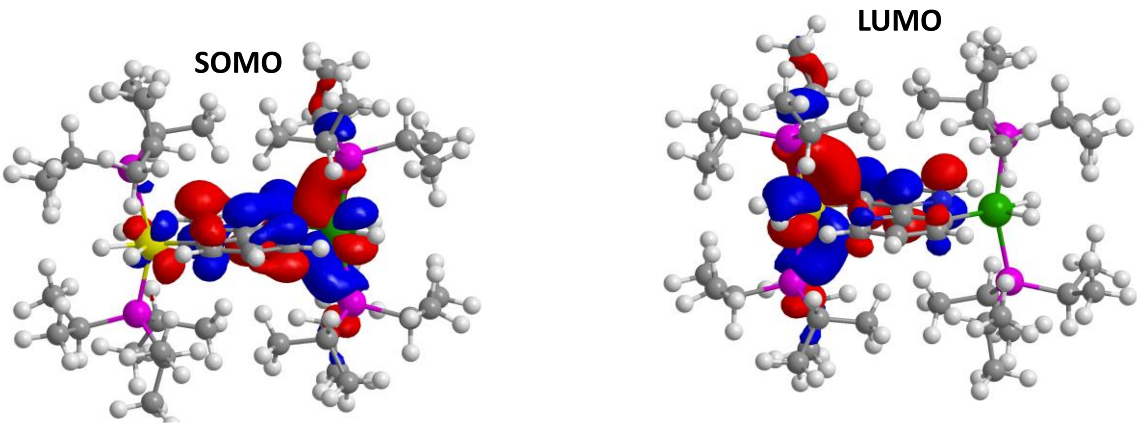

Figure S113. SOMO and LUMO of complex [**10**]$^{+}$.

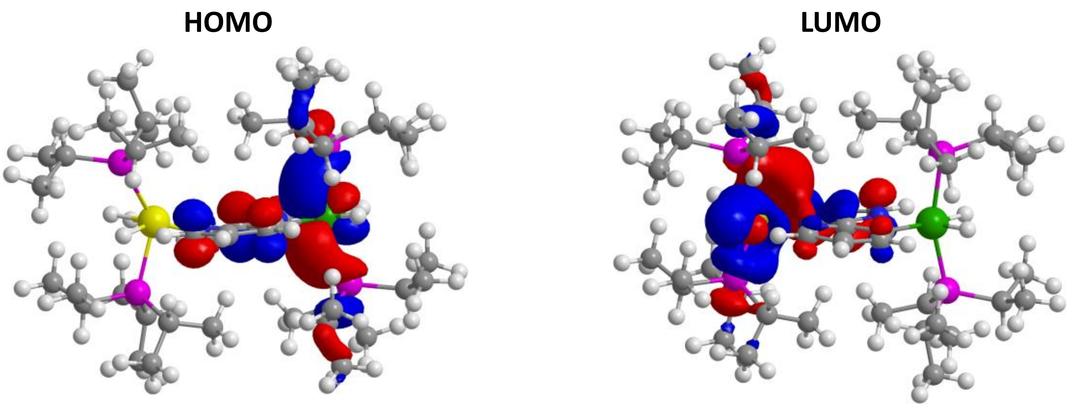

Figure S114. HOMO and LUMO of complex [**10**]$^{2+}$.

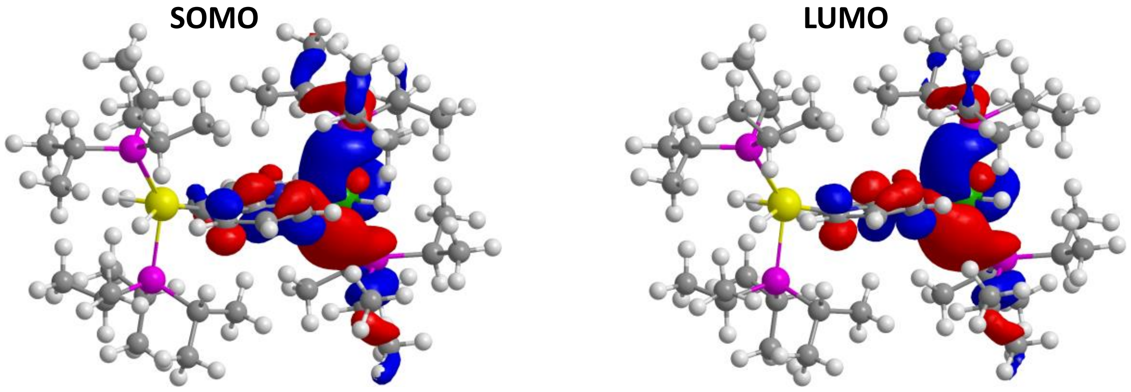

Figure S115. SOMO and LUMO of complex [**10**]$^{3+}$.

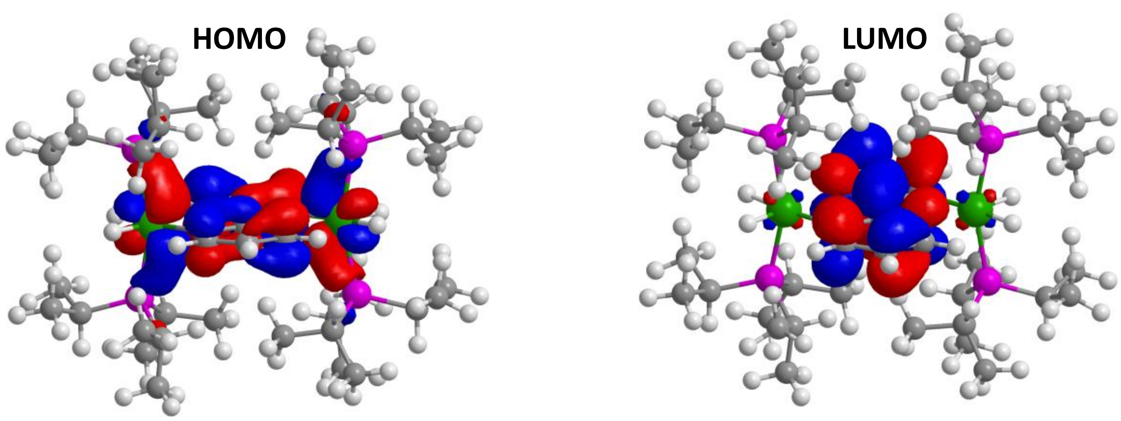

Figure S116. HOMO and LUMO of complex **11**.

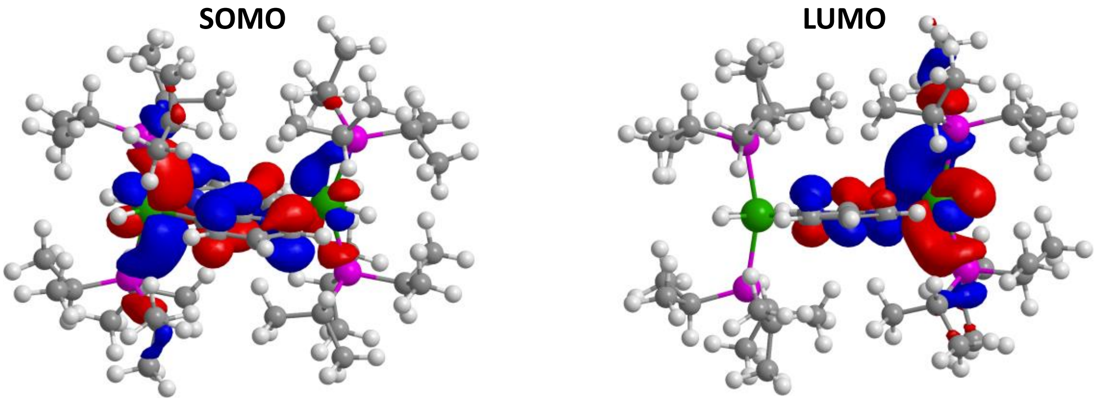

Figure S117. SOMO and LUMO of complex [**11**]$^{+}$.

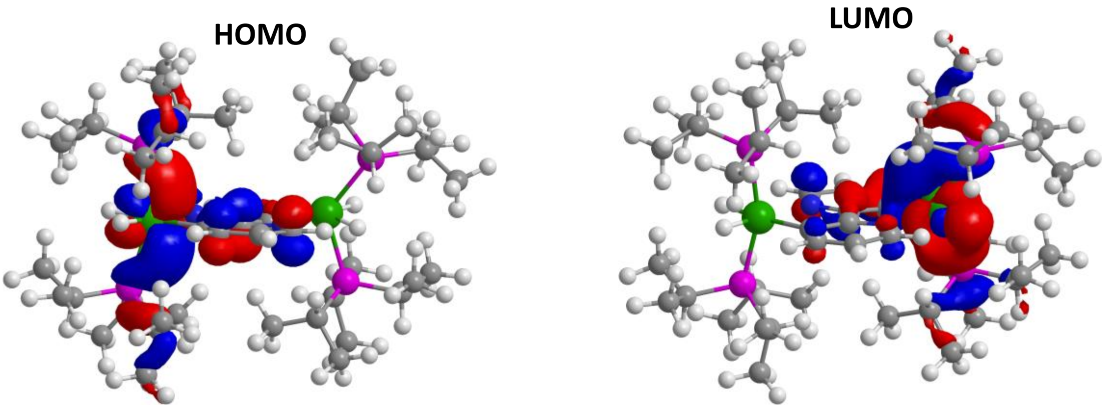

Figure S118. HOMO and LUMO of complex [**11**]$^{2+}$.

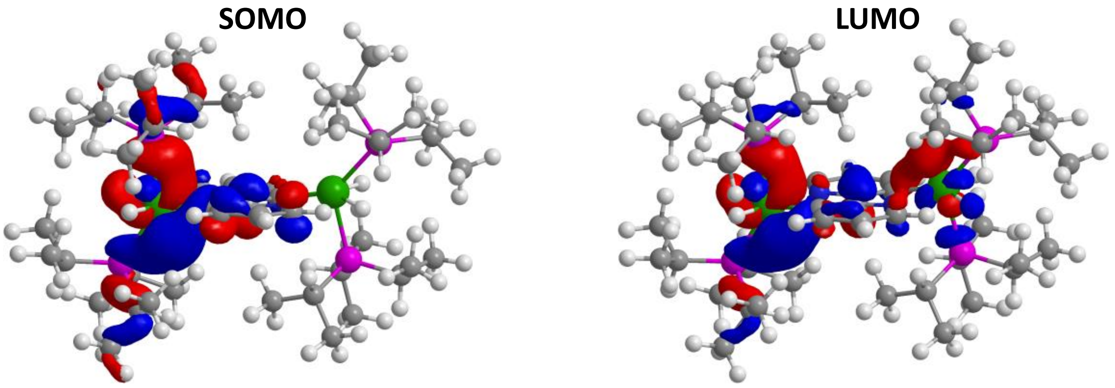

Figure S119. HOMO and LUMO of complex [**11**]$^{3+}$.

• Spin Density Distributions for the Optimized Triplet T_1 of 3-8, and 10-11.

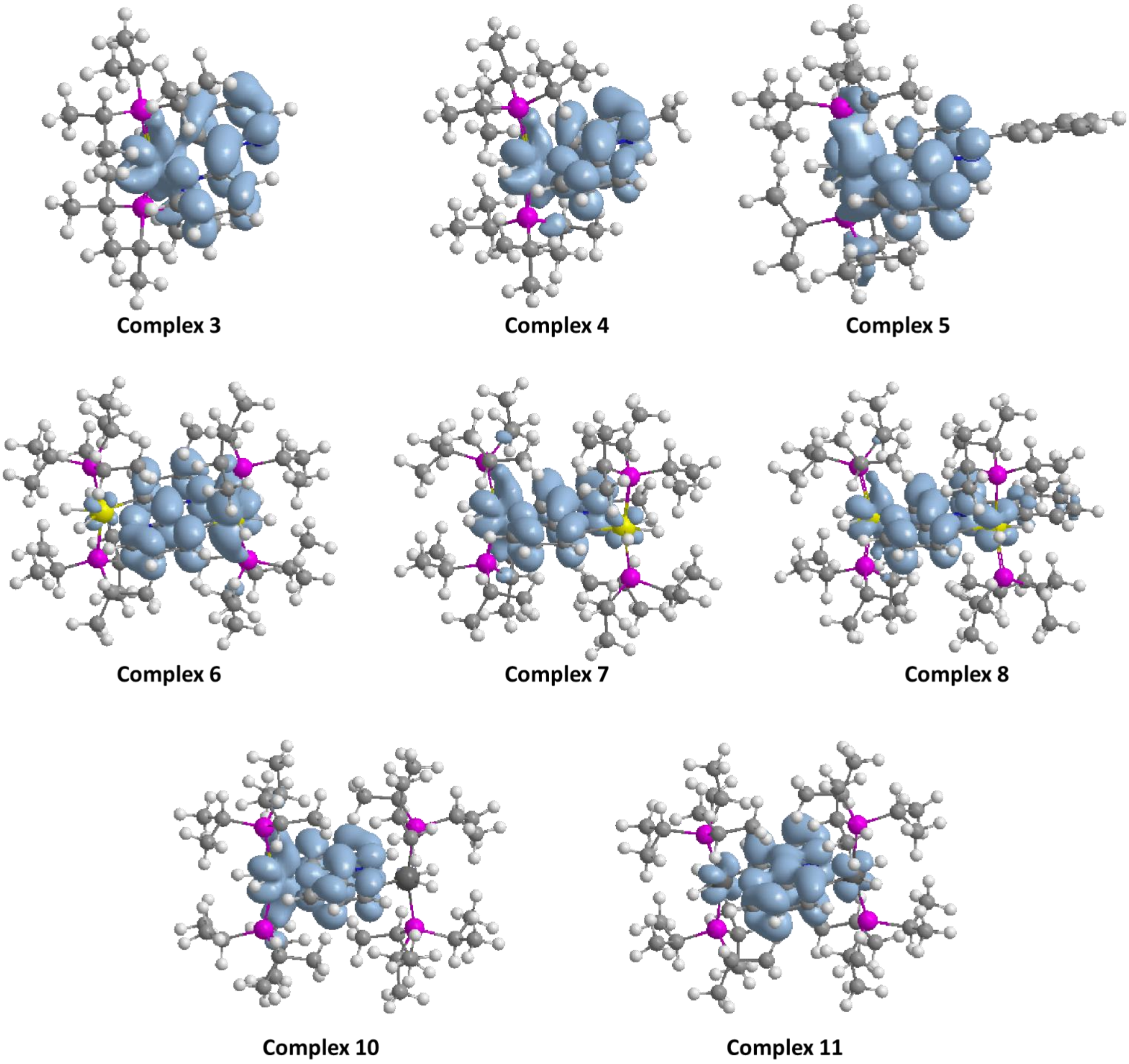

Figure S120. Spin density distributions for the optimized triplets T_1 of **3-8** and **10-11** (0.0004 isovalue).

● Spin Density Distributions for the Optimized Structure sof [6]$^+$, [7]$^+$, [8]$^+$, [10]$^+$ and [11]$^+$.

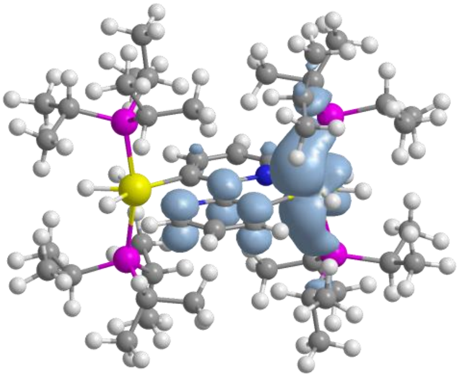

Complex [6]$^+$

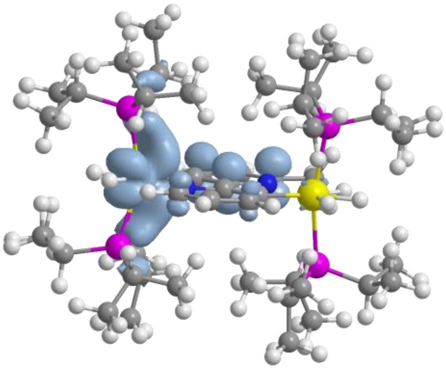

Complex [7]$^+$

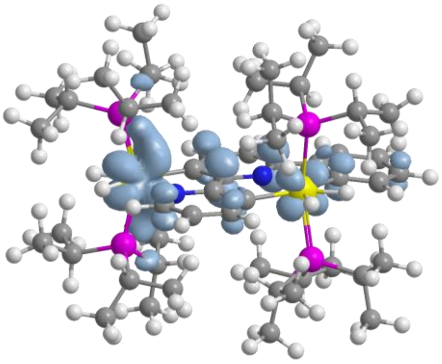

Complex [8]$^+$

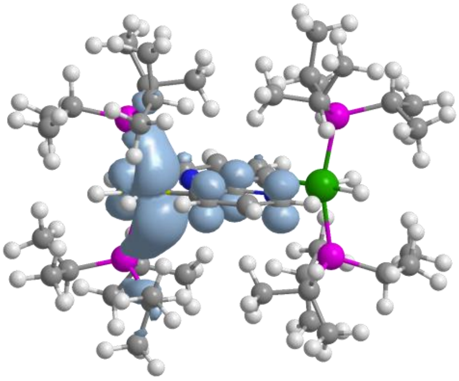

Complex [10]$^+$

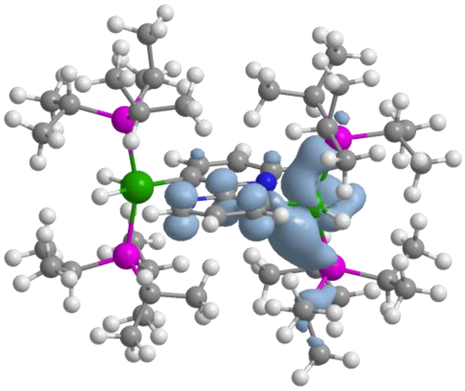

Complex [11]$^+$

Figure S121. Spin density distributions for the optimized structures of [**6**]$^+$, [**7**]$^+$, [**8**]$^+$, [**10**]$^+$ and [**11**]$^+$ (0.0004 isovalue).

● **References**

(1) Aracama, M.; Esteruelas, M. A.; Lahoz, F. J.; Lopez, J. A.; Meyer, U.; A. Oro, L.; Werner, H. Synthesis, Reactivity, Molecular Structure, and Catalytic Activity of the Novel Dichlorodihydridoosmium(IV) Complexes $OsH_2Cl_2(PR_3)_2$ (PR_3 = P^iPr_3, PMe^tBu_2). *Inorg. Chem.* **1991**, *30*, 288-293.

(2) Werner, H.; Schulz, M.; Esteruelas, M. A.; Oro, L. A. $IrCl_2H(P^iPr_3)_2$ as Catalyst Precursor for the Reduction of Unsaturated Substrates. *J. Organomet. Chem.* **1993**, *445*, 261-265.

(3) Kauffmann, T.; König, J.; Woltermann, A. Nucleophile Alkylierung un Arylierung des 2,2'-Bipyridyils. *Chem. Ber*. **1976**, *109*, 3964-3868.

(4) Serrano, E.; Martin, R. Nickel-Catalyzed Reductive Amidation of Unactivated Alkyl Bromides. *Angew. Chem. Int. Ed.,* **2016**, *55*, 11207-11211.

(5) Cancela, L.; Esteruelas, M. A.; López, A. M.; Oliván, M.; Oñate, E.; San-Torcuato, A.; Vélez, A. Osmium- and Iridium-Promoted C-H Bond Activation of 2,2'-Bipyridines and Related Heterocycles: Kinetic and Thermodynamic Preferences. *Organometallics* **2020**, *39*, 2102-2115.

(6) Blessing, R. H. *Acta Crystallogr.* **1995**, *A51*, 33. SADABS: Area-detector absorption correction; Bruker- AXS, Madison, WI, 1996.

(7) SHELXL-2016/6. Sheldrick, G. M. *Acta Cryst.* **2008**, *A64*, 112-122.

(8) (a) Lee, C.; Yang, W.; Parr, R. G. Development of the Colle-Salvetti correlation-energy formula into a functional of the electron density. *Phys. Rev. B* **1988**, *37*, 785-789. (b) Becke, A. D. Density-functional exchange-energy approximation with correct asymptotic behavior. *J. Chem. Phys.* **1993**, *98*, 5648-5652. (c) Stephens, P. J.; Devlin, F. J.; Chabalowski, C. F.; Frisch, M. J. Ab Initio Calculation of Vibrational Absorption and Circular Dichroism Spectra Using Density Functional Force Fields. *J. Phys. Chem.* **1994**, *98*, 11623-11627.

(9) Grimme, S.; Antony, J.; Ehrlich, S.; Krieg, H. A consistent and accurate ab initio parametrization of density functional dispersion correction (DFT-D) for the 94 elements H-Pu. *J. Chem. Phys.* **2010**, *132*, 154104.

(10) Gaussian 09, Revision D.01, Frisch, M. J.; Trucks, G. W.; Schlegel H. B.; Scuseria, G. E.; Robb, M. A.; Cheeseman, J. R.; Scalmani, G.; Barone, V.; Mennucci, B.; Petersson, G. A.; Nakatsuji, H.; Caricato, M.; Li, X.; Hratchian, H. P.; Izmaylov, A. F.; Bloino, J.; Zheng, G.; Sonnenberg, J. L.; Hada, M.; Ehara, M.; Toyota, K.; Fukuda, R.; Hasegawa, J.; Ishida, M.; Nakajima, T.; Honda, Y.; Kitao, O.; Nakai, H.; Vreven, T.; Montgomery, J. A.; Peralta, Jr., J. E.; Ogliaro, F.; Bearpark, M.; Heyd, J. J.; Brothers, E.; Kudin, K. N.; Staroverov, V. N.; Keith, T.; Kobayashi, R.; Normand, J.; Raghavachari, K.; Rendell, A.; Burant, J. C.; Iyengar, S. S.; Tomasi, J.; Cossi, M.; Rega, N.; Millam, J. M.; Klene, M.; Knox, J. E.; Cross, J. B.; Bakken, V.; Adamo, C.; Jaramillo, J.; Gomperts, R.; Stratmann, R. E.; Yazyev, O.; Austin, A. J.; Cammi, R.; Pomelli, C.; Ochterski, J. W.; Martin, R. L.; Morokuma, K.; Zakrzewski, V. G.; Voth, G. A.; Salvador, P.; Dannenberg, J. J.; Dapprich, S.; Daniels, A. D.; Farkas, O.;

Foresman, J. B.; Ortiz, J. V.; Cioslowski, J.; Fox, D. J. Gaussian, Inc., Wallingford CT, 2013.

(11) Andrea, D.; Häußermann, U. M.; Dolg, M.; Stoll, H.; Preuss, H. Energy-adjusted ab initio pseudopotentials for the second and third row transition elements. *Theor. Chim. Acta* **1990**, *77*, 123-141.

(12) Ehlers, A. W.; Bohme, M.; Dapprich, S.; Gobbi, A.; Hollwarth, A.; Jonas, V.; Kohler, K. F.; Stegmann, R.; Veldkamp, A.; Frenking, G. A set of f-polarization functions for pseudo-potential basis sets of the transition metals SC-Cu, Y-Ag and La-Au. *Chem. Phys. Lett.* **1993**, *208*, 111-114.

(13) (a) Hehre, W. J.; Ditchfield, R.; Pople, J. A. Self-Consistent Molecular Orbital Methods. XII. Further Extensions of Gaussian-Type Basis Sets for Use in Molecular Orbital Studies of Organic Molecules. *J. Chem. Phys.* **1972**, *56*, 2257-2261. (b) Francl, M. M.; Pietro, W. J.; Hehre, W. J.; Binkley, J. S.; Gordon, M. S.; DeFrees, D. J.; Pople, J. A. Self-consistent molecular orbital methods. XXIII. A polarization-type basis set for second-row elements. *J. Chem. Phys.* **1982**, *77*, 3654-3665.

(14) Marenich, A. V.; Cramer, C. J.; Truhlar, D. G. Universal Solvation Model Based on Solute Electron Density and on a Continuum Model of the Solvent Defined by the Bulk Dielectric Constant and Atomic Surface Tensions. *J. Phys. Chem. B* **2009**, *113*, 6378-6396.

(15) O'Boyle, N. M.; Tenderholt, A. L.; Langner, K. M. cclib: A Library for Package-Independent Computational Chemistry Algorithms. *J. Comput. Chem.* **2008**, *29*, 839-845
